# A Framework to Evaluate the Impact of Armourstones on the Chemical Quality of Surface Water

**DOI:** 10.1371/journal.pone.0168926

**Published:** 2017-01-06

**Authors:** Lars Duester, Dierk-Steffen Wahrendorf, Corinna Brinkmann, Anne-Lena Fabricius, Björn Meermann, Juergen Pelzer, Dennis Ecker, Monika Renner, Harald Schmid, Thomas A. Ternes, Peter Heininger

**Affiliations:** Federal Institute of Hydrology, Division G–Qualitative Hydrology, Am Mainzer Tor 1, Koblenz, Germany; Leibniz-Institut fur Pflanzengenetik und Kulturpflanzenforschung Gatersleben, GERMANY

## Abstract

Today, basic requirements for construction works include the protection of human health and of the environment. In the tension area between economic demands, circular flow economy and environmental safety, a link between the results from standardized leaching tests and the respective environmental quality standards must be created. To derive maximum release limits of metals and metalloids for armourstones in hydraulic engineering, this link is accomplished via a simple model approach. By treating natural materials and industrial by-products the same way, the article delivers an overview on the recent regulative situation in Europe as well as describes and discusses an innovative approach to derive maximum release limits for monolithic construction products in hydraulic engineering on a conceptual level. On a practical level, a list of test parameters is derived by connecting an extensive dataset (seven armourstone materials with five repetitions and 31 elements tested with the worldwide applied dynamic surface leaching test) with surface water quality standards and predicted no effect concentrations. Finally, the leaching tests results are compared with the envisaged maximum release limits, offering a direct comparison between natural materials and industrial by-products.

## Introduction

Several European directives set the frame for a sustainable management of water resources in the future. Examples are available from the nature conservation perspective [1,2], on chemicals [3] and for water resources [4–6]. Due to changes in the legislative structures in Europe, especially with respect to new requirements on the environmental acceptability of construction works and construction products, the legal limits in all EU member states need to be currently reevaluated. In this process also the German Technical Terms of Delivery for Armourstone (TLW, 2003; EU notification No. 2003/0362/D) must be revised [7]. The German Federal Institute of Hydrology (BfG) is assigned to establish a framework for the evaluation of environmental impacts and to derive maximum release limits (MRL) for armourstones in hydraulic engineering. The general procedure is under the aegis of the German Federal Waterways Engineering and Research Institute (BAW) which is responsible for the technical and physical evaluation of armourstones. The national administrative directive TLW with its four supporting documents is part of the collection Technical Rules and Standards Waterways (TR-W, 2015) [8]. It is based on the DIN EN 13383–1 (Armourstone—Part 1: Specification, 2002) [9] and -2 (Armourstone—Part 2: Test methods, 2002) [10] which are currently under revision at the EU level. With revision of the TLW several modifications in the requirements on armourstones are associated. Probably, the most pronounced change is an equal treatment of natural armourstones and armourstones derived from industrial processes (slags), since the current version of the TLW defines maximum release limits solely for the industrial by-products.

By March 2011, the Regulation No 305/2011 of the European parliament on harmonized conditions for the marketing of construction products was released (CPD—construction product directive: http://ec.europa.eu/growth/single-market/european-standards/harmonised-standards/construction-products/index_en.htm 2016/01/25). The directive delivers a framework for the harmonized EU market. It defines basic requirements for construction works—and not for the specific construction products. Though, in article 1 (3) of the CPD it is specified: “This Regulation should not affect the right of Member States to specify the requirements they deem necessary to ensure the protection of health, the environment and workers when using construction products.” and in article 3 basic requirements are defined in point 2 as: “The essential characteristics of construction products shall be laid down in harmonized technical specifications in relation to the basic requirements for construction works.” In addition, in chapter III the declaration of performance and CE (Conformité Européenne) marking are detailed and in Annex I surface waters are addressed via basic requirements for construction products with respect to hygiene, health and the environment: “The construction works must be designed and built in such a way that they will, throughout their life cycle, not be a threat to the hygiene or health and safety of workers, occupants or neighbors, nor have an exceedingly high impact, over their entire life cycle, on the environmental quality or on the climate during their construction, use and demolition, in particular as a result of any of the following: … d) the release of dangerous substances into groundwater, marine waters, surface waters or soil;…”.

This framework must be equipped on national basis with legal limits based on the recently released harmonized test methods. Decisions on potential release limits are influenced by the Construction Products Regulation (CPR; e.g., M 125 mandate to CEN/CENELEC). In this context, the execution of standardization work for harmonized standards on aggregates [11] as well as the environmental requirements based on the Water Framework Directive (WFD, 2000) [6] on the EU level and the German Surface Water Directive (OGewV) on the national level [12] are relevant legal basis. As a monolithic construction product for armourstones the DSLT (DIN CEN/TS 16637–2, dynamic surface leaching test) [13] is envisaged to be the relevant technical specification in the EU (cf. experimental section). Like in the EU, in the US a need for resource conservation is given [14] and to test natural and artificial products the EPA method 1315 (http://www.epa.gov/epawaste/hazard/testmethods/sw846/pdfs/1315.pdf 2016/01/25) is going to be used. The EPA 1315 [15] and the TS [13] are very comparable. Thus, the status of implementation as well as the general processes is comparable between the EU and the US. The former test system used for monolithic products DIN EN 1744–3:2002 will most likely be replaced [16]. As a consequence, in several countries the results from new standardized test systems must be linked in regulation to current environmental quality standards by adapted modelling approaches. Within this publication a framework designed to derive MRLs for metals and metalloid (metal(loid)s) is presented and discussed. Results for seven materials (five natural and two industrial by-products) are discussed and the challenges as well as the benefits in connection with the DSLT are addressed.

Information on the free market relevance and the test material selection is given in the Fig A and B in the S1 File.

## Methods and Model Conceptions

### Dynamic surface leaching test (DSLT)

In a nutshell, the DIN CEN/TS 16637–2:2014–11 was recently prepared by the Technical Committee CEN/TC 351 “Construction Products—Assessment of release of dangerous substances”. It specifies “a dynamic surface leaching test for determination of surface dependent release of substances from monolithic or plate-like or sheet-like construction products or granular construction products with low hydraulic conductivity under standardized conditions” [13]. In the test method the specimen is leached in a container under static conditions with deionized or ultrapure water over a total time period of 64 days (d). It consists of eight times sampling and full exchange of the eluent after 0.25, 1, 2.25, 4, 9, 16, 36 and 64 d, cf. Table A and Fig C in the S1 File. The method is very comparable to the EPA method 1315 [15]. Most important, in contrast to former technical guidelines such as DIN EN 1744–3 [16] the release is calculated based on the surface areas and not solely based on the masses which is well known to result, in connection with short testing times, in biased interpretation [17]. In this study the surface area was determined based on the aluminum foil method as published by Schmukat *et al*., 2013 [18]. For more details please refer to the technical specification [13] and the S1 File, page 4.

### Description of the model conception

**Fig 1:**
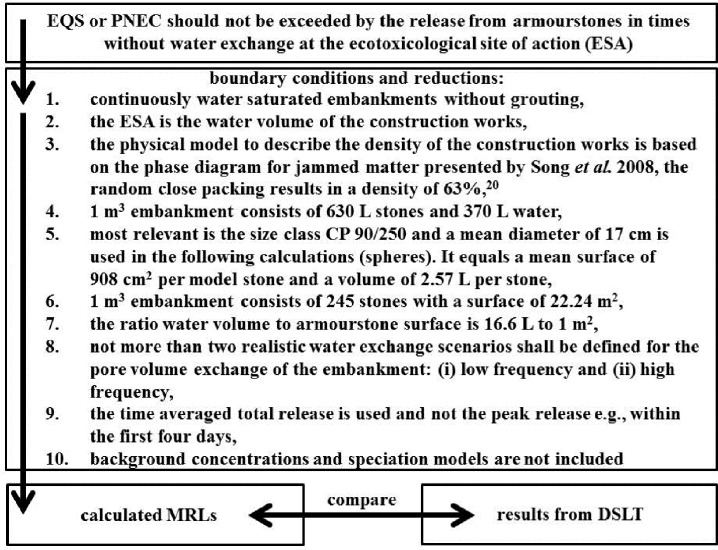
Overview on the model developed to connect environmental target values and results from DSLTs.

In order to establish a realistic link between environmental quality standards (EQS) or predicted no effect concentrations (PNECs) and the results from the DSLTs, a simple model has been developed by the authors. Fig 1 presents an overview on boundary conditions and reduction steps.

Reduction and calculation steps 1–10 are detailed as follows:

1. The most common use of armourstones is under full or intermitted water saturated conditions. Grouting reduces the available surface, but is a niche application compared to non-grouted embankments. In other applications armourstones are used as, e.g., pothole filling materials in river beds. Those applications are also covered by this scenario. The above water line parts of banks are temporary leached either by waves (from vessels or natural) or by rain and the runoff dewaters into the respective water body.
2. The ecotoxicological site of action (ESA) is defined as the water volume of the construction works where the EQS or PNEC should not be exceed. It can be expected that the pore water of the rip-rap, breakwaters, embankments, groin, etc., is the location with the highest concentrations of the substances released by the armourstones. It is also a habitat and shelter area of the aquatic communities and therefore the most exposed part of the biocenosis.
3. From the different available sphere packing models (hexagonal close packing, cubic close packing, primitive cubic packing etc.) the random closed packing is the model closest to the real structure of an embankment. The value (63%) published by Song *et al*. 2008 [19] for random closed packing is in very good agreement with values used by engineers from BAW and WSV in hydraulic engineering [20].
4. The values are derived from 63% packing density. 37% hollow space is given.
5. This is based on the analysis of calls for bids from the WSV and the authors’ experience.
6. Calculation: 630 L stones per m^3^ embankment / 2.57 L per stone = 245 stones per m^3^ embankment and 245 stones have a surface of 245 x 908 cm^2^ = 22.24 m^2^.
7. The ratio is a 370 L pore volume to 22.24 m^2^ (16.6 L: 1 m^2^).
8. The most important scenarios are: (i) canal = quasi static flow condition, (ii) lowland rivers = low dynamic and turbulence flow and (iii) mountain rivers and wave impacted coastal areas = high dynamic flow exchange. All other scenarios (e.g. harbors) can be grouped into one of the three situations. With respect to administrative needs, the authors adopted the engineers’ and WSV practitioners’ demands and reduced the concept to a maximum of two scenarios. Since the TLW addresses hydraulic engineering in waterways, the low dynamic and the high dynamic scenario fitting purpose for canals, major rivers and costal sites were chosen.
9. The DSLT delivers the opportunity to use the initial release (e.g., first four days), the mean or the sum release up to the test period of 64 d as a regulative basis in models. In the proposed model the option to integrate the peak release (wash off) as well as the midterm release was taken. Normally, armourstones own a “lifetime” of more than 50 years in banks what is addressed in best manner via the sum of analyte released during 64 d. For practical reasons and in a second step after initial testing the use of a reduced test time of nine days is envisaged.
10. The input requirement from the Federal Ministry of Transport and Digital Infrastructure was the creation of a robust and simple system for all German waterways. Hence, speciation based models (e.g., biotic ligand models—BLMs, e.g., Ruedel *et al*. 2015) [21] or surface water metal(loid)s background concentrations were not considered, since these are known to vary significantly within German waterways [22].

### Test parameters

In order to derive a parameter list in connection with the legal situation (WFD, OGewV and dangerous substances), six commercial products from the market survey, plus one additional industrial by-product, which is part of the TLW at the moment, were tested. An exhaustive screening of 31 metal(loid)s and nonmetals was initially performed in the eluates (Silver, Aluminum, Arsenic, Boron, Barium, Calcium, Cadmium, Cobalt, Chromium, Copper, Iron, Mercury, Potassium, Magnesium, Manganese, Molybdenum, Sodium, Nickel, Phosphorus, Lead, Sulphur, Antimony, Selenium, Silicon, Tin, Strontium, Thallium, Uranium, Vanadium, Tungsten, Zinc). The initial screening parameters were then reduced step-wise (Fig D in the S1 File).

In a first reduction step all analytes which were in more than 95% of the eluates below the limit of quantification (LoQ, blank+10σ criterion) were excluded. For these analytes no repeated quantifiable release from at least one of the seven materials was observed. The LoQs are part of S1 File, Tables B-H. As consequence, Ag, B, Hg, Tl and W were excluded from the parameter list (data not shown). In a second step analytes that may help to understand release mechanisms but display no relevance with respect to the ecotoxicological assessment in the given concentration in surface waters (Ca, Fe, K, Mg, Na, P, S and Si, data not shown) were omitted. After this reduction 18 elements remained (Al, As, Ba, Cd, Co, Cr, Cu, Mn, Mo, Ni, Pb, Sb, Se, Sn, Sr, U, V and Zn, S1 File, Tables B-H). The parameter list was then subjected to further reduction steps based on the ecotoxicological parameter evaluation.

Subsequently, the analytes with potential ecotoxicological relevance were identified. Due to the fact that different species may show different sensitivities to individual contaminants the results of the most sensitive species were used to derive threshold values. This was carried out by an ecotoxicological risk assessment that calculates the PEC/PNEC ratio, where PEC is the predicted environment concentration and PNEC is the concentration where adverse effect on the environment are not expected (predicted no effect concentration) [23]. If the ratio is above 1, adverse effects cannot be excluded. The PNEC is extrapolated from the available ecotoxicological data mostly with the consideration of an assessment factor (AF, if the data is not sufficient to account for the confidence of the toxicity data and due to the restricted exposure of the laboratory bioassays). Further information on the derivation of the PEC, PNEC and environmental quality standards can be found in a technical guidance document of the European Commission [23].

For the remaining metal(loid)s the EQS and PNEC concentrations were compiled as presented in Table 1. Primarily, the threshold values (annual average EQS concentrations for the water phase (AA-EQS—JD-UQN)) of the decree of the EU water framework directive implementation in Germany (OGewV) were applied (As, Cd, Ni, Pb and Se) [12]. For Cd different thresholds are provided in the OGewV depending on the water hardness onsite. The water hardness of the waterways in Germany can be classified to hardness level 5 (cf. LUNG MV 2012) [24], therefore the corresponding value was applied. For Ni and Pb the “added risk” approach has to be applied, which takes the background levels onsite into account. Due to the fact that the emitted concentrations from the armourstones pose the additional contaminant loads for the water phase at the ESA, these values were adopted directly. For Ba, Sn and V the environmental quality standard recommendations of a research project commissioned by the German Federal Environment Agency (UBA) were adopted. With respect to the ecotoxicological data available for these metals an assessment factor of AF = 50 was applied to derive the quality threshold concentration [25,26]. These thresholds are based on a detailed literature research and a subsequent ecotoxicological risk assessment [27]. The thresholds for Cr (PNEC value Cr(VI) 3.4 μg/L; Cr(III) soft water 4.7 and hard water 13 μg/L; the Cr(VI) value was used) is based on a risk assessment in a report of the European Union [28]. The proposed thresholds from Maycock *et al*. (2007) [29], Wenzel *et al*. (2015) [30] and Nendza (2014) [31] are comparable. For Cu the risk assessment provided to European Chemical Agency (ECHA) by the European Copper Institute (ECI 2008) for the European Union was used [32]. The Zn PNEC was derived from the recommended annual average for freshwater from several studies commissioned by the UBA [27,30,33]. Similar values were determined by an UK advisory group (UKTAG 2012) [34,35]. Comparable values were also derived in assessments of the Joint Research Centre of the European Commission (EC 2010) [36] and the Norwegian Institute for Water Research (NIVA 2007) [37]. For Al, Co, Mn, Mo, Sb and Sr the databases currently available are not sufficient to derive reliable PNECs and hence, these parameters were excluded from consideration (reduction criteria 3 in S1 File, Fig D, data presented in S1 File, Tables B-H). In addition, in a fourth reduction step Se and U were removed from the parameter list since these metal(loid)s are either not covered by the WFD [6], the OGewV [12] or the construction product requirements [11] (Uranium) or the experimentally observed release was insignificantly above the limits of quantification (LoQs, Se—only one tested Greywacke armourstone of five was above LoQ, all others negative compliance status).

**Table 1:** MRLs for two different scenarios based on the EQS or PNEC and the model deduced as explained in the previous section. Italic = EQS, rest = PNEC as explained in the previous paragraph (*based on class 5, water hardness ≥ 200 mg CaCO_3_/L), **as Cr(VI), ***as explained in Fig 1).

Analyte	*As*	Ba	Cd*	Cr**	Cu	*Ni*	*Pb*	Sn	V	Zn	Calculation***
**EQS or PNEC as max. release at the ESA [μg/L]**	*1*	60	*0*.*25*	3.4	7.8	*4*	*1*.*2*	3.5	2.4	11	
**MRL low frequency exchange 24 h [mg/m**^**2**^**/64d]**	1.1	64	0.3	3.6	8.3	4.5	1.3	3.7	2.5	12	= EQS or PNEC [μg/L]/1000*16.6 [m^2^/L] *64 [d]
**MRL high frequency exchange 6 h [mg/m**^**2**^**/64d]**	4.2	255	1.1	14	33	17.2	5.1	15	10	46	= MRL 24 h*4

However, the parameter list is not closed. If a significant release becomes visible or new findings indicate a potential concern for a certain product during authorization, new parameters can easily be included and monitored in future testing and assessments.

In addition to parameters such as metal(loid)s, the previous version of the TLW also defined maximum pH and conductivity changes. From the authors’ experience the conductivity is a good sum parameter for the total release of ions, but insignificant in the specific regulation context. In contrast to this, pH is relevant. Certain materials such as steel slag (Linz Donawitz slag, LDS) are known for their potential to cause adverse environmental effects in surface water by changing the pH value. Hence, it is advisable that the leachate must not exceed pH 10 or fall below pH 4 at any time in DSLTs.

### Calculation of the maximum release limits

In Table 1, the MRLs derived from the simple model approach from Fig 1 and the EQS or PNECs from the previous section are presented. For the high frequency exchange scenario six hour calculations are chosen. The value for 6 h equals 50% of the time between two high tides at the North Sea coast in Germany. Since the exact hydraulic conditions in different major rivers and different coastal areas vary significantly, the authors are of the opinion that six hours is a realistic and balanced value with respect to the environmental conditions and the limited amount of quantifiable data available. In the low frequency scenario a water exchange is carried out every 24 h. This is based on the water exchange scenario in the canal banks, solely caused by vessel traffic (no vessel traffic during public holidays). A comprehensive overview on this issue is delivered by the PIANC–InCom WG 27 report 2008 which focusses on the environmental impact of vessels and describes also the basic physical forces from wave fields on river and canal banks [38].

## Results and Discussion

### Maximum release limits

The different steps of the simple model approach of Fig 1 (cf. also bullet points 1–10) are based on scientific evidence (point 2 and 3), practical experience in hydraulic engineering (1, 5 and 8), calculation (4, 6 and 7), input requirements from the WSV and the Ministry (8 and 10) as well as in one case on the long term character of the intended use of the materials (9). By taking the benthic biocenosis as primary subject of protection, several secondary alignments are connected. First of all, annual mean values of the EQS (and PNECs if no formal EQS available) referring to a water body are applied to the pore water of the construction works as one part of the water body. Furthermore, using only the peak release of the first ~1–4 days is not advisable. For example during building activities where significant amounts of the banks are equipped with armourstones (e.g., in canal, on new construction sites and in case of bigger maintenance activities) the benthic community is not present in the recently build construction works and the water exchange is very high, caused by the vessels. For smaller and midsized maintenance activities the effect of potential contaminant release at the beginning of the exposure is less pronounced, also due to their size in comparison to the water body. However, if a peak release (wash off) causes a short term exceedance of the EQS or PNEC in the embankment in the first days after placing, it is taken into account. In addition the derivation of EQS and PNEC values are based on long term exposures, higher concentrations in short termed exposures are also taken into account.

Considering the timescales of the water exchange, the low frequency exchange is based on the scenario with no or very little traffic (e.g., in winter or on holidays) and is derived from experience. An issue for further discussions between different interest groups might evolve, since 24 h is not a worst case scenario with respect to shipping restrictions in winter.

### Comparison of results from the DSLTs and of the envisaged MRLs

In S1 File, Fig I-R the results from the DSLTs are presented in the common log/log form [39]. A major difference between the natural products and the industrial by-products is also visible in Table 2 and with respect to the leaching range. The release from the natural stones varies between μg/m^2^ to mg/m^2^, whereas for some elements (Cu and Zn from CUS and V from LDS) hundreds of mg/m^2^ was released from the industrial by-products. Detailed descriptions of the mineral phases that contribute to the metal(loid) release from CUS are available in the publications authored by Schmukat *et al*. [18, 40–41], Hullebusch *et al*. [42] and Duester *et al*. [43]. The release over 64 days is dominantly from sulphides and the sulphur content of copper slags may vary significantly, depending most of all on the processed concentrates and on the melting process. For the release from LDS the most significant factor is the CaO content of the slag. With its hydration a co-release of vanadium is given. For other metals that have also high total content in the slag, an insignificant release from LDS is visible. A good example is chromium, which is bond in the less soluble spinel phases.

**Table 2:** Mean release values [mg/m^2^/64d] of n = 5 DSLTs (Lab2; S1 File, Table B-H, SD = standard deviation of independent tests) compared to MRLs. Values >low frequency exchange MRL = italic, underlined and light grey; values > high frequency exchange value 6 h = bold, underlined and dark grey, *EQS Cr(III) **<LoQs; the LoQs are given in S1 File, Tables B-H for the respective elements.

Analyte	As	SD	Ba	SD	Cd	SD	Cr*	SD	Cu	SD	Ni	SD	Pb	SD	Sn	SD	V	SD	Zn	SD
**MRL 24 h**	1.1	-	64	-	0.3	-	3.6	-	8.3	-	4.5	-	1.3	-	3.7	-	2.5	-	12	-
**MRL 6 h**	4.2	-	255	-	1.1	-	14	-	33	-	17	-	5.1	-	15	-	10	-	46	-
**Karbon Quartzite**	0.03	0.01	0.9	1.2	**	-	0.1	0.06	**	-	0.07	0.05	**	-	**	-	**		0.3	0.06
**Basalt**	**	-	**	-	**	-	**	-	0.4	0.3	0.03	0.002	0.03	0.01	**	-	0.14	0.11	0.4	0.3
**Greywacke**	0.1	0.1	4.3	2.3	**	-	0.02	0.01	0.03	0.01	0.1	0.04	0.04	0.04	0.01	0.001	0.01	0.001	1	0.5
**Granodiorite**	0.1	0.1	0.7	0.1	**	-	**	-	0.06	0.04	0.1	0.1	0.02	0.001	**	-	0.03	0.01	0.4	0.2
**Granite**	0.4	0.4	0.2	0.4	**	-	0.01	0.002	0.3	0.01	0.02	0.01	0.01	0.001	**	-	0.02	0.004	3.5	2.4
**CUS1**	0.7	0.2	3.4	0.5	0.2	0.2	**	-	**37**	2.3	3.6	3.2	0.7	0.4	**	**	**	-	**51**	39
**CUS2**	0.3	0.2	1.1	0.4	0.3	0.12	**	-	**95**	31	*6*.*9*	6.1	**27**	22	0.1	0.002	**	-	*31*	13
**CUS3**	0.8	0.5	1.1	0.4	0.1	0.03	**	-	**74**	24	2.5	1.7	0.4	0.3	**	-	0.04	0.001	*13*	4
**LDS**	**	-	2.8	1.4	**	-	0.6	0.2	0.2	0.3	0.1	0.1	0.01	0.0001	**	-	***51***	17	0.4	0.04

With the log/log figures it is possible to easily check for elements and materials that have a potential for a high release into the environment. For CUS these are Cu, Zn and Pb and for LDS it is V. Regarding the release over a time period of 64 d none of the natural stones display a noticeable release curve. The lowest overall release was found for Basalt, Granodiorite and Karbon Quartzite which did not exceed a sum of 1 mg/m^2^ in 64 d for all elements of the parameter list.

Comparing the slope lines in the upper right corner of each graph as a rough indicator for different release mechanisms (cf. S1 File, Fig I-R) it becomes visible that the release of most of the analytes in all materials is based on diffusion controlled processes. Only few analytes show a tendency to be released via dissolution of mineral phases (Basalt: Cu, Pb, Zn; Granite: Ba, U, Zn; Granodiorite: As and Ba; Greywacke: V and Zn; Karbon Quartzite: Cr; CUS: As and Pb; LDS: Mo—the increased concentration of Cu in the last sample was verified, but must be treated as an outlier). Respective results have been discussed for Cu and CUS from sulphides elsewhere [18]. In Table 1 the calculations and the MRLs for the low frequency scenario and the high frequency scenario are presented. Table 2 compares the MRLs calculated based on the model displayed in Fig 1 and the released concentrations from S1 File, I-R Fig and Tables B-H. It becomes visible that all natural materials tested are far from reaching the MRLs derived from the proposed 64 d model. The four charges of the industrial by-products do not fulfill the MRL criteria (CUS: Cu, Pb and Zn; LDS: V and the pH (S1 File, Table I)).

Taken together, the proposed parameter list is: Arsenic, Barium, Cadmium, Chromium, Copper, Nickel, Lead, Tin, Vanadium, Zinc and the pH value. If needed (e.g., a new material is accredited) the list can be extended and new parameters can easily be included via the proposed model.

## Conclusions

Even though the TS [13] can still be improved in some minor aspects (detailed in the S1 File, page 151 and tables J-L) the use of the DSLT is a significant progress towards a more comprehensive evaluation of monolithic construction products. In contrast to the formerly used DIN EN 1744–3 (based on the release per mass and integrating only wash off dominated 24 h) it enables the integration of longer release periods in the basic characterization and thereby the identification of basic release mechanisms of a multitude of analytes from a certain product. In addition, the results can be displayed and interpreted based on the surface, the mass and on different time intervals, delivering a better knowledge on the release, a higher degree of freedom to national regulation and a better reliability for customers. In conclusion, whereas the DIN EN 1744–3 [16] left regulators and customers with a high degree of uncertainties, the DIN CEN/TS 16637–2 [13] enables to create data bases on different natural materials and industrial by-products with a high degree of reliability. This reliability in data bases is urgently needed with respect to new challenges in the construction sector, e.g., close loop recycling management. After the initial testing, the presented framework can easily be simplified by reducing the testing times in order to reduce manufacturers’ and costumers workloads and costs. With respect to the presented leaching test results, for materials that fulfill a nine days DSLT MRLs, a reduced test time is sufficient. Even though a certain accumulation of regulated dangerous in sediments cannot be avoided [40] the authors are from the opinion the proposed model and MRLs are sufficient to protect biota, surface water and sediments.

The aim of the publication is to support a progress in the international discussion on the urgent matter: How to connect results from standardized test systems with environmental quality demands? By delivering a starting point for further discussions the authors are aware of the fact that the proposed simple model for armourstones in hydraulic engineering may be changed and adopted. This is inherent to the system since parameters like the water exchange time intervals are not derived from scientific basis, but from experience and regulation demands (e.g., maximum of two scenarios).

The comprehensive dataset of seven armourstone materials tested with the DSLT is for the BfG, and hopefully also for others, a starting point for an open source database on construction products. Such a database open to producers, costumers and regulators worldwide may significantly combat the nowadays visible fragmentation of efforts in the international construction sector via delivering a common information and decision basis.

## Supporting Information

S1 File **Fig A** and **B** show the results of two armourstone surveys performed by the BfG. **Table A** and **Fig C** basic information on the DSLT is presented. Information on chemicals and materials used in the study as well as on analyzes performed is given on page fou. **Fig D** shows the stepwise reduction of the initial parameter list. The full DSLT dataset with seven stones is presented in **Tables B-H**. All calculations (release_max_ and release_min_) are based on the TS. In **Table I** conductivity and pH values are presented. **Table J** compares the nine days MRLs with the DSLT results. An overview on the evaluation of the blank criteria is given in **Tables K and L**. **Figs I-R** show the log/log graphs for CUS1-3, Granite, Granodiorite, Greywacke, Basalt, Karbon Quartzite and LDS. A chapter on “DSLT practical considerations and potential improvements of potential following technical specification” is also given.
(PDF)
