# Supplementary material for: A Framework to Evaluate the Impact of Armourstones on the Chemical Quality of Surface Water
Source: PLoS One. 2017 Jan 6;12(1):e0168926. doi: 10.1371/journal.pone.0168926 (PMC5217939; doi:10.1371/journal.pone.0168926)
Supplement: S1 File — Fig A and B show the results of two armourstone surveys performed by the BfG. Table A and Fig C basic information on the DSLT is presented. Information on chemicals and materials used in the study as well as on analyzes performed is given on page fou. Fig D shows the stepwise reduction of the initial parameter list. The full DSLT dataset with seven stones is presented in Tables B-H. All calculations (releasemax and releasemin) are based on the TS. In Table I conductivity and pH values are presented. Table J compares the nine days MRLs with the DSLT results. An overview on the evaluation of the blank criteria is given in Tables K and L. Figs I-R show the log/log graphs for CUS1-3, Granite, Granodiorite, Greywacke, Basalt, Karbon Quartzite and LDS. A chapter on “DSLT practical considerations and potential improvements of potential following technical specification” is also given. (PDF) [file pone.0168926.s001.pdf]

Supporting information

A framework to evaluate the impact of armourstones on the chemical quality of surface water

Lars Duester, Dierk-Steffen Wahrendorf, Corinna Brinkmann, Anne-Lena Fabricius, Björn Meermann, Juergen Pelzer, Dennis Ecker, Monika Renner, Harald Schmid, Thomas A. Ternes and Peter Heininger

Federal Institute of Hydrology, Division G – Qualitative Hydrology,

Am Mainzer Tor 1, 56068 Koblenz, Germany.

Number of pages:	163
Number of figures:	13
Number of tables:	12

Per year quantities of armourstones obtained by the WSV In order to achieve an overview on the market diversity, in two surveys the armourstone purchases of the German Federal Waterways and Shipping Administration (WSV) in 2010 – 2012 and in 2012 – 2014 were requested from the administrations and summarized. In the first survey period (Fig A) the actually installed armourstone quantities and their origins were inquired. In order to verify these results, in a second survey period (Fig B) calls for tenders from the WSV were collected from two different online tender portals (national and EU based). After the bid period, the respective departments of the WSV were contacted and the acquired products were listed.

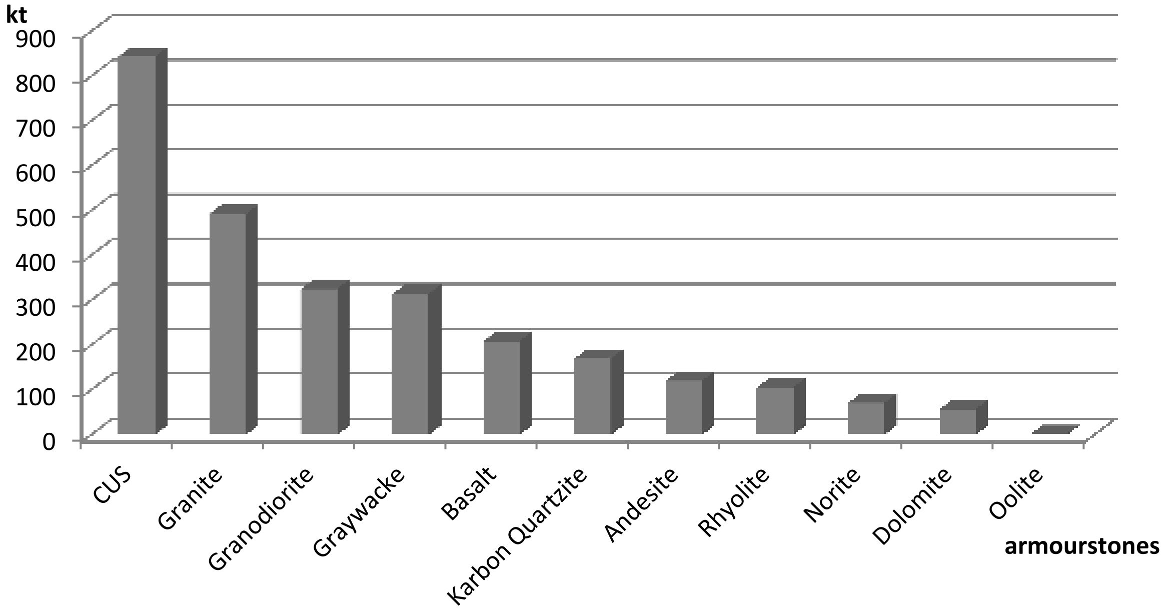

Fig A. Armourstones [kilo tons] installed by the WSV in German waterways between 01.2010 and 06.2012 (CUS = copper slag/product name: iron silicate stone). The names of the materials are as given by the distributors with exception of CUS.

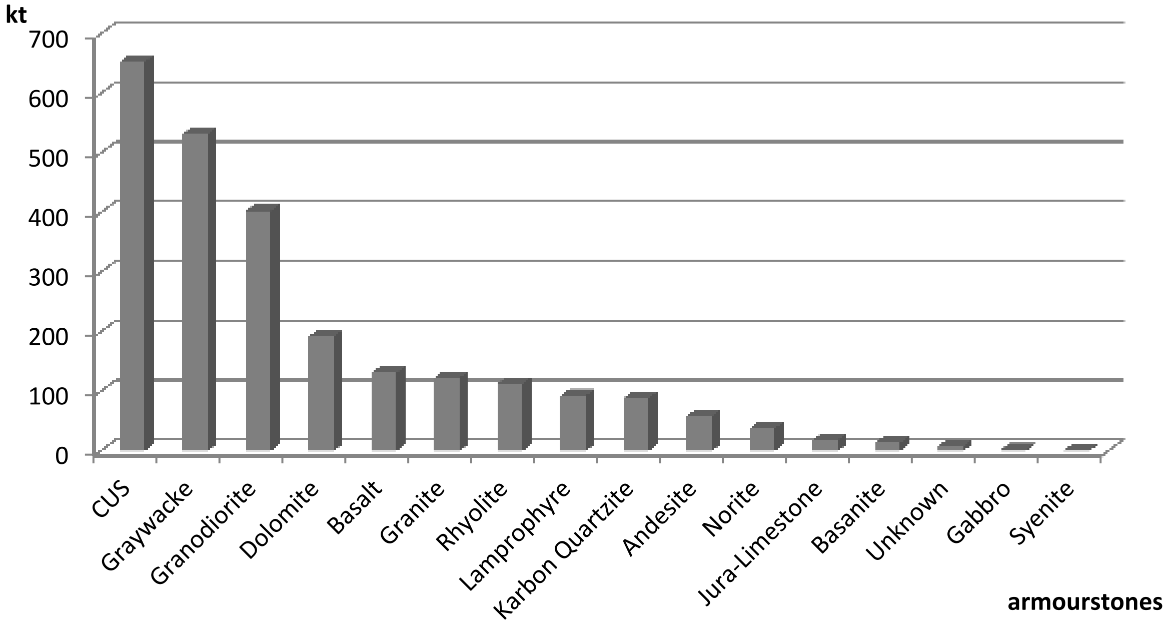

Fig B. Armourstones [kilo tons] purchased between 04.2012 and 06.2014 by the WSV. The names of the materials are as given by the distributors with exception of CUS.

The total amounts of armourstones purchased by the Federal Water and Shipping Administration (WSV) in Germany (two surveys, two years each) are very comparable (year 2010-12 and 2012-14). In both periods about 1.8 million tons of natural armourstones and between 0.6 and 0.8 million tons copper slag (CUS) were obtained. CUS was the only industrial by-product purchased. By widening the perspective on amounts potentially obtained by other customers (e.g., Federal States, communities and the industry/private sector like port authorities), only for Germany an annual volume of more than 20 million € can be expected. Based on two surveys, the most important armourstone materials were selected for testing with the DSLT method. The samples were obtained either directly on-site at the producer facilities or at the WSV storage facilities.

Table A. Time schedule for the water exchanges in DSLTs.

Step/ Fraction	Time [d]	Variation accepted	Time sum [d]
1	0.25	± 15 min	0.25
2	0.75	± 15 min	1
3	1.25	± 45 min	2.25
4	1.75	± 75 min	4
5	5	± 75 min	9
6	7	± 75 min	16
7	20	± 7 h	36
8	28	± 12 h	64

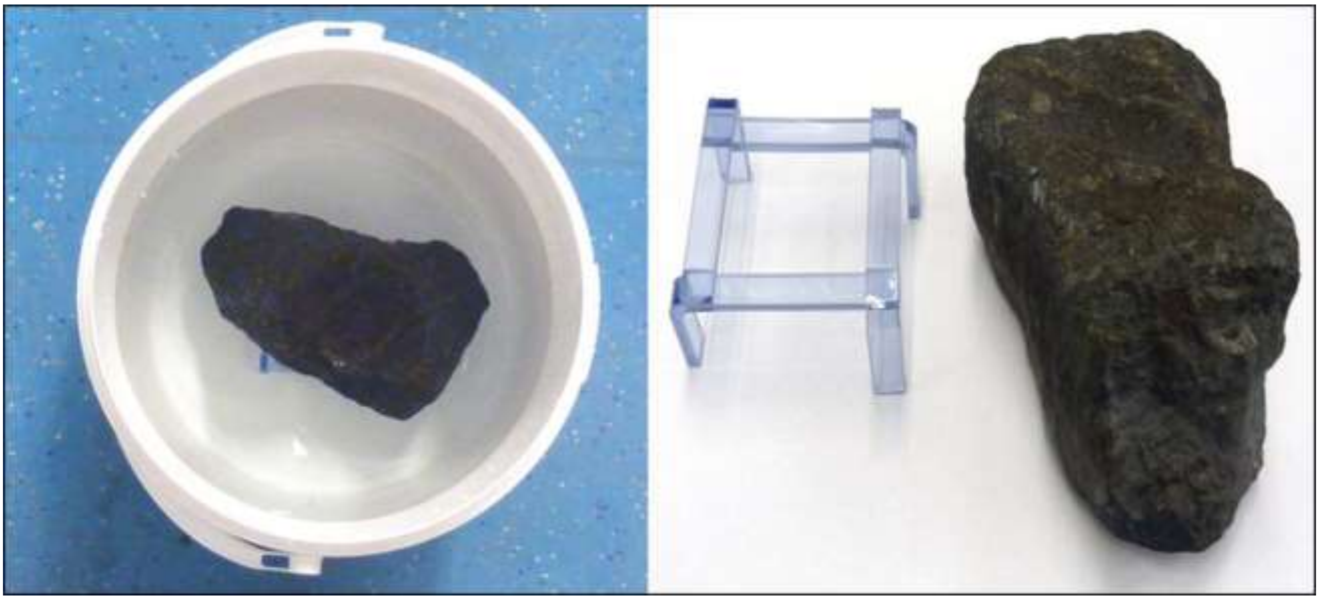

Fig C. DSLT setup as performed at the BfG.

Testing procedures

The experimental setup is presented in Fig C. In this study 70 DSLTs were performed. Each of the 7 test materials was tested with 5 repetitions by two laboratories (BfG and a contract laboratory). This equals a total number of ~650 samples analyzed for 31 elements (including blanks, quality checks and certified reference materials). Since former studies had already shown pronounced charge dependencies of the metal(loid) release from CUS, two additional CUS materials

(CUS2 and CUS3) were included to increase the reliability of the dataset [1]. In derogation from the procedure described above, CUS 2 and 3 were only tested in the BfG laboratory and CUS3 only in four repetitions (due to a limited material availability). The eluate samples, including those from the DSLTs carried out by the contract laboratory, were analyzed by the BfG. All results presented are based on these data. In addition, analytical data of eluates were delivered by the contract laboratory, which are discussed in the results and discussion section.

Chemicals, analyzes and materials BfG. Ultrapure water was produced using an USF ELGA Purelab Plus system (ELGA LabWater GmbH, Germany) or Sartorius Arium pro VF (Sartorius Lab Instruments GmbH, Germany). ICP-elemental standards (1 g/L) and nitric acid (HNO_3, 65% *w/w*, for analysis) were purchased from Merck GmbH (Germany) or Bernd Kraft GmbH (Germany). The acid was sub-boiled using a dst-1000 (Savillex Corporation, USA). Prior to use, all test vessels were rinsed at least for 24 hours with nitric acid (HNO_3, 1.3%). For filtration cellulose acetate syringe filter units with a 0.45 µm cut-off (Minisart, Sartorius Lab Instruments GmbH, Germany) with disposable 10 mL syringes (B.Braun AG, Germany) were used. All samples were acidified to 1.3% HNO_3 after filtration. The Al, B, Ba, Ca, Mg, Mn, Na, P, S, and Si concentrations in the eluates were determined by means of inductively coupled plasma-atomic emission spectroscopy (ICP-AES, Optima 8300, Perkin Elmer Inc., USA) and for the other elements by ICP-mass spectrometry (ICP-MS, Agilent 7700 series, Agilent Inc., Japan). The following certified reference materials were analyzed each measuring day and the deviation from the certified values was always < 10% for MS analyzes and for Al and Ba analyses from AES < 15%: SPS-SW1 and 2 (Spectrapure Standards, Spectrapure Inc., Norway); SLRS 5 (National Research Council Canada, Canada); TM 27.3 (Environment Canada, Canada); 1640a (National Institute of Standards and Technology, USA). The pH and the

conductivity presented in S9 Table were determined with a Multimeter (Multilab 540, WTW GmbH, Germany).

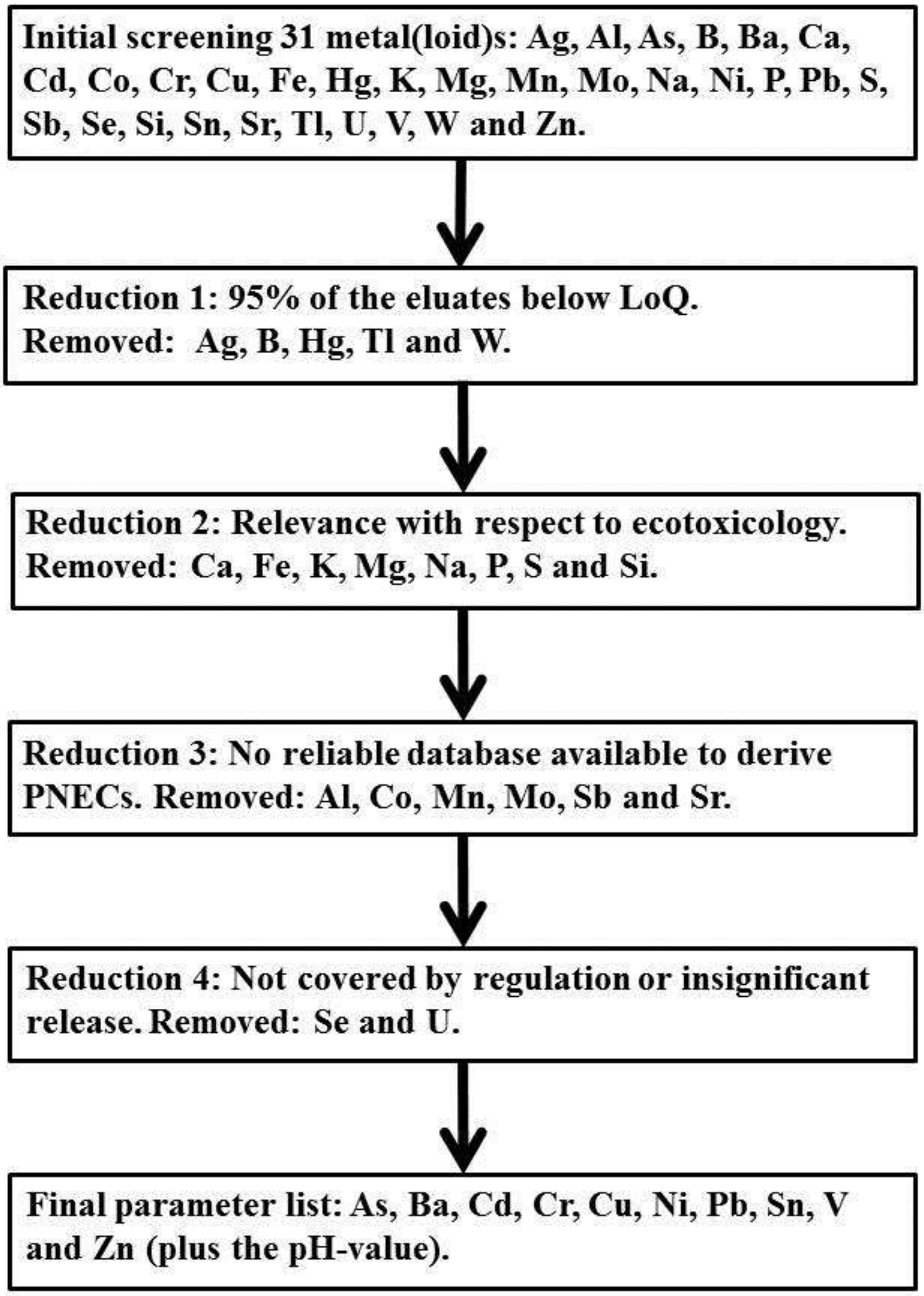

Fig D. Stepwise reduction of the parameter list as detailed in the text.

Table B. DSLT results Karbon Quartzite (LAB 1 DSLT contract laboratory, LAB 2 DSLT BfG).

Stone	Fraction	LAB 1 Al µg/l	min r mg/m²	max r mg/m²	LAB 2 Al µg/l	min r mg/m²	max r mg/m²
1	1	<40.8	0.0	3.26	<40.8	0.0	3.26
1	2	<40.8	0.0	3.26	<40.8	0.0	3.26
1	3	<40.8	0.0	3.26	<40.8	0.0	3.26
1	4	<40.8	0.0	3.26	<40.8	0.0	3.26
1	5	<40.8	0.0	3.26	<40.8	0.0	3.26
1	6	<40.8	0.0	3.26	<40.8	0.0	3.26
1	7	<40.8	0.0	3.26	<40.8	0.0	3.26
1	8	<40.8	0.0	3.26	<40.8	0.0	3.26
2	1	<40.8	0.0	3.26	<40.8	0.0	3.26
2	2	<40.8	0.0	3.26	<40.8	0.0	3.26
2	3	<40.8	0.0	3.26	<40.8	0.0	3.26
2	4	<40.8	0.0	3.26	<40.8	0.0	3.26
2	5	<40.8	0.0	3.26	<40.8	0.0	3.26
2	6	<40.8	0.0	3.26	<40.8	0.0	3.26
2	7	<40.8	0.0	3.26	<40.8	0.0	3.26
2	8	<40.8	0.0	3.26	<40.8	0.0	3.26
3	1	<40.8	0.0	3.26	<40.8	0.0	3.26
3	2	<40.8	0.0	3.26	<40.8	0.0	3.26
3	3	<40.8	0.0	3.26	<40.8	0.0	3.26
3	4	<40.8	0.0	3.26	<40.8	0.0	3.26
3	5	<40.8	0.0	3.26	<40.8	0.0	3.26
3	6	<40.8	0.0	3.26	<40.8	0.0	3.26
3	7	<40.8	0.0	3.26	<40.8	0.0	3.26
3	8	<40.8	0.0	3.26	<40.8	0.0	3.26
4	1	<40.8	0.0	3.26	<40.8	0.0	3.26
4	2	<40.8	0.0	3.26	<40.8	0.0	3.26
4	3	<40.8	0.0	3.26	<40.8	0.0	3.26
4	4	<40.8	0.0	3.26	<40.8	0.0	3.26
4	5	<40.8	0.0	3.26	<40.8	0.0	3.26
4	6	<40.8	0.0	3.26	<40.8	0.0	3.26
4	7	<40.8	0.0	3.26	<40.8	0.0	3.26
4	8	<40.8	0.0	3.26	<40.8	0.0	3.26
5	1	<40.8	0.0	3.26	<40.8	0.0	3.26
5	2	<40.8	0.0	3.26	<40.8	0.0	3.26
5	3	<40.8	0.0	3.26	<40.8	0.0	3.26
5	4	<40.8	0.0	3.26	<40.8	0.0	3.26
5	5	<40.8	0.0	3.26	<40.8	0.0	3.26
5	6	<40.8	0.0	3.26	<40.8	0.0	3.26
5	7	<40.8	0.0	3.26	<40.8	0.0	3.26
5	8	<40.8	0.0	3.26	<40.8	0.0	3.26
			∑ min	∑ max		∑ min	∑ max
1			0.0	26.1		0.0	26.1
2			0.0	26.1		0.0	26.1
3			0.0	26.1		0.0	26.1
4			0.0	26.1		0.0	26.1
5			0.0	26.1		0.0	26.1
Mean			0.0	26.1		0.0	26.1
SD			0.0	0.0		0.0	0.0

Table B. Continued Karbon Quartzite.

Stone	Fraction	LAB 1 As µg/l	min r mg/m²	max r mg/m²	LAB 2 As µg/l	min r mg/m²	max r mg/m²
1	1	0.09	0.01	0.01	<0.04	0.00	0.00
1	2	0.06	0.00	0.00	<0.04	0.00	0.00
1	3	0.06	0.00	0.00	<0.04	0.00	0.00
1	4	0.04	0.00	0.00	<0.04	0.00	0.00
1	5	0.09	0.01	0.01	0.05	0.00	0.00
1	6	0.13	0.01	0.01	<0.04	0.00	0.00
1	7	0.12	0.01	0.01	0.06	0.00	0.00
1	8	0.16	0.01	0.01	0.07	0.01	0.01
2	1	0.20	0.02	0.02	<0.04	0.00	0.00
2	2	0.24	0.02	0.02	<0.04	0.00	0.00
2	3	0.19	0.02	0.02	<0.04	0.00	0.00
2	4	0.13	0.01	0.01	<0.04	0.00	0.00
2	5	0.25	0.02	0.02	<0.04	0.00	0.00
2	6	0.32	0.03	0.03	<0.04	0.00	0.00
2	7	0.42	0.03	0.03	<0.04	0.00	0.00
2	8	0.80	0.06	0.06	<0.04	0.00	0.00
3	1	0.06	0.00	0.00	0.06	0.00	0.00
3	2	0.06	0.00	0.00	0.07	0.01	0.01
3	3	0.03	0.00	0.00	0.06	0.00	0.00
3	4	0.03	0.00	0.00	0.05	0.00	0.00
3	5	0.07	0.01	0.01	0.09	0.01	0.01
3	6	0.07	0.01	0.01	0.08	0.01	0.01
3	7	0.05	0.00	0.00	0.12	0.01	0.01
3	8	0.12	0.01	0.01	0.13	0.01	0.01
4	1	0.06	0.01	0.01	<0.04	0.00	0.00
4	2	0.06	0.00	0.00	<0.04	0.00	0.00
4	3	0.05	0.00	0.00	<0.04	0.00	0.00
4	4	0.10	0.01	0.01	<0.04	0.00	0.00
4	5	0.13	0.01	0.01	<0.04	0.00	0.00
4	6	0.13	0.01	0.01	<0.04	0.00	0.00
4	7	0.13	0.01	0.01	<0.04	0.00	0.00
4	8	0.21	0.02	0.02	<0.04	0.00	0.00
5	1	0.05	0.00	0.00	<0.04	0.00	0.00
5	2	0.06	0.00	0.00	<0.04	0.00	0.00
5	3	<0.03	0.00	0.00	<0.04	0.00	0.00
5	4	0.05	0.00	0.00	<0.04	0.00	0.00
5	5	0.05	0.00	0.00	<0.04	0.00	0.00
5	6	0.06	0.01	0.01	<0.04	0.00	0.00
5	7	0.06	0.00	0.00	0.06	0.01	0.01
5	8	0.13	0.01	0.01	0.07	0.01	0.01
			∑ min	∑ max		∑ min	∑ max
1			0.06	0.06		0.01	0.03
2			0.20	0.20		0.00	0.03
3			0.04	0.04		0.05	0.05
4			0.07	0.07		0.00	0.03
5			0.04	0.04		0.01	0.03
Mean			0.08	0.08		0.02	0.03
SD			0.07	0.07		0.02	0.01

Table B. Continued Karbon Quartzite.

Stone	Fraction	LAB 1 Ba µg/l	min r mg/m^2	max r mg/m^2	LAB 2 Ba µg/l	min r mg/m^2	max r mg/m^2
1	1	0.8	0.1	0.1	0.3	0.02	0.02
1	2	349.1	27.9	27.9	0.4	0.04	0.04
1	3	384.5	30.8	30.8	0.4	0.03	0.03
1	4	413.1	33.1	33.1	0.4	0.03	0.03
1	5	368.0	29.4	29.4	0.5	0.04	0.04
1	6	386.7	30.9	30.9	0.5	0.04	0.04
1	7	474.5	38.0	38.0	0.4	0.03	0.03
1	8	348.3	27.9	27.9	0.4	0.03	0.03
2	1	1.2	0.1	0.1	0.3	0.03	0.03
2	2	406.7	32.5	32.5	0.3	0.03	0.03
2	3	411.4	32.9	32.9	0.4	0.03	0.03
2	4	26.1	2.1	2.1	0.4	0.03	0.03
2	5	393.4	31.5	31.5	0.5	0.04	0.04
2	6	338.3	27.1	27.1	0.4	0.03	0.03
2	7	434.3	34.7	34.7	0.4	0.03	0.03
2	8	365.3	29.2	29.2	0.4	0.03	0.03
3	1	4.0	0.3	0.3	3.5	0.28	0.28
3	2	387.5	31.0	31.0	5.0	0.40	0.40
3	3	40.0	3.2	3.2	2.7	0.22	0.22
3	4	41.2	3.3	3.3	4.2	0.34	0.34
3	5	496.5	39.7	39.7	4.4	0.35	0.35
3	6	470.3	37.6	37.6	6.0	0.48	0.48
3	7	484.9	38.8	38.8	6.1	0.49	0.49
3	8	393.1	31.4	31.4	6.4	0.51	0.51
4	1	2.6	0.2	0.2	0.5	0.04	0.04
4	2	484.9	38.8	38.8	0.7	0.05	0.05
4	3	384.0	30.7	30.7	0.7	0.06	0.06
4	4	440.0	35.2	35.2	0.9	0.08	0.08
4	5	400.6	32.0	32.0	1.0	0.08	0.08
4	6	437.8	35.0	35.0	0.6	0.05	0.05
4	7	445.3	35.6	35.6	0.6	0.05	0.05
4	8	412.6	33.0	33.0	0.4	0.03	0.03
5	1	0.9	0.1	0.1	0.5	0.04	0.04
5	2	408.1	32.6	32.6	0.4	0.03	0.03
5	3	25.9	2.1	2.1	0.5	0.04	0.04
5	4	397.6	31.8	31.8	1.2	0.09	0.09
5	5	448.0	35.8	35.8	0.5	0.04	0.04
5	6	444.3	35.5	35.5	0.7	0.06	0.06
5	7	491.4	39.3	39.3	1.3	0.10	0.10
5	8	458.3	36.7	36.7	1.0	0.08	0.08
			∑ min	∑ max		∑ min	∑ max
1			218.0	218.0		0.26	0.26
2			190.1	190.1		0.26	0.26
3			185.4	185.4		3.06	3.06
4			240.6	240.6		0.44	0.44
5			214.0	214.0		0.49	0.49
Mean			209.6	209.6		0.90	0.90
SD			22.5	22.5		1.21	1.21

Table B. Continued Karbon Quartzite.

Stone	Fraction	LAB 1 Cd µg/l	min r mg/m²	max r mg/m²	LAB 2 Cd µg/l	min r mg/m²	max r mg/m²
1	1	0.07	0.005	0.005	<0.04	0.000	0.003
1	2	0.03	0.002	0.002	<0.04	0.000	0.003
1	3	0.03	0.003	0.003	<0.04	0.000	0.003
1	4	0.03	0.002	0.002	<0.04	0.000	0.003
1	5	0.03	0.003	0.003	<0.04	0.000	0.003
1	6	0.04	0.003	0.003	<0.04	0.000	0.003
1	7	0.04	0.003	0.003	<0.04	0.000	0.003
1	8	0.04	0.003	0.003	<0.04	0.000	0.003
2	1	0.01	0.001	0.001	<0.04	0.000	0.003
2	2	0.03	0.002	0.002	<0.04	0.000	0.003
2	3	0.02	0.002	0.002	<0.04	0.000	0.003
2	4	<0.01	0.000	0.001	<0.04	0.000	0.003
2	5	0.03	0.002	0.002	<0.04	0.000	0.003
2	6	0.02	0.001	0.001	<0.04	0.000	0.003
2	7	0.03	0.003	0.003	<0.04	0.000	0.003
2	8	0.02	0.002	0.002	<0.04	0.000	0.003
3	1	0.07	0.006	0.006	<0.04	0.000	0.003
3	2	0.03	0.002	0.002	<0.04	0.000	0.003
3	3	<0.01	0.000	0.001	<0.04	0.000	0.003
3	4	<0.01	0.000	0.001	<0.04	0.000	0.003
3	5	0.04	0.004	0.004	<0.04	0.000	0.003
3	6	0.03	0.002	0.002	<0.04	0.000	0.003
3	7	0.04	0.003	0.003	<0.04	0.000	0.003
3	8	0.02	0.002	0.002	<0.04	0.000	0.003
4	1	0.32	0.026	0.026	<0.04	0.000	0.003
4	2	0.02	0.002	0.002	<0.04	0.000	0.003
4	3	0.03	0.002	0.002	<0.04	0.000	0.003
4	4	0.02	0.002	0.002	<0.04	0.000	0.003
4	5	0.03	0.002	0.002	<0.04	0.000	0.003
4	6	0.03	0.002	0.002	<0.04	0.000	0.003
4	7	0.03	0.003	0.003	<0.04	0.000	0.003
4	8	0.04	0.003	0.003	<0.04	0.000	0.003
5	1	0.02	0.002	0.002	<0.04	0.000	0.003
5	2	0.02	0.002	0.002	<0.04	0.000	0.003
5	3	0.00	0.000	0.000	<0.04	0.000	0.003
5	4	0.03	0.003	0.003	<0.04	0.000	0.003
5	5	0.03	0.002	0.002	<0.04	0.000	0.003
5	6	0.02	0.002	0.002	<0.04	0.000	0.003
5	7	0.04	0.003	0.003	<0.04	0.000	0.003
5	8	0.04	0.003	0.003	<0.04	0.000	0.003
			∑ min	∑ max		∑ min	∑ max
1			0.025	0.025		0.000	0.026
2			0.013	0.014		0.000	0.026
3			0.019	0.020		0.000	0.026
4			0.042	0.042		0.000	0.026
5			0.017	0.017		0.000	0.026
Mean			0.023	0.023		0.000	0.026
SD			0.011	0.011		0.000	0.000

Table B. Continued Karbon Quartzite.

Stone	Fraction	LAB 1 Co µg/l	min r mg/m²	max r mg/m²	LAB 2 Co µg/l	min r mg/m²	max r mg/m²
1	1	0.23	0.018	0.018	<0.04	0.000	0.003
1	2	0.07	0.006	0.006	<0.04	0.000	0.003
1	3	0.08	0.006	0.006	<0.04	0.000	0.003
1	4	0.08	0.006	0.006	<0.04	0.000	0.003
1	5	0.10	0.008	0.008	<0.04	0.000	0.003
1	6	0.13	0.010	0.010	<0.04	0.000	0.003
1	7	0.45	0.036	0.036	<0.04	0.000	0.003
1	8	0.84	0.067	0.067	<0.04	0.000	0.003
2	1	0.09	0.007	0.007	<0.04	0.000	0.003
2	2	0.08	0.006	0.006	<0.04	0.000	0.003
2	3	0.07	0.006	0.006	<0.04	0.000	0.003
2	4	0.04	0.003	0.003	<0.04	0.000	0.003
2	5	0.09	0.007	0.007	<0.04	0.000	0.003
2	6	0.08	0.007	0.007	0.07	0.000	0.005
2	7	0.09	0.007	0.007	0.21	0.000	0.016
2	8	0.15	0.012	0.012	0.24	0.000	0.019
3	1	0.06	0.004	0.004	0.05	0.000	0.004
3	2	0.09	0.007	0.007	<0.04	0.000	0.003
3	3	0.03	0.002	0.002	<0.04	0.000	0.003
3	4	0.03	0.002	0.002	<0.04	0.000	0.003
3	5	0.12	0.009	0.009	<0.04	0.000	0.003
3	6	0.11	0.009	0.009	0.04	0.000	0.003
3	7	0.09	0.007	0.007	0.14	0.000	0.011
3	8	0.12	0.010	0.010	0.20	0.000	0.016
4	1	0.22	0.018	0.018	<0.04	0.000	0.003
4	2	0.10	0.008	0.008	<0.04	0.000	0.003
4	3	0.07	0.006	0.006	<0.04	0.000	0.003
4	4	0.10	0.008	0.008	<0.04	0.000	0.003
4	5	0.09	0.007	0.007	<0.04	0.000	0.003
4	6	0.12	0.010	0.010	<0.04	0.000	0.003
4	7	0.30	0.024	0.024	<0.04	0.000	0.003
4	8	0.60	0.048	0.048	<0.04	0.000	0.003
5	1	0.15	0.012	0.012	<0.04	0.000	0.003
5	2	0.08	0.006	0.006	<0.04	0.000	0.003
5	3	0.05	0.004	0.004	<0.04	0.000	0.003
5	4	0.11	0.009	0.009	<0.04	0.000	0.003
5	5	0.19	0.015	0.015	<0.04	0.000	0.003
5	6	0.30	0.024	0.024	<0.04	0.000	0.003
5	7	0.79	0.063	0.063	<0.04	0.000	0.003
5	8	1.23	0.098	0.098	<0.04	0.000	0.003
			∑ min	∑ max		∑ min	∑ max
1			0.159	0.159		0.000	0.026
2			0.057	0.057		0.000	0.057
3			0.052	0.052		0.000	0.046
4			0.129	0.129		0.000	0.026
5			0.232	0.232		0.000	0.026
Mean			0.126	0.126		0.000	0.036
SD			0.075	0.075		0.000	0.015

Table B. Continued Karbon Quartzite.

Stone	Fraction	LAB 1 Cr µg/l	min r mg/m²	max r mg/m²	LAB 2 Cr µg/l	min r mg/m²	max r mg/m²
1	1	0.09	0.007	0.007	<0.05	0.000	0.004
1	2	0.09	0.007	0.007	1.87	0.149	0.149
1	3	0.09	0.007	0.007	<0.05	0.000	0.004
1	4	0.09	0.007	0.007	<0.05	0.000	0.004
1	5	0.07	0.006	0.006	<0.05	0.000	0.004
1	6	0.11	0.009	0.009	<0.05	0.000	0.004
1	7	0.10	0.008	0.008	<0.05	0.000	0.004
1	8	<0.06	0.000	0.005	<0.05	0.000	0.004
2	1	0.08	0.006	0.006	<0.05	0.000	0.004
2	2	0.10	0.008	0.008	0.13	0.010	0.010
2	3	0.06	0.005	0.005	<0.05	0.000	0.004
2	4	<0.06	0.000	0.005	<0.05	0.000	0.004
2	5	0.09	0.007	0.007	<0.05	0.000	0.004
2	6	0.08	0.006	0.006	<0.05	0.000	0.004
2	7	0.09	0.007	0.007	<0.05	0.000	0.004
2	8	<0.06	0.000	0.005	<0.05	0.000	0.004
3	1	0.11	0.009	0.009	<0.05	0.000	0.004
3	2	0.09	0.007	0.007	0.08	0.007	0.007
3	3	0.07	0.006	0.006	<0.05	0.000	0.004
3	4	0.08	0.006	0.006	<0.05	0.000	0.004
3	5	0.17	0.014	0.014	<0.05	0.000	0.004
3	6	0.12	0.010	0.010	<0.05	0.000	0.004
3	7	0.07	0.005	0.005	<0.05	0.000	0.004
3	8	<0.06	0.000	0.005	<0.05	0.000	0.004
4	1	0.14	0.011	0.011	<0.05	0.000	0.004
4	2	0.07	0.006	0.006	0.09	0.008	0.008
4	3	0.07	0.005	0.005	<0.05	0.000	0.004
4	4	0.10	0.008	0.008	<0.05	0.000	0.004
4	5	0.08	0.006	0.006	<0.05	0.000	0.004
4	6	0.10	0.008	0.008	<0.05	0.000	0.004
4	7	0.15	0.012	0.012	<0.05	0.000	0.004
4	8	0.05	0.004	0.004	<0.05	0.000	0.004
5	1	0.09	0.007	0.007	<0.05	0.000	0.004
5	2	0.07	0.006	0.006	0.11	0.009	0.009
5	3	<0.06	0.000	0.005	<0.05	0.000	0.004
5	4	0.09	0.007	0.007	<0.05	0.000	0.004
5	5	0.09	0.007	0.007	<0.05	0.000	0.004
5	6	0.07	0.006	0.006	<0.05	0.000	0.004
5	7	0.07	0.006	0.006	<0.05	0.000	0.004
5	8	0.06	0.005	0.005	<0.05	0.000	0.004
			∑ min	∑ max		∑ min	∑ max
1			0.051	0.056		0.149	0.177
2			0.040	0.049		0.010	0.038
3			0.056	0.061		0.007	0.035
4			0.061	0.061		0.008	0.036
5			0.044	0.049		0.009	0.037
Mean			0.050	0.055		0.037	0.065
SD			0.009	0.006		0.063	0.063

Table B. Continued Karbon Quartzite.

Stone	Fraction	LAB 1 Cu µg/l	min r mg/m²	max r mg/m²	LAB 2 Cu µg/l	min r mg/m²	max r mg/m²
1	1	2.097	0.168	0.168	<0.06	0.000	0.005
1	2	2.833	0.227	0.227	<0.06	0.000	0.005
1	3	2.052	0.164	0.164	<0.06	0.000	0.005
1	4	2.235	0.179	0.179	<0.06	0.000	0.005
1	5	2.396	0.192	0.192	<0.06	0.000	0.005
1	6	2.486	0.199	0.199	<0.06	0.000	0.005
1	7	4.372	0.350	0.350	<0.06	0.000	0.005
1	8	1.578	0.126	0.126	<0.06	0.000	0.005
2	1	1.116	0.089	0.089	<0.06	0.000	0.005
2	2	2.550	0.204	0.204	<0.06	0.000	0.005
2	3	1.681	0.134	0.134	<0.06	0.000	0.005
2	4	1.007	0.081	0.081	<0.06	0.000	0.005
2	5	2.840	0.227	0.227	<0.06	0.000	0.005
2	6	1.659	0.133	0.133	<0.06	0.000	0.005
2	7	2.694	0.216	0.216	<0.06	0.000	0.005
2	8	1.276	0.102	0.102	<0.06	0.000	0.005
3	1	4.513	0.361	0.361	<0.06	0.000	0.005
3	2	3.236	0.259	0.259	<0.06	0.000	0.005
3	3	1.958	0.157	0.157	<0.06	0.000	0.005
3	4	1.375	0.110	0.110	<0.06	0.000	0.005
3	5	3.459	0.277	0.277	<0.06	0.000	0.005
3	6	2.314	0.185	0.185	<0.06	0.000	0.005
3	7	1.804	0.144	0.144	<0.06	0.000	0.005
3	8	1.803	0.144	0.144	<0.06	0.000	0.005
4	1	7.607	0.609	0.609	<0.06	0.000	0.005
4	2	3.973	0.318	0.318	<0.06	0.000	0.005
4	3	3.043	0.243	0.243	<0.06	0.000	0.005
4	4	3.016	0.241	0.241	<0.06	0.000	0.005
4	5	3.632	0.291	0.291	<0.06	0.000	0.005
4	6	3.491	0.279	0.279	<0.06	0.000	0.005
4	7	3.114	0.249	0.249	<0.06	0.000	0.005
4	8	3.330	0.266	0.266	<0.06	0.000	0.005
5	1	1.767	0.141	0.141	<0.06	0.000	0.005
5	2	2.220	0.178	0.178	<0.06	0.000	0.005
5	3	0.819	0.065	0.065	<0.06	0.000	0.005
5	4	2.106	0.168	0.168	<0.06	0.000	0.005
5	5	2.731	0.218	0.218	<0.06	0.000	0.005
5	6	2.313	0.185	0.185	<0.06	0.000	0.005
5	7	1.891	0.151	0.151	<0.06	0.000	0.005
5	8	1.921	0.154	0.154	<0.06	0.000	0.005
			∑ min	∑ max		∑ min	∑ max
1			1.604	1.604		0.000	0.038
2			1.186	1.186		0.000	0.038
3			1.637	1.637		0.000	0.038
4			2.496	2.496		0.000	0.038
5			1.261	1.261		0.000	0.038
Mean			1.637	1.637		0.000	0.038
SD			0.521	0.521		0.000	0.000

Table B. Continued Karbon Quartzite.

Stone	Fraction	LAB 1 Mn µg/l	min r mg/m^2	max r mg/m^2	LAB 2 Mn µg/l	min r mg/m^2	max r mg/m^2
1	1	3.86	0.31	0.31	0.43	0.03	0.03
1	2	0.31	0.02	0.02	1.16	0.09	0.09
1	3	0.34	0.03	0.03	0.33	0.03	0.03
1	4	0.35	0.03	0.03	0.55	0.04	0.04
1	5	0.36	0.03	0.03	0.39	0.03	0.03
1	6	0.53	0.04	0.04	0.46	0.04	0.04
1	7	4.16	0.33	0.33	0.56	0.04	0.04
1	8	8.27	0.66	0.66	0.56	0.04	0.04
2	1	5.96	0.48	0.48	3.79	0.30	0.30
2	2	0.40	0.03	0.03	5.96	0.48	0.48
2	3	0.31	0.02	0.02	8.32	0.66	0.66
2	4	0.83	0.07	0.07	12.03	0.96	0.96
2	5	0.51	0.04	0.04	26.41	2.11	2.11
2	6	0.89	0.07	0.07	31.41	2.51	2.51
2	7	1.11	0.09	0.09	61.32	4.90	4.90
2	8	1.81	0.14	0.14	59.52	4.76	4.76
3	1	4.97	0.40	0.40	1.78	0.14	0.14
3	2	0.78	0.06	0.06	0.57	0.05	0.05
3	3	2.45	0.20	0.20	0.25	0.02	0.02
3	4	2.76	0.22	0.22	0.28	0.02	0.02
3	5	2.34	0.19	0.19	0.63	0.05	0.05
3	6	3.03	0.24	0.24	1.42	0.11	0.11
3	7	3.45	0.28	0.28	4.06	0.32	0.32
3	8	3.78	0.30	0.30	5.24	0.42	0.42
4	1	6.76	0.54	0.54	0.36	0.03	0.03
4	2	0.56	0.04	0.04	2.31	0.18	0.18
4	3	0.31	0.02	0.02	1.05	0.08	0.08
4	4	0.48	0.04	0.04	1.28	0.10	0.10
4	5	0.38	0.03	0.03	1.13	0.09	0.09
4	6	0.53	0.04	0.04	1.28	0.10	0.10
4	7	2.32	0.19	0.19	1.36	0.11	0.11
4	8	4.52	0.36	0.36	1.33	0.11	0.11
5	1	7.27	0.58	0.58	0.61	0.05	0.05
5	2	0.57	0.05	0.05	0.47	0.04	0.04
5	3	2.19	0.17	0.17	0.22	0.02	0.02
5	4	1.07	0.09	0.09	0.22	0.02	0.02
5	5	4.61	0.37	0.37	0.39	0.03	0.03
5	6	8.60	0.69	0.69	0.30	0.02	0.02
5	7	22.52	1.80	1.80	0.75	0.06	0.06
5	8	32.70	2.62	2.62	0.93	0.07	0.07
			∑ min	∑ max		∑ min	∑ max
1			1.45	1.45		0.36	0.36
2			0.95	0.95		16.69	16.69
3			1.88	1.88		1.14	1.14
4			1.27	1.27		0.81	0.81
5			6.36	6.36		0.31	0.31
Mean			2.38	2.38		3.86	3.86
SD			2.25	2.25		7.18	7.18

Table B. Continued Karbon Quartzite.

Stone	Fraction	LAB 1 Mo µg/l	min r mg/m²	max r mg/m²	LAB 2 Mo µg/l	min r mg/m²	max r mg/m²
1	1	0.080	0.006	0.006	<0.07	0.000	0.006
1	2	0.061	0.005	0.005	0.26	0.021	0.021
1	3	0.057	0.005	0.005	<0.07	0.000	0.006
1	4	<0.06	0.000	0.005	<0.07	0.000	0.006
1	5	0.059	0.005	0.005	<0.07	0.000	0.006
1	6	0.069	0.006	0.006	<0.07	0.000	0.006
1	7	0.060	0.005	0.005	<0.07	0.000	0.006
1	8	<0.06	0.000	0.005	<0.07	0.000	0.006
2	1	0.197	0.016	0.016	<0.07	0.000	0.006
2	2	0.185	0.015	0.015	0.07	0.005	0.005
2	3	0.104	0.008	0.008	<0.07	0.000	0.006
2	4	<0.06	0.000	0.005	<0.07	0.000	0.006
2	5	<0.06	0.000	0.005	<0.07	0.000	0.006
2	6	<0.06	0.000	0.005	<0.07	0.000	0.006
2	7	0.062	0.005	0.005	<0.07	0.000	0.006
2	8	0.089	0.007	0.007	<0.07	0.000	0.006
3	1	<0.06	0.000	0.005	<0.07	0.000	0.006
3	2	0.067	0.005	0.005	<0.07	0.000	0.006
3	3	<0.06	0.000	0.005	<0.07	0.000	0.006
3	4	<0.06	0.000	0.005	<0.07	0.000	0.006
3	5	0.079	0.006	0.006	<0.07	0.000	0.006
3	6	0.067	0.005	0.005	<0.07	0.000	0.006
3	7	<0.06	0.000	0.005	<0.07	0.000	0.006
3	8	0.077	0.006	0.006	<0.07	0.000	0.006
4	1	<0.06	0.000	0.005	<0.07	0.000	0.006
4	2	<0.06	0.000	0.005	<0.07	0.000	0.006
4	3	<0.06	0.000	0.005	<0.07	0.000	0.006
4	4	<0.06	0.000	0.005	<0.07	0.000	0.006
4	5	<0.06	0.000	0.005	<0.07	0.000	0.006
4	6	<0.06	0.000	0.005	<0.07	0.000	0.006
4	7	<0.06	0.000	0.005	<0.07	0.000	0.006
4	8	<0.06	0.000	0.005	<0.07	0.000	0.006
5	1	0.077	0.006	0.006	<0.07	0.000	0.006
5	2	<0.06	0.000	0.005	<0.07	0.000	0.006
5	3	<0.06	0.000	0.005	<0.07	0.000	0.006
5	4	<0.06	0.000	0.005	<0.07	0.000	0.006
5	5	<0.06	0.000	0.005	<0.07	0.000	0.006
5	6	<0.06	0.000	0.005	<0.07	0.000	0.006
5	7	<0.06	0.000	0.005	<0.07	0.000	0.006
5	8	<0.06	0.000	0.005	<0.07	0.000	0.006
			∑ min	∑ max		∑ min	∑ max
1			0.031	0.040		0.021	0.060
2			0.051	0.065		0.005	0.045
3			0.023	0.042		0.000	0.045
4			0.000	0.038		0.000	0.045
5			0.006	0.040		0.000	0.045
Mean			0.022	0.045		0.005	0.048
SD			0.020	0.011		0.009	0.007

Table B. Continued Karbon Quartzite.

Stone	Fraction	LAB 1 Ni µg/l	min r mg/m^2	max r mg/m^2	LAB 2 Ni µg/l	min r mg/m^2	max r mg/m^2
1	1	0.50	0.040	0.040	<0.05	0.000	0.004
1	2	0.36	0.029	0.029	0.53	0.042	0.042
1	3	0.24	0.020	0.020	<0.05	0.000	0.004
1	4	0.26	0.021	0.021	<0.05	0.000	0.004
1	5	0.28	0.022	0.022	<0.05	0.000	0.004
1	6	0.38	0.030	0.030	<0.05	0.000	0.004
1	7	1.13	0.091	0.091	<0.05	0.000	0.004
1	8	1.40	0.112	0.112	<0.05	0.000	0.004
2	1	0.26	0.021	0.021	0.18	0.014	0.014
2	2	0.37	0.029	0.029	0.11	0.009	0.009
2	3	0.23	0.019	0.019	0.09	0.007	0.007
2	4	0.11	0.009	0.009	0.08	0.007	0.007
2	5	0.33	0.027	0.027	0.16	0.013	0.013
2	6	0.35	0.028	0.028	0.22	0.018	0.018
2	7	0.41	0.033	0.033	0.51	0.000	0.040
2	8	0.41	0.033	0.033	0.54	0.000	0.043
3	1	1.74	0.139	0.139	0.10	0.008	0.008
3	2	0.74	0.059	0.059	<0.05	0.000	0.004
3	3	0.29	0.023	0.023	<0.05	0.000	0.004
3	4	0.21	0.017	0.017	<0.05	0.000	0.004
3	5	0.41	0.033	0.033	<0.05	0.000	0.004
3	6	0.46	0.037	0.037	0.08	0.007	0.007
3	7	0.30	0.024	0.024	0.24	0.020	0.020
3	8	0.75	0.060	0.060	0.31	0.000	0.025
4	1	2.19	0.175	0.175	<0.05	0.000	0.004
4	2	0.63	0.051	0.051	<0.05	0.000	0.004
4	3	0.26	0.021	0.021	<0.05	0.000	0.004
4	4	0.37	0.030	0.030	<0.05	0.000	0.004
4	5	0.32	0.026	0.026	<0.05	0.000	0.004
4	6	0.41	0.033	0.033	<0.05	0.000	0.004
4	7	0.70	0.056	0.056	<0.05	0.000	0.004
4	8	1.33	0.107	0.107	<0.05	0.000	0.004
5	1	0.82	0.066	0.066	<0.05	0.000	0.004
5	2	0.47	0.038	0.038	<0.05	0.000	0.004
5	3	0.24	0.020	0.020	<0.05	0.000	0.004
5	4	0.37	0.029	0.029	<0.05	0.000	0.004
5	5	0.61	0.049	0.049	<0.05	0.000	0.004
5	6	0.95	0.076	0.076	<0.05	0.000	0.004
5	7	1.74	0.139	0.139	<0.05	0.000	0.004
5	8	2.33	0.186	0.186	<0.05	0.000	0.004
			∑ min	∑ max		∑ min	∑ max
1			0.365	0.365		0.042	0.070
2			0.198	0.198		0.068	0.152
3			0.391	0.391		0.034	0.075
4			0.499	0.499		0.000	0.032
5			0.603	0.603		0.000	0.032
Mean			0.411	0.411		0.029	0.072
SD			0.152	0.152		0.029	0.049

Table B. Continued Karbon Quartzite.

Stone	Fraction	LAB 1 Pb µg/l	min r mg/m^2	max r mg/m^2	LAB 2 Pb µg/l	min r mg/m^2	max r mg/m^2
1	1	0.30	0.024	0.024	0.01	0.001	0.001
1	2	0.08	0.007	0.007	<0.01	0.000	0.001
1	3	0.12	0.009	0.009	<0.01	0.000	0.001
1	4	0.15	0.012	0.012	<0.01	0.000	0.001
1	5	0.15	0.012	0.012	<0.01	0.000	0.001
1	6	0.21	0.017	0.017	<0.01	0.000	0.001
1	7	0.16	0.013	0.013	<0.01	0.000	0.001
1	8	0.02	0.001	0.001	<0.01	0.000	0.001
2	1	0.12	0.010	0.010	<0.01	0.000	0.001
2	2	0.06	0.005	0.005	<0.01	0.000	0.001
2	3	0.07	0.005	0.005	<0.01	0.000	0.001
2	4	0.13	0.010	0.010	<0.01	0.000	0.001
2	5	0.09	0.007	0.007	<0.01	0.000	0.001
2	6	0.08	0.007	0.007	<0.01	0.000	0.001
2	7	0.09	0.007	0.007	<0.01	0.000	0.001
2	8	0.04	0.003	0.003	<0.01	0.000	0.001
3	1	0.24	0.019	0.019	<0.01	0.000	0.001
3	2	0.08	0.007	0.007	<0.01	0.000	0.001
3	3	0.17	0.013	0.013	<0.01	0.000	0.001
3	4	0.29	0.023	0.023	<0.01	0.000	0.001
3	5	0.43	0.034	0.034	<0.01	0.000	0.001
3	6	0.26	0.021	0.021	<0.01	0.000	0.001
3	7	0.12	0.009	0.009	<0.01	0.000	0.001
3	8	< 0.03	0.000	0.002	<0.01	0.000	0.001
4	1	0.51	0.041	0.041	<0.01	0.000	0.001
4	2	0.07	0.006	0.006	<0.01	0.000	0.001
4	3	0.12	0.010	0.010	<0.01	0.000	0.001
4	4	0.12	0.009	0.009	<0.01	0.000	0.001
4	5	0.10	0.008	0.008	<0.01	0.000	0.001
4	6	0.11	0.009	0.009	<0.01	0.000	0.001
4	7	0.05	0.004	0.004	<0.01	0.000	0.001
4	8	< 0.03	0.000	0.002	<0.01	0.000	0.001
5	1	0.12	0.009	0.009	<0.01	0.000	0.001
5	2	0.07	0.006	0.006	<0.01	0.000	0.001
5	3	0.08	0.006	0.006	<0.01	0.000	0.001
5	4	0.09	0.007	0.007	<0.01	0.000	0.001
5	5	0.17	0.014	0.014	<0.01	0.000	0.001
5	6	0.07	0.006	0.006	<0.01	0.000	0.001
5	7	0.06	0.005	0.005	<0.01	0.000	0.001
5	8	0.05	0.004	0.004	<0.01	0.000	0.001
			∑ min	∑ max		∑ min	∑ max
1			0.095	0.095		0.001	0.006
2			0.055	0.055		0.000	0.006
3			0.127	0.129		0.000	0.006
4			0.086	0.089		0.000	0.006
5			0.056	0.056		0.000	0.006
Mean			0.084	0.085		0.000	0.006
SD			0.030	0.031		0.000	0.000

Table B. Continued Karbon Quartzite.

Stone	Fraction	**LAB 1 Sb µg/l**	**min r mg/m²**	**max r mg/m²**	**LAB 2 Sb µg/l**	**min r mg/m²**	**max r mg/m²**
1	1	0.142	0.011	0.011	<0.06	0.000	0.005
1	2	0.093	0.007	0.007	<0.06	0.000	0.005
1	3	0.093	0.007	0.007	<0.06	0.000	0.005
1	4	0.078	0.006	0.006	<0.06	0.000	0.005
1	5	0.074	0.006	0.006	<0.06	0.000	0.005
1	6	0.116	0.009	0.009	<0.06	0.000	0.005
1	7	0.152	0.012	0.012	<0.06	0.000	0.005
1	8	0.235	0.019	0.019	<0.06	0.000	0.005
2	1	<0.06	0.000	0.005	<0.06	0.000	0.005
2	2	0.093	0.007	0.007	<0.06	0.000	0.005
2	3	0.083	0.007	0.007	<0.06	0.000	0.005
2	4	<0.06	0.000	0.005	<0.06	0.000	0.005
2	5	0.078	0.006	0.006	<0.06	0.000	0.005
2	6	0.102	0.008	0.008	<0.06	0.000	0.005
2	7	0.092	0.007	0.007	<0.06	0.000	0.005
2	8	0.252	0.020	0.020	<0.06	0.000	0.005
3	1	<0.06	0.000	0.005	<0.06	0.000	0.005
3	2	0.081	0.006	0.006	<0.06	0.000	0.005
3	3	<0.06	0.000	0.005	<0.06	0.000	0.005
3	4	<0.06	0.000	0.005	<0.06	0.000	0.005
3	5	0.096	0.008	0.008	<0.06	0.000	0.005
3	6	0.100	0.008	0.008	<0.06	0.000	0.005
3	7	0.090	0.007	0.007	<0.06	0.000	0.005
3	8	0.231	0.018	0.018	<0.06	0.000	0.005
4	1	<0.06	0.000	0.005	<0.06	0.000	0.005
4	2	0.097	0.008	0.008	<0.06	0.000	0.005
4	3	0.094	0.008	0.008	<0.06	0.000	0.005
4	4	0.094	0.007	0.007	<0.06	0.000	0.005
4	5	0.081	0.006	0.006	<0.06	0.000	0.005
4	6	0.166	0.013	0.013	<0.06	0.000	0.005
4	7	0.109	0.009	0.009	<0.06	0.000	0.005
4	8	0.255	0.020	0.020	<0.06	0.000	0.005
5	1	<0.06	0.000	0.005	<0.06	0.000	0.005
5	2	0.077	0.006	0.006	<0.06	0.000	0.005
5	3	<0.06	0.000	0.005	<0.06	0.000	0.005
5	4	0.062	0.005	0.005	<0.06	0.000	0.005
5	5	0.083	0.007	0.007	<0.06	0.000	0.005
5	6	0.073	0.006	0.006	<0.06	0.000	0.005
5	7	0.090	0.007	0.007	<0.06	0.000	0.005
5	8	0.255	0.020	0.020	<0.06	0.000	0.005
			∑ min	∑ max		∑ min	∑ max
1			0.079	0.079		0.000	0.038
2			0.056	0.066		0.000	0.038
3			0.048	0.062		0.000	0.038
4			0.072	0.076		0.000	0.038
5			0.051	0.061		0.000	0.038
Mean			0.061	0.069		0.000	0.038
SD			0.013	0.008		0.000	0.000

Table B. Continued Karbon Quartzite.

Stone	Fraction	LAB 1 Se µg/l	min r mg/m²	max r mg/m²	LAB 2 Se µg/l	min r mg/m²	max r mg/m²
1	1	0.05	0.004	0.004	<0.83	0.00	0.07
1	2	<0.04	0.000	0.003	<0.83	0.00	0.07
1	3	<0.04	0.000	0.003	<0.83	0.00	0.07
1	4	<0.04	0.000	0.003	<0.83	0.00	0.07
1	5	<0.04	0.000	0.003	<0.83	0.00	0.07
1	6	0.04	0.003	0.003	<0.83	0.00	0.07
1	7	<0.04	0.000	0.003	<0.83	0.00	0.07
1	8	<0.04	0.000	0.003	<0.83	0.00	0.07
2	1	<0.04	0.000	0.003	<0.83	0.00	0.07
2	2	<0.04	0.000	0.003	<0.83	0.00	0.07
2	3	<0.04	0.000	0.003	<0.83	0.00	0.07
2	4	<0.04	0.000	0.003	<0.83	0.00	0.07
2	5	<0.04	0.000	0.003	<0.83	0.00	0.07
2	6	<0.04	0.000	0.003	<0.83	0.00	0.07
2	7	<0.04	0.000	0.003	<0.83	0.00	0.07
2	8	<0.04	0.000	0.003	<0.83	0.00	0.07
3	1	<0.04	0.000	0.003	<0.83	0.00	0.07
3	2	<0.04	0.000	0.003	<0.83	0.00	0.07
3	3	<0.04	0.000	0.003	<0.83	0.00	0.07
3	4	<0.04	0.000	0.003	<0.83	0.00	0.07
3	5	<0.04	0.000	0.003	<0.83	0.00	0.07
3	6	<0.04	0.000	0.003	<0.83	0.00	0.07
3	7	<0.04	0.000	0.003	<0.83	0.00	0.07
3	8	<0.04	0.000	0.003	<0.83	0.00	0.07
4	1	0.06	0.005	0.005	<0.83	0.00	0.07
4	2	0.05	0.004	0.004	<0.83	0.00	0.07
4	3	<0.04	0.000	0.003	<0.83	0.00	0.07
4	4	0.05	0.004	0.004	<0.83	0.00	0.07
4	5	0.13	0.011	0.011	<0.83	0.00	0.07
4	6	0.21	0.017	0.017	<0.83	0.00	0.07
4	7	0.43	0.034	0.034	<0.83	0.00	0.07
4	8	0.50	0.040	0.040	<0.83	0.00	0.07
5	1	0.12	0.009	0.009	<0.83	0.00	0.07
5	2	0.07	0.006	0.006	<0.83	0.00	0.07
5	3	0.08	0.007	0.007	<0.83	0.00	0.07
5	4	0.13	0.010	0.010	<0.83	0.00	0.07
5	5	0.29	0.023	0.023	<0.83	0.00	0.07
5	6	0.47	0.037	0.037	<0.83	0.00	0.07
5	7	0.91	0.073	0.073	<0.83	0.00	0.07
5	8	1.12	0.090	0.090	<0.83	0.00	0.07
			∑ min	∑ max		∑ min	∑ max
1			0.008	0.027		0.00	0.53
2			0.000	0.026		0.00	0.53
3			0.000	0.026		0.00	0.53
4			0.114	0.117		0.00	0.53
5			0.255	0.255		0.00	0.53
Mean			0.075	0.090		0.00	0.53
SD			0.112	0.100		0.00	0.00

Table B. Continued Karbon Quartzite.

Stone	Fraction	LAB 1 Sn µg/l	min r mg/m^2	max r mg/m^2	LAB 2 Sn µg/l	min r mg/m^2	max r mg/m^2
1	1	0.156	0.012	0.012	<0.02	0.00	0.002
1	2	0.106	0.008	0.008	<0.02	0.00	0.002
1	3	0.090	0.007	0.007	<0.02	0.00	0.002
1	4	0.182	0.015	0.015	<0.02	0.00	0.002
1	5	0.066	0.005	0.005	<0.02	0.00	0.002
1	6	0.119	0.010	0.010	<0.02	0.00	0.002
1	7	0.143	0.011	0.011	<0.02	0.00	0.002
1	8	0.031	0.002	0.002	<0.02	0.00	0.002
2	1	0.059	0.005	0.005	<0.02	0.00	0.002
2	2	0.117	0.009	0.009	<0.02	0.00	0.002
2	3	0.086	0.007	0.007	<0.02	0.00	0.002
2	4	0.226	0.018	0.018	<0.02	0.00	0.002
2	5	0.053	0.004	0.004	<0.02	0.00	0.002
2	6	0.116	0.009	0.009	<0.02	0.00	0.002
2	7	0.037	0.003	0.003	<0.02	0.00	0.002
2	8	<0.03	0.000	0.002	<0.02	0.00	0.002
3	1	0.114	0.009	0.009	<0.02	0.00	0.002
3	2	0.465	0.037	0.037	<0.02	0.00	0.002
3	3	0.236	0.019	0.019	<0.02	0.00	0.002
3	4	0.254	0.020	0.020	<0.02	0.00	0.002
3	5	0.133	0.011	0.011	<0.02	0.00	0.002
3	6	0.185	0.015	0.015	<0.02	0.00	0.002
3	7	0.064	0.005	0.005	<0.02	0.00	0.002
3	8	<0.03	0.000	0.002	<0.02	0.00	0.002
4	1	0.117	0.009	0.009	<0.02	0.00	0.002
4	2	0.139	0.011	0.011	<0.02	0.00	0.002
4	3	0.121	0.010	0.010	<0.02	0.00	0.002
4	4	0.179	0.014	0.014	<0.02	0.00	0.002
4	5	0.087	0.007	0.007	<0.02	0.00	0.002
4	6	0.149	0.012	0.012	<0.02	0.00	0.002
4	7	0.081	0.007	0.007	<0.02	0.00	0.002
4	8	0.031	0.002	0.002	<0.02	0.00	0.002
5	1	0.051	0.004	0.004	<0.02	0.00	0.002
5	2	0.149	0.012	0.012	<0.02	0.00	0.002
5	3	0.089	0.007	0.007	<0.02	0.00	0.002
5	4	0.221	0.018	0.018	<0.02	0.00	0.002
5	5	0.059	0.005	0.005	<0.02	0.00	0.002
5	6	0.118	0.009	0.009	<0.02	0.00	0.002
5	7	0.050	0.004	0.004	<0.02	0.00	0.002
5	8	0.019	0.002	0.002	<0.02	0.00	0.002
			∑ min	∑ max		∑ min	∑ max
1			0.071	0.071		0.00	0.013
2			0.056	0.058		0.00	0.013
3			0.116	0.118		0.00	0.013
4			0.072	0.072		0.00	0.013
5			0.060	0.060		0.00	0.013
Mean			0.075	0.076		0.00	0.013
SD			0.024	0.025		0.00	0.000

Table B. Continued Karbon Quartzite.

Stone	Fraction	LAB 1 Sr µg/l	min r mg/m^2	max r mg/m^2	LAB 2 Sr µg/l	min r mg/m^2	max r mg/m^2
1	1	<0.71	0.00	0.06	<0.71	0.00	0.06
1	2	6.05	0.48	0.48	<0.71	0.00	0.06
1	3	6.13	0.49	0.49	<0.71	0.00	0.06
1	4	7.02	0.56	0.56	<0.71	0.00	0.06
1	5	6.45	0.52	0.52	<0.71	0.00	0.06
1	6	6.73	0.54	0.54	<0.71	0.00	0.06
1	7	7.77	0.62	0.62	0.74	0.06	0.06
1	8	6.72	0.54	0.54	0.72	0.06	0.06
2	1	<0.71	0.00	0.06	<0.71	0.00	0.06
2	2	6.49	0.52	0.52	<0.71	0.00	0.06
2	3	6.37	0.51	0.51	<0.71	0.00	0.06
2	4	0.85	0.07	0.07	<0.71	0.00	0.06
2	5	6.92	0.55	0.55	1.03	0.08	0.08
2	6	6.51	0.52	0.52	1.18	0.09	0.09
2	7	7.66	0.61	0.61	2.09	0.17	0.17
2	8	6.37	0.51	0.51	2.11	0.17	0.17
3	1	<0.71	0.00	0.06	<0.71	0.00	0.06
3	2	6.95	0.56	0.56	<0.71	0.00	0.06
3	3	<0.71	0.00	0.06	<0.71	0.00	0.06
3	4	0.73	0.06	0.06	<0.71	0.00	0.06
3	5	8.55	0.68	0.68	<0.71	0.00	0.06
3	6	8.25	0.66	0.66	<0.71	0.00	0.06
3	7	8.66	0.69	0.69	<0.71	0.00	0.06
3	8	7.38	0.59	0.59	<0.71	0.00	0.06
4	1	1.45	0.12	0.12	<0.71	0.00	0.06
4	2	7.76	0.62	0.62	<0.71	0.00	0.06
4	3	6.67	0.53	0.53	<0.71	0.00	0.06
4	4	7.43	0.59	0.59	<0.71	0.00	0.06
4	5	7.00	0.56	0.56	<0.71	0.00	0.06
4	6	7.56	0.60	0.60	<0.71	0.00	0.06
4	7	7.42	0.59	0.59	0.83	0.07	0.07
4	8	6.75	0.54	0.54	0.89	0.07	0.07
5	1	<0.71	0.00	0.06	<0.71	0.00	0.06
5	2	6.58	0.53	0.53	<0.71	0.00	0.06
5	3	<0.71	0.00	0.06	<0.71	0.00	0.06
5	4	6.64	0.53	0.53	<0.71	0.00	0.06
5	5	7.42	0.59	0.59	<0.71	0.00	0.06
5	6	7.96	0.64	0.64	<0.71	0.00	0.06
5	7	8.36	0.67	0.67	<0.71	0.00	0.06
5	8	7.74	0.62	0.62	<0.71	0.00	0.06
			∑ min	∑ max		∑ min	∑ max
1			3.75	3.81		0.12	0.46
2			3.29	3.35		0.51	0.74
3			3.24	3.36		0.00	0.45
4			4.16	4.16		0.14	0.48
5			3.58	3.69		0.00	0.45
Mean			3.60	3.67		0.15	0.52
SD			0.37	0.34		0.21	0.13

Table B. Continued Karbon Quartzite:

Stone	Fraction	LAB 1 U µg/l	min r mg/m²	max r mg/m²	LAB 2 U µg/l	min r mg/m²	max r mg/m²
1	1	0.09	0.007	0.007	<0.01	0.000	0.001
1	2	<0.04	0.000	0.003	<0.01	0.000	0.001
1	3	<0.04	0.000	0.003	<0.01	0.000	0.001
1	4	<0.04	0.000	0.003	<0.01	0.000	0.001
1	5	<0.04	0.000	0.003	<0.01	0.000	0.001
1	6	<0.04	0.000	0.003	<0.01	0.000	0.001
1	7	0.08	0.006	0.006	<0.01	0.000	0.001
1	8	<0.04	0.000	0.003	<0.01	0.000	0.001
2	1	<0.04	0.000	0.003	<0.01	0.000	0.001
2	2	<0.04	0.000	0.003	<0.01	0.000	0.001
2	3	<0.04	0.000	0.003	<0.01	0.000	0.001
2	4	<0.04	0.000	0.003	<0.01	0.000	0.001
2	5	<0.04	0.000	0.003	<0.01	0.000	0.001
2	6	<0.04	0.000	0.003	<0.01	0.000	0.001
2	7	<0.04	0.000	0.003	<0.01	0.000	0.001
2	8	<0.04	0.000	0.003	<0.01	0.000	0.001
3	1	<0.04	0.000	0.003	<0.01	0.000	0.001
3	2	<0.04	0.000	0.003	<0.01	0.000	0.001
3	3	<0.04	0.000	0.003	<0.01	0.000	0.001
3	4	<0.04	0.000	0.003	<0.01	0.000	0.001
3	5	<0.04	0.000	0.003	<0.01	0.000	0.001
3	6	<0.04	0.000	0.003	<0.01	0.000	0.001
3	7	<0.04	0.000	0.003	<0.01	0.000	0.001
3	8	<0.04	0.000	0.003	<0.01	0.000	0.001
4	1	<0.04	0.000	0.003	<0.01	0.000	0.001
4	2	<0.04	0.000	0.003	<0.01	0.000	0.001
4	3	<0.04	0.000	0.003	<0.01	0.000	0.001
4	4	<0.04	0.000	0.003	<0.01	0.000	0.001
4	5	<0.04	0.000	0.003	<0.01	0.000	0.001
4	6	<0.04	0.000	0.003	<0.01	0.000	0.001
4	7	<0.04	0.000	0.003	<0.01	0.000	0.001
4	8	<0.04	0.000	0.003	<0.01	0.000	0.001
5	1	<0.04	0.000	0.003	<0.01	0.000	0.001
5	2	<0.04	0.000	0.003	<0.01	0.000	0.001
5	3	<0.04	0.000	0.003	<0.01	0.000	0.001
5	4	<0.04	0.000	0.003	<0.01	0.000	0.001
5	5	<0.04	0.000	0.003	<0.01	0.000	0.001
5	6	<0.04	0.000	0.003	<0.01	0.000	0.001
5	7	<0.04	0.000	0.003	<0.01	0.000	0.001
5	8	<0.04	0.000	0.003	<0.01	0.000	0.001
			∑ min	∑ max		∑ min	∑ max
1			0.014	0.033		0.000	0.006
2			0.000	0.026		0.000	0.006
3			0.000	0.026		0.000	0.006
4			0.000	0.026		0.000	0.006
5			0.000	0.026		0.000	0.006
Mean			0.003	0.027		0.000	0.006
SD			0.006	0.003		0.000	0.000

Table B. Continued Karbon Quartzite.

Stone	Fraction	LAB 1 V µg/l	min r mg/m²	max r mg/m²	LAB 2 V µg/l	min r mg/m²	max r mg/m²
1	1	0.119	0.010	0.010	<0.05	0.000	0.004
1	2	0.024	0.002	0.002	<0.05	0.000	0.004
1	3	0.033	0.003	0.003	<0.05	0.000	0.004
1	4	<0.02	0.000	0.002	<0.05	0.000	0.004
1	5	<0.02	0.000	0.002	<0.05	0.000	0.004
1	6	0.034	0.003	0.003	<0.05	0.000	0.004
1	7	0.023	0.002	0.002	<0.05	0.000	0.004
1	8	<0.02	0.000	0.002	<0.05	0.000	0.004
2	1	0.081	0.007	0.007	<0.05	0.000	0.004
2	2	0.032	0.003	0.003	<0.05	0.000	0.004
2	3	0.023	0.002	0.002	<0.05	0.000	0.004
2	4	<0.02	0.000	0.002	<0.05	0.000	0.004
2	5	<0.02	0.000	0.002	<0.05	0.000	0.004
2	6	<0.02	0.000	0.002	<0.05	0.000	0.004
2	7	<0.02	0.000	0.002	<0.05	0.000	0.004
2	8	0.023	0.002	0.002	<0.05	0.000	0.004
3	1	0.076	0.006	0.006	<0.05	0.000	0.004
3	2	0.025	0.002	0.002	<0.05	0.000	0.004
3	3	<0.02	0.000	0.002	<0.05	0.000	0.004
3	4	<0.02	0.000	0.002	<0.05	0.000	0.004
3	5	0.029	0.002	0.002	<0.05	0.000	0.004
3	6	<0.02	0.000	0.002	<0.05	0.000	0.004
3	7	<0.02	0.000	0.002	<0.05	0.000	0.004
3	8	<0.02	0.000	0.002	<0.05	0.000	0.004
4	1	0.079	0.006	0.006	<0.05	0.000	0.004
4	2	0.022	0.002	0.002	<0.05	0.000	0.004
4	3	<0.02	0.000	0.002	<0.05	0.000	0.004
4	4	<0.02	0.000	0.002	<0.05	0.000	0.004
4	5	<0.02	0.000	0.002	<0.05	0.000	0.004
4	6	<0.02	0.000	0.002	<0.05	0.000	0.004
4	7	<0.02	0.000	0.002	<0.05	0.000	0.004
4	8	<0.02	0.000	0.002	<0.05	0.000	0.004
5	1	0.071	0.006	0.006	<0.05	0.000	0.004
5	2	0.027	0.002	0.002	<0.05	0.000	0.004
5	3	<0.02	0.000	0.002	<0.05	0.000	0.004
5	4	0.029	0.002	0.002	<0.05	0.000	0.004
5	5	0.022	0.002	0.002	<0.05	0.000	0.004
5	6	<0.02	0.000	0.002	<0.05	0.000	0.004
5	7	<0.02	0.000	0.002	<0.05	0.000	0.004
5	8	0.028	0.002	0.002	<0.05	0.000	0.004
			∑ min	∑ max		∑ min	∑ max
1			0.019	0.024		0.000	0.032
2			0.013	0.019		0.000	0.032
3			0.010	0.018		0.000	0.032
4			0.008	0.018		0.000	0.032
5			0.014	0.019		0.000	0.032
Mean			0.013	0.020		0.000	0.032
SD			0.004	0.002		0.000	0.000

Table B. Continued Karbon Quartzite.

Stone	Fraction	LAB 1 Zn µg/l	min r mg/m²	max r mg/m²	LAB 2 Zn µg/l	min r mg/m²	max r mg/m²
1	1	5	0.4	0.4	<0.47	0.00	0.04
1	2	432	34.5	34.5	<0.47	0.00	0.04
1	3	285	22.8	22.8	1.08	0.09	0.09
1	4	425	34.0	34.0	<0.47	0.00	0.04
1	5	427	34.1	34.1	<0.47	0.00	0.04
1	6	414	33.1	33.1	<0.47	0.00	0.04
1	7	468	37.4	37.4	<0.47	0.00	0.04
1	8	335	26.8	26.8	<0.47	0.00	0.04
2	1	3	0.2	0.2	<0.47	0.00	0.04
2	2	438	35.0	35.0	<0.47	0.00	0.04
2	3	273	21.9	21.9	0.64	0.05	0.05
2	4	29	2.3	2.3	<0.47	0.00	0.04
2	5	434	34.7	34.7	<0.47	0.00	0.04
2	6	224	17.9	17.9	<0.47	0.00	0.04
2	7	480	38.4	38.4	<0.47	0.00	0.04
2	8	249	19.9	19.9	<0.47	0.00	0.04
3	1	11	0.9	0.9	1.01	0.08	0.08
3	2	441	35.3	35.3	<0.47	0.00	0.04
3	3	99	7.9	7.9	<0.47	0.00	0.04
3	4	42	3.4	3.4	<0.47	0.00	0.04
3	5	527	42.2	42.2	<0.47	0.00	0.04
3	6	309	24.7	24.7	<0.47	0.00	0.04
3	7	498	39.9	39.9	1.05	0.08	0.08
3	8	291	23.3	23.3	1.27	0.10	0.10
4	1	27	2.1	2.1	<0.47	0.00	0.04
4	2	310	24.8	24.8	<0.47	0.00	0.04
4	3	468	37.4	37.4	<0.47	0.00	0.04
4	4	323	25.9	25.9	<0.47	0.00	0.04
4	5	469	37.6	37.6	<0.47	0.00	0.04
4	6	466	37.2	37.2	<0.47	0.00	0.04
4	7	473	37.8	37.8	<0.47	0.00	0.04
4	8	367	29.4	29.4	<0.47	0.00	0.04
5	1	4	0.3	0.3	<0.47	0.00	0.04
5	2	267	21.4	21.4	<0.47	0.00	0.04
5	3	35	2.8	2.8	<0.47	0.00	0.04
5	4	286	22.9	22.9	<0.47	0.00	0.04
5	5	328	26.2	26.2	<0.47	0.00	0.04
5	6	287	22.9	22.9	<0.47	0.00	0.04
5	7	392	31.4	31.4	<0.47	0.00	0.04
5	8	294	23.5	23.5	<0.47	0.00	0.04
			∑ min	∑ max		∑ min	∑ max
1			223.2	223.2		0.09	0.35
2			170.3	170.3		0.05	0.31
3			177.5	177.5		0.27	0.45
4			232.1	232.1		0.00	0.30
5			151.4	151.4		0.00	0.30
Mean			190.9	190.9		0.08	0.34
SD			35.0	35.0		0.11	0.06

Table C. DSLT results Basalt (LAB 1 DSLT contract laboratory, LAB 2 DSLT BfG).

Stone	Fraction	LAB 1 Al µg/l	min r mg/m²	max r mg/m²	LAB 2 Al µg/l	min r mg/m²	max r mg/m²
1	1	<40.8	0.00	3.26	39.96	3.20	3.20
1	2	<40.8	0.00	3.26	36.97	2.96	2.96
1	3	<40.8	0.00	3.26	40.53	3.24	3.24
1	4	<40.8	0.00	3.26	46.24	3.70	3.70
1	5	<40.8	0.00	3.26	61.32	4.91	4.91
1	6	<40.8	0.00	3.26	64.39	5.15	5.15
1	7	<40.8	0.00	3.26	56.51	4.52	4.52
1	8	<40.8	0.00	3.26	60.97	4.88	4.88
2	1	<40.8	0.00	3.26	61.91	4.88	4.88
2	2	<40.8	0.00	3.26	33.16	2.61	2.61
2	3	<40.8	0.00	3.26	27.30	2.15	2.15
2	4	<40.8	0.00	3.26	23.74	1.87	1.87
2	5	<40.8	0.00	3.26	32.91	2.59	2.59
2	6	<40.8	0.00	3.26	32.16	2.53	2.53
2	7	<40.8	0.00	3.26	31.65	2.49	2.49
2	8	126.2	10.10	10.10	24.59	1.94	1.94
3	1	60.83	4.87	4.87	196.70	15.75	15.75
3	2	<40.8	0.00	3.26	78.87	6.31	6.31
3	3	<40.8	0.00	3.26	36.70	2.94	2.94
3	4	<40.8	0.00	3.26	31.04	2.48	2.48
3	5	<40.8	0.00	3.26	40.40	3.23	3.23
3	6	<40.8	0.00	3.26	49.55	3.97	3.97
3	7	<40.8	0.00	3.26	38.23	3.06	3.06
3	8	<40.8	0.00	3.26	27.59	2.21	2.21
4	1	111.6	8.93	8.93	24.26	1.94	1.94
4	2	<40.8	0.00	3.26	24.32	1.95	1.95
4	3	<40.8	0.00	3.26	27.05	2.16	2.16
4	4	<40.8	0.00	3.26	29.98	2.40	2.40
4	5	<40.8	0.00	3.26	40.15	3.21	3.21
4	6	<40.8	0.00	3.26	41.95	3.36	3.36
4	7	<40.8	0.00	3.26	41.10	3.29	3.29
4	8	<40.8	0.00	3.26	48.30	3.87	3.87
5	1	<40.8	0.00	3.26	93.10	7.45	7.45
5	2	<40.8	0.00	3.26	47.69	3.82	3.82
5	3	<40.8	0.00	3.26	38.03	3.04	3.04
5	4	<40.8	0.00	3.26	32.41	2.59	2.59
5	5	<40.8	0.00	3.26	31.46	2.52	2.52
5	6	<40.8	0.00	3.26	36.97	2.96	2.96
5	7	<40.8	0.00	3.26	31.18	2.50	2.50
5	8	172.5	13.80	13.80	24.23	1.94	1.94
			∑ min	∑ max		∑ min	∑ max
1			0.00	26.11		32.55	32.55
2			10.10	32.95		21.08	21.08
3			4.87	27.71		39.95	39.95
4			8.93	31.77		22.18	22.18
5			13.80	36.64		26.82	26.82
Mean			7.54	31.04		28.51	28.51
SD			5.28	4.21		7.83	7.83

Table C. Continued Basalt.

Stone	Fraction	LAB 1 As µg/l	min r mg/m²	max r mg/m²	LAB 2 As µg/l	min r mg/m²	max r mg/m²
1	1	0.05	0.004	0.004	<0.04	0.000	0.003
1	2	0.06	0.005	0.005	<0.04	0.000	0.003
1	3	0.04	0.003	0.003	<0.04	0.000	0.003
1	4	0.04	0.003	0.003	<0.04	0.000	0.003
1	5	0.05	0.004	0.004	<0.04	0.000	0.003
1	6	0.06	0.005	0.005	<0.04	0.000	0.003
1	7	0.06	0.004	0.004	<0.04	0.000	0.003
1	8	0.07	0.006	0.006	<0.04	0.000	0.003
2	1	0.06	0.005	0.005	<0.04	0.000	0.003
2	2	0.04	0.003	0.003	<0.04	0.000	0.003
2	3	<0.03	0.000	0.002	<0.04	0.000	0.003
2	4	0.04	0.003	0.003	<0.04	0.000	0.003
2	5	0.06	0.005	0.005	<0.04	0.000	0.003
2	6	0.08	0.006	0.006	<0.04	0.000	0.003
2	7	0.08	0.007	0.007	<0.04	0.000	0.003
2	8	0.13	0.010	0.010	<0.04	0.000	0.003
3	1	0.07	0.006	0.006	<0.04	0.000	0.003
3	2	0.05	0.004	0.004	<0.04	0.000	0.003
3	3	0.04	0.003	0.003	<0.04	0.000	0.003
3	4	0.05	0.004	0.004	<0.04	0.000	0.003
3	5	0.06	0.005	0.005	<0.04	0.000	0.003
3	6	0.07	0.005	0.005	<0.04	0.000	0.003
3	7	0.07	0.005	0.005	<0.04	0.000	0.003
3	8	0.11	0.008	0.008	<0.04	0.000	0.003
4	1	0.09	0.007	0.007	<0.04	0.000	0.003
4	2	0.05	0.004	0.004	<0.04	0.000	0.003
4	3	0.03	0.003	0.003	<0.04	0.000	0.003
4	4	0.03	0.003	0.003	<0.04	0.000	0.003
4	5	0.04	0.003	0.003	<0.04	0.000	0.003
4	6	0.05	0.004	0.004	<0.04	0.000	0.003
4	7	0.05	0.004	0.004	<0.04	0.000	0.003
4	8	0.06	0.005	0.005	<0.04	0.000	0.003
5	1	0.06	0.005	0.005	<0.04	0.000	0.003
5	2	0.06	0.005	0.005	<0.04	0.000	0.003
5	3	<0.03	0.000	0.002	<0.04	0.000	0.003
5	4	0.05	0.004	0.004	<0.04	0.000	0.003
5	5	0.06	0.005	0.005	<0.04	0.000	0.003
5	6	0.07	0.006	0.006	<0.04	0.000	0.003
5	7	0.07	0.005	0.005	<0.04	0.000	0.003
5	8	0.12	0.010	0.010	<0.04	0.000	0.003
			∑ min	∑ max		∑ min	∑ max
1			0.034	0.034		0.000	0.026
2			0.040	0.042		0.000	0.025
3			0.040	0.040		0.000	0.026
4			0.032	0.032		0.000	0.026
5			0.039	0.041		0.000	0.026
Mean			0.037	0.038		0.000	0.026
SD			0.004	0.004		0.000	0.000

Table C. Continued Basalt.

Stone	Fraction	LAB 1 Ba µg/l	min r mg/m^2	max r mg/m^2	LAB 2 Ba µg/l	min r mg/m^2	max r mg/m^2
1	1	0.7	0.1	0.1	0.56	0.05	0.05
1	2	400.1	32.0	32.0	0.58	0.05	0.05
1	3	369.2	29.5	29.5	0.52	0.04	0.04
1	4	437.5	35.0	35.0	0.45	0.04	0.04
1	5	451.5	36.1	36.1	<0.31	0.00	0.02
1	6	463.6	37.1	37.1	<0.31	0.00	0.02
1	7	477.4	38.2	38.2	<0.31	0.00	0.02
1	8	363.8	29.1	29.1	<0.31	0.00	0.02
2	1	0.6	0.1	0.1	<0.31	0.00	0.02
2	2	460.4	36.8	36.8	<0.31	0.00	0.02
2	3	46.4	3.7	3.7	<0.31	0.00	0.02
2	4	475.2	38.0	38.0	<0.31	0.00	0.02
2	5	513.7	41.1	41.1	<0.31	0.00	0.02
2	6	492.8	39.4	39.4	<0.31	0.00	0.02
2	7	412.7	33.0	33.0	<0.31	0.00	0.02
2	8	386.6	30.9	30.9	<0.31	0.00	0.02
3	1	0.7	0.1	0.1	<0.31	0.00	0.02
3	2	412.9	33.0	33.0	<0.31	0.00	0.02
3	3	393.4	31.5	31.5	<0.31	0.00	0.02
3	4	443.6	35.5	35.5	<0.31	0.00	0.02
3	5	437.8	35.0	35.0	<0.31	0.00	0.02
3	6	438.8	35.1	35.1	<0.31	0.00	0.02
3	7	141.7	11.3	11.3	<0.31	0.00	0.02
3	8	437.1	35.0	35.0	<0.31	0.00	0.02
4	1	0.9	0.1	0.1	<0.31	0.00	0.02
4	2	392.9	31.4	31.4	<0.31	0.00	0.02
4	3	392.4	31.4	31.4	<0.31	0.00	0.02
4	4	385.1	30.8	30.8	<0.31	0.00	0.02
4	5	464.8	37.2	37.2	<0.31	0.00	0.02
4	6	404.1	32.3	32.3	<0.31	0.00	0.02
4	7	478.3	38.3	38.3	<0.31	0.00	0.02
4	8	359.7	28.8	28.8	<0.31	0.00	0.02
5	1	0.7	0.1	0.1	<0.31	0.00	0.02
5	2	371.4	29.7	29.7	<0.31	0.00	0.02
5	3	30.3	2.4	2.4	<0.31	0.00	0.02
5	4	446.1	35.7	35.7	<0.31	0.00	0.02
5	5	499.8	40.0	40.0	<0.31	0.00	0.02
5	6	493.5	39.5	39.5	<0.31	0.00	0.02
5	7	162.3	13.0	13.0	<0.31	0.00	0.02
5	8	575.8	46.1	46.1	<0.31	0.00	0.02
			∑ min	∑ max		∑ min	∑ max
1			237.1	237.1		0.17	0.27
2			223.1	223.1		0.00	0.20
3			216.5	216.5		0.00	0.20
4			230.2	230.2		0.00	0.20
5			206.4	206.4		0.00	0.20
Mean			222.7	222.7		0.03	0.21
SD			11.9	11.9		0.08	0.03

Table C. Continued Basalt.

Stone	Fraction	LAB 1 Cd µg/l	min r mg/m²	max r mg/m²	LAB 2 Cd µg/l	min r mg/m²	max r mg/m²
1	1	<0.01	0.000	0.001	<0.03	0.000	0.002
1	2	0.023	0.002	0.002	<0.03	0.000	0.002
1	3	0.024	0.002	0.002	<0.03	0.000	0.002
1	4	0.023	0.002	0.002	<0.03	0.000	0.002
1	5	0.023	0.002	0.002	<0.03	0.000	0.002
1	6	0.026	0.002	0.002	<0.03	0.000	0.002
1	7	0.029	0.002	0.002	<0.03	0.000	0.002
1	8	0.025	0.002	0.002	<0.03	0.000	0.002
2	1	0.008	0.001	0.001	<0.03	0.000	0.002
2	2	0.029	0.002	0.002	<0.03	0.000	0.002
2	3	<0.01	0.000	0.001	<0.03	0.000	0.002
2	4	0.029	0.002	0.002	<0.03	0.000	0.002
2	5	0.030	0.002	0.002	<0.03	0.000	0.002
2	6	0.036	0.003	0.003	<0.03	0.000	0.002
2	7	0.033	0.003	0.003	<0.03	0.000	0.002
2	8	0.031	0.002	0.002	<0.03	0.000	0.002
3	1	0.014	0.001	0.001	<0.03	0.000	0.002
3	2	0.019	0.002	0.002	<0.03	0.000	0.002
3	3	0.021	0.002	0.002	<0.03	0.000	0.002
3	4	0.025	0.002	0.002	<0.03	0.000	0.002
3	5	0.024	0.002	0.002	<0.03	0.000	0.002
3	6	0.027	0.002	0.002	<0.03	0.000	0.002
3	7	0.015	0.001	0.001	<0.03	0.000	0.002
3	8	0.025	0.002	0.002	<0.03	0.000	0.002
4	1	0.012	0.001	0.001	<0.03	0.000	0.002
4	2	0.028	0.002	0.002	<0.03	0.000	0.002
4	3	0.026	0.002	0.002	<0.03	0.000	0.002
4	4	0.026	0.002	0.002	<0.03	0.000	0.002
4	5	0.028	0.002	0.002	<0.03	0.000	0.002
4	6	0.024	0.002	0.002	<0.03	0.000	0.002
4	7	0.031	0.002	0.002	<0.03	0.000	0.002
4	8	0.026	0.002	0.002	<0.03	0.000	0.002
5	1	<0.01	0.000	0.001	<0.03	0.000	0.002
5	2	0.028	0.002	0.002	<0.03	0.000	0.002
5	3	<0.01	0.000	0.001	<0.03	0.000	0.002
5	4	0.022	0.002	0.002	<0.03	0.000	0.002
5	5	0.028	0.002	0.002	<0.03	0.000	0.002
5	6	0.027	0.002	0.002	<0.03	0.000	0.002
5	7	0.011	0.001	0.001	<0.03	0.000	0.002
5	8	0.030	0.002	0.002	<0.03	0.000	0.002
			∑ min	∑ max		∑ min	∑ max
1			0.014	0.015		0.000	0.019
2			0.016	0.017		0.000	0.019
3			0.013	0.013		0.000	0.019
4			0.016	0.016		0.000	0.019
5			0.012	0.013		0.000	0.019
Mean			0.014	0.015		0.000	0.019
SD			0.002	0.001		0.000	0.000

Table C. Continued Basalt.

Stone	Fraction	LAB 1 Co µg/l	min r mg/m^2	max r mg/m^2	LAB 2 Co µg/l	min r mg/m^2	max r mg/m^2
1	1	0.017	0.001	0.001	<0.03	0.000	0.002
1	2	0.074	0.006	0.006	<0.03	0.000	0.002
1	3	0.064	0.005	0.005	<0.03	0.000	0.002
1	4	0.065	0.005	0.005	<0.03	0.000	0.002
1	5	0.072	0.006	0.006	<0.03	0.000	0.002
1	6	0.076	0.006	0.006	<0.03	0.000	0.002
1	7	0.072	0.006	0.006	<0.03	0.000	0.002
1	8	0.115	0.009	0.009	<0.03	0.000	0.002
2	1	0.018	0.001	0.001	<0.03	0.000	0.002
2	2	0.082	0.007	0.007	<0.03	0.000	0.002
2	3	0.020	0.000	0.002	<0.03	0.000	0.002
2	4	0.077	0.006	0.006	<0.03	0.000	0.002
2	5	0.081	0.006	0.006	<0.03	0.000	0.002
2	6	0.080	0.006	0.006	<0.03	0.000	0.002
2	7	0.068	0.005	0.005	<0.03	0.000	0.002
2	8	0.122	0.010	0.010	<0.03	0.000	0.002
3	1	0.039	0.003	0.003	<0.03	0.000	0.002
3	2	0.075	0.006	0.006	<0.03	0.000	0.002
3	3	0.065	0.005	0.005	<0.03	0.000	0.002
3	4	0.073	0.006	0.006	<0.03	0.000	0.002
3	5	0.070	0.006	0.006	<0.03	0.000	0.002
3	6	0.075	0.006	0.006	<0.03	0.000	0.002
3	7	0.048	0.004	0.004	<0.03	0.000	0.002
3	8	0.122	0.010	0.010	<0.03	0.000	0.002
4	1	0.041	0.003	0.003	<0.03	0.000	0.002
4	2	0.073	0.006	0.006	<0.03	0.000	0.002
4	3	0.065	0.005	0.005	<0.03	0.000	0.002
4	4	0.070	0.006	0.006	<0.03	0.000	0.002
4	5	0.071	0.006	0.006	<0.03	0.000	0.002
4	6	0.078	0.006	0.006	<0.03	0.000	0.002
4	7	0.078	0.006	0.006	<0.03	0.000	0.002
4	8	0.113	0.009	0.009	<0.03	0.000	0.002
5	1	0.020	0.000	0.002	<0.03	0.000	0.002
5	2	0.089	0.007	0.007	<0.03	0.000	0.002
5	3	0.020	0.000	0.002	<0.03	0.000	0.002
5	4	0.071	0.006	0.006	<0.03	0.000	0.002
5	5	0.074	0.006	0.006	<0.03	0.000	0.002
5	6	0.072	0.006	0.006	<0.03	0.000	0.002
5	7	0.029	0.002	0.002	<0.03	0.000	0.002
5	8	0.115	0.009	0.009	<0.03	0.000	0.002
			∑ min	∑ max		∑ min	∑ max
1			0.044	0.044		0.000	0.019
2			0.042	0.044		0.000	0.019
3			0.045	0.045		0.000	0.019
4			0.047	0.047		0.000	0.019
5			0.036	0.039		0.000	0.019
Mean			0.043	0.044		0.000	0.019
SD			0.004	0.003		0.000	0.000

Table C. Continued Basalt.

Stone	Fraction	LAB 1 Cr µg/l	min r mg/m^2	max r mg/m^2	LAB 2 Cr µg/l	min r mg/m^2	max r mg/m^2
1	1	0.079	0.006	0.006	<0.11	0.000	0.009
1	2	0.063	0.005	0.005	<0.11	0.000	0.009
1	3	0.063	0.005	0.005	<0.11	0.000	0.009
1	4	0.068	0.005	0.005	<0.11	0.000	0.009
1	5	0.078	0.006	0.006	<0.11	0.000	0.009
1	6	0.139	0.011	0.011	<0.11	0.000	0.009
1	7	<0.06	0.000	0.005	<0.11	0.000	0.009
1	8	<0.06	0.000	0.005	<0.11	0.000	0.009
2	1	0.092	0.007	0.007	<0.11	0.000	0.009
2	2	0.077	0.006	0.006	<0.11	0.000	0.009
2	3	<0.06	0.000	0.005	<0.11	0.000	0.009
2	4	0.100	0.008	0.008	<0.11	0.000	0.009
2	5	<0.06	0.000	0.005	<0.11	0.000	0.009
2	6	0.082	0.007	0.007	<0.11	0.000	0.009
2	7	<0.06	0.000	0.005	<0.11	0.000	0.009
2	8	0.114	0.009	0.009	<0.11	0.000	0.009
3	1	0.106	0.009	0.009	<0.11	0.000	0.009
3	2	<0.06	0.000	0.005	<0.11	0.000	0.009
3	3	0.064	0.005	0.005	<0.11	0.000	0.009
3	4	0.071	0.006	0.006	<0.11	0.000	0.009
3	5	0.091	0.007	0.007	<0.11	0.000	0.009
3	6	0.084	0.007	0.007	<0.11	0.000	0.009
3	7	<0.06	0.000	0.005	<0.11	0.000	0.009
3	8	<0.06	0.000	0.005	<0.11	0.000	0.009
4	1	0.119	0.009	0.009	<0.11	0.000	0.009
4	2	0.071	0.006	0.006	<0.11	0.000	0.009
4	3	0.067	0.005	0.005	<0.11	0.000	0.009
4	4	0.059	0.005	0.005	<0.11	0.000	0.009
4	5	0.059	0.005	0.005	<0.11	0.000	0.009
4	6	0.083	0.007	0.007	<0.11	0.000	0.009
4	7	0.065	0.005	0.005	<0.11	0.000	0.009
4	8	<0.06	0.000	0.005	<0.11	0.000	0.009
5	1	0.090	0.007	0.007	<0.11	0.000	0.009
5	2	0.070	0.006	0.006	<0.11	0.000	0.009
5	3	<0.06	0.000	0.005	<0.11	0.000	0.009
5	4	0.063	0.005	0.005	<0.11	0.000	0.009
5	5	0.079	0.006	0.006	<0.11	0.000	0.009
5	6	<0.06	0.000	0.005	<0.11	0.000	0.009
5	7	<0.06	0.000	0.005	<0.11	0.000	0.009
5	8	0.181	0.015	0.015	<0.11	0.000	0.009
			∑ min	∑ max		∑ min	∑ max
1			0.039	0.049		0.000	0.070
2			0.037	0.052		0.000	0.070
3			0.033	0.048		0.000	0.070
4			0.042	0.047		0.000	0.070
5			0.039	0.053		0.000	0.070
Mean			0.038	0.050		0.000	0.070
SD			0.003	0.003		0.000	0.000

Table C. Continued Basalt.

Stone	Fraction	LAB 1 Cu µg/l	min r mg/m^2	max r mg/m^2	LAB 2 Cu µg/l	min r mg/m^2	max r mg/m^2
1	1	0.99	0.08	0.08	0.99	0.08	0.08
1	2	2.09	0.17	0.17	1.04	0.08	0.08
1	3	2.31	0.18	0.18	0.23	0.02	0.02
1	4	1.67	0.13	0.13	<0.10	0.00	0.01
1	5	1.89	0.15	0.15	<0.10	0.00	0.01
1	6	2.05	0.16	0.16	0.14	0.01	0.01
1	7	1.98	0.16	0.16	<0.10	0.00	0.01
1	8	1.79	0.14	0.14	<0.10	0.00	0.01
2	1	1.97	0.16	0.16	0.51	0.04	0.04
2	2	3.79	0.30	0.30	0.20	0.02	0.02
2	3	1.66	0.13	0.13	0.29	0.02	0.02
2	4	3.00	0.24	0.24	<0.10	0.00	0.01
2	5	2.49	0.20	0.20	<0.10	0.00	0.01
2	6	2.45	0.20	0.20	<0.10	0.00	0.01
2	7	1.67	0.13	0.13	<0.10	0.00	0.01
2	8	1.41	0.11	0.11	<0.10	0.00	0.01
3	1	3.60	0.29	0.29	1.17	0.09	0.09
3	2	2.46	0.20	0.20	7.71	0.62	0.62
3	3	2.61	0.21	0.21	1.15	0.09	0.09
3	4	1.91	0.15	0.15	<0.10	0.00	0.01
3	5	2.31	0.19	0.19	<0.10	0.00	0.01
3	6	2.36	0.19	0.19	<0.10	0.00	0.01
3	7	1.82	0.15	0.15	<0.10	0.00	0.01
3	8	2.21	0.18	0.18	<0.10	0.00	0.01
4	1	0.59	0.05	0.05	0.78	0.06	0.06
4	2	2.79	0.22	0.22	0.39	0.03	0.03
4	3	2.65	0.21	0.21	0.34	0.03	0.03
4	4	1.72	0.14	0.14	<0.10	0.00	0.01
4	5	1.97	0.16	0.16	<0.10	0.00	0.01
4	6	2.20	0.18	0.18	<0.10	0.00	0.01
4	7	2.20	0.18	0.18	<0.10	0.00	0.01
4	8	1.20	0.10	0.10	<0.10	0.00	0.01
5	1	0.98	0.08	0.08	0.52	0.04	0.04
5	2	3.91	0.31	0.31	<0.10	0.00	0.01
5	3	0.71	0.06	0.06	4.28	0.34	0.34
5	4	2.39	0.19	0.19	<0.10	0.00	0.01
5	5	2.04	0.16	0.16	<0.10	0.00	0.01
5	6	1.48	0.12	0.12	<0.10	0.00	0.01
5	7	1.25	0.10	0.10	0.10	0.01	0.01
5	8	1.34	0.11	0.11	<0.10	0.00	0.01
			∑ min	∑ max		∑ min	∑ max
1			1.18	1.18		0.19	0.22
2			1.48	1.48		0.08	0.12
3			1.54	1.54		0.80	0.84
4			1.23	1.23		0.12	0.16
5			1.13	1.13		0.39	0.43
Mean			1.31	1.31		0.32	0.36
SD			0.19	0.19		0.30	0.30

Table C. Continued Basalt.

Stone	Fraction	LAB 1 Mn µg/l	min r mg/m²	max r mg/m²	LAB 2 Mn µg/l	min r mg/m²	max r mg/m²
1	1	1.14	0.09	0.09	0.47	0.04	0.04
1	2	0.38	0.03	0.03	0.56	0.05	0.05
1	3	0.28	0.02	0.02	0.54	0.04	0.04
1	4	0.32	0.03	0.03	0.44	0.03	0.03
1	5	0.46	0.04	0.04	0.71	0.06	0.06
1	6	0.56	0.04	0.04	0.54	0.04	0.04
1	7	0.82	0.07	0.07	0.35	0.03	0.03
1	8	0.76	0.06	0.06	<0.22	0.00	0.02
2	1	1.27	0.10	0.10	<0.22	0.00	0.02
2	2	0.65	0.05	0.05	<0.22	0.00	0.02
2	3	0.43	0.03	0.03	<0.22	0.00	0.02
2	4	0.62	0.05	0.05	<0.22	0.00	0.02
2	5	0.64	0.05	0.05	<0.22	0.00	0.02
2	6	0.66	0.05	0.05	<0.22	0.00	0.02
2	7	0.69	0.06	0.06	0.33	0.03	0.03
2	8	0.89	0.07	0.07	<0.22	0.00	0.02
3	1	3.23	0.26	0.26	<0.22	0.00	0.02
3	2	0.38	0.03	0.03	<0.22	0.00	0.02
3	3	0.31	0.02	0.02	<0.22	0.00	0.02
3	4	0.30	0.02	0.02	<0.22	0.00	0.02
3	5	0.46	0.04	0.04	<0.22	0.00	0.02
3	6	0.59	0.05	0.05	0.37	0.03	0.03
3	7	1.11	0.09	0.09	1.02	0.08	0.08
3	8	1.50	0.12	0.12	0.63	0.05	0.05
4	1	2.95	0.24	0.24	<0.22	0.00	0.02
4	2	0.36	0.03	0.03	<0.22	0.00	0.02
4	3	0.30	0.02	0.02	<0.22	0.00	0.02
4	4	0.33	0.03	0.03	<0.22	0.00	0.02
4	5	0.39	0.03	0.03	<0.22	0.00	0.02
4	6	0.44	0.04	0.04	<0.22	0.00	0.02
4	7	0.65	0.05	0.05	<0.22	0.00	0.02
4	8	0.63	0.05	0.05	<0.22	0.00	0.02
5	1	1.51	0.12	0.12	0.33	0.03	0.03
5	2	0.38	0.03	0.03	<0.22	0.00	0.02
5	3	0.32	0.03	0.03	<0.22	0.00	0.02
5	4	0.31	0.02	0.02	<0.22	0.00	0.02
5	5	0.47	0.04	0.04	0.39	0.03	0.03
5	6	0.58	0.05	0.05	0.42	0.03	0.03
5	7	0.59	0.05	0.05	0.90	0.07	0.07
5	8	0.94	0.07	0.07	0.75	0.06	0.06
			∑ min	∑ max		∑ min	∑ max
1			0.38	0.38		0.29	0.31
2			0.47	0.47		0.03	0.15
3			0.63	0.63		0.16	0.25
4			0.48	0.48		0.00	0.14
5			0.41	0.41		0.22	0.28
Mean			0.47	0.47		0.14	0.22
SD			0.10	0.10		0.12	0.08

Table C. Continued Basalt.

Stone	Fraction	LAB 1 Mo µg/l	min r mg/m^2	max r mg/m^2	LAB 2 Mo µg/l	min r mg/m^2	max r mg/m^2
1	1	0.15	0.01	0.01	<0.21	0.00	0.02
1	2	0.13	0.01	0.01	<0.21	0.00	0.02
1	3	0.08	0.01	0.01	<0.21	0.00	0.02
1	4	0.09	0.01	0.01	<0.21	0.00	0.02
1	5	0.22	0.02	0.02	<0.21	0.00	0.02
1	6	0.29	0.02	0.02	<0.21	0.00	0.02
1	7	0.35	0.03	0.03	<0.21	0.00	0.02
1	8	0.49	0.04	0.04	<0.21	0.00	0.02
2	1	<0.06	0.00	0.00	<0.21	0.00	0.02
2	2	<0.06	0.00	0.00	<0.21	0.00	0.02
2	3	<0.06	0.00	0.00	<0.21	0.00	0.02
2	4	0.07	0.01	0.01	<0.21	0.00	0.02
2	5	0.18	0.01	0.01	<0.21	0.00	0.02
2	6	0.34	0.03	0.03	<0.21	0.00	0.02
2	7	0.53	0.04	0.04	<0.21	0.00	0.02
2	8	1.01	0.08	0.08	<0.21	0.00	0.02
3	1	0.13	0.01	0.01	<0.21	0.00	0.02
3	2	0.12	0.01	0.01	<0.21	0.00	0.02
3	3	0.12	0.01	0.01	<0.21	0.00	0.02
3	4	0.13	0.01	0.01	<0.21	0.00	0.02
3	5	0.31	0.02	0.02	<0.21	0.00	0.02
3	6	0.46	0.04	0.04	<0.21	0.00	0.02
3	7	0.65	0.05	0.05	<0.21	0.00	0.02
3	8	1.17	0.09	0.09	<0.21	0.00	0.02
4	1	0.11	0.01	0.01	<0.21	0.00	0.02
4	2	<0.06	0.00	0.00	<0.21	0.00	0.02
4	3	<0.06	0.00	0.00	<0.21	0.00	0.02
4	4	0.08	0.01	0.01	<0.21	0.00	0.02
4	5	0.09	0.01	0.01	<0.21	0.00	0.02
4	6	0.12	0.01	0.01	<0.21	0.00	0.02
4	7	0.13	0.01	0.01	<0.21	0.00	0.02
4	8	0.20	0.02	0.02	<0.21	0.00	0.02
5	1	0.12	0.01	0.01	<0.21	0.00	0.02
5	2	0.10	0.01	0.01	<0.21	0.00	0.02
5	3	0.07	0.01	0.01	<0.21	0.00	0.02
5	4	0.09	0.01	0.01	<0.21	0.00	0.02
5	5	0.24	0.02	0.02	<0.21	0.00	0.02
5	6	0.37	0.03	0.03	<0.21	0.00	0.02
5	7	0.55	0.04	0.04	<0.21	0.00	0.02
5	8	0.93	0.07	0.07	<0.21	0.00	0.02
			∑ min	∑ max		∑ min	∑ max
1			0.14	0.14		0.00	0.13
2			0.17	0.18		0.00	0.13
3			0.25	0.25		0.00	0.13
4			0.06	0.07		0.00	0.13
5			0.20	0.20		0.00	0.13
Mean			0.16	0.17		0.00	0.13
SD			0.07	0.07		0.00	0.00

Table C. Continued Basalt.

Stone	Fraction	LAB 1 Ni µg/l	min r mg/m²	max r mg/m²	LAB 2 Ni µg/l	min r mg/m²	max r mg/m²
1	1	0.180	0.014	0.014	0.101	0.008	0.008
1	2	0.286	0.023	0.023	<0.04	0.000	0.003
1	3	0.223	0.018	0.018	<0.04	0.000	0.003
1	4	0.245	0.020	0.020	<0.04	0.000	0.003
1	5	0.246	0.020	0.020	<0.04	0.000	0.003
1	6	0.340	0.027	0.027	<0.04	0.000	0.003
1	7	0.354	0.028	0.028	<0.04	0.000	0.003
1	8	0.251	0.020	0.020	<0.04	0.000	0.003
2	1	0.363	0.029	0.029	<0.04	0.000	0.003
2	2	0.594	0.048	0.048	<0.04	0.000	0.003
2	3	0.176	0.014	0.014	<0.04	0.000	0.003
2	4	0.600	0.048	0.048	<0.04	0.000	0.003
2	5	0.511	0.041	0.041	<0.04	0.000	0.003
2	6	0.272	0.022	0.022	<0.04	0.000	0.003
2	7	0.223	0.018	0.018	<0.04	0.000	0.003
2	8	0.260	0.021	0.021	<0.04	0.000	0.003
3	1	0.539	0.043	0.043	<0.04	0.000	0.003
3	2	0.323	0.026	0.026	<0.04	0.000	0.003
3	3	0.235	0.019	0.019	<0.04	0.000	0.003
3	4	0.251	0.020	0.020	<0.04	0.000	0.003
3	5	0.257	0.021	0.021	<0.04	0.000	0.003
3	6	0.330	0.026	0.026	<0.04	0.000	0.003
3	7	0.305	0.024	0.024	<0.04	0.000	0.003
3	8	0.347	0.028	0.028	<0.04	0.000	0.003
4	1	0.451	0.036	0.036	<0.04	0.000	0.003
4	2	0.273	0.022	0.022	<0.04	0.000	0.003
4	3	0.230	0.018	0.018	<0.04	0.000	0.003
4	4	0.231	0.018	0.018	<0.04	0.000	0.003
4	5	0.238	0.019	0.019	<0.04	0.000	0.003
4	6	0.251	0.020	0.020	<0.04	0.000	0.003
4	7	0.294	0.024	0.024	<0.04	0.000	0.003
4	8	0.190	0.015	0.015	<0.04	0.000	0.003
5	1	0.285	0.023	0.023	<0.04	0.000	0.003
5	2	0.360	0.029	0.029	<0.04	0.000	0.003
5	3	0.100	0.008	0.008	<0.04	0.000	0.003
5	4	0.322	0.026	0.026	<0.04	0.000	0.003
5	5	0.382	0.031	0.031	<0.04	0.000	0.003
5	6	0.234	0.019	0.019	<0.04	0.000	0.003
5	7	0.126	0.010	0.010	<0.04	0.000	0.003
5	8	0.208	0.017	0.017	<0.04	0.000	0.003
			∑ min	∑ max		∑ min	∑ max
1			0.170	0.170		0.008	0.030
2			0.240	0.240		0.000	0.025
3			0.207	0.207		0.000	0.026
4			0.173	0.173		0.000	0.026
5			0.161	0.161		0.000	0.026
Mean			0.190	0.190		0.002	0.027
SD			0.033	0.033		0.004	0.002

Table C. Continued Basalt.

Stone	Fraction	LAB 1 Pb µg/l	min r mg/m²	max r mg/m²	LAB 2 Pb µg/l	min r mg/m²	max r mg/m²
1	1	0.108	0.009	0.009	0.093	0.007	0.007
1	2	0.104	0.008	0.008	0.063	0.005	0.005
1	3	0.105	0.008	0.008	<0.03	0.000	0.002
1	4	0.116	0.009	0.009	<0.03	0.000	0.002
1	5	0.168	0.013	0.013	<0.03	0.000	0.002
1	6	0.154	0.012	0.012	<0.03	0.000	0.002
1	7	0.067	0.005	0.005	<0.03	0.000	0.002
1	8	<0.03	0.000	0.002	<0.03	0.000	0.002
2	1	0.063	0.005	0.005	0.057	0.004	0.004
2	2	0.367	0.029	0.029	<0.03	0.000	0.002
2	3	0.124	0.010	0.010	0.056	0.004	0.004
2	4	0.127	0.010	0.010	<0.03	0.000	0.002
2	5	0.153	0.012	0.012	<0.03	0.000	0.002
2	6	0.105	0.008	0.008	<0.03	0.000	0.002
2	7	0.073	0.006	0.006	<0.03	0.000	0.002
2	8	0.489	0.039	0.039	<0.03	0.000	0.002
3	1	0.107	0.009	0.009	0.091	0.007	0.007
3	2	0.154	0.012	0.012	0.371	0.030	0.030
3	3	0.077	0.006	0.006	0.079	0.006	0.006
3	4	0.080	0.006	0.006	<0.03	0.000	0.002
3	5	0.106	0.008	0.008	<0.03	0.000	0.002
3	6	0.054	0.004	0.004	<0.03	0.000	0.002
3	7	0.050	0.004	0.004	<0.03	0.000	0.002
3	8	0.041	0.003	0.003	<0.03	0.000	0.002
4	1	0.130	0.010	0.010	<0.03	0.000	0.002
4	2	0.222	0.018	0.018	<0.03	0.000	0.002
4	3	0.093	0.007	0.007	<0.03	0.000	0.002
4	4	0.075	0.006	0.006	<0.03	0.000	0.002
4	5	0.077	0.006	0.006	<0.03	0.000	0.002
4	6	0.088	0.007	0.007	<0.03	0.000	0.002
4	7	0.062	0.005	0.005	<0.03	0.000	0.002
4	8	<0.03	0.000	0.002	<0.03	0.000	0.002
5	1	0.052	0.004	0.004	0.062	0.005	0.005
5	2	0.228	0.018	0.018	<0.03	0.000	0.002
5	3	0.071	0.006	0.006	0.148	0.012	0.012
5	4	0.078	0.006	0.006	<0.03	0.000	0.002
5	5	0.111	0.009	0.009	<0.03	0.000	0.002
5	6	0.084	0.007	0.007	<0.03	0.000	0.002
5	7	0.072	0.006	0.006	<0.03	0.000	0.002
5	8	0.090	0.007	0.007	<0.03	0.000	0.002
			∑ min	∑ max		∑ min	∑ max
1			0.066	0.068		0.013	0.027
2			0.120	0.120		0.009	0.023
3			0.053	0.053		0.043	0.055
4			0.060	0.062		0.000	0.019
5			0.063	0.063		0.017	0.031
Mean			0.072	0.073		0.016	0.031
SD			0.027	0.027		0.016	0.014

Table C. Continued Basalt.

Stone	Fraction	LAB 1 Sb µg/l	min r mg/m^2	max r mg/m^2	LAB 2 Sb µg/l	min r mg/m^2	max r mg/m^2
1	1	<0.06	0.000	0.005	<0.07	0.000	0.006
1	2	0.073	0.006	0.006	<0.07	0.000	0.006
1	3	0.070	0.006	0.006	<0.07	0.000	0.006
1	4	0.069	0.006	0.006	<0.07	0.000	0.006
1	5	0.069	0.006	0.006	<0.07	0.000	0.006
1	6	0.094	0.008	0.008	<0.07	0.000	0.006
1	7	0.077	0.006	0.006	<0.07	0.000	0.006
1	8	0.235	0.019	0.019	<0.07	0.000	0.006
2	1	<0.06	0.000	0.005	<0.07	0.000	0.006
2	2	0.081	0.006	0.006	<0.07	0.000	0.006
2	3	<0.06	0.000	0.005	<0.07	0.000	0.006
2	4	0.080	0.006	0.006	<0.07	0.000	0.006
2	5	0.070	0.006	0.006	<0.07	0.000	0.006
2	6	0.072	0.006	0.006	<0.07	0.000	0.006
2	7	<0.06	0.000	0.005	<0.07	0.000	0.006
2	8	0.222	0.018	0.018	<0.07	0.000	0.006
3	1	<0.06	0.000	0.005	<0.07	0.000	0.006
3	2	0.072	0.006	0.006	<0.07	0.000	0.006
3	3	0.071	0.006	0.006	<0.07	0.000	0.006
3	4	0.071	0.006	0.006	<0.07	0.000	0.006
3	5	0.072	0.006	0.006	<0.07	0.000	0.006
3	6	0.072	0.006	0.006	<0.07	0.000	0.006
3	7	<0.06	0.000	0.005	<0.07	0.000	0.006
3	8	0.201	0.016	0.016	<0.07	0.000	0.006
4	1	0.024	0.002	0.002	<0.07	0.000	0.006
4	2	0.065	0.005	0.005	<0.07	0.000	0.006
4	3	0.068	0.005	0.005	<0.07	0.000	0.006
4	4	0.081	0.006	0.006	<0.07	0.000	0.006
4	5	0.065	0.005	0.005	<0.07	0.000	0.006
4	6	0.079	0.006	0.006	<0.07	0.000	0.006
4	7	<0.06	0.000	0.005	<0.07	0.000	0.006
4	8	0.234	0.019	0.019	<0.07	0.000	0.006
5	1	<0.06	0.000	0.005	<0.07	0.000	0.006
5	2	0.156	0.013	0.013	<0.07	0.000	0.006
5	3	<0.06	0.000	0.005	<0.07	0.000	0.006
5	4	0.077	0.006	0.006	<0.07	0.000	0.006
5	5	0.079	0.006	0.006	<0.07	0.000	0.006
5	6	0.083	0.007	0.007	<0.07	0.000	0.006
5	7	<0.06	0.000	0.005	<0.07	0.000	0.006
5	8	0.264	0.021	0.021	<0.07	0.000	0.006
			∑ min	∑ max		∑ min	∑ max
1			0.055	0.060		0.000	0.045
2			0.042	0.056		0.000	0.044
3			0.045	0.054		0.000	0.045
4			0.049	0.054		0.000	0.045
5			0.053	0.067		0.000	0.045
Mean			0.049	0.058		0.000	0.045
SD			0.005	0.005		0.000	0.000

Table C. Continued Basalt.

Stone	Fraction	LAB 1 Se µg/l	min r mg/m^2	max r mg/m^2	LAB 2 Se µg/l	min r mg/m^2	max r mg/m^2
1	1	<0.96	0.000	0.077	<0.04	0.000	0.003
1	2	<0.96	0.000	0.077	<0.04	0.000	0.003
1	3	<0.96	0.000	0.077	<0.04	0.000	0.003
1	4	<0.96	0.000	0.077	<0.04	0.000	0.003
1	5	<0.96	0.000	0.077	<0.04	0.000	0.003
1	6	<0.96	0.000	0.077	<0.04	0.000	0.003
1	7	<0.96	0.000	0.077	<0.04	0.000	0.003
1	8	<0.96	0.000	0.077	<0.04	0.000	0.003
2	1	<0.96	0.000	0.076	<0.04	0.000	0.003
2	2	<0.96	0.000	0.076	<0.04	0.000	0.003
2	3	<0.96	0.000	0.076	<0.04	0.000	0.003
2	4	<0.96	0.000	0.076	<0.04	0.000	0.003
2	5	<0.96	0.000	0.076	<0.04	0.000	0.003
2	6	<0.96	0.000	0.076	<0.04	0.000	0.003
2	7	<0.96	0.000	0.076	<0.04	0.000	0.003
2	8	<0.96	0.000	0.076	<0.04	0.000	0.003
3	1	<0.96	0.000	0.077	<0.04	0.000	0.003
3	2	<0.96	0.000	0.077	<0.04	0.000	0.003
3	3	<0.96	0.000	0.077	<0.04	0.000	0.003
3	4	<0.96	0.000	0.077	<0.04	0.000	0.003
3	5	<0.96	0.000	0.077	<0.04	0.000	0.003
3	6	<0.96	0.000	0.077	<0.04	0.000	0.003
3	7	<0.96	0.000	0.077	<0.04	0.000	0.003
3	8	<0.96	0.000	0.077	<0.04	0.000	0.003
4	1	<0.96	0.000	0.077	<0.04	0.000	0.003
4	2	<0.96	0.000	0.077	<0.04	0.000	0.003
4	3	<0.96	0.000	0.077	<0.04	0.000	0.003
4	4	<0.96	0.000	0.077	<0.04	0.000	0.003
4	5	<0.96	0.000	0.077	<0.04	0.000	0.003
4	6	<0.96	0.000	0.077	<0.04	0.000	0.003
4	7	<0.96	0.000	0.077	<0.04	0.000	0.003
4	8	<0.96	0.000	0.077	<0.04	0.000	0.003
5	1	<0.96	0.000	0.077	<0.04	0.000	0.003
5	2	<0.96	0.000	0.077	<0.04	0.000	0.003
5	3	<0.96	0.000	0.077	<0.04	0.000	0.003
5	4	<0.96	0.000	0.077	<0.04	0.000	0.003
5	5	<0.96	0.000	0.077	<0.04	0.000	0.003
5	6	<0.96	0.000	0.077	<0.04	0.000	0.003
5	7	<0.96	0.000	0.077	<0.04	0.000	0.003
5	8	<0.96	0.000	0.077	<0.04	0.000	0.003
			∑ min	∑ max		∑ min	∑ max
1			0.000	0.614		0.000	0.026
2			0.000	0.605		0.000	0.026
3			0.000	0.615		0.000	0.026
4			0.000	0.615		0.000	0.026
5			0.000	0.615		0.000	0.026
Mean			0.000	0.613		0.000	0.026
SD			0.000	0.004		0.000	0.000

Table C. Continued Basalt.

Stone	Fraction	LAB 1 Sn µg/l	min r mg/m^2	max r mg/m^2	LAB 2 Sn µg/l	min r mg/m^2	max r mg/m^2
1	1	0.098	0.008	0.008	<0.02	0.000	0.002
1	2	0.111	0.009	0.009	<0.02	0.000	0.002
1	3	0.048	0.004	0.004	<0.02	0.000	0.002
1	4	0.043	0.003	0.003	<0.02	0.000	0.002
1	5	0.038	0.003	0.003	<0.02	0.000	0.002
1	6	0.053	0.004	0.004	<0.02	0.000	0.002
1	7	0.039	0.003	0.003	<0.02	0.000	0.002
1	8	0.030	0.002	0.002	<0.02	0.000	0.002
2	1	0.100	0.008	0.008	<0.02	0.000	0.002
2	2	0.177	0.014	0.014	<0.02	0.000	0.002
2	3	0.101	0.008	0.008	<0.02	0.000	0.002
2	4	0.242	0.019	0.019	<0.02	0.000	0.002
2	5	0.098	0.008	0.008	<0.02	0.000	0.002
2	6	0.137	0.011	0.011	<0.02	0.000	0.002
2	7	0.093	0.007	0.007	<0.02	0.000	0.002
2	8	0.252	0.020	0.020	<0.02	0.000	0.002
3	1	0.117	0.009	0.009	<0.02	0.000	0.002
3	2	0.112	0.009	0.009	<0.02	0.000	0.002
3	3	0.499	0.040	0.040	<0.02	0.000	0.002
3	4	0.122	0.010	0.010	<0.02	0.000	0.002
3	5	0.065	0.005	0.005	<0.02	0.000	0.002
3	6	0.068	0.005	0.005	<0.02	0.000	0.002
3	7	0.049	0.004	0.004	<0.02	0.000	0.002
3	8	0.047	0.004	0.004	<0.02	0.000	0.002
4	1	0.074	0.006	0.006	<0.02	0.000	0.002
4	2	0.140	0.011	0.011	<0.02	0.000	0.002
4	3	0.047	0.004	0.004	<0.02	0.000	0.002
4	4	0.054	0.004	0.004	<0.02	0.000	0.002
4	5	0.042	0.003	0.003	<0.02	0.000	0.002
4	6	0.056	0.004	0.004	<0.02	0.000	0.002
4	7	0.046	0.004	0.004	<0.02	0.000	0.002
4	8	0.037	0.003	0.003	<0.02	0.000	0.002
5	1	0.064	0.005	0.005	<0.02	0.000	0.002
5	2	0.132	0.011	0.011	<0.02	0.000	0.002
5	3	0.062	0.005	0.005	<0.02	0.000	0.002
5	4	0.105	0.008	0.008	<0.02	0.000	0.002
5	5	0.051	0.004	0.004	<0.02	0.000	0.002
5	6	0.066	0.005	0.005	<0.02	0.000	0.002
5	7	0.035	0.003	0.003	<0.02	0.000	0.002
5	8	0.041	0.003	0.003	<0.02	0.000	0.002
			∑ min	∑ max		∑ min	∑ max
1			0.037	0.037		0.000	0.013
2			0.096	0.096		0.000	0.013
3			0.086	0.086		0.000	0.013
4			0.040	0.040		0.000	0.013
5			0.044	0.044		0.000	0.013
Mean			0.061	0.061		0.000	0.013
SD			0.028	0.028		0.000	0.000

Table C. Continued Basalt.

Stone	Fraction	LAB 1 Sr µg/l	min r mg/m^2	max r mg/m^2	LAB 2 Sr µg/l	min r mg/m^2	max r mg/m^2
1	1	1.43	0.11	0.11	5.20	0.42	0.42
1	2	6.67	0.53	0.53	4.57	0.37	0.37
1	3	5.99	0.48	0.48	4.20	0.34	0.34
1	4	7.20	0.58	0.58	4.13	0.33	0.33
1	5	7.47	0.60	0.60	6.63	0.53	0.53
1	6	8.02	0.64	0.64	6.72	0.54	0.54
1	7	8.89	0.71	0.71	11.67	0.93	0.93
1	8	8.71	0.70	0.70	9.45	0.76	0.76
2	1	1.97	0.16	0.16	2.57	0.20	0.20
2	2	8.37	0.67	0.67	1.96	0.15	0.15
2	3	1.57	0.13	0.13	1.83	0.14	0.14
2	4	8.96	0.72	0.72	1.82	0.14	0.14
2	5	11.42	0.91	0.91	2.01	0.16	0.16
2	6	12.44	1.00	1.00	2.34	0.18	0.18
2	7	16.32	1.31	1.31	3.89	0.31	0.31
2	8	19.68	1.57	1.57	3.23	0.25	0.25
3	1	2.90	0.23	0.23	2.90	0.23	0.23
3	2	6.79	0.54	0.54	2.53	0.20	0.20
3	3	6.62	0.53	0.53	2.34	0.19	0.19
3	4	7.26	0.58	0.58	2.10	0.17	0.17
3	5	7.66	0.61	0.61	2.54	0.20	0.20
3	6	8.46	0.68	0.68	3.02	0.24	0.24
3	7	5.58	0.45	0.45	5.20	0.42	0.42
3	8	10.92	0.87	0.87	4.21	0.34	0.34
4	1	5.72	0.46	0.46	2.26	0.18	0.18
4	2	6.86	0.55	0.55	1.71	0.14	0.14
4	3	6.60	0.53	0.53	1.57	0.13	0.13
4	4	6.70	0.54	0.54	1.58	0.13	0.13
4	5	7.72	0.62	0.62	2.25	0.18	0.18
4	6	7.60	0.61	0.61	2.71	0.22	0.22
4	7	9.14	0.73	0.73	6.49	0.52	0.52
4	8	8.50	0.68	0.68	5.67	0.45	0.45
5	1	2.15	0.17	0.17	2.45	0.20	0.20
5	2	6.45	0.52	0.52	2.04	0.16	0.16
5	3	0.86	0.07	0.07	2.04	0.16	0.16
5	4	7.06	0.56	0.56	1.78	0.14	0.14
5	5	8.37	0.67	0.67	1.98	0.16	0.16
5	6	8.81	0.70	0.70	2.00	0.16	0.16
5	7	5.84	0.47	0.47	3.23	0.26	0.26
5	8	12.07	0.97	0.97	3.14	0.25	0.25
			∑ min	∑ max		∑ min	∑ max
1			4.35	4.35		4.20	4.20
2			6.46	6.46		1.55	1.55
3			4.49	4.49		1.99	1.99
4			4.71	4.71		1.94	1.94
5			4.13	4.13		1.49	1.49
Mean			4.83	4.83		2.23	2.23
SD			0.94	0.94		1.12	1.12

Table C. Continued Basalt.

Stone	Fraction	LAB 1 U µg/l	min r mg/m²	max r mg/m²	LAB 2 U µg/l	min r mg/m²	max r mg/m²
1	1	<0.04	0.0	0.003	<0.01	0.00	0.0008
1	2	<0.04	0.0	0.003	<0.01	0.00	0.0008
1	3	<0.04	0.0	0.003	<0.01	0.00	0.0008
1	4	<0.04	0.0	0.003	<0.01	0.00	0.0008
1	5	<0.04	0.0	0.003	<0.01	0.00	0.0008
1	6	<0.04	0.0	0.003	<0.01	0.00	0.0008
1	7	<0.04	0.0	0.003	<0.01	0.00	0.0008
1	8	<0.04	0.0	0.003	<0.01	0.00	0.0008
2	1	<0.04	0.0	0.003	<0.01	0.00	0.0008
2	2	<0.04	0.0	0.003	<0.01	0.00	0.0008
2	3	<0.04	0.0	0.003	<0.01	0.00	0.0008
2	4	<0.04	0.0	0.003	<0.01	0.00	0.0008
2	5	<0.04	0.0	0.003	<0.01	0.00	0.0008
2	6	<0.04	0.0	0.003	<0.01	0.00	0.0008
2	7	<0.04	0.0	0.003	<0.01	0.00	0.0008
2	8	<0.04	0.0	0.003	<0.01	0.00	0.0008
3	1	<0.04	0.0	0.003	<0.01	0.00	0.0008
3	2	<0.04	0.0	0.003	<0.01	0.00	0.0008
3	3	<0.04	0.0	0.003	<0.01	0.00	0.0008
3	4	<0.04	0.0	0.003	<0.01	0.00	0.0008
3	5	<0.04	0.0	0.003	<0.01	0.00	0.0008
3	6	<0.04	0.0	0.003	<0.01	0.00	0.0008
3	7	<0.04	0.0	0.003	<0.01	0.00	0.0008
3	8	<0.04	0.0	0.003	<0.01	0.00	0.0008
4	1	<0.04	0.0	0.003	<0.01	0.00	0.0008
4	2	<0.04	0.0	0.003	<0.01	0.00	0.0008
4	3	<0.04	0.0	0.003	<0.01	0.00	0.0008
4	4	<0.04	0.0	0.003	<0.01	0.00	0.0008
4	5	<0.04	0.0	0.003	<0.01	0.00	0.0008
4	6	<0.04	0.0	0.003	<0.01	0.00	0.0008
4	7	<0.04	0.0	0.003	<0.01	0.00	0.0008
4	8	<0.04	0.0	0.003	<0.01	0.00	0.0008
5	1	<0.04	0.0	0.003	<0.01	0.00	0.0008
5	2	<0.04	0.0	0.003	<0.01	0.00	0.0008
5	3	<0.04	0.0	0.003	<0.01	0.00	0.0008
5	4	<0.04	0.0	0.003	<0.01	0.00	0.0008
5	5	<0.04	0.0	0.003	<0.01	0.00	0.0008
5	6	<0.04	0.0	0.003	<0.01	0.00	0.0008
5	7	<0.04	0.0	0.003	<0.01	0.00	0.0008
5	8	<0.04	0.0	0.003	<0.01	0.00	0.0008
			∑ min	∑ max		∑ min	∑ max
1			0.000	0.026		0.00	0.01
2			0.000	0.026		0.00	0.01
3			0.000	0.026		0.00	0.01
4			0.000	0.026		0.00	0.01
5			0.000	0.026		0.000	0.006
Mean			0.000	0.026		0.000	0.006
SD			0.000	0.000		0.000	0.000

Table C. Continued Basalt.

Stone	Fraction	LAB 1 V µg/l	min r mg/m²	max r mg/m²	LAB 2 V µg/l	min r mg/m²	max r mg/m²
1	1	0.66	0.05	0.05	0.43	0.03	0.03
1	2	0.60	0.05	0.05	0.32	0.03	0.03
1	3	0.33	0.03	0.03	0.28	0.02	0.02
1	4	0.31	0.02	0.02	0.24	0.02	0.02
1	5	0.57	0.05	0.05	0.39	0.03	0.03
1	6	0.70	0.06	0.06	0.38	0.03	0.03
1	7	0.61	0.05	0.05	0.15	0.01	0.01
1	8	0.92	0.07	0.07	<0.02	0.00	0.00
2	1	0.29	0.02	0.02	0.22	0.02	0.02
2	2	0.22	0.02	0.02	0.12	0.01	0.01
2	3	0.14	0.01	0.01	<0.02	0.00	0.00
2	4	0.15	0.01	0.01	<0.02	0.00	0.00
2	5	0.26	0.02	0.02	0.10	0.01	0.01
2	6	0.35	0.03	0.03	<0.02	0.00	0.00
2	7	0.37	0.03	0.03	0.13	0.01	0.01
2	8	0.62	0.05	0.05	<0.02	0.00	0.00
3	1	0.59	0.05	0.05	0.77	0.06	0.06
3	2	0.48	0.04	0.04	0.13	0.01	0.01
3	3	0.32	0.03	0.03	<0.02	0.00	0.00
3	4	0.30	0.02	0.02	<0.02	0.00	0.00
3	5	0.62	0.05	0.05	0.06	0.01	0.01
3	6	0.76	0.06	0.06	0.21	0.02	0.02
3	7	0.82	0.07	0.07	0.27	0.02	0.02
3	8	1.50	0.12	0.12	0.14	0.01	0.01
4	1	1.05	0.08	0.08	<0.02	0.00	0.00
4	2	0.50	0.04	0.04	<0.02	0.00	0.00
4	3	0.26	0.02	0.02	<0.02	0.00	0.00
4	4	0.21	0.02	0.02	<0.02	0.00	0.00
4	5	0.36	0.03	0.03	<0.02	0.00	0.00
4	6	0.42	0.03	0.03	<0.02	0.00	0.00
4	7	0.37	0.03	0.03	0.25	0.02	0.02
4	8	0.59	0.05	0.05	0.21	0.02	0.02
5	1	1.21	0.10	0.10	0.85	0.07	0.07
5	2	0.98	0.08	0.08	0.43	0.03	0.03
5	3	0.51	0.04	0.04	0.31	0.02	0.02
5	4	0.43	0.03	0.03	0.22	0.02	0.02
5	5	0.85	0.07	0.07	0.62	0.05	0.05
5	6	1.09	0.09	0.09	0.57	0.05	0.05
5	7	0.99	0.08	0.08	0.58	0.05	0.05
5	8	1.88	0.15	0.15	0.41	0.03	0.03
			∑ min	∑ max		∑ min	∑ max
1			0.38	0.38		0.17	0.18
2			0.19	0.19		0.04	0.05
3			0.43	0.43		0.13	0.13
4			0.30	0.30		0.04	0.05
5			0.64	0.64		0.32	0.32
Mean			0.39	0.39		0.14	0.14
SD			0.17	0.17		0.12	0.11

Table C. Continued Basalt.

Stone	Fraction	LAB 1 Zn µg/l	min r mg/m^2	max r mg/m^2	LAB 2 Zn µg/l	min r mg/m^2	max r mg/m^2
1	1	0.4	0.0	0.0	2.98	0.24	0.24
1	2	296.7	23.7	23.7	2.24	0.18	0.18
1	3	406.8	32.5	32.5	1.40	0.11	0.11
1	4	304.5	24.4	24.4	1.24	0.10	0.10
1	5	305.5	24.4	24.4	0.98	0.08	0.08
1	6	289.3	23.1	23.1	0.64	0.05	0.05
1	7	376.6	30.1	30.1	<0.12	0.00	0.01
1	8	322.4	25.8	25.8	<0.12	0.00	0.01
2	1	1.9	0.1	0.1	<0.12	0.00	0.01
2	2	295.9	23.7	23.7	<0.12	0.00	0.01
2	3	101.5	8.1	8.1	<0.12	0.00	0.01
2	4	389.4	31.2	31.2	<0.12	0.00	0.01
2	5	371.3	29.7	29.7	<0.12	0.00	0.01
2	6	471.8	37.7	37.7	<0.12	0.00	0.01
2	7	445.9	35.7	35.7	<0.12	0.00	0.01
2	8	418.7	33.5	33.5	<0.12	0.00	0.01
3	1	5.0	0.4	0.4	0.26	0.02	0.02
3	2	281.3	22.5	22.5	5.03	0.40	0.40
3	3	297.5	23.8	23.8	<0.12	0.00	0.01
3	4	329.6	26.4	26.4	<0.12	0.00	0.01
3	5	331.2	26.5	26.5	<0.12	0.00	0.01
3	6	455.2	36.4	36.4	<0.12	0.00	0.01
3	7	225.0	18.0	18.0	<0.12	0.00	0.01
3	8	369.7	29.6	29.6	<0.12	0.00	0.01
4	1	0.6	0.0	0.0	0.50	0.04	0.04
4	2	455.0	36.4	36.4	0.24	0.02	0.02
4	3	448.5	35.9	35.9	0.13	0.01	0.01
4	4	417.4	33.4	33.4	<0.12	0.00	0.01
4	5	328.0	26.2	26.2	<0.12	0.00	0.01
4	6	433.9	34.7	34.7	<0.12	0.00	0.01
4	7	484.7	38.8	38.8	<0.12	0.00	0.01
4	8	327.7	26.2	26.2	<0.12	0.00	0.01
5	1	0.5	0.0	0.0	0.36	0.03	0.03
5	2	452.5	36.2	36.2	0.15	0.01	0.01
5	3	33.3	2.7	2.7	3.26	0.26	0.26
5	4	278.9	22.3	22.3	<0.12	0.00	0.01
5	5	331.3	26.5	26.5	0.47	0.04	0.04
5	6	298.1	23.8	23.8	0.97	0.08	0.08
5	7	146.1	11.7	11.7	0.83	0.07	0.07
5	8	506.2	40.5	40.5	0.41	0.03	0.03
			∑ min	∑ max		∑ min	∑ max
1			184.2	184.2		0.76	0.78
2			199.7	199.7		0.00	0.08
3			183.6	183.6		0.42	0.48
4			231.6	231.6		0.07	0.12
5			163.7	163.7		0.52	0.53
Mean			192.6	192.6		0.35	0.40
SD			25.3	25.3		0.32	0.30

Table D. DSLT results Greywacke (LAB 1 DSLT contract laboratory, LAB 2 DSLT BfG).

Stone	Fraction	LAB 1 Al µg/l	min r mg/m²	max r mg/m²	LAB 2 Al µg/l	min r mg/m²	max r mg/m²
1	1	41.1	3.3	3.3	<50	0.0	4.0
1	2	<40.8	0.0	3.3	<50	0.0	4.0
1	3	<40.8	0.0	3.3	<50	0.0	4.0
1	4	<40.8	0.0	3.3	<50	0.0	4.0
1	5	<40.8	0.0	3.3	<50	0.0	4.0
1	6	<40.8	0.0	3.3	<50	0.0	4.0
1	7	<40.8	0.0	3.3	<50	0.0	4.0
1	8	76	6.1	6.1	<50	0.0	4.0
2	1	<40.8	0.0	3.3	<50	0.0	4.0
2	2	<40.8	0.0	3.3	<50	0.0	4.0
2	3	<40.8	0.0	3.3	<50	0.0	4.0
2	4	<40.8	0.0	3.3	<50	0.0	4.0
2	5	<40.8	0.0	3.3	<50	0.0	4.0
2	6	<40.8	0.0	3.3	<50	0.0	4.0
2	7	<40.8	0.0	3.3	<50	0.0	4.0
2	8	327	26.2	26.2	<50	0.0	4.0
3	1	<40.8	0.0	3.3	<50	0.0	4.0
3	2	<40.8	0.0	3.3	<50	0.0	4.0
3	3	<40.8	0.0	3.3	<50	0.0	4.0
3	4	<40.8	0.0	3.3	<50	0.0	4.0
3	5	<40.8	0.0	3.3	<50	0.0	4.0
3	6	<40.8	0.0	3.3	<50	0.0	4.0
3	7	<40.8	0.0	3.3	<50	0.0	4.0
3	8	269	21.5	21.5	<50	0.0	4.0
4	1	<40.8	0.0	3.3	<50	0.0	4.0
4	2	<40.8	0.0	3.3	<50	0.0	4.0
4	3	<40.8	0.0	3.3	<50	0.0	4.0
4	4	<40.8	0.0	3.3	<50	0.0	4.0
4	5	<40.8	0.0	3.3	<50	0.0	4.0
4	6	<40.8	0.0	3.3	<50	0.0	4.0
4	7	<40.8	0.0	3.3	<50	0.0	4.0
4	8	<40.8	0.0	3.3	<50	0.0	4.0
5	1	<40.8	0.0	3.3	<50	0.0	4.0
5	2	<40.8	0.0	3.3	<50	0.0	4.0
5	3	<40.8	0.0	3.3	<50	0.0	4.0
5	4	<40.8	0.0	3.3	<50	0.0	4.0
5	5	<40.8	0.0	3.3	<50	0.0	4.0
5	6	<40.8	0.0	3.3	<50	0.0	4.0
5	7	<40.8	0.0	3.3	<50	0.0	4.0
5	8	<40.8	0.0	3.3	<50	0.0	4.0
			∑ min	∑ max		∑ min	∑ max
1			9.4	28.9		0.0	32.0
2			26.2	49.0		0.0	32.0
3			21.5	44.3		0.0	32.0
4			0.0	26.1		0.0	32.0
5			0.0	26.1		0.0	32.0
Mean			11.4	34.9		0.0	32.0
SD			12.1	10.9		0.0	0.0

Table D. Continued Greywacke.

Stone	Fraction	LAB 1 As µg/l	min r mg/m²	max r mg/m²	LAB 2 As µg/l	min r mg/m²	max r mg/m²
1	1	0.35	0.028	0.028	0.10	0.01	0.01
1	2	0.25	0.020	0.020	0.03	0.00	0.00
1	3	0.24	0.019	0.019	0.03	0.00	0.00
1	4	0.21	0.017	0.017	0.03	0.00	0.00
1	5	0.41	0.033	0.033	0.04	0.00	0.00
1	6	0.27	0.022	0.022	0.03	0.00	0.00
1	7	0.23	0.018	0.018	0.03	0.00	0.00
1	8	0.27	0.022	0.022	0.03	0.00	0.00
2	1	0.10	0.008	0.008	0.39	0.03	0.03
2	2	0.05	0.004	0.004	0.16	0.01	0.01
2	3	0.04	0.003	0.003	0.13	0.01	0.01
2	4	0.04	0.003	0.003	0.13	0.01	0.01
2	5	0.04	0.003	0.003	0.20	0.02	0.02
2	6	0.04	0.003	0.003	0.16	0.01	0.01
2	7	0.04	0.003	0.003	0.16	0.01	0.01
2	8	0.09	0.007	0.007	0.12	0.01	0.01
3	1	0.11	0.009	0.009	0.23	0.02	0.02
3	2	0.17	0.014	0.014	0.17	0.01	0.01
3	3	0.08	0.007	0.007	0.12	0.01	0.01
3	4	0.09	0.008	0.008	0.14	0.01	0.01
3	5	0.19	0.015	0.015	0.22	0.02	0.02
3	6	0.17	0.014	0.014	0.23	0.02	0.02
3	7	0.22	0.018	0.018	0.18	0.01	0.01
3	8	0.20	0.016	0.016	0.14	0.01	0.01
4	1	0.08	0.006	0.006	0.29	0.02	0.02
4	2	0.10	0.008	0.008	0.26	0.02	0.02
4	3	0.10	0.008	0.008	0.19	0.01	0.01
4	4	0.11	0.008	0.008	0.16	0.01	0.01
4	5	0.23	0.018	0.018	0.19	0.02	0.02
4	6	0.19	0.015	0.015	0.12	0.01	0.01
4	7	0.14	0.011	0.011	0.09	0.01	0.01
4	8	0.14	0.011	0.011	0.05	0.00	0.00
5	1	0.12	0.010	0.010	0.10	0.01	0.01
5	2	0.11	0.009	0.009	0.03	0.002	0.002
5	3	0.04	0.003	0.003	0.05	0.004	0.004
5	4	0.05	0.004	0.004	0.04	0.003	0.003
5	5	0.05	0.004	0.004	0.05	0.004	0.004
5	6	0.04	0.003	0.003	0.05	0.004	0.004
5	7	0.03	0.003	0.003	0.04	0.003	0.003
5	8	0.03	0.003	0.003	0.05	0.004	0.004
			∑ min	∑ max		∑ min	∑ max
1			0.18	0.18		0.026	0.026
2			0.04	0.04		0.116	0.116
3			0.10	0.10		0.113	0.113
4			0.09	0.09		0.107	0.107
5			0.04	0.04		0.032	0.032
Mean			0.088	0.088		0.08	0.08
SD			0.059	0.059		0.05	0.05

Table D. Continued Greywacke.

Stone	Fraction	LAB 1 Ba µg/l	min r mg/m^2	max r mg/m^2	LAB 2 Ba µg/l	min r mg/m^2	max r mg/m^2
1	1	514	41.10	41.10	8.44	0.00	0.68
1	2	508	40.68	40.68	8.27	0.00	0.66
1	3	485	38.77	38.77	3.64	0.29	0.29
1	4	530	42.37	42.37	2.56	0.00	0.21
1	5	550	43.97	43.97	2.78	0.22	0.22
1	6	466	37.31	37.31	2.63	0.21	0.21
1	7	533	42.63	42.63	2.55	0.00	0.20
1	8	274	21.92	21.92	1.98	0.16	0.16
2	1	484	38.69	38.69	15.8	0.00	1.27
2	2	454	36.29	36.29	15.5	0.00	1.24
2	3	388	31.05	31.05	4.33	0.35	0.35
2	4	491	39.26	39.26	4.17	0.33	0.33
2	5	435	34.81	34.81	5.28	0.42	0.42
2	6	440	35.22	35.22	5.73	0.46	0.46
2	7	466	37.29	37.29	7.22	0.58	0.58
2	8	878	70.23	70.23	6.79	0.54	0.54
3	1	548	43.81	43.81	15.7	1.26	1.26
3	2	474	37.90	37.90	7.76	0.62	0.62
3	3	57	4.60	4.60	4.06	0.33	0.33
3	4	463	37.04	37.04	4.26	0.34	0.34
3	5	498	39.84	39.84	7.01	0.56	0.56
3	6	535	42.81	42.81	9.00	0.72	0.72
3	7	561	44.88	44.88	14.9	1.19	1.19
3	8	911	72.84	72.84	19.7	1.58	1.58
4	1	546	43.71	43.71	2.99	0.24	0.24
4	2	509	40.71	40.71	1.97	0.00	0.16
4	3	461	36.86	36.86	1.06	0.00	0.08
4	4	491	39.30	39.30	1.01	0.08	0.08
4	5	484	38.73	38.73	1.65	0.13	0.13
4	6	613	49.00	49.00	1.92	0.15	0.15
4	7	579	46.28	46.28	2.55	0.20	0.20
4	8	322	25.77	25.77	2.17	0.17	0.17
5	1	546	43.70	43.70	22.5	1.80	1.80
5	2	531	42.50	42.50	15.7	1.25	1.25
5	3	87	6.99	6.99	7.86	0.63	0.63
5	4	477	38.13	38.13	6.11	0.49	0.49
5	5	505	40.43	40.43	7.34	0.59	0.59
5	6	251	20.05	20.05	5.97	0.48	0.48
5	7	603	48.27	48.27	5.77	0.46	0.46
5	8	641	51.29	51.29	4.69	0.38	0.38
			∑ min	∑ max		∑ min	∑ max
1			308.7	308.7		0.88	2.63
2			322.9	322.9		2.68	5.19
3			323.7	323.7		6.60	6.60
4			320.4	320.4		0.98	1.23
5			291.4	291.4		6.07	6.07
Mean			313.4	313.4		3.44	4.34
SD			13.7	13.7		2.74	2.32

Table D. Continued Greywacke.

Stone	Fraction	LAB 1 Cd µg/l	min r mg/m²	max r mg/m²	LAB 2 Cd µg/l	min r mg/m²	max r mg/m²
1	1	0.037	0.003	0.003	<0.01	0.000	0.001
1	2	0.034	0.003	0.003	<0.01	0.000	0.001
1	3	0.033	0.003	0.003	<0.01	0.000	0.001
1	4	0.032	0.003	0.003	<0.01	0.000	0.001
1	5	0.039	0.003	0.003	0.010	0.001	0.001
1	6	0.040	0.003	0.003	<0.01	0.000	0.001
1	7	0.041	0.003	0.003	0.015	0.001	0.001
1	8	0.027	0.002	0.002	0.010	0.001	0.001
2	1	0.032	0.003	0.003	<0.01	0.000	0.001
2	2	0.032	0.003	0.003	<0.01	0.000	0.001
2	3	0.028	0.002	0.002	<0.01	0.000	0.001
2	4	0.024	0.002	0.002	<0.01	0.000	0.001
2	5	0.028	0.002	0.002	<0.01	0.000	0.001
2	6	0.023	0.002	0.002	<0.01	0.000	0.001
2	7	0.030	0.002	0.002	<0.01	0.000	0.001
2	8	0.027	0.002	0.002	<0.01	0.000	0.001
3	1	0.039	0.003	0.003	<0.01	0.000	0.001
3	2	0.079	0.006	0.006	<0.01	0.000	0.001
3	3	0.005	0.000	0.000	<0.01	0.000	0.001
3	4	0.031	0.002	0.002	<0.01	0.000	0.001
3	5	0.032	0.003	0.003	<0.01	0.000	0.001
3	6	0.039	0.003	0.003	<0.01	0.000	0.001
3	7	0.035	0.003	0.003	<0.01	0.000	0.001
3	8	0.035	0.003	0.003	<0.01	0.000	0.001
4	1	0.032	0.003	0.003	<0.01	0.000	0.001
4	2	0.034	0.003	0.003	<0.01	0.000	0.001
4	3	0.028	0.002	0.002	<0.01	0.000	0.001
4	4	0.036	0.003	0.003	<0.01	0.000	0.001
4	5	0.040	0.003	0.003	<0.01	0.000	0.001
4	6	0.055	0.004	0.004	0.009	0.001	0.001
4	7	0.048	0.004	0.004	<0.01	0.000	0.001
4	8	0.028	0.002	0.002	<0.01	0.000	0.001
5	1	0.031	0.003	0.003	<0.01	0.000	0.001
5	2	0.027	0.002	0.002	<0.01	0.000	0.001
5	3	<0.01	0.000	0.001	<0.01	0.000	0.001
5	4	0.029	0.002	0.002	<0.01	0.000	0.001
5	5	0.039	0.003	0.003	<0.01	0.000	0.001
5	6	0.030	0.002	0.002	<0.01	0.000	0.001
5	7	0.035	0.003	0.003	<0.01	0.000	0.001
5	8	0.034	0.003	0.003	<0.01	0.000	0.001
			∑ min	∑ max		∑ min	∑ max
1			0.023	0.023		0.003	0.01
2			0.018	0.018		0.000	0.01
3			0.023	0.023		0.000	0.01
4			0.024	0.024		0.001	0.01
5			0.018	0.019		0.000	0.01
Mean			0.021	0.021		0.001	0.01
SD			0.003	0.003		0.001	0.00

Table D. Continued Greywacke.

Stone	Fraction	LAB 1 Co µg/l	min r mg/m^2	max r mg/m^2	LAB 2 Co µg/l	min r mg/m^2	max r mg/m^2
1	1	23.37	1.870	1.870	0.106	0.008	0.008
1	2	10.38	0.831	0.831	0.195	0.016	0.016
1	3	9.07	0.726	0.726	0.259	0.021	0.021
1	4	10.05	0.804	0.804	0.227	0.018	0.018
1	5	13.99	1.119	1.119	0.329	0.026	0.026
1	6	10.28	0.822	0.822	0.461	0.037	0.037
1	7	10.41	0.833	0.833	0.665	0.053	0.053
1	8	8.24	0.659	0.659	0.579	0.046	0.046
2	1	0.11	0.009	0.009	0.032	0.003	0.003
2	2	0.09	0.007	0.007	0.059	0.005	0.005
2	3	0.08	0.006	0.006	0.069	0.006	0.006
2	4	0.08	0.006	0.006	0.069	0.006	0.006
2	5	0.09	0.007	0.007	0.100	0.008	0.008
2	6	0.08	0.006	0.006	0.094	0.008	0.008
2	7	0.08	0.006	0.006	0.157	0.013	0.013
2	8	0.11	0.009	0.009	0.137	0.011	0.011
3	1	0.10	0.008	0.008	0.039	0.003	0.003
3	2	0.15	0.012	0.012	0.039	0.003	0.003
3	3	0.03	0.002	0.002	0.033	0.003	0.003
3	4	0.08	0.007	0.007	0.027	0.002	0.002
3	5	0.10	0.008	0.008	0.038	0.003	0.003
3	6	0.11	0.008	0.008	0.038	0.003	0.003
3	7	0.09	0.008	0.008	0.074	0.006	0.006
3	8	0.14	0.011	0.011	0.082	0.007	0.007
4	1	1.82	0.145	0.145	0.058	0.005	0.005
4	2	1.42	0.113	0.113	0.098	0.008	0.008
4	3	1.46	0.117	0.117	0.078	0.006	0.006
4	4	1.33	0.107	0.107	0.130	0.010	0.010
4	5	1.82	0.146	0.146	0.222	0.018	0.018
4	6	1.02	0.081	0.081	0.251	0.020	0.020
4	7	0.76	0.061	0.061	0.290	0.023	0.023
4	8	0.67	0.053	0.053	0.218	0.017	0.017
5	1	0.17	0.013	0.013	0.013	0.001	0.001
5	2	0.11	0.009	0.009	0.021	0.002	0.002
5	3	0.02	0.002	0.002	0.017	0.001	0.001
5	4	0.08	0.006	0.006	0.012	0.001	0.001
5	5	0.12	0.009	0.009	<0.01	0.000	0.001
5	6	0.09	0.008	0.008	<0.01	0.000	0.001
5	7	0.09	0.007	0.007	<0.01	0.000	0.001
5	8	0.15	0.012	0.012	<0.01	0.000	0.001
			∑ min	∑ max		∑ min	∑ max
1			7.664	7.664		0.226	0.226
2			0.057	0.057		0.057	0.057
3			0.065	0.065		0.030	0.030
4			0.823	0.823		0.107	0.107
5			0.066	0.066		0.005	0.008
Mean			1.735	1.735		0.085	0.086
SD			3.331	3.331		0.087	0.087

Table D. Continued Greywacke.

Stone	Fraction	LAB 1 Cr µg/l	min r mg/m²	max r mg/m²	LAB 2 Cr µg/l	min r mg/m²	max r mg/m²
1	1	0.188	0.015	0.015	0.041	0.003	0.003
1	2	0.087	0.007	0.007	0.019	0.002	0.002
1	3	0.080	0.006	0.006	0.013	0.001	0.001
1	4	0.089	0.007	0.007	0.011	0.001	0.001
1	5	0.093	0.007	0.007	0.012	0.001	0.001
1	6	0.078	0.006	0.006	0.023	0.002	0.002
1	7	0.066	0.005	0.005	0.014	0.001	0.001
1	8	0.067	0.005	0.005	0.027	0.002	0.002
2	1	0.087	0.007	0.007	0.025	0.002	0.002
2	2	0.075	0.006	0.006	0.021	0.002	0.002
2	3	0.075	0.006	0.006	0.008	0.001	0.001
2	4	0.074	0.006	0.006	0.008	0.001	0.001
2	5	0.072	0.006	0.006	0.012	0.001	0.001
2	6	0.075	0.006	0.006	0.013	0.001	0.001
2	7	0.080	0.006	0.006	0.011	0.001	0.001
2	8	0.308	0.025	0.025	0.028	0.002	0.002
3	1	0.219	0.017	0.017	0.034	0.003	0.003
3	2	0.138	0.011	0.011	0.013	0.001	0.001
3	3	0.018	0.001	0.001	0.010	0.001	0.001
3	4	0.089	0.007	0.007	<0.01	0.000	0.001
3	5	0.107	0.009	0.009	0.011	0.001	0.001
3	6	0.167	0.013	0.013	0.012	0.001	0.001
3	7	0.080	0.006	0.006	0.016	0.001	0.001
3	8	0.262	0.021	0.021	0.040	0.003	0.003
4	1	0.107	0.009	0.009	0.075	0.006	0.006
4	2	0.102	0.008	0.008	0.021	0.002	0.002
4	3	0.072	0.006	0.006	0.022	0.002	0.002
4	4	0.065	0.005	0.005	0.010	0.001	0.001
4	5	0.057	0.005	0.005	0.013	0.001	0.001
4	6	0.084	0.007	0.007	0.017	0.001	0.001
4	7	0.064	0.005	0.005	0.012	0.001	0.001
4	8	<0.06	0.000	0.005	0.030	0.002	0.002
5	1	0.102	0.008	0.008	0.363	0.029	0.029
5	2	0.113	0.009	0.009	0.024	0.002	0.002
5	3	0.013	0.001	0.001	<0.01	0.000	0.001
5	4	0.068	0.005	0.005	<0.01	0.000	0.001
5	5	0.104	0.008	0.008	0.010	0.001	0.001
5	6	0.271	0.022	0.022	0.016	0.001	0.001
5	7	0.077	0.006	0.006	0.022	0.002	0.002
5	8	0.060	0.005	0.005	0.041	0.003	0.003
			∑ min	∑ max		∑ min	∑ max
1			0.060	0.060		0.013	0.013
2			0.068	0.068		0.010	0.010
3			0.086	0.086		0.011	0.012
4			0.044	0.049		0.016	0.016
5			0.065	0.065		0.038	0.040
Mean			0.064	0.065		0.018	0.018
SD			0.015	0.014		0.012	0.012

Table D. Continued Greywacke.

Stone	Fraction	LAB 1 Cu µg/l	min r mg/m²	max r mg/m²	LAB 2 Cu µg/l	min r mg/m²	max r mg/m²
1	1	7.232	0.579	0.579	0.040	0.003	0.003
1	2	2.658	0.213	0.213	0.079	0.006	0.006
1	3	2.581	0.206	0.206	0.032	0.003	0.003
1	4	3.858	0.309	0.309	0.034	0.003	0.003
1	5	4.050	0.324	0.324	0.047	0.004	0.004
1	6	3.243	0.259	0.259	0.037	0.003	0.003
1	7	2.870	0.230	0.230	0.039	0.003	0.003
1	8	1.609	0.129	0.129	0.053	0.004	0.004
2	1	3.305	0.264	0.264	0.404	0.032	0.032
2	2	2.856	0.228	0.228	0.028	0.002	0.002
2	3	2.014	0.161	0.161	<0.02	0.000	0.002
2	4	1.651	0.132	0.132	<0.02	0.000	0.002
2	5	1.948	0.156	0.156	0.028	0.002	0.002
2	6	1.792	0.143	0.143	0.038	0.003	0.003
2	7	1.713	0.137	0.137	0.032	0.003	0.003
2	8	1.067	0.085	0.085	0.053	0.004	0.004
3	1	6.733	0.539	0.539	0.059	0.005	0.005
3	2	2.569	0.206	0.206	0.021	0.002	0.002
3	3	1.000	0.080	0.080	<0.02	0.000	0.002
3	4	3.858	0.309	0.309	<0.02	0.000	0.002
3	5	3.892	0.311	0.311	0.023	0.002	0.002
3	6	4.507	0.361	0.361	0.024	0.002	0.002
3	7	2.835	0.227	0.227	0.048	0.004	0.004
3	8	2.851	0.228	0.228	0.047	0.004	0.004
4	1	5.534	0.443	0.443	0.039	0.003	0.003
4	2	3.817	0.305	0.305	0.027	0.002	0.002
4	3	2.274	0.182	0.182	<0.02	0.000	0.002
4	4	2.659	0.213	0.213	0.021	0.002	0.002
4	5	2.576	0.206	0.206	0.023	0.002	0.002
4	6	3.194	0.256	0.256	0.027	0.002	0.002
4	7	2.641	0.211	0.211	0.054	0.004	0.004
4	8	1.805	0.144	0.144	0.042	0.003	0.003
5	1	5.567	0.445	0.445	0.100	0.008	0.008
5	2	2.196	0.176	0.176	0.024	0.002	0.002
5	3	0.870	0.070	0.070	<0.02	0.002	0.002
5	4	3.500	0.280	0.280	0.030	0.002	0.002
5	5	4.227	0.338	0.338	0.039	0.003	0.003
5	6	3.319	0.266	0.266	0.051	0.004	0.004
5	7	3.132	0.251	0.251	0.039	0.003	0.003
5	8	2.269	0.182	0.182	0.025	0.002	0.002
			∑ min	∑ max		∑ min	∑ max
1			2.248	2.248		0.029	0.029
2			1.308	1.308		0.047	0.050
3			2.260	2.260		0.018	0.021
4			1.960	1.960		0.019	0.020
5			2.006	2.006		0.026	0.026
Mean			1.956	1.956		0.028	0.029
SD			0.387	0.387		0.012	0.012

Table D. Continued Greywacke.

Stone	Fraction	LAB 1 Mn µg/l	min r mg/m²	max r mg/m²	LAB 2 Mn µg/l	min r mg/m²	max r mg/m²
1	1	25.07	2.01	2.01	12.91	1.03	1.03
1	2	12.78	1.02	1.02	13.06	1.05	1.05
1	3	9.76	0.78	0.78	10.88	0.87	0.87
1	4	13.07	1.05	1.05	10.84	0.87	0.87
1	5	32.63	2.61	2.61	17.05	1.37	1.37
1	6	32.19	2.58	2.58	19.84	1.59	1.59
1	7	56.39	4.51	4.51	29.93	2.40	2.40
1	8	69.93	5.59	5.59	27.53	2.20	2.20
2	1	4.30	0.34	0.34	11.32	0.91	0.91
2	2	1.69	0.13	0.13	15.61	1.25	1.25
2	3	0.82	0.07	0.07	8.42	0.67	0.67
2	4	0.77	0.06	0.06	7.25	0.58	0.58
2	5	1.12	0.09	0.09	8.06	0.64	0.64
2	6	1.45	0.12	0.12	6.81	0.54	0.54
2	7	1.04	0.08	0.08	8.52	0.68	0.68
2	8	2.87	0.23	0.23	7.37	0.59	0.59
3	1	8.73	0.70	0.70	12.62	1.01	1.01
3	2	6.26	0.50	0.50	11.94	0.96	0.96
3	3	6.98	0.56	0.56	6.55	0.52	0.52
3	4	5.56	0.44	0.44	5.31	0.43	0.43
3	5	9.61	0.77	0.77	4.94	0.40	0.40
3	6	10.25	0.82	0.82	3.19	0.26	0.26
3	7	11.38	0.91	0.91	3.86	0.31	0.31
3	8	18.74	1.50	1.50	4.65	0.37	0.37
4	1	23.25	1.86	1.86	9.98	0.80	0.80
4	2	19.40	1.55	1.55	8.85	0.71	0.71
4	3	22.43	1.79	1.79	6.59	0.53	0.53
4	4	21.05	1.68	1.68	9.72	0.78	0.78
4	5	40.67	3.25	3.25	14.88	1.19	1.19
4	6	35.64	2.85	2.85	13.95	1.12	1.12
4	7	42.65	3.41	3.41	13.34	1.07	1.07
4	8	38.09	3.05	3.05	10.94	0.87	0.87
5	1	17.35	1.39	1.39	11.83	0.95	0.95
5	2	12.24	0.98	0.98	13.51	1.08	1.08
5	3	9.22	0.74	0.74	6.87	0.55	0.55
5	4	5.82	0.47	0.47	4.15	0.33	0.33
5	5	7.79	0.62	0.62	1.85	0.15	0.15
5	6	11.16	0.89	0.89	0.61	0.05	0.05
5	7	17.85	1.43	1.43	<0.46	0.00	0.04
5	8	38.13	3.05	3.05	<0.46	0.00	0.04
			∑ min	∑ max		∑ min	∑ max
1			20.15	20.15		11.37	11.37
2			1.12	1.12		5.87	5.87
3			6.20	6.20		4.25	4.25
4			19.45	19.45		7.06	7.06
5			9.56	9.56		3.11	3.18
Mean			11.30	11.30		6.33	6.35
SD			8.33	8.33		3.20	3.18

Table D. Continued Greywacke.

Stone	Fraction	LAB 1 Mo µg/l	min r mg/m²	max r mg/m²	LAB 2 Mo µg/l	min r mg/m²	max r mg/m²
1	1	0.159	0.013	0.013	0.879	0.070	0.070
1	2	0.081	0.007	0.007	0.424	0.034	0.034
1	3	0.056	0.004	0.004	0.331	0.027	0.027
1	4	<0.06	0.000	0.005	0.282	0.023	0.023
1	5	<0.06	0.000	0.005	0.661	0.053	0.053
1	6	<0.06	0.000	0.005	0.730	0.059	0.059
1	7	<0.06	0.000	0.005	1.403	0.112	0.112
1	8	<0.06	0.000	0.005	1.515	0.121	0.121
2	1	0.637	0.051	0.051	0.275	0.022	0.022
2	2	0.203	0.016	0.016	0.137	0.011	0.011
2	3	0.096	0.008	0.008	0.076	0.006	0.006
2	4	<0.06	0.000	0.005	0.100	0.008	0.008
2	5	<0.06	0.000	0.005	0.152	0.012	0.012
2	6	<0.06	0.000	0.005	0.184	0.015	0.015
2	7	<0.06	0.000	0.005	0.282	0.023	0.023
2	8	0.057	0.005	0.005	0.296	0.024	0.024
3	1	0.124	0.010	0.010	0.987	0.079	0.079
3	2	0.115	0.009	0.009	0.333	0.027	0.027
3	3	<0.06	0.000	0.005	0.301	0.024	0.024
3	4	<0.06	0.000	0.005	0.340	0.027	0.027
3	5	<0.06	0.000	0.005	0.554	0.044	0.044
3	6	<0.06	0.000	0.005	0.602	0.048	0.048
3	7	<0.06	0.000	0.005	1.005	0.080	0.080
3	8	0.066	0.005	0.005	0.908	0.073	0.073
4	1	0.277	0.022	0.022	0.200	0.016	0.016
4	2	0.262	0.021	0.021	0.092	0.007	0.007
4	3	0.278	0.022	0.022	0.042	0.003	0.003
4	4	0.294	0.024	0.024	0.028	0.002	0.002
4	5	0.611	0.049	0.049	0.064	0.005	0.005
4	6	0.533	0.043	0.043	0.070	0.006	0.006
4	7	0.894	0.072	0.072	0.135	0.011	0.011
4	8	1.066	0.085	0.085	0.111	0.009	0.009
5	1	0.289	0.023	0.023	0.635	0.051	0.051
5	2	<0.06	0.000	0.005	0.272	0.022	0.022
5	3	<0.06	0.000	0.005	0.168	0.013	0.013
5	4	<0.06	0.000	0.005	0.154	0.012	0.012
5	5	0.056	0.004	0.004	0.184	0.015	0.015
5	6	<0.06	0.000	0.005	0.251	0.020	0.020
5	7	<0.06	0.000	0.005	0.341	0.027	0.027
5	8	0.058	0.005	0.005	0.386	0.031	0.031
			∑ min	∑ max		∑ min	∑ max
1			0.024	0.048		0.499	0.499
2			0.079	0.099		0.120	0.120
3			0.024	0.048		0.403	0.403
4			0.337	0.337		0.059	0.059
5			0.032	0.056		0.191	0.191
Mean			0.099	0.118		0.254	0.254
SD			0.135	0.125		0.188	0.188

Table D. Continued Greywacke.

Stone	Fraction	LAB 1 Ni µg/l	min r mg/m²	max r mg/m²	LAB 2 Ni µg/l	min r mg/m²	max r mg/m²
1	1	5.77	0.462	0.462	0.068	0.005	0.005
1	2	2.71	0.217	0.217	0.100	0.008	0.008
1	3	2.57	0.206	0.206	0.098	0.008	0.008
1	4	3.15	0.252	0.252	0.121	0.010	0.010
1	5	7.33	0.587	0.587	0.153	0.012	0.012
1	6	3.94	0.315	0.315	0.235	0.019	0.019
1	7	4.39	0.351	0.351	0.373	0.030	0.030
1	8	3.65	0.292	0.292	0.347	0.028	0.028
2	1	0.33	0.026	0.026	0.177	0.014	0.014
2	2	0.30	0.024	0.024	0.058	0.005	0.005
2	3	0.24	0.019	0.019	0.043	0.003	0.003
2	4	0.24	0.019	0.019	0.023	0.002	0.002
2	5	0.27	0.021	0.021	0.039	0.003	0.003
2	6	0.23	0.019	0.019	0.046	0.004	0.004
2	7	0.23	0.018	0.018	0.085	0.007	0.007
2	8	0.19	0.015	0.015	0.114	0.009	0.009
3	1	0.51	0.041	0.041	0.074	0.006	0.006
3	2	0.45	0.036	0.036	0.044	0.004	0.004
3	3	0.17	0.013	0.013	0.044	0.004	0.004
3	4	0.41	0.033	0.033	0.091	0.007	0.007
3	5	0.68	0.054	0.054	0.044	0.004	0.004
3	6	0.31	0.025	0.025	0.034	0.003	0.003
3	7	0.37	0.030	0.030	0.072	0.006	0.006
3	8	0.31	0.025	0.025	0.100	0.008	0.008
4	1	1.30	0.104	0.104	0.106	0.009	0.009
4	2	0.94	0.076	0.076	0.089	0.007	0.007
4	3	0.88	0.070	0.070	0.055	0.004	0.004
4	4	0.77	0.062	0.062	0.045	0.004	0.004
4	5	1.05	0.084	0.084	0.099	0.008	0.008
4	6	0.85	0.068	0.068	0.118	0.009	0.009
4	7	0.62	0.050	0.050	0.149	0.012	0.012
4	8	0.51	0.041	0.041	0.159	0.013	0.013
5	1	0.78	0.062	0.062	0.204	0.016	0.016
5	2	0.53	0.042	0.042	0.028	0.002	0.002
5	3	0.14	0.011	0.011	<0.02	0.000	0.002
5	4	0.44	0.035	0.035	<0.02	0.000	0.002
5	5	2.34	0.187	0.187	<0.02	0.000	0.002
5	6	0.40	0.032	0.032	<0.02	0.000	0.002
5	7	0.32	0.026	0.026	<0.02	0.000	0.002
5	8	0.34	0.027	0.027	<0.02	0.000	0.002
			∑ min	∑ max		∑ min	∑ max
1			2.682	2.682		0.120	0.120
2			0.162	0.162		0.047	0.047
3			0.257	0.257		0.040	0.040
4			0.555	0.555		0.066	0.066
5			0.424	0.424		0.019	0.028
Mean			0.816	0.816		0.058	0.060
SD			1.054	1.054		0.038	0.036

Table D. Continued Greywacke.

Stone	Fraction	LAB 1 Pb µg/l	min r mg/m^2	max r mg/m^2	LAB 2 Pb µg/l	min r mg/m^2	max r mg/m^2
1	1	0.72	0.058	0.058	0.02	0.001	0.001
1	2	0.13	0.010	0.010	0.10	0.008	0.008
1	3	0.11	0.009	0.009	0.03	0.002	0.002
1	4	0.06	0.005	0.005	0.01	0.001	0.001
1	5	0.16	0.013	0.013	0.01	0.001	0.001
1	6	0.36	0.029	0.029	0.01	0.001	0.001
1	7	0.38	0.030	0.030	0.01	0.001	0.001
1	8	0.21	0.017	0.017	0.01	0.001	0.001
2	1	0.14	0.011	0.011	0.06	0.005	0.005
2	2	0.07	0.006	0.006	0.10	0.008	0.008
2	3	0.07	0.006	0.006	0.20	0.016	0.016
2	4	0.07	0.006	0.006	0.12	0.009	0.009
2	5	0.09	0.007	0.007	0.13	0.010	0.010
2	6	0.10	0.008	0.008	0.21	0.017	0.017
2	7	0.06	0.005	0.005	0.25	0.020	0.020
2	8	0.10	0.008	0.008	0.29	0.023	0.023
3	1	1.53	0.123	0.123	0.02	0.001	0.001
3	2	0.07	0.006	0.006	0.03	0.002	0.002
3	3	0.08	0.006	0.006	0.01	0.001	0.001
3	4	0.96	0.077	0.077	0.01	0.001	0.001
3	5	0.17	0.013	0.013	0.01	0.001	0.001
3	6	0.24	0.019	0.019	0.01	0.001	0.001
3	7	0.05	0.004	0.004	0.02	0.001	0.001
3	8	0.10	0.008	0.008	0.02	0.002	0.002
4	1	1.35	0.108	0.108	0.04	0.003	0.003
4	2	0.42	0.034	0.034	0.05	0.004	0.004
4	3	0.41	0.033	0.033	0.09	0.007	0.007
4	4	0.33	0.027	0.027	0.03	0.003	0.003
4	5	0.54	0.043	0.043	0.03	0.002	0.002
4	6	0.47	0.038	0.038	0.03	0.002	0.002
4	7	0.06	0.005	0.005	0.04	0.003	0.003
4	8	0.03	0.002	0.002	0.10	0.008	0.008
5	1	0.27	0.022	0.022	0.02	0.002	0.002
5	2	0.14	0.011	0.011	0.02	0.002	0.002
5	3	0.04	0.003	0.003	0.01	0.001	0.001
5	4	0.06	0.005	0.005	0.01	0.001	0.001
5	5	0.12	0.010	0.010	0.01	0.001	0.001
5	6	0.21	0.017	0.017	0.02	0.002	0.002
5	7	0.06	0.005	0.005	0.07	0.006	0.006
5	8	0.04	0.003	0.003	0.02	0.002	0.002
			∑ min	∑ max		∑ min	∑ max
1			0.171	0.171		0.02	0.02
2			0.057	0.057		0.11	0.11
3			0.256	0.256		0.01	0.01
4			0.290	0.290		0.03	0.03
5			0.076	0.076		0.02	0.02
Mean			0.170	0.170		0.04	0.04
SD			0.104	0.104		0.04	0.04

Table D. Continued Greywacke.

Stone	Fraction	LAB 1 Sb µg/l	min r mg/m²	max r mg/m²	LAB 2 Sb µg/l	min r mg/m²	max r mg/m²
1	1	0.15	0.012	0.012	0.04	0.003	0.003
1	2	0.19	0.015	0.015	0.05	0.004	0.004
1	3	0.17	0.013	0.013	0.05	0.004	0.004
1	4	0.17	0.013	0.013	0.04	0.003	0.003
1	5	0.23	0.018	0.018	0.05	0.004	0.004
1	6	0.16	0.013	0.013	0.07	0.006	0.006
1	7	0.14	0.012	0.012	0.09	0.008	0.008
1	8	0.24	0.019	0.019	0.09	0.007	0.007
2	1	0.07	0.006	0.006	0.08	0.006	0.006
2	2	0.10	0.008	0.008	0.07	0.005	0.005
2	3	0.08	0.007	0.007	0.07	0.005	0.005
2	4	0.07	0.006	0.006	0.06	0.005	0.005
2	5	0.09	0.007	0.007	0.10	0.008	0.008
2	6	0.07	0.006	0.006	0.10	0.008	0.008
2	7	0.08	0.006	0.006	0.15	0.012	0.012
2	8	0.19	0.015	0.015	0.14	0.011	0.011
3	1	0.09	0.008	0.008	0.07	0.005	0.005
3	2	0.14	0.011	0.011	0.06	0.005	0.005
3	3	<0.06	0.0	0.005	0.06	0.004	0.004
3	4	0.08	0.006	0.006	0.05	0.004	0.004
3	5	0.12	0.010	0.010	0.08	0.006	0.006
3	6	0.11	0.009	0.009	0.08	0.006	0.006
3	7	0.10	0.008	0.008	0.10	0.008	0.008
3	8	0.20	0.016	0.016	0.10	0.008	0.008
4	1	0.13	0.011	0.011	0.14	0.011	0.011
4	2	0.12	0.009	0.009	0.13	0.010	0.010
4	3	0.16	0.013	0.013	0.11	0.009	0.009
4	4	0.12	0.010	0.010	0.10	0.008	0.008
4	5	0.16	0.013	0.013	0.13	0.011	0.011
4	6	0.20	0.016	0.016	0.13	0.010	0.010
4	7	0.16	0.013	0.013	0.17	0.014	0.014
4	8	0.39	0.031	0.031	0.15	0.012	0.012
5	1	0.12	0.010	0.010	0.06	0.005	0.005
5	2	0.08	0.006	0.006	0.04	0.003	0.003
5	3	<0.06	0.0	0.005	0.03	0.003	0.003
5	4	0.09	0.007	0.007	0.03	0.003	0.003
5	5	0.11	0.009	0.009	0.05	0.004	0.004
5	6	0.09	0.008	0.008	0.05	0.004	0.004
5	7	0.07	0.006	0.006	0.08	0.007	0.007
5	8	0.23	0.019	0.019	0.08	0.007	0.007
			∑ min	∑ max		∑ min	∑ max
1			0.115	0.115		0.04	0.04
2			0.060	0.060		0.06	0.06
3			0.068	0.072		0.05	0.05
4			0.115	0.115		0.08	0.08
5			0.064	0.069		0.03	0.03
Mean			0.085	0.087		0.05	0.05
SD			0.028	0.027		0.02	0.02

Table D. Continued Greywacke.

Stone	Fraction	LAB 1 Se µg/l	min r mg/m²	max r mg/m²	LAB 2 Se µg/l	min r mg/m²	max r mg/m²
1	1	0.06	0.005	0.005	<0.07	0.000	0.006
1	2	0.05	0.004	0.004	<0.07	0.000	0.006
1	3	0.05	0.004	0.004	<0.07	0.000	0.006
1	4	0.07	0.006	0.006	<0.07	0.000	0.006
1	5	0.12	0.010	0.010	<0.07	0.000	0.006
1	6	0.10	0.008	0.008	<0.07	0.000	0.006
1	7	0.16	0.013	0.013	<0.07	0.000	0.006
1	8	0.15	0.012	0.012	<0.07	0.000	0.006
2	1	<0.04	0.0	0.003	<0.07	0.000	0.006
2	2	<0.04	0.0	0.003	<0.07	0.000	0.006
2	3	<0.04	0.0	0.003	<0.07	0.000	0.006
2	4	<0.04	0.0	0.003	<0.07	0.000	0.006
2	5	<0.04	0.0	0.003	<0.07	0.000	0.006
2	6	<0.04	0.0	0.003	<0.07	0.000	0.006
2	7	<0.04	0.0	0.003	<0.07	0.000	0.006
2	8	<0.04	0.0	0.003	<0.07	0.000	0.006
3	1	<0.04	0.0	0.003	0.14	0.011	0.011
3	2	<0.04	0.0	0.003	0.10	0.008	0.008
3	3	<0.04	0.0	0.003	<0.07	0.000	0.006
3	4	<0.04	0.0	0.003	0.08	0.006	0.006
3	5	<0.04	0.0	0.003	0.11	0.009	0.009
3	6	<0.04	0.0	0.003	<0.07	0.000	0.006
3	7	<0.04	0.0	0.003	0.08	0.007	0.007
3	8	<0.04	0.0	0.003	0.08	0.006	0.006
4	1	<0.04	0.0	0.003	<0.07	0.000	0.006
4	2	0.050	0.004	0.004	<0.07	0.000	0.006
4	3	0.053	0.004	0.004	<0.07	0.000	0.006
4	4	0.068	0.005	0.005	<0.07	0.000	0.006
4	5	0.112	0.009	0.009	<0.07	0.000	0.006
4	6	0.087	0.007	0.007	<0.07	0.000	0.006
4	7	0.106	0.008	0.008	<0.07	0.000	0.006
4	8	0.106	0.008	0.008	<0.07	0.000	0.006
5	1	<0.04	0.0	0.003	<0.07	0.000	0.006
5	2	<0.04	0.0	0.003	<0.07	0.000	0.006
5	3	<0.04	0.0	0.003	<0.07	0.000	0.006
5	4	<0.04	0.0	0.003	<0.07	0.000	0.006
5	5	<0.04	0.0	0.003	<0.07	0.000	0.006
5	6	<0.04	0.0	0.003	<0.07	0.000	0.006
5	7	<0.04	0.0	0.003	<0.07	0.000	0.006
5	8	<0.04	0.0	0.003	<0.07	0.000	0.006
			∑ min	∑ max		∑ min	∑ max
1			0.060	0.060		0.000	0.045
2			0.000	0.026		0.000	0.045
3			0.000	0.026		0.047	0.058
4			0.047	0.050		0.000	0.045
5			0.000	0.026		0.000	0.045
Mean			0.021	0.037		0.009	0.047
SD			0.030	0.016		0.021	0.006

Table D. Continued Greywacke.

Stone	Fraction	LAB 1 Sn µg/l	min r mg/m^2	max r mg/m^2	LAB 2 Sn µg/l	min r mg/m^2	max r mg/m^2
1	1	0.14	0.011	0.011	<0.01	0.000	0.001
1	2	0.07	0.005	0.005	<0.01	0.000	0.001
1	3	0.04	0.003	0.003	<0.01	0.000	0.001
1	4	0.05	0.004	0.004	<0.01	0.000	0.001
1	5	0.10	0.008	0.008	<0.01	0.000	0.001
1	6	0.12	0.009	0.009	<0.01	0.000	0.001
1	7	0.07	0.006	0.006	<0.01	0.000	0.001
1	8	0.03	0.002	0.002	<0.01	0.000	0.001
2	1	0.10	0.008	0.008	<0.01	0.000	0.001
2	2	0.05	0.004	0.004	<0.01	0.000	0.001
2	3	0.10	0.008	0.008	<0.01	0.000	0.001
2	4	0.07	0.006	0.006	<0.01	0.000	0.001
2	5	0.07	0.006	0.006	<0.01	0.000	0.001
2	6	0.06	0.005	0.005	<0.01	0.000	0.001
2	7	<0.03	0.0	0.002	<0.01	0.000	0.001
2	8	<0.03	0.0	0.002	<0.01	0.000	0.001
3	1	2.20	0.176	0.176	<0.01	0.000	0.001
3	2	0.35	0.028	0.028	<0.01	0.000	0.001
3	3	0.15	0.012	0.012	<0.01	0.000	0.001
3	4	0.20	0.016	0.016	<0.01	0.000	0.001
3	5	0.29	0.023	0.023	<0.01	0.000	0.001
3	6	0.28	0.022	0.022	<0.01	0.000	0.001
3	7	0.22	0.017	0.017	<0.01	0.000	0.001
3	8	0.06	0.005	0.005	0.01	0.001	0.001
4	1	0.11	0.009	0.009	<0.01	0.000	0.001
4	2	0.08	0.007	0.007	<0.01	0.000	0.001
4	3	0.08	0.007	0.007	<0.01	0.000	0.001
4	4	0.08	0.007	0.007	<0.01	0.000	0.001
4	5	0.16	0.013	0.013	<0.01	0.000	0.001
4	6	0.15	0.012	0.012	<0.01	0.000	0.001
4	7	0.10	0.008	0.008	<0.01	0.000	0.001
4	8	0.04	0.003	0.003	<0.01	0.000	0.001
5	1	0.33	0.027	0.027	<0.01	0.000	0.001
5	2	0.36	0.029	0.029	<0.01	0.000	0.001
5	3	0.13	0.010	0.010	<0.01	0.000	0.001
5	4	0.25	0.020	0.020	<0.01	0.000	0.001
5	5	0.31	0.025	0.025	<0.01	0.000	0.001
5	6	0.33	0.027	0.027	<0.01	0.000	0.001
5	7	0.20	0.016	0.016	<0.01	0.000	0.001
5	8	0.06	0.005	0.005	<0.01	0.000	0.001
			∑ min	∑ max		∑ min	∑ max
1			0.049	0.049		0.000	0.006
2			0.036	0.041		0.000	0.006
3			0.300	0.300		0.001	0.006
4			0.065	0.065		0.000	0.006
5			0.159	0.159		0.000	0.006
Mean			0.122	0.123		0.000	0.006
SD			0.111	0.110		0.000	0.000

Table D. Continued Greywacke.

Stone	Fraction	LAB 1 Sr µg/l	min r mg/m²	max r mg/m²	LAB 2 Sr µg/l	min r mg/m²	max r mg/m²
1	1	9.46	0.76	0.76	0.77	0.06	0.06
1	2	8.74	0.70	0.70	<0.5	0.00	0.04
1	3	8.66	0.69	0.69	<0.5	0.00	0.04
1	4	9.11	0.73	0.73	<0.5	0.00	0.04
1	5	9.56	0.77	0.77	<0.5	0.00	0.04
1	6	8.66	0.69	0.69	0.69	0.06	0.06
1	7	9.81	0.78	0.78	1.15	0.09	0.09
1	8	5.54	0.44	0.44	1.08	0.09	0.09
2	1	9.18	0.73	0.73	1.23	0.10	0.10
2	2	8.40	0.67	0.67	<0.5	0.00	0.04
2	3	7.11	0.57	0.57	<0.5	0.00	0.04
2	4	7.98	0.64	0.64	0.62	0.05	0.05
2	5	8.01	0.64	0.64	1.30	0.10	0.10
2	6	7.58	0.61	0.61	1.46	0.12	0.12
2	7	8.41	0.67	0.67	3.06	0.24	0.24
2	8	11.14	0.89	0.89	3.21	0.26	0.26
3	1	9.92	0.79	0.79	0.54	0.04	0.04
3	2	9.15	0.73	0.73	<0.5	0.00	0.04
3	3	1.67	0.13	0.13	<0.5	0.00	0.04
3	4	8.77	0.70	0.70	0.54	0.04	0.04
3	5	11.01	0.88	0.88	<0.5	0.00	0.04
3	6	11.97	0.96	0.96	<0.5	0.00	0.04
3	7	14.48	1.16	1.16	<0.5	0.00	0.04
3	8	19.88	1.59	1.59	<0.5	0.00	0.04
4	1	9.24	0.74	0.74	<0.5	0.00	0.04
4	2	9.01	0.72	0.72	<0.5	0.00	0.04
4	3	8.19	0.65	0.65	<0.5	0.00	0.04
4	4	8.74	0.70	0.70	<0.5	0.00	0.04
4	5	9.91	0.79	0.79	0.54	0.04	0.04
4	6	11.95	0.96	0.96	1.07	0.09	0.09
4	7	13.70	1.10	1.10	1.99	0.16	0.16
4	8	11.52	0.92	0.92	1.76	0.14	0.14
5	1	10.57	0.85	0.85	1.29	0.10	0.10
5	2	9.62	0.77	0.77	0.68	0.05	0.05
5	3	2.51	0.20	0.20	0.68	0.05	0.05
5	4	9.15	0.73	0.73	0.76	0.06	0.06
5	5	11.64	0.93	0.93	1.67	0.13	0.13
5	6	8.64	0.69	0.69	2.21	0.18	0.18
5	7	17.25	1.38	1.38	3.65	0.29	0.29
5	8	19.59	1.57	1.57	3.88	0.31	0.31
			∑ min	∑ max		∑ min	∑ max
1			5.56	5.56		0.30	0.46
2			5.43	5.43		0.87	0.95
3			6.95	6.95		0.09	0.33
4			6.58	6.58		0.43	0.59
5			7.12	7.12		1.19	1.19
Mean			6.33	6.33		0.57	0.70
SD			0.79	0.79		0.45	0.36

Table D. Continued Greywacke.

Stone	Fraction	LAB 1 U µg/l	min r mg/m²	max r mg/m²	LAB 2 U µg/l	min r mg/m²	max r mg/m²
1	1	<0.04	0.0	0.003	0.010	0.001	0.001
1	2	<0.04	0.0	0.003	0.009	0.001	0.001
1	3	<0.04	0.0	0.003	0.008	0.001	0.001
1	4	<0.04	0.0	0.003	0.007	0.001	0.001
1	5	0.042	0.003	0.003	0.009	0.001	0.001
1	6	0.041	0.003	0.003	0.009	0.001	0.001
1	7	0.037	0.003	0.003	0.012	0.001	0.001
1	8	0.035	0.003	0.003	0.012	0.001	0.001
2	1	<0.04	0.0	0.003	0.012	0.001	0.001
2	2	<0.04	0.0	0.003	0.007	0.001	0.001
2	3	<0.04	0.0	0.003	0.006	0.000	0.000
2	4	<0.04	0.0	0.003	0.006	0.000	0.000
2	5	<0.04	0.0	0.003	0.010	0.001	0.001
2	6	<0.04	0.0	0.003	0.010	0.001	0.001
2	7	<0.04	0.0	0.003	0.013	0.001	0.001
2	8	<0.04	0.0	0.003	0.011	0.001	0.001
3	1	<0.04	0.0	0.003	0.008	0.001	0.001
3	2	<0.04	0.0	0.003	0.010	0.001	0.001
3	3	<0.04	0.0	0.003	0.017	0.001	0.001
3	4	<0.04	0.0	0.003	0.024	0.002	0.002
3	5	0.064	0.005	0.005	0.045	0.004	0.004
3	6	0.072	0.006	0.006	0.046	0.004	0.004
3	7	0.131	0.010	0.010	0.057	0.005	0.005
3	8	0.185	0.015	0.015	0.046	0.004	0.004
4	1	<0.04	0.0	0.003	0.011	0.001	0.001
4	2	<0.04	0.0	0.003	0.008	0.001	0.001
4	3	<0.04	0.0	0.003	0.007	0.001	0.001
4	4	<0.04	0.0	0.003	0.007	0.001	0.001
4	5	<0.04	0.0	0.003	0.010	0.001	0.001
4	6	<0.04	0.0	0.003	0.011	0.001	0.001
4	7	<0.04	0.0	0.003	0.014	0.001	0.001
4	8	<0.04	0.0	0.003	0.013	0.001	0.001
5	1	0.055	0.004	0.004	0.010	0.001	0.001
5	2	<0.04	0.0	0.003	0.009	0.001	0.001
5	3	0.048	0.004	0.004	0.008	0.001	0.001
5	4	0.055	0.004	0.004	0.009	0.001	0.001
5	5	0.139	0.011	0.011	0.016	0.001	0.001
5	6	0.160	0.013	0.013	0.019	0.002	0.002
5	7	0.324	0.026	0.026	0.036	0.003	0.003
5	8	0.403	0.032	0.032	0.041	0.003	0.003
			∑ min	∑ max		∑ min	∑ max
1			0.012	0.025		0.006	0.006
2			0.000	0.026		0.006	0.006
3			0.036	0.049		0.020	0.020
4			0.000	0.026		0.006	0.006
5			0.095	0.098		0.012	0.012
Mean			0.029	0.045		0.010	0.010
SD			0.040	0.031		0.006	0.006

Table D. Continued Greywacke.

Stone	Fraction	LAB 1<br>V<br>µg/l	min<br>r<br>mg/m²	max<br>r<br>mg/m²	LAB 2<br>V<br>µg/l	min<br>r<br>mg/m²	max<br>r<br>mg/m²
1	1	0.029	0.002	0.002	0.010	0.001	0.001
1	2	0.022	0.002	0.002	0.017	0.001	0.001
1	3	<0.02	0.000	0.002	<0.01	0.000	0.001
1	4	<0.02	0.000	0.002	0.027	0.002	0.002
1	5	<0.02	0.000	0.002	<0.01	0.000	0.001
1	6	<0.02	0.000	0.002	<0.01	0.000	0.001
1	7	0.029	0.002	0.002	<0.01	0.000	0.001
1	8	<0.02	0.000	0.002	<0.01	0.000	0.001
2	1	0.055	0.004	0.004	0.038	0.003	0.003
2	2	0.030	0.002	0.002	0.015	0.001	0.001
2	3	0.024	0.002	0.002	<0.01	0.000	0.001
2	4	<0.02	0.000	0.002	<0.01	0.000	0.001
2	5	<0.02	0.000	0.002	<0.01	0.000	0.001
2	6	<0.02	0.000	0.002	<0.01	0.000	0.001
2	7	0.026	0.002	0.002	<0.01	0.000	0.001
2	8	0.035	0.003	0.003	<0.01	0.000	0.001
3	1	0.041	0.003	0.003	0.027	0.002	0.002
3	2	0.100	0.008	0.008	0.013	0.001	0.001
3	3	0.030	0.002	0.002	0.010	0.001	0.001
3	4	0.035	0.003	0.003	0.054	0.004	0.004
3	5	0.044	0.004	0.004	0.013	0.001	0.001
3	6	0.031	0.003	0.003	0.013	0.001	0.001
3	7	0.050	0.004	0.004	<0.01	0.000	0.001
3	8	0.032	0.003	0.003	0.011	0.001	0.001
4	1	0.022	0.002	0.002	0.018	0.001	0.001
4	2	<0.02	0.000	0.002	0.013	0.001	0.001
4	3	<0.02	0.000	0.002	0.010	0.001	0.001
4	4	<0.02	0.000	0.002	0.058	0.005	0.005
4	5	<0.02	0.000	0.002	0.011	0.001	0.001
4	6	<0.02	0.000	0.002	<0.01	0.000	0.001
4	7	0.022	0.002	0.002	<0.01	0.000	0.001
4	8	<0.02	0.000	0.002	<0.01	0.000	0.001
5	1	0.049	0.004	0.004	0.019	0.002	0.002
5	2	0.046	0.004	0.004	<0.01	0.000	0.001
5	3	0.030	0.002	0.002	<0.01	0.000	0.001
5	4	0.029	0.002	0.002	0.075	0.006	0.006
5	5	0.039	0.003	0.003	0.011	0.001	0.001
5	6	0.024	0.002	0.002	<0.01	0.000	0.001
5	7	0.156	0.012	0.012	<0.01	0.000	0.001
5	8	<0.02	0.000	0.002	<0.01	0.000	0.001
			∑ min	∑ max		∑ min	∑ max
1			0.006	0.014		0.004	0.008
2			0.013	0.018		0.004	0.009
3			0.029	0.029		0.011	0.012
4			0.004	0.013		0.009	0.011
5			0.030	0.031		0.008	0.012
Mean			0.016	0.021		0.007	0.011
SD			0.012	0.008		0.003	0.002

Table D. Continued Greywacke.

Stone	Fraction	LAB 1 Zn µg/l	min r mg/m^2	max r mg/m^2	LAB 2 Zn µg/l	min r mg/m^2	max r mg/m^2
1	1	513	41.04	41.04	0.93	0.07	0.07
1	2	483	38.64	38.64	4.30	0.34	0.34
1	3	478	38.28	38.28	1.61	0.13	0.13
1	4	498	39.82	39.82	7.40	0.59	0.59
1	5	470	37.63	37.63	1.70	0.14	0.14
1	6	459	36.69	36.69	2.15	0.17	0.17
1	7	469	37.54	37.54	1.76	0.14	0.14
1	8	268	21.43	21.43	1.99	0.16	0.16
2	1	468	37.45	37.45	0.33	0.03	0.03
2	2	480	38.40	38.40	0.87	0.07	0.07
2	3	427	34.15	34.15	0.58	0.05	0.05
2	4	285	22.79	22.79	0.70	0.06	0.06
2	5	408	32.62	32.62	1.39	0.11	0.11
2	6	247	19.72	19.72	1.13	0.09	0.09
2	7	443	35.45	35.45	1.66	0.13	0.13
2	8	623	49.80	49.80	0.98	0.08	0.08
3	1	392	31.36	31.36	0.14	0.01	0.01
3	2	160	12.79	12.79	0.39	0.03	0.03
3	3	45	3.60	3.60	0.43	0.03	0.03
3	4	314	25.14	25.14	4.78	0.38	0.38
3	5	324	25.93	25.93	0.33	0.03	0.03
3	6	492	39.35	39.35	0.25	0.02	0.02
3	7	461	36.90	36.90	0.35	0.03	0.03
3	8	704	56.30	56.30	0.42	0.03	0.03
4	1	328	26.21	26.21	0.37	0.03	0.03
4	2	491	39.31	39.31	0.62	0.05	0.05
4	3	270	21.57	21.57	0.68	0.05	0.05
4	4	448	35.83	35.83	7.64	0.61	0.61
4	5	313	25.01	25.01	0.46	0.04	0.04
4	6	556	44.49	44.49	1.53	0.12	0.12
4	7	530	42.37	42.37	0.67	0.05	0.05
4	8	321	25.71	25.71	0.76	0.06	0.06
5	1	458	36.65	36.65	0.11	0.01	0.01
5	2	271	21.69	21.69	1.90	0.15	0.15
5	3	28	2.26	2.26	0.17	0.01	0.01
5	4	283	22.60	22.60	6.83	0.55	0.55
5	5	430	34.43	34.43	0.40	0.03	0.03
5	6	320	25.61	25.61	0.38	0.03	0.03
5	7	472	37.72	37.72	0.33	0.03	0.03
5	8	363	29.05	29.05	0.42	0.03	0.03
			∑ min	∑ max		∑ min	∑ max
1			291	291		1.75	1.75
2			270	270		0.61	0.61
3			231	231		0.57	0.57
4			261	261		1.02	1.02
5			210	210		0.84	0.84
Mean			253	253		0.96	0.96
SD			32	32		0.48	0.48

Table E. DSLT results Granodiorite (LAB 1 DSLT contract laboratory, LAB 2 DSLT BfG).

Stone	Fraction	LAB 1 Al µg/l	min r mg/m²	max r mg/m²	LAB 2 Al µg/l	min r mg/m²	max r mg/m²
1	1	<40.8	0.0	3.26	<40.8	0.0	3.27
1	2	<40.8	0.0	3.26	<40.8	0.0	3.27
1	3	<40.8	0.0	3.26	<40.8	0.0	3.27
1	4	<40.8	0.0	3.26	<40.8	0.0	3.27
1	5	<40.8	0.0	3.26	<40.8	0.0	3.27
1	6	<40.8	0.0	3.26	<40.8	0.0	3.27
1	7	<40.8	0.0	3.26	<40.8	0.0	3.27
1	8	<40.8	0.0	3.26	<40.8	0.0	3.27
2	1	<40.8	0.0	3.26	<40.8	0.0	3.26
2	2	<40.8	0.0	3.26	<40.8	0.0	3.26
2	3	<40.8	0.0	3.26	<40.8	0.0	3.26
2	4	<40.8	0.0	3.26	<40.8	0.0	3.26
2	5	<40.8	0.0	3.26	<40.8	0.0	3.26
2	6	<40.8	0.0	3.26	<40.8	0.0	3.26
2	7	<40.8	0.0	3.26	<40.8	0.0	3.26
2	8	<40.8	0.0	3.26	<40.8	0.0	3.26
3	1	<40.8	0.0	3.26	<40.8	0.0	3.27
3	2	<40.8	0.0	3.26	<40.8	0.0	3.27
3	3	<40.8	0.0	3.26	<40.8	0.0	3.27
3	4	<40.8	0.0	3.26	<40.8	0.0	3.27
3	5	<40.8	0.0	3.26	<40.8	0.0	3.27
3	6	<40.8	0.0	3.26	<40.8	0.0	3.27
3	7	<40.8	0.0	3.26	<40.8	0.0	3.27
3	8	<40.8	0.0	3.26	<40.8	0.0	3.27
4	1	<40.8	0.0	3.26	<40.8	0.0	3.26
4	2	<40.8	0.0	3.26	<40.8	0.0	3.26
4	3	<40.8	0.0	3.26	<40.8	0.0	3.26
4	4	<40.8	0.0	3.26	<40.8	0.0	3.26
4	5	<40.8	0.0	3.26	<40.8	0.0	3.26
4	6	<40.8	0.0	3.26	<40.8	0.0	3.26
4	7	<40.8	0.0	3.26	<40.8	0.0	3.26
4	8	335.6	26.8	26.84	<40.8	0.0	3.26
5	1	<40.8	0.0	3.26	<40.8	0.0	3.26
5	2	<40.8	0.0	3.26	<40.8	0.0	3.26
5	3	<40.8	0.0	3.26	<40.8	0.0	3.26
5	4	<40.8	0.0	3.26	<40.8	0.0	3.26
5	5	<40.8	0.0	3.26	<40.8	0.0	3.26
5	6	<40.8	0.0	3.26	<40.8	0.0	3.26
5	7	<40.8	0.0	3.26	<40.8	0.0	3.26
5	8	<40.8	0.0	3.26	<40.8	0.0	3.26
			∑ min	∑ max		∑ min	∑ max
1			0.0	26.12		0.00	26.15
2			0.0	26.12		0.00	26.08
3			0.0	26.11		0.00	26.13
4			26.8	49.69		0.00	26.11
5			0.0	26.12		0.00	26.10
Mean			5.4	30.83		0.00	26.11
SD			12.0	10.54		0.00	0.03

Table E. Continued Granodiorite.

Stone	Fraction	LAB 1 As µg/l	min r mg/m^2	max r mg/m^2	LAB 2 As µg/l	min r mg/m^2	max r mg/m^2
1	1	0.39	0.031	0.031	<0.06	0.000	0.005
1	2	0.42	0.033	0.033	<0.06	0.000	0.005
1	3	0.36	0.029	0.029	<0.06	0.000	0.005
1	4	0.35	0.028	0.028	0.07	0.005	0.005
1	5	0.75	0.060	0.060	0.18	0.015	0.015
1	6	0.69	0.055	0.055	0.19	0.015	0.015
1	7	0.68	0.055	0.055	0.43	0.035	0.035
1	8	0.68	0.054	0.054	0.36	0.029	0.029
2	1	0.20	0.016	0.016	<0.06	0.000	0.005
2	2	0.23	0.018	0.018	0.06	0.005	0.005
2	3	0.29	0.023	0.023	0.06	0.005	0.005
2	4	0.35	0.028	0.028	0.06	0.005	0.005
2	5	0.98	0.079	0.079	0.17	0.014	0.014
2	6	1.17	0.093	0.093	0.20	0.016	0.016
2	7	1.88	0.150	0.150	0.45	0.036	0.036
2	8	3.38	0.270	0.270	0.34	0.027	0.027
3	1	0.15	0.012	0.012	<0.06	0.000	0.005
3	2	0.18	0.015	0.015	0.07	0.005	0.005
3	3	0.23	0.019	0.019	0.10	0.008	0.008
3	4	0.25	0.020	0.020	0.12	0.010	0.010
3	5	0.67	0.054	0.054	0.31	0.024	0.024
3	6	0.71	0.056	0.056	0.35	0.028	0.028
3	7	0.75	0.060	0.060	0.86	0.069	0.069
3	8	1.20	0.096	0.096	0.88	0.070	0.070
4	1	0.10	0.008	0.008	0.10	0.008	0.008
4	2	0.14	0.011	0.011	0.09	0.007	0.007
4	3	0.21	0.017	0.017	0.08	0.007	0.007
4	4	0.25	0.020	0.020	0.07	0.006	0.006
4	5	0.65	0.052	0.052	0.13	0.011	0.011
4	6	0.65	0.052	0.052	0.14	0.011	0.011
4	7	0.81	0.065	0.065	0.27	0.021	0.021
4	8	1.74	0.139	0.139	0.22	0.018	0.018
5	1	0.22	0.017	0.017	0.15	0.012	0.012
5	2	0.24	0.020	0.020	0.15	0.012	0.012
5	3	0.27	0.022	0.022	0.14	0.011	0.011
5	4	0.30	0.024	0.024	0.11	0.009	0.009
5	5	0.79	0.063	0.063	0.18	0.014	0.014
5	6	0.84	0.067	0.067	0.18	0.015	0.015
5	7	1.13	0.090	0.090	0.36	0.029	0.029
5	8	2.07	0.166	0.166	0.28	0.022	0.022
			∑ min	∑ max		∑ min	∑ max
1			0.345	0.345		0.099	0.114
2			0.678	0.678		0.108	0.113
3			0.332	0.332		0.215	0.220
4			0.363	0.363		0.088	0.088
5			0.469	0.469		0.123	0.123
Mean			0.438	0.438		0.127	0.131
SD			0.145	0.145		0.051	0.051

Table E. Continued Granodiorite.

Stone	Fraction	LAB 1 Ba µg/l	min r mg/m^2	max r mg/m^2	LAB 2 Ba µg/l	min r mg/m^2	max r mg/m^2
1	1	444	35.56	35.56	0.99	0.08	0.08
1	2	455	36.36	36.36	0.91	0.07	0.07
1	3	414	33.16	33.16	1.18	0.09	0.09
1	4	445	35.64	35.64	1.05	0.08	0.08
1	5	442	35.32	35.32	0.89	0.07	0.07
1	6	132	10.54	10.54	0.83	0.07	0.07
1	7	443	35.48	35.48	1.08	0.09	0.09
1	8	330	26.38	26.38	1.24	0.10	0.10
2	1	442	35.34	35.34	0.9	0.07	0.07
2	2	510	40.79	40.79	1.0	0.08	0.08
2	3	424	33.95	33.95	1.44	0.12	0.12
2	4	482	38.55	38.55	0.83	0.07	0.07
2	5	495	39.64	39.64	1.14	0.09	0.09
2	6	456	36.52	36.52	1.22	0.10	0.10
2	7	452	36.18	36.18	1.54	0.12	0.12
2	8	415	33.17	33.17	1.35	0.11	0.11
3	1	482	38.57	38.57	1.0	0.08	0.08
3	2	465	37.18	37.18	0.81	0.06	0.06
3	3	448	35.85	35.85	1.23	0.10	0.10
3	4	467	37.34	37.34	1.07	0.09	0.09
3	5	401	32.05	32.05	0.83	0.07	0.07
3	6	389	31.09	31.09	0.97	0.08	0.08
3	7	459	36.73	36.73	0.9	0.07	0.07
3	8	404	32.33	32.33	0.8	0.07	0.07
4	1	434	34.68	34.68	1.14	0.09	0.09
4	2	430	34.42	34.42	0.92	0.07	0.07
4	3	428	34.26	34.26	1.41	0.11	0.11
4	4	421	33.71	33.71	0.83	0.07	0.07
4	5	437	34.91	34.91	1.08	0.09	0.09
4	6	448	35.79	35.79	1.08	0.09	0.09
4	7	445	35.60	35.60	1.48	0.12	0.12
4	8	892	71.33	71.33	1.50	0.12	0.12
5	1	486	38.90	38.90	0.9	0.08	0.08
5	2	464	37.16	37.16	1.0	0.08	0.08
5	3	374	29.93	29.93	1.15	0.09	0.09
5	4	458	36.61	36.61	0.80	0.06	0.06
5	5	466	37.25	37.25	1.20	0.10	0.10
5	6	471	37.69	37.69	1.47	0.12	0.12
5	7	486	38.85	38.85	2.71	0.22	0.22
5	8	331	26.48	26.48	2.23	0.18	0.18
			∑ min	∑ max		∑ min	∑ max
1			248	248		0.65	0.65
2			294	294		0.75	0.75
3			281	281		0.61	0.61
4			315	315		0.75	0.75
5			283	283		0.92	0.92
Mean			284	284		0.74	0.74
SD			24	24		0.12	0.12

Table E. Continued Granodiorite.

Stone	Fraction	LAB 1 Cd µg/l	min r mg/m²	max r mg/m²	LAB 2 Cd µg/l	min r mg/m²	max r mg/m²
1	1	0.03	0.002	0.002	<0.02	0.000	0.002
1	2	0.03	0.002	0.002	<0.02	0.000	0.002
1	3	0.02	0.002	0.002	<0.02	0.000	0.002
1	4	0.03	0.002	0.002	<0.02	0.000	0.002
1	5	0.02	0.002	0.002	<0.02	0.000	0.002
1	6	0.02	0.001	0.001	<0.02	0.000	0.002
1	7	0.03	0.002	0.002	<0.02	0.000	0.002
1	8	0.03	0.002	0.002	<0.02	0.000	0.002
2	1	0.03	0.002	0.002	<0.02	0.000	0.002
2	2	0.02	0.002	0.002	<0.02	0.000	0.002
2	3	0.03	0.002	0.002	<0.02	0.000	0.002
2	4	0.03	0.002	0.002	<0.02	0.000	0.002
2	5	0.03	0.003	0.003	<0.02	0.000	0.002
2	6	0.03	0.002	0.002	<0.02	0.000	0.002
2	7	0.03	0.002	0.002	<0.02	0.000	0.002
2	8	0.03	0.002	0.002	<0.02	0.000	0.002
3	1	0.03	0.002	0.002	<0.02	0.000	0.002
3	2	0.03	0.002	0.002	<0.02	0.000	0.002
3	3	0.03	0.003	0.003	<0.02	0.000	0.002
3	4	0.02	0.002	0.002	<0.02	0.000	0.002
3	5	0.02	0.002	0.002	<0.02	0.000	0.002
3	6	0.02	0.002	0.002	<0.02	0.000	0.002
3	7	0.02	0.002	0.002	<0.02	0.000	0.002
3	8	0.02	0.002	0.002	<0.02	0.000	0.002
4	1	0.03	0.002	0.002	<0.02	0.000	0.002
4	2	0.03	0.002	0.002	<0.02	0.000	0.002
4	3	0.03	0.002	0.002	<0.02	0.000	0.002
4	4	0.02	0.002	0.002	<0.02	0.000	0.002
4	5	0.03	0.002	0.002	<0.02	0.000	0.002
4	6	0.03	0.002	0.002	<0.02	0.000	0.002
4	7	0.03	0.002	0.002	<0.02	0.000	0.002
4	8	0.04	0.003	0.003	<0.02	0.000	0.002
5	1	0.03	0.002	0.002	<0.02	0.000	0.002
5	2	0.03	0.002	0.002	<0.02	0.000	0.002
5	3	0.02	0.002	0.002	<0.02	0.000	0.002
5	4	0.03	0.002	0.002	<0.02	0.000	0.002
5	5	0.03	0.002	0.002	<0.02	0.000	0.002
5	6	0.03	0.003	0.003	<0.02	0.000	0.002
5	7	0.02	0.002	0.002	<0.02	0.000	0.002
5	8	0.02	0.002	0.002	<0.02	0.000	0.002
			∑ min	∑ max		∑ min	∑ max
1			0.017	0.017		0.000	0.013
2			0.018	0.018		0.000	0.013
3			0.017	0.017		0.000	0.013
4			0.019	0.019		0.000	0.013
5			0.017	0.017		0.000	0.013
Mean			0.017	0.017		0.000	0.013
SD			0.001	0.001		0.000	0.000

Table E. Continued Granodiorite.

Stone	Fraction	LAB 1 Co µg/l	min r mg/m²	max r mg/m²	LAB 2 Co µg/l	min r mg/m²	max r mg/m²
1	1	0.17	0.014	0.014	0.03	0.003	0.003
1	2	0.20	0.016	0.016	<0.02	0.000	0.002
1	3	0.23	0.019	0.019	<0.02	0.000	0.002
1	4	0.25	0.020	0.020	<0.02	0.000	0.002
1	5	0.45	0.036	0.036	0.03	0.002	0.002
1	6	0.44	0.035	0.035	0.03	0.002	0.002
1	7	0.51	0.041	0.041	0.04	0.003	0.003
1	8	0.68	0.055	0.055	0.04	0.003	0.003
2	1	0.10	0.008	0.008	0.02	0.002	0.002
2	2	0.12	0.010	0.010	<0.02	0.000	0.002
2	3	0.12	0.009	0.009	<0.02	0.000	0.002
2	4	0.11	0.009	0.009	<0.02	0.000	0.002
2	5	0.15	0.012	0.012	0.03	0.002	0.002
2	6	0.16	0.013	0.013	0.04	0.003	0.003
2	7	0.16	0.013	0.013	0.05	0.004	0.004
2	8	0.20	0.016	0.016	0.05	0.004	0.004
3	1	0.12	0.009	0.009	<0.02	0.000	0.002
3	2	0.11	0.009	0.009	<0.02	0.000	0.002
3	3	0.13	0.011	0.011	<0.02	0.000	0.002
3	4	0.14	0.011	0.011	<0.02	0.000	0.002
3	5	0.26	0.021	0.021	0.03	0.002	0.002
3	6	0.24	0.019	0.019	0.04	0.003	0.003
3	7	0.22	0.017	0.017	0.05	0.004	0.004
3	8	0.23	0.018	0.018	0.06	0.004	0.004
4	1	0.14	0.011	0.011	0.04	0.003	0.003
4	2	0.12	0.010	0.010	<0.02	0.000	0.002
4	3	0.17	0.014	0.014	<0.02	0.000	0.002
4	4	0.22	0.018	0.018	<0.02	0.000	0.002
4	5	0.64	0.051	0.051	0.02	0.002	0.002
4	6	0.64	0.051	0.051	0.02	0.002	0.002
4	7	0.87	0.069	0.069	0.03	0.002	0.002
4	8	0.81	0.065	0.065	0.03	0.002	0.002
5	1	0.09	0.007	0.007	0.04	0.003	0.003
5	2	0.10	0.008	0.008	<0.02	0.000	0.002
5	3	0.10	0.008	0.008	<0.02	0.000	0.002
5	4	0.10	0.008	0.008	<0.02	0.000	0.002
5	5	0.12	0.009	0.009	<0.02	0.000	0.002
5	6	0.13	0.010	0.010	<0.02	0.000	0.002
5	7	0.12	0.010	0.010	0.02	0.000	0.002
5	8	0.15	0.012	0.012	0.04	0.000	0.003
			∑ min	∑ max		∑ min	∑ max
1			0.24	0.24		0.01	0.02
2			0.09	0.09		0.01	0.02
3			0.12	0.12		0.01	0.02
4			0.29	0.29		0.01	0.02
5			0.07	0.07		0.00	0.02
Mean			0.161	0.161		0.01	0.02
SD			0.096	0.096		0.00	0.00

Table E. Continued Granodiorite.

Stone	Fraction	LAB 1 Cr µg/l	min r mg/m^2	max r mg/m^2	LAB 2 Cr µg/l	min r mg/m^2	max r mg/m^2
1	1	0.06	0.005	0.005	<0.05	0.0	0.004
1	2	0.12	0.009	0.009	<0.05	0.0	0.004
1	3	0.12	0.010	0.010	<0.05	0.0	0.004
1	4	0.06	0.000	0.005	<0.05	0.0	0.004
1	5	0.07	0.006	0.006	<0.05	0.0	0.004
1	6	0.06	0.000	0.005	<0.05	0.0	0.004
1	7	0.06	0.005	0.005	<0.05	0.0	0.004
1	8	0.06	0.000	0.005	<0.05	0.0	0.004
2	1	0.08	0.006	0.006	<0.05	0.0	0.004
2	2	0.10	0.008	0.008	<0.05	0.0	0.004
2	3	0.43	0.035	0.035	<0.05	0.0	0.004
2	4	0.11	0.009	0.009	<0.05	0.0	0.004
2	5	0.07	0.005	0.005	<0.05	0.0	0.004
2	6	0.07	0.006	0.006	<0.05	0.0	0.004
2	7	0.06	0.000	0.005	<0.05	0.0	0.004
2	8	0.06	0.000	0.005	<0.05	0.0	0.004
3	1	0.08	0.006	0.006	<0.05	0.0	0.004
3	2	0.10	0.008	0.008	<0.05	0.0	0.004
3	3	0.24	0.019	0.019	<0.05	0.0	0.004
3	4	0.06	0.005	0.005	<0.05	0.0	0.004
3	5	0.06	0.005	0.005	<0.05	0.0	0.004
3	6	0.06	0.005	0.005	<0.05	0.0	0.004
3	7	0.06	0.000	0.005	<0.05	0.0	0.004
3	8	0.06	0.000	0.005	<0.05	0.0	0.004
4	1	0.06	0.005	0.005	<0.05	0.0	0.004
4	2	0.07	0.006	0.006	<0.05	0.0	0.004
4	3	0.06	0.005	0.005	<0.05	0.0	0.004
4	4	0.10	0.008	0.008	<0.05	0.0	0.004
4	5	0.14	0.012	0.012	<0.05	0.0	0.004
4	6	0.07	0.006	0.006	<0.05	0.0	0.004
4	7	0.09	0.007	0.007	<0.05	0.0	0.004
4	8	0.45	0.036	0.036	<0.05	0.0	0.004
5	1	0.09	0.007	0.007	<0.05	0.0	0.004
5	2	0.08	0.007	0.007	<0.05	0.0	0.004
5	3	0.08	0.007	0.007	<0.05	0.0	0.004
5	4	0.07	0.006	0.006	<0.05	0.0	0.004
5	5	0.08	0.007	0.007	<0.05	0.0	0.004
5	6	0.08	0.006	0.006	<0.05	0.0	0.004
5	7	0.07	0.006	0.006	<0.05	0.0	0.004
5	8	0.06	0.000	0.005	<0.05	0.0	0.004
			∑ min	∑ max		∑ min	∑ max
1			0.035	0.049		0.000	0.032
2			0.069	0.079		0.000	0.032
3			0.049	0.058		0.000	0.032
4			0.084	0.084		0.000	0.032
5			0.045	0.050		0.000	0.032
Mean			0.056	0.064		0.000	0.032
SD			0.020	0.016		0.000	0.000

Table E. Continued Granodiorite.

Stone	Fraction	LAB 1 Cu µg/l	min r mg/m^2	max r mg/m^2	LAB 2 Cu µg/l	min r mg/m^2	max r mg/m^2
1	1	4.39	0.351	0.351	0.12	0.010	0.010
1	2	2.39	0.191	0.191	<0.07	0.000	0.006
1	3	2.20	0.176	0.176	<0.07	0.000	0.006
1	4	1.85	0.148	0.148	<0.07	0.000	0.006
1	5	2.12	0.170	0.170	<0.07	0.000	0.006
1	6	2.29	0.183	0.183	<0.07	0.000	0.006
1	7	2.54	0.203	0.203	<0.07	0.000	0.006
1	8	1.61	0.129	0.129	<0.07	0.000	0.006
2	1	5.39	0.431	0.431	<0.07	0.000	0.006
2	2	3.09	0.247	0.247	<0.07	0.000	0.006
2	3	3.04	0.243	0.243	<0.07	0.000	0.006
2	4	2.42	0.194	0.194	<0.07	0.000	0.006
2	5	3.05	0.244	0.244	<0.07	0.000	0.006
2	6	2.63	0.210	0.210	<0.07	0.000	0.006
2	7	2.63	0.210	0.210	<0.07	0.000	0.006
2	8	1.53	0.122	0.122	<0.07	0.000	0.006
3	1	4.44	0.355	0.355	0.08	0.006	0.006
3	2	2.96	0.237	0.237	<0.07	0.000	0.006
3	3	2.69	0.215	0.215	<0.07	0.000	0.006
3	4	2.25	0.180	0.180	<0.07	0.000	0.006
3	5	2.49	0.199	0.199	<0.07	0.000	0.006
3	6	2.72	0.218	0.218	<0.07	0.000	0.006
3	7	2.04	0.163	0.163	<0.07	0.000	0.006
3	8	1.82	0.146	0.146	<0.07	0.000	0.006
4	1	3.57	0.285	0.285	1.16	0.093	0.093
4	2	2.78	0.223	0.223	<0.07	0.000	0.006
4	3	2.33	0.187	0.187	<0.07	0.000	0.006
4	4	2.41	0.193	0.193	<0.07	0.000	0.006
4	5	2.23	0.178	0.178	<0.07	0.000	0.006
4	6	2.02	0.162	0.162	<0.07	0.000	0.006
4	7	2.44	0.195	0.195	<0.07	0.000	0.006
4	8	1.25	0.100	0.100	<0.07	0.000	0.006
5	1	3.36	0.269	0.269	0.14	0.011	0.011
5	2	2.25	0.180	0.180	<0.07	0.000	0.006
5	3	2.05	0.164	0.164	<0.07	0.000	0.006
5	4	2.20	0.176	0.176	<0.07	0.000	0.006
5	5	2.38	0.190	0.190	<0.07	0.000	0.006
5	6	2.48	0.198	0.198	<0.07	0.000	0.006
5	7	1.85	0.148	0.148	<0.07	0.000	0.006
5	8	1.01	0.081	0.081	<0.07	0.000	0.006
			∑ min	∑ max		∑ min	∑ max
1			1.552	1.552		0.010	0.049
2			1.902	1.902		0.000	0.045
3			1.714	1.714		0.006	0.046
4			1.522	1.522		0.093	0.132
5			1.406	1.406		0.011	0.050
Mean			1.619	1.619		0.024	0.064
SD			0.193	0.193		0.039	0.038

Table E. Continued Granodiorite.

Stone	Fraction	LAB 1 Mn µg/l	min r mg/m^2	max r mg/m^2	LAB 2 Mn µg/l	min r mg/m^2	max r mg/m^2
1	1	1.24	0.099	0.099	0.43	0.034	0.034
1	2	0.80	0.064	0.064	0.42	0.034	0.034
1	3	0.97	0.078	0.078	0.46	0.037	0.037
1	4	0.96	0.077	0.077	0.37	0.030	0.030
1	5	1.97	0.158	0.158	0.49	0.039	0.039
1	6	2.10	0.168	0.168	0.37	0.030	0.030
1	7	2.74	0.220	0.220	0.55	0.044	0.044
1	8	5.00	0.400	0.400	0.40	0.032	0.032
2	1	1.63	0.130	0.130	0.31	0.025	0.025
2	2	1.26	0.101	0.101	0.40	0.032	0.032
2	3	1.13	0.090	0.090	0.48	0.038	0.038
2	4	1.20	0.096	0.096	0.45	0.036	0.036
2	5	1.51	0.121	0.121	0.54	0.043	0.043
2	6	1.40	0.112	0.112	0.40	0.032	0.032
2	7	1.21	0.097	0.097	0.57	0.045	0.045
2	8	1.24	0.099	0.099	0.45	0.036	0.036
3	1	1.16	0.093	0.093	0.31	0.025	0.025
3	2	0.66	0.053	0.053	0.33	0.026	0.026
3	3	0.68	0.055	0.055	0.35	0.028	0.028
3	4	0.77	0.061	0.061	0.36	0.029	0.029
3	5	1.51	0.121	0.121	0.60	0.048	0.048
3	6	1.42	0.113	0.113	0.59	0.048	0.048
3	7	1.32	0.105	0.105	0.86	0.069	0.069
3	8	1.40	0.112	0.112	0.64	0.051	0.051
4	1	0.57	0.046	0.046	0.77	0.062	0.062
4	2	0.40	0.032	0.032	0.69	0.055	0.055
4	3	0.51	0.041	0.041	0.81	0.065	0.065
4	4	0.62	0.050	0.050	0.69	0.055	0.055
4	5	1.77	0.142	0.142	0.69	0.055	0.055
4	6	1.87	0.149	0.149	0.53	0.043	0.043
4	7	3.00	0.240	0.240	0.72	0.057	0.057
4	8	4.05	0.324	0.324	0.53	0.042	0.042
5	1	1.07	0.086	0.086	0.44	0.035	0.035
5	2	1.08	0.086	0.086	0.54	0.043	0.043
5	3	0.92	0.074	0.074	0.61	0.048	0.048
5	4	0.91	0.073	0.073	0.61	0.048	0.048
5	5	1.35	0.108	0.108	0.63	0.051	0.051
5	6	1.18	0.095	0.095	0.54	0.043	0.043
5	7	1.24	0.100	0.100	0.93	0.000	0.075
5	8	1.24	0.099	0.099	0.97	0.000	0.077
			∑ min	∑ max		∑ min	∑ max
1			1.262	1.262		0.280	0.280
2			0.847	0.847		0.287	0.287
3			0.713	0.713		0.323	0.323
4			1.024	1.024		0.435	0.435
5			0.720	0.720		0.269	0.421
Mean			0.913	0.913		0.319	0.349
SD			0.232	0.232		0.07	0.07

Table E. Continued Granodiorite.

Stone	Fraction	LAB 1 Mo µg/l	min r mg/m^2	max r mg/m^2	LAB 2 Mo µg/l	min r mg/m^2	max r mg/m^2
1	1	0.13	0.011	0.011	<0.12	0.000	0.010
1	2	0.09	0.007	0.007	<0.12	0.000	0.010
1	3	0.08	0.006	0.006	<0.12	0.000	0.010
1	4	0.06	0.005	0.005	<0.12	0.000	0.010
1	5	0.10	0.008	0.008	<0.12	0.000	0.010
1	6	0.07	0.005	0.005	<0.12	0.000	0.010
1	7	0.09	0.007	0.007	<0.12	0.000	0.010
1	8	0.07	0.006	0.006	<0.12	0.000	0.010
2	1	0.05	0.004	0.004	<0.12	0.000	0.010
2	2	<0.06	0.000	0.005	<0.12	0.000	0.010
2	3	<0.06	0.000	0.005	<0.12	0.000	0.010
2	4	<0.06	0.000	0.005	<0.12	0.000	0.010
2	5	0.08	0.006	0.006	<0.12	0.000	0.010
2	6	0.07	0.006	0.006	<0.12	0.000	0.010
2	7	0.10	0.008	0.008	<0.12	0.000	0.010
2	8	0.13	0.011	0.011	<0.12	0.000	0.010
3	1	<0.06	0.000	0.005	<0.12	0.000	0.010
3	2	<0.06	0.000	0.005	<0.12	0.000	0.010
3	3	<0.06	0.000	0.005	<0.12	0.000	0.010
3	4	<0.06	0.000	0.005	<0.12	0.000	0.010
3	5	0.09	0.007	0.007	<0.12	0.000	0.010
3	6	0.06	0.005	0.005	<0.12	0.000	0.010
3	7	0.10	0.008	0.008	<0.12	0.000	0.010
3	8	0.12	0.009	0.009	<0.12	0.000	0.010
4	1	<0.06	0.000	0.005	<0.12	0.000	0.010
4	2	<0.06	0.000	0.005	<0.12	0.000	0.010
4	3	<0.06	0.000	0.005	<0.12	0.000	0.010
4	4	<0.06	0.000	0.005	<0.12	0.000	0.010
4	5	<0.06	0.000	0.005	<0.12	0.000	0.010
4	6	<0.06	0.000	0.005	<0.12	0.000	0.010
4	7	0.06	0.005	0.005	<0.12	0.000	0.010
4	8	0.06	0.005	0.005	<0.12	0.000	0.010
5	1	0.06	0.005	0.005	<0.12	0.000	0.010
5	2	<0.06	0.000	0.005	<0.12	0.000	0.010
5	3	<0.06	0.000	0.005	<0.12	0.000	0.010
5	4	<0.06	0.000	0.005	<0.12	0.000	0.010
5	5	<0.06	0.000	0.005	<0.12	0.000	0.010
5	6	<0.06	0.000	0.005	<0.12	0.000	0.010
5	7	<0.06	0.000	0.005	<0.12	0.000	0.010
5	8	<0.06	0.000	0.005	<0.12	0.000	0.010
			∑ min	∑ max		∑ min	∑ max
1			0.055	0.055		0.000	0.077
2			0.036	0.050		0.000	0.077
3			0.029	0.048		0.000	0.077
4			0.009	0.038		0.000	0.077
5			0.005	0.038		0.000	0.077
Mean			0.027	0.046		0.000	0.077
SD			0.020	0.007		0.000	0.000

Table E. Continued Granodiorite.

Stone	Fraction	LAB 1 Ni µg/l	min r mg/m^2	max r mg/m^2	LAB 2 Ni µg/l	min r mg/m^2	max r mg/m^2
1	1	0.53	0.042	0.042	0.15	0.012	0.012
1	2	0.40	0.032	0.032	0.07	0.006	0.006
1	3	0.43	0.035	0.035	0.05	0.004	0.004
1	4	0.46	0.036	0.036	0.05	0.004	0.004
1	5	0.79	0.063	0.063	0.07	0.006	0.006
1	6	0.73	0.058	0.058	0.05	0.004	0.004
1	7	0.93	0.075	0.075	0.06	0.005	0.005
1	8	1.08	0.086	0.086	0.08	0.006	0.006
2	1	0.63	0.051	0.051	0.10	0.008	0.008
2	2	0.52	0.041	0.041	0.06	0.005	0.005
2	3	0.47	0.037	0.037	0.07	0.005	0.005
2	4	0.39	0.031	0.031	0.04	0.004	0.004
2	5	0.89	0.071	0.071	0.08	0.007	0.007
2	6	0.53	0.043	0.043	0.08	0.006	0.006
2	7	0.66	0.053	0.053	0.09	0.007	0.007
2	8	0.38	0.030	0.030	0.09	0.007	0.007
3	1	0.54	0.043	0.043	0.15	0.012	0.012
3	2	0.36	0.028	0.028	0.06	0.005	0.005
3	3	0.40	0.032	0.032	0.07	0.005	0.005
3	4	0.35	0.028	0.028	0.07	0.006	0.006
3	5	0.93	0.075	0.075	0.09	0.007	0.007
3	6	0.64	0.052	0.052	0.12	0.009	0.009
3	7	0.65	0.052	0.052	0.10	0.008	0.008
3	8	0.47	0.038	0.038	0.08	0.006	0.006
4	1	0.42	0.033	0.033	2.21	0.177	0.177
4	2	0.35	0.028	0.028	0.07	0.006	0.006
4	3	0.49	0.039	0.039	0.06	0.005	0.005
4	4	0.51	0.041	0.041	0.04	0.003	0.003
4	5	1.30	0.104	0.104	0.05	0.004	0.004
4	6	1.18	0.094	0.094	0.06	0.005	0.005
4	7	1.61	0.129	0.129	0.06	0.005	0.005
4	8	1.31	0.105	0.105	0.06	0.005	0.005
5	1	0.40	0.032	0.032	0.13	0.011	0.011
5	2	0.41	0.033	0.033	0.03	0.002	0.002
5	3	0.40	0.032	0.032	0.04	0.000	0.003
5	4	0.32	0.026	0.026	0.03	0.000	0.003
5	5	0.48	0.038	0.038	0.04	0.000	0.003
5	6	0.40	0.032	0.032	<0.03	0.000	0.002
5	7	0.35	0.028	0.028	0.06	0.000	0.004
5	8	0.25	0.020	0.020	0.09	0.000	0.007
			∑ min	∑ max		∑ min	∑ max
1			0.428	0.428		0.047	0.047
2			0.357	0.357		0.049	0.049
3			0.347	0.347		0.059	0.059
4			0.574	0.574		0.209	0.209
5			0.241	0.241		0.013	0.036
Mean			0.389	0.389		0.075	0.080
SD			0.12	0.12		0.077	0.073

Table E. Continued Granodiorite.

Stone	Fraction	LAB 1 Pb µg/l	min r mg/m^2	max r mg/m^2	LAB 2 Pb µg/l	min r mg/m^2	max r mg/m^2
1	1	0.12	0.010	0.010	0.03	0.002	0.002
1	2	0.06	0.005	0.005	<0.03	0.000	0.002
1	3	0.07	0.005	0.005	<0.03	0.000	0.002
1	4	0.05	0.004	0.004	<0.03	0.000	0.002
1	5	0.06	0.004	0.004	<0.03	0.000	0.002
1	6	0.08	0.006	0.006	<0.03	0.000	0.002
1	7	0.05	0.004	0.004	<0.03	0.000	0.002
1	8	<0.03	0.000	0.002	<0.03	0.000	0.002
2	1	0.35	0.028	0.028	<0.03	0.000	0.002
2	2	0.07	0.006	0.006	<0.03	0.000	0.002
2	3	0.12	0.010	0.010	<0.03	0.000	0.002
2	4	0.10	0.008	0.008	<0.03	0.000	0.002
2	5	0.14	0.011	0.011	<0.03	0.000	0.002
2	6	0.10	0.008	0.008	<0.03	0.000	0.002
2	7	0.06	0.005	0.005	<0.03	0.000	0.002
2	8	0.05	0.004	0.004	<0.03	0.000	0.002
3	1	0.27	0.022	0.022	<0.03	0.000	0.002
3	2	0.04	0.003	0.003	<0.03	0.000	0.002
3	3	0.10	0.008	0.008	<0.03	0.000	0.002
3	4	0.05	0.004	0.004	<0.03	0.000	0.002
3	5	0.19	0.015	0.015	<0.03	0.000	0.002
3	6	0.07	0.005	0.005	<0.03	0.000	0.002
3	7	0.04	0.003	0.003	<0.03	0.000	0.002
3	8	0.04	0.003	0.003	<0.03	0.000	0.002
4	1	0.36	0.028	0.028	0.05	0.004	0.004
4	2	0.07	0.006	0.006	<0.03	0.000	0.002
4	3	0.09	0.008	0.008	<0.03	0.000	0.002
4	4	0.07	0.006	0.006	<0.03	0.000	0.002
4	5	0.42	0.034	0.034	<0.03	0.000	0.002
4	6	0.07	0.006	0.006	<0.03	0.000	0.002
4	7	0.06	0.005	0.005	<0.03	0.000	0.002
4	8	0.12	0.010	0.010	<0.03	0.000	0.002
5	1	0.45	0.036	0.036	<0.03	0.000	0.002
5	2	0.08	0.006	0.006	<0.03	0.000	0.002
5	3	0.08	0.006	0.006	<0.03	0.000	0.002
5	4	0.06	0.005	0.005	<0.03	0.000	0.002
5	5	0.08	0.006	0.006	<0.03	0.000	0.002
5	6	0.13	0.010	0.010	<0.03	0.000	0.002
5	7	0.06	0.004	0.004	<0.03	0.000	0.002
5	8	0.03	0.003	0.003	<0.03	0.000	0.002
			∑ min	∑ max		∑ min	∑ max
1			0.039	0.041		0.002	0.019
2			0.080	0.080		0.000	0.019
3			0.064	0.064		0.000	0.019
4			0.102	0.102		0.004	0.021
5			0.077	0.077		0.000	0.019
Mean			0.072	0.073		0.001	0.020
SD		0.04	0.011	0.011	0.03	0.000	0.001

Table E. Continued Granodiorite.

Stone	Fraction	LAB 1 Sb µg/l	min r mg/m²	max r mg/m²	LAB 2 Sb µg/l	min r mg/m²	max r mg/m²
1	1	0.11	0.009	0.009	<0.15	0.000	0.012
1	2	0.15	0.012	0.012	<0.15	0.000	0.012
1	3	0.14	0.011	0.011	<0.15	0.000	0.012
1	4	0.10	0.008	0.008	<0.15	0.000	0.012
1	5	0.15	0.012	0.012	<0.15	0.000	0.012
1	6	0.09	0.007	0.007	<0.15	0.000	0.012
1	7	0.08	0.007	0.007	0.19	0.015	0.015
1	8	0.20	0.016	0.016	0.24	0.019	0.019
2	1	0.11	0.009	0.009	<0.15	0.000	0.012
2	2	0.08	0.006	0.006	<0.15	0.000	0.012
2	3	0.09	0.007	0.007	<0.15	0.000	0.012
2	4	0.10	0.008	0.008	<0.15	0.000	0.012
2	5	0.16	0.013	0.013	<0.15	0.000	0.012
2	6	0.09	0.007	0.007	<0.15	0.000	0.012
2	7	0.10	0.008	0.008	<0.15	0.000	0.012
2	8	0.24	0.019	0.019	<0.15	0.000	0.012
3	1	0.10	0.008	0.008	<0.15	0.000	0.012
3	2	0.10	0.008	0.008	<0.15	0.000	0.012
3	3	0.11	0.009	0.009	<0.15	0.000	0.012
3	4	0.11	0.009	0.009	<0.15	0.000	0.012
3	5	0.23	0.019	0.019	<0.15	0.000	0.012
3	6	0.11	0.009	0.009	<0.15	0.000	0.012
3	7	0.10	0.008	0.008	<0.15	0.000	0.012
3	8	0.24	0.019	0.019	<0.15	0.000	0.012
4	1	0.08	0.006	0.006	<0.15	0.000	0.012
4	2	0.09	0.007	0.007	<0.15	0.000	0.012
4	3	0.08	0.006	0.006	<0.15	0.000	0.012
4	4	0.08	0.006	0.006	<0.15	0.000	0.012
4	5	0.31	0.025	0.025	<0.15	0.000	0.012
4	6	0.09	0.007	0.007	<0.15	0.000	0.012
4	7	0.09	0.007	0.007	<0.15	0.000	0.012
4	8	0.19	0.015	0.015	<0.15	0.000	0.012
5	1	0.10	0.008	0.008	<0.15	0.000	0.012
5	2	0.09	0.007	0.007	<0.15	0.000	0.012
5	3	0.12	0.010	0.010	<0.15	0.000	0.012
5	4	0.10	0.008	0.008	<0.15	0.000	0.012
5	5	0.12	0.010	0.010	<0.15	0.000	0.012
5	6	0.09	0.007	0.007	<0.15	0.000	0.012
5	7	0.09	0.007	0.007	<0.15	0.000	0.012
5	8	0.20	0.016	0.016	<0.15	0.000	0.012
			∑ min	∑ max		∑ min	∑ max
1			0.082	0.082		0.034	0.106
2			0.078	0.078		0.000	0.096
3			0.087	0.087		0.000	0.096
4			0.081	0.081		0.000	0.096
5			0.073	0.073		0.000	0.096
Mean			0.080	0.080		0.007	0.098
SD			0.005	0.005		0.015	0.005

Table E. Continued Granodiorite.

Stone	Fraction	LAB 1 Se µg/l	min r mg/m²	max r mg/m²	LAB 2 Se µg/l	min r mg/m²	max r mg/m²
1	1	0.07	0.01	0.01	<0.13	0.00	0.01
1	2	0.06	0.00	0.00	<0.13	0.00	0.01
1	3	0.04	0.00	0.00	<0.13	0.00	0.01
1	4	0.04	0.00	0.00	<0.13	0.00	0.01
1	5	0.08	0.01	0.01	<0.13	0.00	0.01
1	6	0.08	0.01	0.01	<0.13	0.00	0.01
1	7	0.16	0.01	0.01	<0.13	0.00	0.01
1	8	0.19	0.01	0.01	<0.13	0.00	0.01
2	1	0.06	0.00	0.00	<0.13	0.00	0.01
2	2	0.05	0.00	0.00	<0.13	0.00	0.01
2	3	0.05	0.00	0.00	<0.13	0.00	0.01
2	4	0.04	0.00	0.00	<0.13	0.00	0.01
2	5	0.08	0.01	0.01	<0.13	0.00	0.01
2	6	0.06	0.01	0.01	<0.13	0.00	0.01
2	7	0.06	0.01	0.01	<0.13	0.00	0.01
2	8	0.07	0.01	0.01	<0.13	0.00	0.01
3	1	0.06	0.00	0.00	<0.13	0.00	0.01
3	2	<0.04	0.00	0.00	<0.13	0.00	0.01
3	3	0.05	0.00	0.00	<0.13	0.00	0.01
3	4	<0.04	0.00	0.00	<0.13	0.00	0.01
3	5	0.06	0.01	0.01	<0.13	0.00	0.01
3	6	0.07	0.01	0.01	<0.13	0.00	0.01
3	7	0.08	0.01	0.01	<0.13	0.00	0.01
3	8	0.07	0.01	0.01	<0.13	0.00	0.01
4	1	0.09	0.01	0.01	<0.13	0.00	0.01
4	2	0.07	0.01	0.01	<0.13	0.00	0.01
4	3	0.07	0.01	0.01	<0.13	0.00	0.01
4	4	0.08	0.01	0.01	<0.13	0.00	0.01
4	5	0.20	0.02	0.02	<0.13	0.00	0.01
4	6	0.24	0.02	0.02	<0.13	0.00	0.01
4	7	0.43	0.03	0.03	<0.13	0.00	0.01
4	8	0.45	0.04	0.04	<0.13	0.00	0.01
5	1	0.05	0.00	0.00	<0.13	0.00	0.01
5	2	<0.04	0.00	0.00	<0.13	0.00	0.01
5	3	<0.04	0.00	0.00	<0.13	0.00	0.01
5	4	0.04	0.00	0.00	<0.13	0.00	0.01
5	5	0.07	0.01	0.01	<0.13	0.00	0.01
5	6	0.07	0.01	0.01	<0.13	0.00	0.01
5	7	0.10	0.01	0.01	<0.13	0.00	0.01
5	8	0.07	0.01	0.01	<0.13	0.00	0.01
			∑ min	∑ max		∑ min	∑ max
1			0.06	0.06		0.00	0.08
2			0.04	0.04		0.00	0.08
3			0.03	0.04		0.00	0.08
4			0.13	0.13		0.00	0.08
5			0.03	0.04		0.00	0.08
Mean			0.06	0.06		0.00	0.08
SD			0.04	0.04		0.00	0.00

Table E. Continued Granodiorite.

Stone	Fraction	LAB 1 Sn µg/l	min r mg/m^2	max r mg/m^2	LAB 2 Sn µg/l	min r mg/m^2	max r mg/m^2
1	1	0.062	0.005	0.005	<0.06	0.000	0.005
1	2	0.029	0.002	0.002	<0.06	0.000	0.005
1	3	0.085	0.007	0.007	<0.06	0.000	0.005
1	4	0.046	0.004	0.004	<0.06	0.000	0.005
1	5	0.063	0.005	0.005	<0.06	0.000	0.005
1	6	0.040	0.003	0.003	<0.06	0.000	0.005
1	7	0.033	0.003	0.003	<0.06	0.000	0.005
1	8	0.033	0.003	0.003	<0.06	0.000	0.005
2	1	0.103	0.008	0.008	<0.06	0.000	0.005
2	2	0.049	0.004	0.004	<0.06	0.000	0.005
2	3	0.313	0.025	0.025	<0.06	0.000	0.005
2	4	0.080	0.006	0.006	<0.06	0.000	0.005
2	5	0.128	0.010	0.010	<0.06	0.000	0.005
2	6	0.090	0.007	0.007	<0.06	0.000	0.005
2	7	0.074	0.006	0.006	<0.06	0.000	0.005
2	8	0.353	0.028	0.028	<0.06	0.000	0.005
3	1	0.080	0.006	0.006	<0.06	0.000	0.005
3	2	0.049	0.004	0.004	<0.06	0.000	0.005
3	3	0.370	0.030	0.030	<0.06	0.000	0.005
3	4	0.090	0.007	0.007	<0.06	0.000	0.005
3	5	0.195	0.016	0.016	<0.06	0.000	0.005
3	6	0.100	0.008	0.008	<0.06	0.000	0.005
3	7	0.110	0.009	0.009	<0.06	0.000	0.005
3	8	0.060	0.005	0.005	<0.06	0.000	0.005
4	1	0.054	0.004	0.004	<0.06	0.000	0.005
4	2	0.087	0.007	0.007	<0.06	0.000	0.005
4	3	0.119	0.010	0.010	<0.06	0.000	0.005
4	4	0.066	0.005	0.005	<0.06	0.000	0.005
4	5	0.272	0.022	0.022	<0.06	0.000	0.005
4	6	0.061	0.005	0.005	<0.06	0.000	0.005
4	7	0.061	0.005	0.005	<0.06	0.000	0.005
4	8	0.059	0.005	0.005	<0.06	0.000	0.005
5	1	0.064	0.005	0.005	<0.06	0.000	0.005
5	2	0.098	0.008	0.008	<0.06	0.000	0.005
5	3	0.236	0.019	0.019	<0.06	0.000	0.005
5	4	0.102	0.008	0.008	<0.06	0.000	0.005
5	5	0.093	0.007	0.007	<0.06	0.000	0.005
5	6	0.135	0.011	0.011	<0.06	0.000	0.005
5	7	0.158	0.013	0.013	<0.06	0.000	0.005
5	8	0.057	0.005	0.005	<0.06	0.000	0.005
			∑ min	∑ max		∑ min	∑ max
1			0.031	0.031		0.000	0.038
2			0.095	0.095		0.000	0.038
3			0.084	0.084		0.000	0.038
4			0.062	0.062		0.000	0.038
5			0.075	0.075		0.000	0.038
Mean			0.070	0.070		0.000	0.038
SD			0.025	0.025		0.000	0.000

Table E. Continued Granodiorite.

Stone	Fraction	LAB 1 Sr µg/l	min r mg/m^2	max r mg/m^2	LAB 2 Sr µg/l	min r mg/m^2	max r mg/m^2
1	1	8.50	0.680	0.680	<0.71	0.000	0.057
1	2	8.30	0.664	0.664	<0.71	0.000	0.057
1	3	7.10	0.568	0.568	<0.71	0.000	0.057
1	4	7.74	0.619	0.619	<0.71	0.000	0.057
1	5	8.08	0.646	0.646	0.77	0.062	0.062
1	6	3.69	0.295	0.295	0.73	0.058	0.058
1	7	8.24	0.659	0.659	1.42	0.114	0.114
1	8	6.83	0.546	0.546	1.16	0.093	0.093
2	1	8.65	0.692	0.692	<0.71	0.000	0.057
2	2	8.74	0.699	0.699	<0.71	0.000	0.057
2	3	8.03	0.643	0.643	<0.71	0.000	0.057
2	4	8.32	0.665	0.665	<0.71	0.000	0.057
2	5	9.18	0.734	0.734	<0.71	0.000	0.057
2	6	7.87	0.630	0.630	0.72	0.057	0.057
2	7	9.22	0.737	0.737	1.28	0.103	0.103
2	8	8.22	0.658	0.658	1.12	0.089	0.089
3	1	8.40	0.672	0.672	<0.71	0.000	0.057
3	2	8.03	0.643	0.643	<0.71	0.000	0.057
3	3	8.00	0.640	0.640	<0.71	0.000	0.057
3	4	7.82	0.626	0.626	<0.71	0.000	0.057
3	5	7.17	0.574	0.574	<0.71	0.000	0.057
3	6	6.76	0.541	0.541	<0.71	0.000	0.057
3	7	8.34	0.667	0.667	0.94	0.075	0.075
3	8	7.27	0.582	0.582	0.89	0.071	0.071
4	1	7.71	0.617	0.617	1.06	0.085	0.085
4	2	7.45	0.595	0.595	<0.71	0.000	0.057
4	3	7.81	0.625	0.625	<0.71	0.000	0.057
4	4	7.66	0.612	0.612	<0.71	0.000	0.057
4	5	7.84	0.627	0.627	0.81	0.065	0.065
4	6	7.68	0.614	0.614	0.73	0.058	0.058
4	7	10.36	0.829	0.829	1.44	0.115	0.115
4	8	12.25	0.980	0.980	1.14	0.091	0.091
5	1	8.50	0.680	0.680	<0.71	0.000	0.057
5	2	8.04	0.643	0.643	<0.71	0.000	0.057
5	3	6.97	0.558	0.558	<0.71	0.000	0.057
5	4	8.15	0.652	0.652	<0.71	0.000	0.057
5	5	8.36	0.669	0.669	0.99	0.079	0.079
5	6	8.36	0.669	0.669	1.01	0.081	0.081
5	7	9.01	0.721	0.721	2.17	0.174	0.174
5	8	7.19	0.575	0.575	2.18	0.174	0.174
			∑ min	∑ max		∑ min	∑ max
1			4.678	4.678		0.327	0.554
2			5.459	5.459		0.249	0.533
3			4.943	4.943		0.146	0.488
4			5.499	5.499		0.413	0.584
5			5.167	5.167		0.508	0.736
Mean			5.149	5.149		0.329	0.579
SD			0.347	0.347		0.141	0.094

Table E. Continued Granodiorite.

Stone	Fraction	LAB 1 U µg/l	min r mg/m²	max r mg/m²	LAB 2 U µg/l	min r mg/m²	max r mg/m²
1	1	0.071	0.006	0.006	0.081	0.006	0.006
1	2	0.061	0.005	0.005	0.045	0.004	0.004
1	3	0.050	0.004	0.004	0.046	0.004	0.004
1	4	0.058	0.005	0.005	0.049	0.004	0.004
1	5	0.088	0.007	0.007	0.097	0.008	0.008
1	6	0.139	0.011	0.011	0.112	0.009	0.009
1	7	0.097	0.008	0.008	0.284	0.023	0.023
1	8	0.091	0.007	0.007	0.237	0.019	0.019
2	1	0.083	0.007	0.007	0.061	0.005	0.005
2	2	0.054	0.004	0.004	0.034	0.003	0.003
2	3	0.055	0.004	0.004	<0.03	0.000	0.002
2	4	0.042	0.003	0.003	<0.03	0.000	0.002
2	5	0.059	0.005	0.005	0.049	0.004	0.004
2	6	0.096	0.008	0.008	0.051	0.004	0.004
2	7	0.089	0.007	0.007	0.122	0.010	0.010
2	8	0.109	0.009	0.009	0.126	0.010	0.010
3	1	0.114	0.009	0.009	0.050	0.004	0.004
3	2	0.111	0.009	0.009	<0.03	0.000	0.002
3	3	0.097	0.008	0.008	<0.03	0.000	0.002
3	4	0.081	0.006	0.006	<0.03	0.000	0.002
3	5	0.117	0.009	0.009	0.051	0.004	0.004
3	6	0.177	0.014	0.014	0.065	0.005	0.005
3	7	0.144	0.011	0.011	0.111	0.009	0.009
3	8	0.189	0.015	0.015	0.110	0.009	0.009
4	1	0.043	0.003	0.003	0.355	0.028	0.028
4	2	0.068	0.005	0.005	0.191	0.015	0.015
4	3	0.115	0.009	0.009	0.122	0.010	0.010
4	4	0.088	0.007	0.007	0.091	0.007	0.007
4	5	0.392	0.031	0.031	0.150	0.012	0.012
4	6	0.209	0.017	0.017	0.129	0.010	0.010
4	7	0.163	0.013	0.013	0.240	0.019	0.019
4	8	0.241	0.019	0.019	0.187	0.015	0.015
5	1	0.048	0.004	0.004	0.223	0.018	0.018
5	2	0.046	0.004	0.004	0.130	0.010	0.010
5	3	0.038	0.003	0.003	0.091	0.007	0.007
5	4	<0.04	0.0	0.003	0.063	0.005	0.005
5	5	0.047	0.004	0.004	0.119	0.010	0.010
5	6	0.074	0.006	0.006	0.092	0.007	0.007
5	7	0.057	0.005	0.005	0.190	0.015	0.015
5	8	0.078	0.006	0.006	0.158	0.013	0.013
			∑ min	∑ max		∑ min	∑ max
1			0.052	0.052		0.076	0.076
2			0.047	0.047		0.035	0.040
3			0.082	0.082		0.031	0.038
4			0.106	0.106		0.117	0.117
5			0.031	0.034		0.085	0.085
Mean			0.064	0.064		0.069	0.071
SD			0.030	0.029		0.036	0.033

Table E. Continued Granodiorite.

Stone	Fraction	LAB 1 V µg/l	min r mg/m²	max r mg/m²	LAB 2 V µg/l	min r mg/m²	max r mg/m²
1	1	0.081	0.007	0.007	0.064	0.005	0.005
1	2	0.074	0.006	0.006	0.038	0.003	0.003
1	3	0.062	0.005	0.005	<0.03	0.000	0.002
1	4	0.055	0.004	0.004	<0.03	0.000	0.002
1	5	0.080	0.006	0.006	<0.03	0.000	0.002
1	6	0.073	0.006	0.006	<0.03	0.000	0.002
1	7	0.078	0.006	0.006	0.042	0.000	0.003
1	8	0.065	0.005	0.005	0.034	0.000	0.003
2	1	0.066	0.005	0.005	0.045	0.004	0.004
2	2	0.049	0.004	0.004	0.038	0.003	0.003
2	3	0.043	0.003	0.003	<0.03	0.000	0.002
2	4	0.038	0.003	0.003	<0.03	0.000	0.002
2	5	0.058	0.005	0.005	<0.03	0.000	0.002
2	6	0.045	0.004	0.004	<0.03	0.000	0.002
2	7	0.075	0.006	0.006	0.053	0.000	0.004
2	8	0.072	0.006	0.006	0.036	0.000	0.003
3	1	0.049	0.004	0.004	0.132	0.011	0.011
3	2	0.046	0.004	0.004	0.111	0.009	0.009
3	3	0.035	0.003	0.003	0.032	0.003	0.003
3	4	0.029	0.002	0.002	<0.03	0.000	0.002
3	5	0.038	0.003	0.003	<0.03	0.000	0.002
3	6	0.029	0.002	0.002	<0.03	0.000	0.002
3	7	0.040	0.003	0.003	0.062	0.000	0.005
3	8	0.029	0.002	0.002	0.060	0.005	0.005
4	1	0.025	0.002	0.002	0.049	0.004	0.004
4	2	0.023	0.002	0.002	<0.03	0.000	0.002
4	3	<0.02	0.000	0.002	<0.03	0.000	0.002
4	4	<0.02	0.000	0.002	<0.03	0.000	0.002
4	5	0.032	0.003	0.003	<0.03	0.000	0.002
4	6	0.031	0.003	0.003	<0.03	0.000	0.002
4	7	0.040	0.003	0.003	<0.03	0.000	0.002
4	8	0.050	0.004	0.004	<0.03	0.000	0.002
5	1	0.059	0.005	0.005	0.063	0.005	0.005
5	2	0.056	0.004	0.004	0.045	0.000	0.004
5	3	0.046	0.004	0.004	0.038	0.000	0.003
5	4	0.034	0.003	0.003	0.033	0.003	0.003
5	5	0.050	0.004	0.004	0.047	0.004	0.004
5	6	0.040	0.003	0.003	0.040	0.000	0.003
5	7	0.055	0.004	0.004	0.056	0.000	0.004
5	8	0.050	0.004	0.004	0.040	0.000	0.003
			∑ min	∑ max		∑ min	∑ max
1			0.045	0.045		0.008	0.024
2			0.036	0.036		0.007	0.023
3			0.023	0.023		0.027	0.039
4			0.016	0.019		0.004	0.021
5			0.031	0.031		0.011	0.029
Mean			0.030	0.031		0.011	0.027
SD			0.011	0.010		0.009	0.007

Table E. Continued Granodiorite.

Stone	Fraction	LAB 1 Zn µg/l	min r mg/m^2	max r mg/m^2	LAB 2 Zn µg/l	min r mg/m^2	max r mg/m^2
1	1	462	36.98	36.98	0.95	0.076	0.076
1	2	468	37.48	37.48	0.54	0.043	0.043
1	3	240	19.18	19.18	0.67	0.053	0.053
1	4	281	22.47	22.47	0.41	0.032	0.032
1	5	261	20.91	20.91	0.29	0.024	0.024
1	6	249	19.89	19.89	0.24	0.019	0.019
1	7	387	30.96	30.96	0.27	0.021	0.021
1	8	254	20.35	20.35	0.27	0.021	0.021
2	1	422	33.74	33.74	0.63	0.051	0.051
2	2	311	24.89	24.89	0.57	0.046	0.046
2	3	436	34.88	34.88	0.54	0.043	0.043
2	4	319	25.50	25.50	0.32	0.026	0.026
2	5	465	37.22	37.22	0.28	0.022	0.022
2	6	311	24.85	24.85	0.41	0.033	0.033
2	7	419	33.51	33.51	0.29	0.023	0.023
2	8	282	22.58	22.58	0.42	0.033	0.033
3	1	276	22.05	22.05	0.77	0.061	0.061
3	2	456	36.47	36.47	0.40	0.032	0.032
3	3	452	36.13	36.13	0.76	0.061	0.061
3	4	262	20.97	20.97	1.17	0.093	0.093
3	5	225	17.98	17.98	0.73	0.058	0.058
3	6	255	20.36	20.36	0.97	0.078	0.078
3	7	293	23.40	23.40	0.89	0.071	0.071
3	8	251	20.05	20.05	1.02	0.082	0.082
4	1	437	34.93	34.93	5.64	0.451	0.451
4	2	440	35.19	35.19	0.78	0.063	0.063
4	3	412	32.94	32.94	0.87	0.070	0.070
4	4	413	33.04	33.04	0.43	0.034	0.034
4	5	264	21.08	21.08	0.36	0.029	0.029
4	6	289	23.14	23.14	0.40	0.032	0.032
4	7	304	24.28	24.28	0.64	0.051	0.051
4	8	690	55.19	55.19	0.46	0.037	0.037
5	1	305	24.38	24.38	0.54	0.043	0.043
5	2	299	23.93	23.93	0.31	0.025	0.025
5	3	225	18.03	18.03	0.34	0.027	0.027
5	4	297	23.79	23.79	0.18	0.014	0.014
5	5	303	24.27	24.27	0.15	0.012	0.012
5	6	444	35.56	35.56	0.24	0.019	0.019
5	7	295	23.57	23.57	0.15	0.012	0.012
5	8	276	22.05	22.05	0.23	0.019	0.019
			∑ min	∑ max		∑ min	∑ max
1			208.2	208.2		0.291	0.291
2			237.2	237.2		0.277	0.277
3			197.4	197.4		0.537	0.537
4			259.8	259.8		0.766	0.766
5			195.6	195.6		0.171	0.171
Mean			219.6	219.6		0.408	0.408
SD			27.95	27.95		0.241	0.241

Table F. DSLT results Granite (LAB 1 DSLT contract laboratory, LAB 2 DSLT BfG)

Stone	Fraction	LAB 1 Al µg/l	min r mg/m²	max r mg/m²	LAB 2 Al µg/l	min r mg/m²	max r mg/m²
1	1	<40.8	0.0	3.263	<6	0.00	0.48
1	2	<40.8	0.0	3.263	<6	0.00	0.48
1	3	<40.8	0.0	3.263	<6	0.00	0.48
1	4	<40.8	0.0	3.263	<6	0.00	0.48
1	5	<40.8	0.0	3.263	<6	0.00	0.48
1	6	<40.8	0.0	3.263	<6	0.00	0.48
1	7	<40.8	0.0	3.263	<6	0.00	0.48
1	8	<40.8	0.0	3.263	<6	0.00	0.48
2	1	<40.8	0.0	3.264	6.64	0.53	0.53
2	2	<40.8	0.0	3.264	10.65	0.85	0.85
2	3	<40.8	0.0	3.264	6.82	0.54	0.54
2	4	<40.8	0.0	3.264	7.63	0.61	0.61
2	5	<40.8	0.0	3.264	19.50	1.56	1.56
2	6	<40.8	0.0	3.264	14.64	1.17	1.17
2	7	<40.8	0.0	3.264	17.73	1.42	1.42
2	8	<40.8	0.0	3.264	12.57	1.00	1.00
3	1	<40.8	0.0	3.264	<6	0.00	0.48
3	2	<40.8	0.0	3.264	<6	0.00	0.48
3	3	<40.8	0.0	3.264	<6	0.00	0.48
3	4	<40.8	0.0	3.264	<6	0.00	0.48
3	5	<40.8	0.0	3.264	<6	0.00	0.48
3	6	<40.8	0.0	3.264	<6	0.00	0.48
3	7	<40.8	0.0	3.264	<6	0.00	0.48
3	8	<40.8	0.0	3.264	<6	0.00	0.48
4	1	<40.8	0.0	3.264	7.60	0.61	0.61
4	2	<40.8	0.0	3.264	10.23	0.82	0.82
4	3	<40.8	0.0	3.264	8.18	0.65	0.65
4	4	<40.8	0.0	3.264	8.56	0.68	0.68
4	5	<40.8	0.0	3.264	21.21	1.70	1.70
4	6	<40.8	0.0	3.264	15.76	1.26	1.26
4	7	<40.8	0.0	3.264	16.83	1.35	1.35
4	8	<40.8	0.0	3.264	14.65	1.17	1.17
5	1	<40.8	0.0	3.264	<6	0.00	0.48
5	2	<40.8	0.0	3.264	<6	0.00	0.48
5	3	<40.8	0.0	3.264	<6	0.00	0.48
5	4	<40.8	0.0	3.264	<6	0.00	0.48
5	5	<40.8	0.0	3.264	<6	0.00	0.48
5	6	<40.8	0.0	3.264	<6	0.00	0.48
5	7	<40.8	0.0	3.264	<6	0.00	0.48
5	8	<40.8	0.0	3.264	<6	0.00	0.48
			∑ min	∑ max		∑ min	∑ max
1			0.00	26.11		0.00	3.84
2			0.00	26.11		7.69	7.69
3			0.00	26.11		0.00	3.84
4			0.00	26.11		8.23	8.23
5			0.00	26.11		0.00	3.84
Mean			0.00	26.11		3.18	5.49
SD			0.00	0.00		4.37	2.27

Table F. Continued Granite.

Stone	Fraction	LAB 1 As µg/l	min r mg/m^2	max r mg/m^2	LAB 2 As µg/l	min r mg/m^2	max r mg/m^2
1	1	0.30	0.024	0.024	0.10	0.008	0.008
1	2	0.25	0.020	0.020	0.12	0.009	0.009
1	3	0.22	0.018	0.018	0.11	0.009	0.009
1	4	0.18	0.014	0.014	0.13	0.011	0.011
1	5	0.25	0.020	0.020	0.23	0.018	0.018
1	6	0.37	0.029	0.029	0.29	0.024	0.024
1	7	1.42	0.113	0.113	0.42	0.034	0.034
1	8	0.77	0.061	0.061	0.43	0.035	0.035
2	1	0.21	0.017	0.017	0.16	0.013	0.013
2	2	0.23	0.018	0.018	0.18	0.014	0.014
2	3	0.33	0.026	0.026	0.16	0.012	0.012
2	4	0.30	0.024	0.024	0.15	0.012	0.012
2	5	0.58	0.046	0.046	0.25	0.020	0.020
2	6	0.74	0.059	0.059	0.24	0.019	0.019
2	7	2.42	0.194	0.194	0.26	0.021	0.021
2	8	2.22	0.178	0.178	0.22	0.017	0.017
3	1	0.16	0.013	0.013	0.06	0.005	0.005
3	2	0.14	0.011	0.011	0.06	0.004	0.004
3	3	0.15	0.012	0.012	0.06	0.004	0.004
3	4	0.14	0.011	0.011	0.07	0.005	0.005
3	5	0.22	0.018	0.018	0.09	0.007	0.007
3	6	0.27	0.022	0.022	0.10	0.008	0.008
3	7	0.77	0.062	0.062	0.16	0.013	0.013
3	8	0.36	0.029	0.029	0.23	0.019	0.019
4	1	0.18	0.015	0.015	0.39	0.031	0.031
4	2	0.14	0.011	0.011	0.86	0.069	0.069
4	3	0.13	0.010	0.010	0.81	0.065	0.065
4	4	0.12	0.010	0.010	0.87	0.070	0.070
4	5	0.22	0.017	0.017	1.40	0.112	0.112
4	6	0.30	0.024	0.024	1.47	0.118	0.118
4	7	1.10	0.088	0.088	1.89	0.151	0.151
4	8	0.59	0.047	0.047	1.74	0.139	0.139
5	1	0.19	0.015	0.015	1.27	0.101	0.101
5	2	0.13	0.010	0.010	0.81	0.064	0.064
5	3	0.14	0.011	0.011	0.69	0.055	0.055
5	4	0.10	0.008	0.008	0.82	0.066	0.066
5	5	0.15	0.012	0.012	1.20	0.096	0.096
5	6	0.19	0.015	0.015	1.41	0.113	0.113
5	7	0.51	0.040	0.040	1.83	0.146	0.146
5	8	0.44	0.035	0.035	2.15	0.172	0.172
			∑ min	∑ max		∑ min	∑ max
1			0.30	0.30		0.147	0.147
2			0.56	0.56		0.129	0.129
3			0.18	0.18		0.066	0.066
4			0.22	0.22		0.754	0.754
5			0.15	0.15		0.814	0.814
Mean			0.282	0.282		0.38	0.38
SD			0.167	0.167		0.37	0.37

Table F. Continued Granite.

Stone	Fraction	LAB 1 Ba µg/l	min r mg/m^2	max r mg/m^2	LAB 2 Ba µg/l	min r mg/m^2	max r mg/m^2
1	1	350	28.02	28.02	0.38	0.031	0.031
1	2	429	34.29	34.29	0.54	0.043	0.043
1	3	482	38.51	38.51	0.15	0.012	0.012
1	4	434	34.70	34.70	0.12	0.009	0.009
1	5	447	35.76	35.76	0.28	0.022	0.022
1	6	443	35.42	35.42	0.17	0.013	0.013
1	7	355	28.43	28.43	0.20	0.016	0.016
1	8	430	34.38	34.38	0.13	0.011	0.011
2	1	431	34.46	34.46	0.26	0.021	0.021
2	2	415	33.16	33.16	0.77	0.062	0.062
2	3	436	34.86	34.86	0.16	0.013	0.013
2	4	365	29.20	29.20	0.16	0.013	0.013
2	5	473	37.81	37.81	0.31	0.025	0.025
2	6	412	32.97	32.97	0.29	0.023	0.023
2	7	366	29.30	29.30	0.36	0.029	0.029
2	8	394	31.55	31.55	0.29	0.023	0.023
3	1	407	32.57	32.57	0.29	0.023	0.023
3	2	411	32.85	32.85	0.73	0.059	0.059
3	3	403	32.20	32.20	0.12	0.010	0.010
3	4	403	32.21	32.21	0.10	0.008	0.008
3	5	429	34.34	34.34	0.25	0.020	0.020
3	6	434	34.74	34.74	0.12	0.010	0.010
3	7	400	31.97	31.97	0.14	0.011	0.011
3	8	381	30.50	30.50	0.11	0.009	0.009
4	1	392	31.39	31.39	0.27	0.022	0.022
4	2	351	28.09	28.09	0.44	0.035	0.035
4	3	114	9.09	9.09	0.18	0.015	0.015
4	4	90	7.22	7.22	0.22	0.018	0.018
4	5	82	6.57	6.57	0.46	0.037	0.037
4	6	455	36.37	36.37	0.28	0.023	0.023
4	7	387	30.93	30.93	0.40	0.032	0.032
4	8	255	20.40	20.40	0.32	0.025	0.025
5	1	330	26.40	26.40	0.18	0.014	0.014
5	2	382	30.57	30.57	0.52	0.042	0.042
5	3	406	32.50	32.50	0.11	0.009	0.009
5	4	172	13.77	13.77	0.11	0.008	0.008
5	5	94	7.52	7.52	0.21	0.017	0.017
5	6	110	8.82	8.82	0.12	0.010	0.010
5	7	368	29.40	29.40	0.16	0.013	0.013
5	8	381	30.47	30.47	0.06	0.005	0.005
			∑ min	∑ max		∑ min	∑ max
1			270	270		0.158	0.158
2			263	263		0.208	0.208
3			261	261		0.150	0.150
4			170	170		0.206	0.206
5			179	179		0.118	0.118
Mean			229	229		0.168	0.168
SD			49.5	49.5		0.039	0.039

Table F. Continued Granite.

Stone	Fraction	LAB 1 Cd µg/l	min r mg/m^2	max r mg/m^2	LAB 2 Cd µg/l	min r mg/m^2	max r mg/m^2
1	1	0.02	0.002	0.002	<0.01	0.000	0.001
1	2	0.02	0.002	0.002	<0.01	0.000	0.001
1	3	0.03	0.002	0.002	<0.01	0.000	0.001
1	4	0.03	0.002	0.002	<0.01	0.000	0.001
1	5	0.03	0.003	0.003	<0.01	0.000	0.001
1	6	0.03	0.003	0.003	<0.01	0.000	0.001
1	7	0.03	0.002	0.002	<0.01	0.000	0.001
1	8	0.04	0.003	0.003	<0.01	0.000	0.001
2	1	0.02	0.002	0.002	<0.01	0.000	0.001
2	2	0.02	0.002	0.002	<0.01	0.000	0.001
2	3	0.02	0.002	0.002	<0.01	0.000	0.001
2	4	0.03	0.002	0.002	<0.01	0.000	0.001
2	5	0.03	0.003	0.003	<0.01	0.000	0.001
2	6	0.03	0.002	0.002	<0.01	0.000	0.001
2	7	0.03	0.003	0.003	<0.01	0.000	0.001
2	8	0.03	0.002	0.002	<0.01	0.000	0.001
3	1	0.03	0.002	0.002	<0.01	0.000	0.001
3	2	0.02	0.002	0.002	<0.01	0.000	0.001
3	3	0.03	0.002	0.002	<0.01	0.000	0.001
3	4	0.03	0.002	0.002	<0.01	0.000	0.001
3	5	0.03	0.002	0.002	<0.01	0.000	0.001
3	6	0.03	0.003	0.003	<0.01	0.000	0.001
3	7	0.03	0.002	0.002	<0.01	0.000	0.001
3	8	0.03	0.002	0.002	<0.01	0.000	0.001
4	1	0.02	0.002	0.002	<0.01	0.000	0.001
4	2	0.03	0.002	0.002	<0.01	0.000	0.001
4	3	0.01	0.001	0.001	<0.01	0.000	0.001
4	4	0.01	0.001	0.001	<0.01	0.000	0.001
4	5	0.01	0.001	0.001	<0.01	0.000	0.001
4	6	0.03	0.002	0.002	<0.01	0.000	0.001
4	7	0.03	0.002	0.002	<0.01	0.000	0.001
4	8	0.02	0.001	0.001	<0.01	0.000	0.001
5	1	0.03	0.002	0.002	<0.01	0.000	0.001
5	2	0.03	0.002	0.002	<0.01	0.000	0.001
5	3	0.03	0.002	0.002	<0.01	0.000	0.001
5	4	0.02	0.001	0.001	<0.01	0.000	0.001
5	5	0.01	0.001	0.001	<0.01	0.000	0.001
5	6	0.02	0.001	0.001	<0.01	0.000	0.001
5	7	0.03	0.002	0.002	<0.01	0.000	0.001
5	8	0.03	0.002	0.002	<0.01	0.000	0.001
			∑ min	∑ max		∑ min	∑ max
1			0.018	0.018		0.000	0.006
2			0.017	0.017		0.000	0.006
3			0.018	0.018		0.000	0.006
4			0.012	0.012		0.000	0.006
5			0.015	0.015		0.000	0.006
Mean			0.016	0.016		0.000	0.006
SD			0.002	0.002		0.000	0.000

Table F. Continued Granite.

Stone	Fraction	LAB 1 Co µg/l	min r mg/m²	max r mg/m²	LAB 2 Co µg/l	min r mg/m²	max r mg/m²
1	1	0.07	0.006	0.006	<0.01	0.000	0.001
1	2	0.06	0.005	0.005	<0.01	0.000	0.001
1	3	0.07	0.006	0.006	<0.01	0.000	0.001
1	4	0.07	0.006	0.006	<0.01	0.000	0.001
1	5	0.07	0.006	0.006	<0.01	0.000	0.001
1	6	0.07	0.006	0.006	<0.01	0.000	0.001
1	7	0.11	0.009	0.009	<0.01	0.000	0.001
1	8	0.12	0.010	0.010	<0.01	0.000	0.001
2	1	0.07	0.006	0.006	<0.01	0.000	0.001
2	2	0.06	0.005	0.005	<0.01	0.000	0.001
2	3	0.07	0.005	0.005	<0.01	0.000	0.001
2	4	0.07	0.005	0.005	<0.01	0.000	0.001
2	5	0.08	0.006	0.006	<0.01	0.000	0.001
2	6	0.07	0.006	0.006	<0.01	0.000	0.001
2	7	0.12	0.010	0.010	<0.01	0.000	0.001
2	8	0.12	0.010	0.010	<0.01	0.000	0.001
3	1	0.07	0.006	0.006	<0.01	0.000	0.001
3	2	0.07	0.005	0.005	<0.01	0.000	0.001
3	3	0.07	0.006	0.006	<0.01	0.000	0.001
3	4	0.07	0.006	0.006	<0.01	0.000	0.001
3	5	0.08	0.006	0.006	<0.01	0.000	0.001
3	6	0.07	0.006	0.006	<0.01	0.000	0.001
3	7	0.12	0.009	0.009	<0.01	0.000	0.001
3	8	0.11	0.008	0.008	<0.01	0.000	0.001
4	1	0.07	0.006	0.006	<0.01	0.000	0.001
4	2	0.07	0.006	0.006	<0.01	0.000	0.001
4	3	0.03	0.003	0.003	<0.01	0.000	0.001
4	4	0.05	0.004	0.004	<0.01	0.000	0.001
4	5	0.03	0.003	0.003	<0.01	0.000	0.001
4	6	0.08	0.006	0.006	<0.01	0.000	0.001
4	7	0.12	0.010	0.010	<0.01	0.000	0.001
4	8	0.08	0.006	0.006	<0.01	0.000	0.001
5	1	0.07	0.006	0.006	<0.01	0.000	0.001
5	2	0.07	0.006	0.006	<0.01	0.000	0.001
5	3	0.08	0.006	0.006	<0.01	0.000	0.001
5	4	0.05	0.004	0.004	<0.01	0.000	0.001
5	5	0.04	0.003	0.003	<0.01	0.000	0.001
5	6	0.05	0.004	0.004	<0.01	0.000	0.001
5	7	0.13	0.010	0.010	<0.01	0.000	0.001
5	8	0.11	0.009	0.009	<0.01	0.000	0.001
			∑ min	∑ max		∑ min	∑ max
1			0.05	0.05		0.000	0.006
2			0.05	0.05		0.000	0.006
3			0.05	0.05		0.000	0.006
4			0.04	0.04		0.000	0.006
5			0.05	0.05		0.000	0.006
Mean			0.050	0.050		0.000	0.006
SD			0.004	0.004		0.000	0.000

Table F. Continued Granite.

Stone	Fraction	LAB 1 Cr µg/l	min r mg/m²	max r mg/m²	LAB 2 Cr µg/l	min r mg/m²	max r mg/m²
1	1	0.16	0.013	0.013	0.03	0.002	0.002
1	2	0.09	0.008	0.008	0.01	0.001	0.001
1	3	0.09	0.007	0.007	<0.01	0.000	0.001
1	4	0.06	0.005	0.005	<0.01	0.000	0.001
1	5	0.14	0.011	0.011	0.03	0.003	0.003
1	6	0.07	0.006	0.006	0.01	0.001	0.001
1	7	<0.06	0.000	0.005	0.02	0.002	0.002
1	8	0.42	0.034	0.034	0.01	0.001	0.001
2	1	0.15	0.012	0.012	0.01	0.001	0.001
2	2	0.07	0.006	0.006	0.01	0.001	0.001
2	3	0.09	0.007	0.007	0.06	0.005	0.005
2	4	0.09	0.007	0.007	<0.01	0.000	0.001
2	5	0.07	0.006	0.006	<0.01	0.000	0.001
2	6	0.12	0.010	0.010	0.01	0.001	0.001
2	7	0.06	0.005	0.005	0.01	0.001	0.001
2	8	0.13	0.010	0.010	0.01	0.001	0.001
3	1	0.22	0.017	0.017	0.01	0.001	0.001
3	2	0.09	0.007	0.007	<0.01	0.000	0.001
3	3	0.09	0.007	0.007	<0.01	0.000	0.001
3	4	0.06	0.005	0.005	<0.01	0.000	0.001
3	5	0.07	0.005	0.005	<0.01	0.000	0.001
3	6	0.07	0.005	0.005	<0.01	0.000	0.001
3	7	<0.06	0.000	0.005	0.01	0.001	0.001
3	8	<0.06	0.000	0.005	<0.01	0.000	0.001
4	1	0.42	0.033	0.033	0.01	0.001	0.001
4	2	0.09	0.007	0.007	<0.01	0.000	0.001
4	3	<0.06	0.000	0.005	<0.01	0.000	0.001
4	4	<0.06	0.000	0.005	<0.01	0.000	0.001
4	5	<0.06	0.000	0.005	0.01	0.001	0.001
4	6	0.07	0.006	0.006	0.01	0.001	0.001
4	7	<0.06	0.000	0.005	0.02	0.002	0.002
4	8	0.06	0.005	0.005	0.02	0.001	0.001
5	1	0.24	0.019	0.019	0.02	0.002	0.002
5	2	0.10	0.008	0.008	0.02	0.002	0.002
5	3	0.09	0.008	0.008	<0.01	0.000	0.001
5	4	0.05	0.004	0.004	<0.01	0.000	0.001
5	5	0.07	0.006	0.006	<0.01	0.000	0.001
5	6	<0.06	0.000	0.005	<0.01	0.000	0.001
5	7	0.06	0.005	0.005	0.02	0.001	0.001
5	8	0.46	0.037	0.037	0.01	0.001	0.001
			∑ min	∑ max		∑ min	∑ max
1			0.083	0.088		0.009	0.011
2			0.063	0.063		0.010	0.011
3			0.048	0.057		0.002	0.007
4			0.051	0.070		0.006	0.009
5			0.086	0.091		0.006	0.009
Mean			0.066	0.074		0.006	0.009
SD			0.018	0.015		0.003	0.002

Table F. Continued Granite.

Stone	Fraction	LAB 1 Cu µg/l	min r mg/m²	max r mg/m²	LAB 2 Cu µg/l	min r mg/m²	max r mg/m²
1	1	6.20	0.496	0.496	0.32	0.026	0.026
1	2	2.40	0.192	0.192	<0.03	0.000	0.002
1	3	2.62	0.210	0.210	<0.03	0.000	0.002
1	4	2.41	0.193	0.193	<0.03	0.000	0.002
1	5	2.86	0.229	0.229	<0.03	0.000	0.002
1	6	2.12	0.170	0.170	<0.03	0.000	0.002
1	7	1.91	0.153	0.153	<0.03	0.000	0.002
1	8	2.17	0.173	0.173	<0.03	0.000	0.002
2	1	3.21	0.257	0.257	0.03	0.002	0.002
2	2	3.22	0.258	0.258	<0.03	0.000	0.002
2	3	2.37	0.190	0.190	<0.03	0.000	0.002
2	4	2.27	0.182	0.182	<0.03	0.000	0.002
2	5	2.26	0.181	0.181	<0.03	0.000	0.002
2	6	2.24	0.179	0.179	<0.03	0.000	0.002
2	7	1.77	0.141	0.141	<0.03	0.000	0.002
2	8	2.01	0.161	0.161	<0.03	0.000	0.002
3	1	7.53	0.603	0.603	0.03	0.003	0.003
3	2	2.96	0.237	0.237	<0.03	0.000	0.002
3	3	2.35	0.188	0.188	<0.03	0.000	0.002
3	4	1.49	0.119	0.119	<0.03	0.000	0.002
3	5	2.30	0.184	0.184	<0.03	0.000	0.002
3	6	2.18	0.174	0.174	<0.03	0.000	0.002
3	7	1.49	0.119	0.119	<0.03	0.000	0.002
3	8	1.43	0.114	0.114	<0.03	0.000	0.002
4	1	14.06	1.125	1.125	0.05	0.004	0.004
4	2	2.91	0.233	0.233	<0.03	0.000	0.002
4	3	1.92	0.154	0.154	<0.03	0.000	0.002
4	4	2.01	0.161	0.161	<0.03	0.000	0.002
4	5	1.71	0.137	0.137	0.03	0.003	0.003
4	6	2.33	0.187	0.187	<0.03	0.000	0.002
4	7	1.46	0.117	0.117	<0.03	0.000	0.002
4	8	1.50	0.120	0.120	<0.03	0.000	0.002
5	1	7.06	0.564	0.564	0.04	0.003	0.003
5	2	4.07	0.326	0.326	<0.03	0.000	0.002
5	3	3.58	0.287	0.287	<0.03	0.000	0.002
5	4	2.35	0.188	0.188	<0.03	0.000	0.002
5	5	3.05	0.244	0.244	<0.03	0.000	0.002
5	6	2.27	0.181	0.181	<0.03	0.000	0.002
5	7	2.34	0.187	0.187	0.08	0.007	0.007
5	8	2.41	0.192	0.192	<0.03	0.000	0.002
			∑ min	∑ max		∑ min	∑ max
1			1.816	1.816		0.026	0.043
2			1.549	1.549		0.002	0.019
3			1.738	1.738		0.003	0.019
4			2.233	2.233		0.007	0.021
5			2.170	2.170		0.010	0.024
Mean			1.901	1.901		0.010	0.025
SD			0.292	0.292		0.010	0.010

Table F. Continued Granite.

Stone	Fraction	LAB 1 Mn µg/l	min r mg/m²	max r mg/m²	LAB 2 Mn µg/l	min r mg/m²	max r mg/m²
1	1	1.977	0.158	0.158	1.78	0.143	0.143
1	2	1.036	0.083	0.083	0.69	0.055	0.055
1	3	1.147	0.092	0.092	0.42	0.033	0.033
1	4	1.281	0.102	0.102	0.37	0.029	0.029
1	5	2.014	0.161	0.161	0.50	0.040	0.040
1	6	2.425	0.194	0.194	0.57	0.046	0.046
1	7	8.181	0.654	0.654	0.66	0.053	0.053
1	8	7.642	0.611	0.611	0.53	0.043	0.043
2	1	0.976	0.078	0.078	2.22	0.177	0.177
2	2	0.620	0.050	0.050	2.03	0.163	0.163
2	3	0.837	0.067	0.067	1.27	0.101	0.101
2	4	0.628	0.050	0.050	0.93	0.074	0.074
2	5	0.996	0.080	0.080	0.22	0.018	0.018
2	6	1.159	0.093	0.093	0.40	0.032	0.032
2	7	3.109	0.249	0.249	0.42	0.034	0.034
2	8	2.996	0.240	0.240	0.41	0.033	0.033
3	1	1.427	0.114	0.114	1.09	0.087	0.087
3	2	1.248	0.100	0.100	1.02	0.081	0.081
3	3	1.065	0.085	0.085	0.79	0.063	0.063
3	4	1.272	0.102	0.102	0.71	0.057	0.057
3	5	1.813	0.145	0.145	1.02	0.082	0.082
3	6	2.020	0.162	0.162	0.86	0.069	0.069
3	7	12.251	0.980	0.980	0.94	0.075	0.075
3	8	4.461	0.357	0.357	0.75	0.060	0.060
4	1	1.986	0.159	0.159	1.81	0.145	0.145
4	2	0.438	0.035	0.035	1.69	0.135	0.135
4	3	0.433	0.035	0.035	1.59	0.127	0.127
4	4	0.576	0.046	0.046	1.46	0.116	0.116
4	5	0.602	0.048	0.048	0.78	0.063	0.063
4	6	0.992	0.079	0.079	0.93	0.074	0.074
4	7	2.786	0.223	0.223	1.28	0.102	0.102
4	8	2.031	0.162	0.162	1.43	0.114	0.114
5	1	2.153	0.172	0.172	1.28	0.102	0.102
5	2	1.336	0.107	0.107	1.06	0.085	0.085
5	3	1.503	0.120	0.120	0.79	0.063	0.063
5	4	0.737	0.059	0.059	0.94	0.075	0.075
5	5	1.948	0.156	0.156	1.09	0.087	0.087
5	6	1.830	0.146	0.146	1.01	0.081	0.081
5	7	6.950	0.556	0.556	0.94	0.075	0.075
5	8	6.677	0.534	0.534	0.82	0.065	0.065
			∑ min	∑ max		∑ min	∑ max
1			2.056	2.056		0.442	0.442
2			0.906	0.906		0.632	0.632
3			2.045	2.045		0.575	0.575
4			0.787	0.787		0.877	0.877
5			1.850	1.850		0.633	0.633
Mean			1.529	1.529		0.632	0.632
SD			0.629	0.629		0.158	0.158

Table F. Continued Granite.

Stone	Fraction	LAB 1 Mo µg/l	min r mg/m²	max r mg/m²	LAB 2 Mo µg/l	min r mg/m²	max r mg/m²
1	1	0.07	0.006	0.006	<0.05	0.000	0.004
1	2	<0.06	0.000	0.005	<0.05	0.000	0.004
1	3	<0.06	0.000	0.005	<0.05	0.000	0.004
1	4	0.06	0.005	0.005	<0.05	0.000	0.004
1	5	<0.06	0.000	0.005	<0.05	0.000	0.004
1	6	<0.06	0.000	0.005	<0.05	0.000	0.004
1	7	<0.06	0.000	0.005	<0.05	0.000	0.004
1	8	<0.06	0.000	0.005	<0.05	0.000	0.004
2	1	<0.06	0.000	0.005	<0.05	0.000	0.004
2	2	<0.06	0.000	0.005	<0.05	0.000	0.004
2	3	<0.06	0.000	0.005	<0.05	0.000	0.004
2	4	<0.06	0.000	0.005	<0.05	0.000	0.004
2	5	<0.06	0.000	0.005	<0.05	0.000	0.004
2	6	<0.06	0.000	0.005	<0.05	0.000	0.004
2	7	<0.06	0.000	0.005	<0.05	0.000	0.004
2	8	<0.06	0.000	0.005	<0.05	0.000	0.004
3	1	<0.06	0.000	0.005	0.06	0.005	0.005
3	2	<0.06	0.000	0.005	<0.05	0.000	0.004
3	3	<0.06	0.000	0.005	<0.05	0.000	0.004
3	4	<0.06	0.000	0.005	<0.05	0.000	0.004
3	5	<0.06	0.000	0.005	<0.05	0.000	0.004
3	6	<0.06	0.000	0.005	<0.05	0.000	0.004
3	7	<0.06	0.000	0.005	<0.05	0.000	0.004
3	8	<0.06	0.000	0.005	<0.05	0.000	0.004
4	1	<0.06	0.000	0.005	<0.05	0.000	0.004
4	2	<0.06	0.000	0.005	<0.05	0.000	0.004
4	3	<0.06	0.000	0.005	<0.05	0.000	0.004
4	4	<0.06	0.000	0.005	<0.05	0.000	0.004
4	5	<0.06	0.000	0.005	<0.05	0.000	0.004
4	6	<0.06	0.000	0.005	<0.05	0.000	0.004
4	7	0.08	0.006	0.006	<0.05	0.000	0.004
4	8	0.07	0.005	0.005	<0.05	0.000	0.004
5	1	0.04	0.003	0.003	<0.05	0.000	0.004
5	2	0.04	0.003	0.003	<0.05	0.000	0.004
5	3	0.04	0.003	0.003	<0.05	0.000	0.004
5	4	0.11	0.008	0.008	<0.05	0.000	0.004
5	5	0.04	0.003	0.003	<0.05	0.000	0.004
5	6	0.04	0.004	0.004	<0.05	0.000	0.004
5	7	0.09	0.007	0.007	<0.05	0.000	0.004
5	8	0.07	0.006	0.006	<0.05	0.000	0.004
			∑ min	∑ max		∑ min	∑ max
1			0.010	0.039		0.000	0.032
2			0.000	0.038		0.000	0.032
3			0.000	0.038		0.005	0.033
4			0.012	0.040		0.000	0.032
5			0.037	0.037		0.000	0.032
Mean			0.012	0.039		0.001	0.032
SD			0.015	0.001		0.002	0.000

Table F. Continued Granite.

Stone	Fraction	LAB 1 Ni µg/l	min r mg/m^2	max r mg/m^2	LAB 2 Ni µg/l	min r mg/m^2	max r mg/m^2
1	1	0.67	0.054	0.054	0.17	0.014	0.014
1	2	0.31	0.025	0.025	0.07	0.006	0.006
1	3	0.32	0.026	0.026	<0.02	0.000	0.002
1	4	0.26	0.021	0.021	<0.02	0.000	0.002
1	5	0.39	0.031	0.031	<0.02	0.000	0.002
1	6	0.22	0.017	0.017	0.03	0.002	0.002
1	7	0.42	0.034	0.034	0.03	0.002	0.002
1	8	0.26	0.021	0.021	0.03	0.002	0.002
2	1	0.43	0.035	0.035	0.05	0.004	0.004
2	2	0.28	0.023	0.023	0.02	0.002	0.002
2	3	0.28	0.023	0.023	0.02	0.002	0.002
2	4	0.27	0.022	0.022	0.02	0.002	0.002
2	5	0.30	0.024	0.024	0.02	0.002	0.002
2	6	0.34	0.027	0.027	0.02	0.002	0.002
2	7	0.52	0.041	0.041	<0.02	0.000	0.002
2	8	0.22	0.017	0.017	<0.02	0.000	0.002
3	1	0.59	0.047	0.047	0.03	0.003	0.003
3	2	0.33	0.026	0.026	0.03	0.002	0.002
3	3	0.28	0.022	0.022	<0.02	0.000	0.002
3	4	0.24	0.019	0.019	<0.02	0.000	0.002
3	5	0.34	0.028	0.028	<0.02	0.000	0.002
3	6	0.25	0.020	0.020	0.03	0.003	0.003
3	7	0.27	0.022	0.022	0.02	0.002	0.002
3	8	0.18	0.015	0.015	<0.02	0.000	0.002
4	1	1.01	0.081	0.081	0.04	0.003	0.003
4	2	0.29	0.023	0.023	<0.02	0.000	0.002
4	3	0.15	0.012	0.012	<0.02	0.000	0.002
4	4	0.16	0.013	0.013	<0.02	0.000	0.002
4	5	0.17	0.013	0.013	<0.02	0.000	0.002
4	6	0.26	0.021	0.021	0.02	0.002	0.002
4	7	0.27	0.022	0.022	0.02	0.002	0.002
4	8	0.17	0.014	0.014	<0.02	0.000	0.002
5	1	0.79	0.064	0.064	0.13	0.011	0.011
5	2	0.46	0.037	0.037	0.04	0.003	0.003
5	3	0.50	0.040	0.040	0.03	0.002	0.002
5	4	0.21	0.017	0.017	0.03	0.002	0.002
5	5	0.36	0.029	0.029	0.03	0.002	0.002
5	6	0.21	0.017	0.017	0.03	0.002	0.002
5	7	0.76	0.060	0.060	0.03	0.003	0.003
5	8	0.39	0.031	0.031	0.03	0.002	0.002
			∑ min	∑ max		∑ min	∑ max
1			0.228	0.228		0.026	0.031
2			0.212	0.212		0.013	0.016
3			0.197	0.197		0.009	0.015
4			0.199	0.199		0.007	0.015
5			0.294	0.294		0.028	0.028
Mean			0.226	0.226		0.016	0.021
SD			0.040	0.040		0.010	0.008

Table F. Continued Granite.

Stone	Fraction	LAB 1 Pb µg/l	min r mg/m²	max r mg/m²	LAB 2 Pb µg/l	min r mg/m²	max r mg/m²
1	1	0.12	0.009	0.009	<0.01	0.000	0.001
1	2	0.12	0.009	0.009	0.01	0.001	0.001
1	3	0.23	0.018	0.018	<0.01	0.000	0.001
1	4	0.19	0.015	0.015	<0.01	0.000	0.001
1	5	0.61	0.049	0.049	<0.01	0.000	0.001
1	6	0.31	0.025	0.025	<0.01	0.000	0.001
1	7	0.08	0.006	0.006	<0.01	0.000	0.001
1	8	0.18	0.014	0.014	<0.01	0.000	0.001
2	1	0.15	0.012	0.012	<0.01	0.000	0.001
2	2	0.12	0.010	0.010	<0.01	0.000	0.001
2	3	0.18	0.014	0.014	<0.01	0.000	0.001
2	4	0.26	0.021	0.021	<0.01	0.000	0.001
2	5	0.26	0.021	0.021	<0.01	0.000	0.001
2	6	0.30	0.024	0.024	<0.01	0.000	0.001
2	7	0.08	0.007	0.007	<0.01	0.000	0.001
2	8	0.10	0.008	0.008	<0.01	0.000	0.001
3	1	0.10	0.008	0.008	0.02	0.002	0.002
3	2	0.08	0.007	0.007	0.02	0.002	0.002
3	3	0.12	0.009	0.009	<0.01	0.000	0.001
3	4	0.66	0.052	0.052	<0.01	0.000	0.001
3	5	0.15	0.012	0.012	<0.01	0.000	0.001
3	6	0.18	0.014	0.014	<0.01	0.000	0.001
3	7	0.06	0.005	0.005	<0.01	0.000	0.001
3	8	0.04	0.003	0.003	<0.01	0.000	0.001
4	1	0.13	0.011	0.011	0.02	0.002	0.002
4	2	0.09	0.007	0.007	<0.01	0.000	0.001
4	3	0.11	0.009	0.009	<0.01	0.000	0.001
4	4	0.10	0.008	0.008	<0.01	0.000	0.001
4	5	0.14	0.011	0.011	0.02	0.001	0.001
4	6	0.16	0.013	0.013	<0.01	0.000	0.001
4	7	0.07	0.006	0.006	<0.01	0.000	0.001
4	8	0.06	0.005	0.005	<0.01	0.000	0.001
5	1	0.43	0.035	0.035	<0.01	0.000	0.001
5	2	0.12	0.010	0.010	<0.01	0.000	0.001
5	3	0.21	0.017	0.017	<0.01	0.000	0.001
5	4	0.12	0.010	0.010	<0.01	0.000	0.001
5	5	0.54	0.043	0.043	<0.01	0.000	0.001
5	6	0.23	0.018	0.018	<0.01	0.000	0.001
5	7	0.10	0.008	0.008	<0.01	0.000	0.001
5	8	0.12	0.010	0.010	<0.01	0.000	0.001
			∑ min	∑ max		∑ min	∑ max
1			0.146	0.146		0.001	0.007
2			0.116	0.116		0.000	0.006
3			0.111	0.111		0.003	0.008
4			0.069	0.069		0.003	0.008
5			0.151	0.151		0.000	0.006
Mean			0.119	0.119		0.002	0.007
SD			0.033	0.033		0.002	0.001

Table F. Continued Granite.

Stone	Fraction	LAB 1 Sb µg/l	min r mg/m^2	max r mg/m^2	LAB 2 Sb µg/l	min r mg/m^2	max r mg/m^2
1	1	0.09	0.007	0.007	0.03	0.002	0.002
1	2	0.06	0.005	0.005	<0.02	0.000	0.002
1	3	0.08	0.006	0.006	<0.02	0.000	0.002
1	4	0.07	0.006	0.006	<0.02	0.000	0.002
1	5	0.08	0.006	0.006	<0.02	0.000	0.002
1	6	0.07	0.005	0.005	<0.02	0.000	0.002
1	7	0.23	0.018	0.018	<0.02	0.000	0.002
1	8	0.26	0.021	0.021	<0.02	0.000	0.002
2	1	0.09	0.007	0.007	<0.02	0.000	0.002
2	2	0.08	0.006	0.006	<0.02	0.000	0.002
2	3	0.08	0.006	0.006	<0.02	0.000	0.002
2	4	0.07	0.006	0.006	<0.02	0.000	0.002
2	5	0.07	0.006	0.006	<0.02	0.000	0.002
2	6	0.08	0.007	0.007	<0.02	0.000	0.002
2	7	0.29	0.023	0.023	<0.02	0.000	0.002
2	8	0.28	0.023	0.023	<0.02	0.000	0.002
3	1	0.08	0.006	0.006	<0.02	0.000	0.002
3	2	0.09	0.007	0.007	<0.02	0.000	0.002
3	3	0.08	0.007	0.007	<0.02	0.000	0.002
3	4	0.07	0.006	0.006	<0.02	0.000	0.002
3	5	0.09	0.007	0.007	<0.02	0.000	0.002
3	6	0.09	0.007	0.007	<0.02	0.000	0.002
3	7	0.28	0.023	0.023	0.03	0.002	0.002
3	8	0.24	0.019	0.019	0.03	0.002	0.002
4	1	0.09	0.007	0.007	<0.02	0.000	0.002
4	2	0.09	0.007	0.007	<0.02	0.000	0.002
4	3	0.09	0.007	0.007	<0.02	0.000	0.002
4	4	0.07	0.006	0.006	<0.02	0.000	0.002
4	5	0.07	0.006	0.006	<0.02	0.000	0.002
4	6	0.10	0.008	0.008	<0.02	0.000	0.002
4	7	0.30	0.024	0.024	<0.02	0.000	0.002
4	8	0.24	0.020	0.020	<0.02	0.000	0.002
5	1	0.10	0.008	0.008	0.03	0.002	0.002
5	2	0.08	0.007	0.007	0.02	0.002	0.002
5	3	0.09	0.008	0.008	0.02	0.002	0.002
5	4	0.07	0.005	0.005	0.03	0.002	0.002
5	5	<0.06	0.0	0.005	0.05	0.004	0.004
5	6	0.08	0.006	0.006	0.04	0.003	0.003
5	7	0.29	0.023	0.023	0.06	0.004	0.004
5	8	0.25	0.020	0.020	0.05	0.004	0.004
			∑ min	∑ max		∑ min	∑ max
1			0.074	0.074		0.002	0.013
2			0.083	0.083		0.000	0.013
3			0.081	0.081		0.005	0.014
4			0.085	0.085		0.000	0.013
5			0.077	0.082		0.023	0.023
Mean			0.080	0.081		0.006	0.015
SD			0.004	0.004		0.010	0.005

Table F. Continued Granite.

Stone	Fraction	LAB 1 Se µg/l	min r mg/m^2	max r mg/m^2	LAB 2 Se µg/l	min r mg/m^2	max r mg/m^2
1	1	<0.04	0.000	0.003	<0.05	0.000	0.004
1	2	<0.04	0.000	0.003	<0.05	0.000	0.004
1	3	<0.04	0.000	0.003	<0.05	0.000	0.004
1	4	<0.04	0.000	0.003	<0.05	0.000	0.004
1	5	<0.04	0.000	0.003	<0.05	0.000	0.004
1	6	<0.04	0.000	0.003	<0.05	0.000	0.004
1	7	<0.04	0.000	0.003	<0.05	0.000	0.004
1	8	<0.04	0.000	0.003	<0.05	0.000	0.004
2	1	<0.04	0.000	0.003	<0.05	0.000	0.004
2	2	<0.04	0.000	0.003	<0.05	0.000	0.004
2	3	<0.04	0.000	0.003	<0.05	0.000	0.004
2	4	<0.04	0.000	0.003	<0.05	0.000	0.004
2	5	<0.04	0.000	0.003	<0.05	0.000	0.004
2	6	<0.04	0.000	0.003	<0.05	0.000	0.004
2	7	<0.04	0.000	0.003	<0.05	0.000	0.004
2	8	<0.04	0.000	0.003	<0.05	0.000	0.004
3	1	<0.04	0.000	0.003	<0.05	0.000	0.004
3	2	<0.04	0.000	0.003	<0.05	0.000	0.004
3	3	<0.04	0.000	0.003	<0.05	0.000	0.004
3	4	<0.04	0.000	0.003	<0.05	0.000	0.004
3	5	<0.04	0.000	0.003	<0.05	0.000	0.004
3	6	<0.04	0.000	0.003	<0.05	0.000	0.004
3	7	<0.04	0.000	0.003	<0.05	0.000	0.004
3	8	<0.04	0.000	0.003	<0.05	0.000	0.004
4	1	<0.04	0.000	0.003	<0.05	0.000	0.004
4	2	<0.04	0.000	0.003	<0.05	0.000	0.004
4	3	<0.04	0.000	0.003	<0.05	0.000	0.004
4	4	<0.04	0.000	0.003	<0.05	0.000	0.004
4	5	<0.04	0.000	0.003	<0.05	0.000	0.004
4	6	<0.04	0.000	0.003	<0.05	0.000	0.004
4	7	<0.04	0.000	0.003	<0.05	0.000	0.004
4	8	<0.04	0.000	0.003	<0.05	0.000	0.004
5	1	<0.04	0.000	0.003	<0.05	0.000	0.004
5	2	<0.04	0.000	0.003	<0.05	0.000	0.004
5	3	<0.04	0.000	0.003	<0.05	0.000	0.004
5	4	<0.04	0.000	0.003	<0.05	0.000	0.004
5	5	<0.04	0.000	0.003	<0.05	0.000	0.004
5	6	<0.04	0.000	0.003	<0.05	0.000	0.004
5	7	<0.04	0.000	0.003	<0.05	0.000	0.004
5	8	<0.04	0.000	0.003	<0.05	0.000	0.004
			∑ min	∑ max		∑ min	∑ max
1			0.000	0.026		0.000	0.032
2			0.000	0.026		0.000	0.032
3			0.000	0.026		0.000	0.032
4			0.000	0.026		0.000	0.032
5			0.000	0.026		0.000	0.032
Mean			0.000	0.026		0.000	0.032
SD			0.000	0.000		0.000	0.000

Table F. Continued Granite.

Stone	Fraction	LAB 1 Sn µg/l	min r mg/m^2	max r mg/m^2	LAB 2 Sn µg/l	min r mg/m^2	max r mg/m^2
1	1	0.28	0.022	0.022	<0.03	0.000	0.002
1	2	0.28	0.022	0.022	<0.03	0.000	0.002
1	3	0.36	0.029	0.029	<0.03	0.000	0.002
1	4	0.25	0.020	0.020	<0.03	0.000	0.002
1	5	0.34	0.027	0.027	<0.03	0.000	0.002
1	6	0.23	0.019	0.019	<0.03	0.000	0.002
1	7	0.11	0.009	0.009	<0.03	0.000	0.002
1	8	0.16	0.013	0.013	<0.03	0.000	0.002
2	1	0.18	0.015	0.015	<0.03	0.000	0.002
2	2	0.15	0.012	0.012	<0.03	0.000	0.002
2	3	0.21	0.017	0.017	<0.03	0.000	0.002
2	4	0.15	0.012	0.012	<0.03	0.000	0.002
2	5	0.19	0.015	0.015	<0.03	0.000	0.002
2	6	0.16	0.013	0.013	<0.03	0.000	0.002
2	7	0.10	0.008	0.008	<0.03	0.000	0.002
2	8	0.14	0.011	0.011	<0.03	0.000	0.002
3	1	0.14	0.011	0.011	<0.03	0.000	0.002
3	2	0.09	0.007	0.007	<0.03	0.000	0.002
3	3	0.12	0.010	0.010	<0.03	0.000	0.002
3	4	0.10	0.008	0.008	<0.03	0.000	0.002
3	5	0.11	0.009	0.009	<0.03	0.000	0.002
3	6	0.09	0.007	0.007	<0.03	0.000	0.002
3	7	0.06	0.005	0.005	<0.03	0.000	0.002
3	8	0.07	0.006	0.006	<0.03	0.000	0.002
4	1	0.15	0.012	0.012	<0.03	0.000	0.002
4	2	0.10	0.008	0.008	<0.03	0.000	0.002
4	3	0.13	0.011	0.011	<0.03	0.000	0.002
4	4	0.10	0.008	0.008	<0.03	0.000	0.002
4	5	0.12	0.010	0.010	<0.03	0.000	0.002
4	6	0.11	0.009	0.009	<0.03	0.000	0.002
4	7	0.08	0.007	0.007	<0.03	0.000	0.002
4	8	0.09	0.007	0.007	<0.03	0.000	0.002
5	1	0.33	0.027	0.027	<0.03	0.000	0.002
5	2	0.49	0.040	0.040	<0.03	0.000	0.002
5	3	0.56	0.044	0.044	<0.03	0.000	0.002
5	4	0.39	0.031	0.031	<0.03	0.000	0.002
5	5	0.19	0.015	0.015	<0.03	0.000	0.002
5	6	0.11	0.009	0.009	<0.03	0.000	0.002
5	7	0.25	0.020	0.020	<0.03	0.000	0.002
5	8	0.17	0.014	0.014	<0.03	0.000	0.002
			∑ min	∑ max		∑ min	∑ max
1			0.161	0.161		0.000	0.019
2			0.103	0.103		0.000	0.019
3			0.063	0.063		0.000	0.019
4			0.072	0.072		0.000	0.019
5			0.199	0.199		0.000	0.019
Mean			0.120	0.120		0.000	0.019
SD			0.059	0.059		0.000	0.000

Table F. Continued Granite.

Stone	Fraction	LAB 1 Sr µg/l	min r mg/m^2	max r mg/m^2	LAB 2 Sr µg/l	min r mg/m^2	max r mg/m^2
1	1	6.52	0.521	0.521	3.10	0.248	0.248
1	2	7.16	0.573	0.573	3.07	0.245	0.245
1	3	8.00	0.640	0.640	3.07	0.245	0.245
1	4	7.67	0.613	0.613	3.06	0.245	0.245
1	5	8.46	0.677	0.677	3.08	0.247	0.247
1	6	8.21	0.657	0.657	3.05	0.244	0.244
1	7	9.47	0.757	0.757	3.10	0.248	0.248
1	8	10.00	0.800	0.800	3.13	0.250	0.250
2	1	7.67	0.614	0.614	3.07	0.246	0.246
2	2	6.93	0.554	0.554	3.20	0.256	0.256
2	3	7.11	0.569	0.569	3.29	0.263	0.263
2	4	6.90	0.552	0.552	3.39	0.271	0.271
2	5	8.43	0.674	0.674	4.19	0.335	0.335
2	6	7.34	0.587	0.587	4.45	0.355	0.355
2	7	8.06	0.645	0.645	5.76	0.461	0.461
2	8	7.98	0.638	0.638	5.96	0.477	0.477
3	1	7.20	0.576	0.576	3.05	0.244	0.244
3	2	7.38	0.590	0.590	3.08	0.246	0.246
3	3	7.34	0.587	0.587	3.06	0.245	0.245
3	4	7.28	0.582	0.582	3.06	0.245	0.245
3	5	8.00	0.640	0.640	3.12	0.250	0.250
3	6	8.49	0.679	0.679	3.11	0.249	0.249
3	7	8.72	0.698	0.698	3.41	0.273	0.273
3	8	7.70	0.616	0.616	3.45	0.276	0.276
4	1	6.76	0.541	0.541	3.24	0.259	0.259
4	2	6.64	0.531	0.531	3.61	0.288	0.288
4	3	2.48	0.199	0.199	3.86	0.308	0.308
4	4	2.39	0.191	0.191	4.03	0.322	0.322
4	5	2.76	0.221	0.221	5.40	0.432	0.432
4	6	8.02	0.642	0.642	5.96	0.476	0.476
4	7	7.82	0.625	0.625	8.22	0.657	0.657
4	8	5.74	0.459	0.459	8.58	0.686	0.686
5	1	6.32	0.505	0.505	3.05	0.244	0.244
5	2	6.72	0.538	0.538	3.07	0.245	0.245
5	3	7.03	0.562	0.562	3.07	0.245	0.245
5	4	3.96	0.317	0.317	3.06	0.245	0.245
5	5	2.15	0.172	0.172	3.23	0.258	0.258
5	6	3.02	0.242	0.242	3.26	0.260	0.260
5	7	7.77	0.622	0.622	3.59	0.287	0.287
5	8	8.58	0.686	0.686	3.62	0.289	0.289
			∑ min	∑ max		∑ min	∑ max
1			5.237	5.237		1.972	1.972
2			4.833	4.833		2.662	2.662
3			4.968	4.968		2.027	2.027
4			3.409	3.409		3.428	3.428
5			3.643	3.643		2.073	2.073
Mean			4.418	4.418		2.433	2.433
SD			0.831	0.831		0.622	0.622

Table F. Continued Granite.

Stone	Fraction	LAB 1 U µg/l	min r mg/m²	max r mg/m²	LAB 2 U µg/l	min r mg/m²	max r mg/m²
1	1	0.04	0.004	0.004	0.04	0.003	0.003
1	2	<0.04	0.000	0.003	0.02	0.001	0.001
1	3	<0.04	0.000	0.003	0.01	0.001	0.001
1	4	0.04	0.003	0.003	0.01	0.001	0.001
1	5	0.06	0.005	0.005	0.01	0.001	0.001
1	6	0.06	0.004	0.004	0.01	0.001	0.001
1	7	0.20	0.016	0.016	0.02	0.001	0.001
1	8	0.14	0.011	0.011	0.02	0.001	0.001
2	1	0.07	0.005	0.005	0.06	0.004	0.004
2	2	0.06	0.005	0.005	0.08	0.006	0.006
2	3	0.07	0.006	0.006	0.09	0.007	0.007
2	4	0.06	0.005	0.005	0.12	0.009	0.009
2	5	0.13	0.010	0.010	0.23	0.018	0.018
2	6	0.15	0.012	0.012	0.21	0.017	0.017
2	7	0.30	0.024	0.024	0.38	0.030	0.030
2	8	0.41	0.032	0.032	0.35	0.028	0.028
3	1	0.06	0.004	0.004	0.02	0.002	0.002
3	2	0.04	0.003	0.003	0.02	0.002	0.002
3	3	0.05	0.004	0.004	0.02	0.002	0.002
3	4	0.06	0.005	0.005	0.02	0.001	0.001
3	5	0.09	0.007	0.007	0.03	0.002	0.002
3	6	0.13	0.010	0.010	0.03	0.003	0.003
3	7	0.25	0.020	0.020	0.06	0.005	0.005
3	8	0.14	0.011	0.011	0.06	0.005	0.005
4	1	0.09	0.007	0.007	0.07	0.006	0.006
4	2	0.06	0.005	0.005	0.11	0.009	0.009
4	3	0.07	0.006	0.006	0.15	0.012	0.012
4	4	0.06	0.005	0.005	0.22	0.017	0.017
4	5	0.12	0.010	0.010	0.71	0.057	0.057
4	6	0.14	0.011	0.011	0.71	0.057	0.057
4	7	0.19	0.015	0.015	1.42	0.113	0.113
4	8	0.25	0.020	0.020	1.54	0.123	0.123
5	1	0.12	0.009	0.009	0.14	0.011	0.011
5	2	0.08	0.006	0.006	0.05	0.004	0.004
5	3	0.08	0.007	0.007	0.06	0.005	0.005
5	4	0.06	0.004	0.004	0.08	0.007	0.007
5	5	0.22	0.018	0.018	0.12	0.009	0.009
5	6	0.16	0.013	0.013	0.13	0.010	0.010
5	7	0.34	0.027	0.027	0.22	0.018	0.018
5	8	0.50	0.040	0.040	0.20	0.016	0.016
			∑ min	∑ max		∑ min	∑ max
1			0.043	0.049		0.011	0.011
2			0.100	0.100		0.120	0.120
3			0.065	0.065		0.021	0.021
4			0.079	0.079		0.394	0.394
5			0.124	0.124		0.081	0.081
Mean			0.082	0.084		0.125	0.125
SD			0.031	0.029		0.156	0.156

Table F. Continued Granite.

Stone	Fraction	LAB 1 V µg/l	min r mg/m²	max r mg/m²	LAB 2 V µg/l	min r mg/m²	max r mg/m²
1	1	0.03	0.002	0.002	0.04	0.003	0.003
1	2	<0.02	0.0	0.002	0.08	0.007	0.007
1	3	<0.02	0.0	0.002	0.03	0.002	0.002
1	4	<0.02	0.0	0.002	0.03	0.002	0.002
1	5	<0.02	0.0	0.002	0.05	0.004	0.004
1	6	<0.02	0.0	0.002	<0.02	0.0	0.002
1	7	0.07	0.005	0.005	<0.02	0.0	0.002
1	8	0.03	0.002	0.002	<0.02	0.0	0.002
2	1	0.05	0.004	0.004	<0.02	0.0	0.002
2	2	<0.02	0.0	0.002	<0.02	0.0	0.002
2	3	<0.02	0.0	0.002	<0.02	0.0	0.002
2	4	<0.02	0.0	0.002	0.02	0.002	0.002
2	5	<0.02	0.0	0.002	0.03	0.003	0.003
2	6	<0.02	0.0	0.002	<0.02	0.0	0.002
2	7	0.04	0.003	0.003	<0.02	0.0	0.002
2	8	<0.02	0.0	0.002	<0.02	0.0	0.002
3	1	0.04	0.003	0.003	<0.02	0.0	0.002
3	2	<0.02	0.0	0.002	<0.02	0.0	0.002
3	3	<0.02	0.0	0.002	<0.02	0.0	0.002
3	4	<0.02	0.0	0.002	<0.02	0.0	0.002
3	5	<0.02	0.0	0.002	0.05	0.004	0.004
3	6	<0.02	0.0	0.002	<0.02	0.0	0.002
3	7	0.04	0.003	0.003	<0.02	0.0	0.002
3	8	<0.02	0.0	0.002	<0.02	0.0	0.002
4	1	0.05	0.004	0.004	<0.02	0.0	0.002
4	2	<0.02	0.0	0.002	<0.02	0.0	0.002
4	3	<0.02	0.0	0.002	<0.02	0.0	0.002
4	4	<0.02	0.0	0.002	0.02	0.002	0.002
4	5	<0.02	0.0	0.002	0.04	0.003	0.003
4	6	<0.02	0.0	0.002	<0.02	0.0	0.002
4	7	0.03	0.003	0.003	<0.02	0.0	0.002
4	8	<0.02	0.0	0.002	<0.02	0.0	0.002
5	1	0.04	0.003	0.003	<0.02	0.0	0.002
5	2	<0.02	0.0	0.002	<0.02	0.0	0.002
5	3	0.03	0.002	0.002	<0.02	0.0	0.002
5	4	<0.02	0.0	0.002	0.02	0.002	0.002
5	5	0.23	0.018	0.018	0.04	0.003	0.003
5	6	<0.02	0.0	0.002	<0.02	0.0	0.002
5	7	0.75	0.060	0.060	<0.02	0.0	0.002
5	8	0.02	0.002	0.002	<0.02	0.0	0.002
			∑ min	∑ max		∑ min	∑ max
1			0.010	0.018		0.02	0.02
2			0.007	0.017		0.00	0.01
3			0.006	0.016		0.00	0.02
4			0.007	0.016		0.00	0.01
5			0.085	0.090		0.00	0.01
Mean			0.023	0.031		0.01	0.02
SD			0.035	0.033		0.01	0.00

Table F. Continued Granite.

Stone	Fraction	LAB 1 Zn µg/l	min r mg/m²	max r mg/m²	LAB 2 Zn µg/l	min r mg/m²	max r mg/m²
1	1	255	20.40	20.40	7.52	0.602	0.602
1	2	259	20.75	20.75	53.42	4.275	4.275
1	3	361	28.90	28.90	3.74	0.299	0.299
1	4	449	35.92	35.92	3.88	0.310	0.310
1	5	451	36.08	36.08	27.05	2.165	2.165
1	6	318	25.43	25.43	0.27	0.021	0.021
1	7	348	27.87	27.87	0.41	0.033	0.033
1	8	334	26.67	26.67	0.20	0.016	0.016
2	1	273	21.81	21.81	0.31	0.025	0.025
2	2	274	21.94	21.94	0.81	0.065	0.065
2	3	268	21.45	21.45	0.36	0.029	0.029
2	4	395	31.56	31.56	3.57	0.285	0.285
2	5	470	37.58	37.58	22.27	1.781	1.781
2	6	287	22.93	22.93	0.33	0.026	0.026
2	7	312	24.98	24.98	0.24	0.019	0.019
2	8	249	19.94	19.94	0.11	0.009	0.009
3	1	390	31.18	31.18	0.53	0.043	0.043
3	2	283	22.62	22.62	1.06	0.085	0.085
3	3	421	33.68	33.68	0.16	0.013	0.013
3	4	307	24.52	24.52	3.98	0.318	0.318
3	5	433	34.62	34.62	37.09	2.967	2.967
3	6	459	36.73	36.73	0.25	0.020	0.020
3	7	295	23.56	23.56	0.23	0.019	0.019
3	8	219	17.53	17.53	0.20	0.016	0.016
4	1	276	22.05	22.05	0.39	0.031	0.031
4	2	416	33.24	33.24	0.40	0.032	0.032
4	3	105	8.40	8.40	0.16	0.013	0.013
4	4	175	14.03	14.03	4.70	0.375	0.375
4	5	144	11.49	11.49	19.11	1.527	1.527
4	6	426	34.08	34.08	0.21	0.017	0.017
4	7	339	27.08	27.08	0.18	0.014	0.014
4	8	194	15.53	15.53	<0.11	0.000	0.009
5	1	393	31.43	31.43	0.38	0.031	0.031
5	2	430	34.38	34.38	0.56	0.045	0.045
5	3	292	23.36	23.36	0.22	0.018	0.018
5	4	290	23.18	23.18	2.87	0.229	0.229
5	5	96	7.65	7.65	22.48	1.797	1.797
5	6	162	12.93	12.93	0.22	0.017	0.017
5	7	283	22.60	22.60	0.21	0.017	0.017
5	8	297	23.72	23.72	<0.11	0.000	0.009
			∑ min	∑ max		∑ min	∑ max
1			222	222		7.721	7.721
2			202	202		2.238	2.238
3			224	224		3.480	3.480
4			166	166		2.010	2.019
5			179	179		2.154	2.162
Mean			199	199		3.521	3.524
SD			25.9	25.9		2.421	2.418

Table G. DSLT results CUS (LAB 1 DSLT contract laboratory, LAB 2 DSLT BfG, for CUS3 n=4)

Stone	Fraction	LAB 1 CUS1 Al µg/l	min r mg/m²	max r mg/m²	LAB 2 CUS1 Al µg/l	min r mg/m²	max r mg/m²
1	1	<40.8	0.00	3.26	<40.8	0.00	3.26
1	2	<40.8	0.00	3.26	<40.8	0.00	3.26
1	3	<40.8	0.00	3.26	<40.8	0.00	3.26
1	4	<40.8	0.00	3.26	<40.8	0.00	3.26
1	5	<40.8	0.00	3.26	<40.8	0.00	3.26
1	6	<40.8	0.00	3.26	<40.8	0.00	3.26
1	7	<40.8	0.00	3.26	<40.8	0.00	3.26
1	8	<40.8	0.00	3.26	<40.8	0.00	3.26
2	1	<40.8	0.00	3.26	<40.8	0.00	3.26
2	2	<40.8	0.00	3.26	<40.8	0.00	3.26
2	3	<40.8	0.00	3.26	<40.8	0.00	3.26
2	4	<40.8	0.00	3.26	<40.8	0.00	3.26
2	5	<40.8	0.00	3.26	<40.8	0.00	3.26
2	6	<40.8	0.00	3.26	<40.8	0.00	3.26
2	7	127	10.17	10.17	<40.8	0.00	10.17
2	8	<40.8	0.00	3.26	<40.8	0.00	3.26
3	1	<40.8	0.00	3.26	<40.8	0.00	3.26
3	2	<40.8	0.00	3.26	<40.8	0.00	3.26
3	3	<40.8	0.00	3.26	<40.8	0.00	3.26
3	4	<40.8	0.00	3.26	<40.8	0.00	3.26
3	5	<40.8	0.00	3.26	<40.8	0.00	3.26
3	6	<40.8	0.00	3.26	<40.8	0.00	3.26
3	7	<40.8	0.00	3.26	<40.8	0.00	3.26
3	8	<40.8	0.00	3.26	<40.8	0.00	3.26
4	1	<40.8	0.00	3.26	<40.8	0.00	3.26
4	2	<40.8	0.00	3.26	<40.8	0.00	3.26
4	3	<40.8	0.00	3.26	<40.8	0.00	3.26
4	4	<40.8	0.00	3.26	<40.8	0.00	3.26
4	5	<40.8	0.00	3.26	<40.8	0.00	3.26
4	6	<40.8	0.00	3.26	<40.8	0.00	3.26
4	7	<40.8	0.00	3.26	<40.8	0.00	3.26
4	8	<40.8	0.00	3.26	109	0.00	3.26
5	1	<40.8	0.00	3.26	<40.8	0.00	3.26
5	2	<40.8	0.00	3.26	<40.8	0.00	3.26
5	3	<40.8	0.00	3.26	<40.8	0.00	3.26
5	4	<40.8	0.00	3.26	<40.8	0.00	3.26
5	5	<40.8	0.00	3.26	<40.8	0.00	3.26
5	6	<40.8	0.00	3.26	<40.8	0.00	3.26
5	7	<40.8	0.00	3.26	<40.8	0.00	3.26
5	8	<40.8	0.00	3.26	<40.8	0.00	3.26
			∑ min	∑ max		∑ min	∑ max
1			0.00	26.11		0.00	25.55
2			10.17	33.02		0.00	25.59
3			0.00	26.11		0.00	25.59
4			0.00	26.11		8.67	31.05
5			0.00	26.11		0.00	25.63
Mean			2.03	27.49		1.73	26.68
SD			4.55	3.09		3.88	2.44

Table G. Continued CUS.

Stone	Fraction	LAB 2 CUS2 Al µg/l	min r mg/m^2	max r mg/m^2	LAB 2 CUS3 Al µg/l	min r mg/m^2	max r mg/m^2
1	1	<50	0.00	4.00			
1	2	<50	0.00	4.00			
1	3	<50	0.00	4.00			
1	4	<50	0.00	4.00			
1	5	<50	0.00	4.00			
1	6	<50	0.00	4.00			
1	7	<50	0.00	4.00			
1	8	<50	0.00	4.00			
2	1	<50	0.00	4.00	<50	0.00	4.01
2	2	<50	0.00	4.00	<50	0.00	4.01
2	3	<50	0.00	4.00	<50	0.00	4.01
2	4	<50	0.00	4.00	<50	0.00	4.01
2	5	<50	0.00	4.00	<50	0.00	4.01
2	6	<50	0.00	4.00	<50	0.00	4.01
2	7	<50	0.00	4.00	<50	0.00	4.01
2	8	<50	0.00	4.00	<50	0.00	4.01
3	1	<50	0.00	4.00	<50	0.00	4.00
3	2	<50	0.00	4.00	<50	0.00	4.00
3	3	<50	0.00	4.00	<50	0.00	4.00
3	4	<50	0.00	4.00	<50	0.00	4.00
3	5	<50	0.00	4.00	<50	0.00	4.00
3	6	<50	0.00	4.00	<50	0.00	4.00
3	7	<50	0.00	4.00	<50	0.00	4.00
3	8	<50	0.00	4.00	<50	0.00	4.00
4	1	<50	0.00	4.00	<50	0.00	4.00
4	2	<50	0.00	4.00	<50	0.00	4.00
4	3	<50	0.00	4.00	<50	0.00	4.00
4	4	<50	0.00	4.00	<50	0.00	4.00
4	5	<50	0.00	4.00	<50	0.00	4.00
4	6	<50	0.00	4.00	<50	0.00	4.00
4	7	<50	0.00	4.00	<50	0.00	4.00
4	8	<50	0.00	4.00	<50	0.00	4.00
5	1	<50	0.00	4.00	<50	0.00	4.00
5	2	<50	0.00	4.00	<50	0.00	4.00
5	3	<50	0.00	4.00	<50	0.00	4.00
5	4	<50	0.00	4.00	<50	0.00	4.00
5	5	<50	0.00	4.00	<50	0.00	4.00
5	6	<50	0.00	4.00	<50	0.00	4.00
5	7	<50	0.00	4.00	<50	0.00	4.00
5	8	<50	0.00	4.00	<50	0.00	4.00
			∑ min	∑ max		∑ min	∑ max
1			0.00	32.0			
2			0.00	32.0		0.00	32.05
3			0.00	32.0		0.00	32.03
4			0.00	32.0		0.00	31.98
5			0.00	32.0		0.00	32.00
Mean			0.00	32.00		0.00	32.01
SD			0.00	0.02		0.00	0.03

Table G. Continued CUS.

Stone	Fraction	LAB 1 CUS1 As µg/l	min r mg/m^2	max r mg/m^2	LAB 2 CUS1 As µg/l	min r mg/m2	max r mg/m2
1	1	0.54	0.04	0.04	0.55	0.00	0.04
1	2	0.54	0.04	0.04	0.60	0.05	0.05
1	3	0.89	0.07	0.07	0.84	0.07	0.07
1	4	1.35	0.11	0.11	1.14	0.09	0.09
1	5	2.39	0.19	0.19	2.01	0.16	0.16
1	6	2.78	0.22	0.22	2.39	0.19	0.19
1	7	6.10	0.49	0.49	2.53	0.20	0.20
1	8	2.19	0.18	0.18	2.48	0.20	0.20
2	1	0.83	0.07	0.07	0.65	0.05	0.05
2	2	1.63	0.13	0.13	0.67	0.05	0.05
2	3	2.46	0.20	0.20	0.68	0.05	0.05
2	4	3.13	0.25	0.25	0.91	0.07	0.07
2	5	5.16	0.41	0.41	2.04	0.16	0.16
2	6	5.87	0.47	0.47	2.01	0.16	0.16
2	7	11.76	0.94	0.94	1.46	0.12	0.12
2	8	3.18	0.25	0.25	1.20	0.10	0.10
3	1	0.36	0.03	0.03	0.55	0.00	0.04
3	2	0.54	0.04	0.04	0.55	0.00	0.04
3	3	0.94	0.08	0.08	0.55	0.00	0.04
3	4	1.73	0.14	0.14	0.71	0.06	0.06
3	5	3.22	0.26	0.26	1.40	0.11	0.11
3	6	4.50	0.36	0.36	1.25	0.10	0.10
3	7	10.11	0.81	0.81	1.20	0.10	0.10
3	8	5.31	0.42	0.42	1.16	0.09	0.09
4	1	0.51	0.04	0.04	0.55	0.00	0.04
4	2	0.57	0.05	0.05	0.55	0.00	0.04
4	3	1.13	0.09	0.09	0.55	0.00	0.04
4	4	1.49	0.12	0.12	0.55	0.00	0.04
4	5	2.34	0.19	0.19	0.83	0.07	0.07
4	6	2.60	0.21	0.21	1.08	0.09	0.09
4	7	4.50	0.36	0.36	0.94	0.07	0.07
4	8	1.84	0.15	0.15	0.80	0.06	0.06
5	1	0.77	0.06	0.06	0.55	0.00	0.04
5	2	0.79	0.06	0.06	0.55	0.00	0.04
5	3	1.16	0.09	0.09	0.55	0.00	0.04
5	4	1.32	0.11	0.11	0.57	0.05	0.05
5	5	2.10	0.17	0.17	1.30	0.10	0.10
5	6	2.44	0.19	0.19	1.31	0.11	0.11
5	7	4.85	0.39	0.39	1.40	0.11	0.11
5	8	2.87	0.23	0.23	1.37	0.11	0.11
			∑ min	∑ max		∑ min	∑ max
1			1.34	1.34		0.96	1.00
2			2.72	2.72		0.77	0.77
3			2.14	2.14		0.46	0.59
4			1.20	1.20		0.29	0.47
5			1.30	1.30		0.48	0.61
Mean			1.74	1.74		0.59	0.69
SD			0.66	0.66		0.27	0.21

Table G. Continued CUS.

Stone	Fraction	LAB 2 CUS2 As µg/l	min r mg/m^2	max r mg/m^2	LAB 2 CUS3 As µg/l	min r mg/m^2	max r mg/m^2
1	1	<0.27	0.00	0.02			
1	2	0.42	0.03	0.03			
1	3	0.57	0.05	0.05			
1	4	0.65	0.05	0.05			
1	5	0.84	0.07	0.07			
1	6	0.69	0.06	0.06			
1	7	0.88	0.07	0.07			
1	8	0.61	0.05	0.05			
2	1	<0.27	0.00	0.02	0.44	0.035	0.035
2	2	<0.27	0.00	0.02	0.55	0.044	0.044
2	3	<0.27	0.00	0.02	0.54	0.043	0.043
2	4	<0.27	0.00	0.02	0.86	0.069	0.069
2	5	<0.27	0.00	0.02	0.85	0.068	0.068
2	6	0.51	0.04	0.04	0.90	0.072	0.072
2	7	<0.27	0.00	0.02	0.70	0.056	0.056
2	8	<0.27	0.00	0.02	0.64	0.051	0.051
3	1	<0.27	0.00	0.02	0.47	0.038	0.038
3	2	0.29	0.02	0.02	1.62	0.130	0.130
3	3	0.44	0.04	0.04	2.40	0.192	0.192
3	4	0.50	0.04	0.04	3.41	0.273	0.273
3	5	0.52	0.04	0.04	3.47	0.277	0.277
3	6	0.53	0.04	0.04	3.03	0.242	0.242
3	7	0.46	0.04	0.04	2.55	0.204	0.204
3	8	0.43	0.03	0.03	2.37	0.190	0.190
4	1	<0.27	0.00	0.02	0.43	0.034	0.034
4	2	0.34	0.03	0.03	0.72	0.057	0.057
4	3	0.44	0.04	0.04	0.75	0.060	0.060
4	4	0.45	0.04	0.04	0.98	0.078	0.078
4	5	0.37	0.03	0.03	1.32	0.106	0.106
4	6	0.29	0.02	0.02	1.24	0.099	0.099
4	7	<0.27	0.00	0.02	1.32	0.105	0.105
4	8	<0.27	0.00	0.02	1.34	0.107	0.107
5	1	0.30	0.02	0.02	0.62	0.049	0.049
5	2	0.63	0.05	0.05	0.66	0.053	0.053
5	3	0.82	0.07	0.07	0.72	0.058	0.058
5	4	0.93	0.07	0.07	0.74	0.059	0.059
5	5	1.51	0.12	0.12	1.03	0.083	0.083
5	6	1.15	0.09	0.09	1.17	0.093	0.093
5	7	1.10	0.09	0.09	1.05	0.084	0.084
5	8	1.12	0.09	0.09	1.01	0.081	0.081
			∑ min	∑ max		∑ min	∑ max
1			0.37	0.39			
2			0.04	0.19		0.439	0.439
3			0.25	0.28		1.547	1.547
4			0.15	0.22		0.648	0.648
5			0.60	0.60		0.561	0.561
Mean			0.28	0.34		0.798	0.798
SD			0.22	0.17		0.506	0.506

Table G. Continued CUS.

Stone	Fraction	LAB 1 CUS1 Ba µg/l	min r mg/m^2	max r mg/m^2	LAB 2 CUS1 Ba µg/l	min r mg/m^2	max r mg/m^2
1	1	190	15.21	15.21	4.03	0.32	0.32
1	2	458	36.61	36.61	2.65	0.21	0.21
1	3	337	26.92	26.92	3.58	0.29	0.29
1	4	62	4.92	4.92	4.69	0.37	0.37
1	5	363	29.02	29.02	5.37	0.43	0.43
1	6	417	33.37	33.37	6.67	0.53	0.53
1	7	346	27.65	27.65	4.52	0.36	0.36
1	8	371	29.65	29.65	4.64	0.37	0.37
2	1	443	35.44	35.44	6.40	0.51	0.51
2	2	406	32.44	32.44	4.28	0.34	0.34
2	3	219	17.52	17.52	3.76	0.30	0.30
2	4	393	31.45	31.45	4.28	0.34	0.34
2	5	178	14.27	14.27	5.23	0.42	0.42
2	6	361	28.85	28.85	4.54	0.36	0.36
2	7	495	39.62	39.62	6.32	0.51	0.51
2	8	233	18.67	18.67	3.44	0.27	0.27
3	1	396	31.66	31.66	6.53	0.52	0.52
3	2	389	31.08	31.08	2.71	0.22	0.22
3	3	413	33.07	33.07	7.61	0.61	0.61
3	4	160	12.76	12.76	5.32	0.43	0.43
3	5	111	8.86	8.86	4.41	0.35	0.35
3	6	447	35.79	35.79	4.23	0.34	0.34
3	7	343	27.47	27.47	3.67	0.29	0.29
3	8	301	24.10	24.10	7.64	0.61	0.61
4	1	382	30.56	30.56	6.56	0.52	0.52
4	2	422	33.74	33.74	4.13	0.33	0.33
4	3	302	24.14	24.14	5.30	0.42	0.42
4	4	369	29.49	29.49	4.68	0.37	0.37
4	5	81	6.51	6.51	4.19	0.33	0.33
4	6	404	32.29	32.29	3.96	0.32	0.32
4	7	357	28.54	28.54	6.51	0.52	0.52
4	8	338	27.05	27.05	17.65	1.41	1.41
5	1	414	33.10	33.10	11.40	0.91	0.91
5	2	118	9.42	9.42	2.70	0.22	0.22
5	3	394	31.49	31.49	4.56	0.37	0.37
5	4	192	15.34	15.34	5.18	0.41	0.41
5	5	412	32.93	32.93	5.06	0.41	0.41
5	6	423	33.82	33.82	3.66	0.29	0.29
5	7	397	31.74	31.74	5.09	0.41	0.41
5	8	364	29.09	29.09			
			∑ min	∑ max		∑ min	∑ max
1			203	203		2.89	2.89
2			218	218		3.06	3.06
3			205	218		3.37	3.37
4			212	212		4.23	4.23
5			217	217		3.66	3.66
Mean			211	214		3.44	3.44
SD			7	6		0.53	0.53

Table G. Continued CUS.

Stone	Fraction	LAB 2 CUS2 Ba µg/l	min r mg/m^2	max r mg/m^2	LAB 2 CUS3 Ba µg/l	min r mg/m^2	max r mg/m^2
1	1	0.68	0.055	0.055			
1	2	0.91	0.073	0.073			
1	3	0.77	0.062	0.062			
1	4	0.65	0.052	0.052			
1	5	0.89	0.071	0.071			
1	6	0.77	0.061	0.061			
1	7	1.38	0.110	0.110			
1	8	1.49	0.119	0.119			
2	1	1.05	0.084	0.084	<0.73	0.000	0.058
2	2	0.69	0.055	0.055	<0.73	0.000	0.058
2	3	0.79	0.063	0.063	0.95	0.076	0.076
2	4	1.03	0.082	0.082	<0.73	0.000	0.058
2	5	2.01	0.161	0.161	0.82	0.066	0.066
2	6	1.38	0.110	0.110	1.05	0.084	0.084
2	7	4.65	0.372	0.372	<0.73	0.000	0.058
2	8	3.98	0.318	0.318	1.90	0.152	0.152
3	1	0.77	0.062	0.062	<0.73	0.000	0.058
3	2	1.08	0.086	0.086	<0.73	0.000	0.058
3	3	1.18	0.094	0.094	0.87	0.070	0.070
3	4	1.17	0.093	0.093	1.28	0.102	0.102
3	5	1.18	0.094	0.094	2.77	0.222	0.222
3	6	1.09	0.087	0.087	2.98	0.239	0.239
3	7	1.63	0.130	0.130	5.19	0.416	0.416
3	8	1.01	0.080	0.080	4.69	0.376	0.376
4	1	1.56	0.125	0.125	0.85	0.068	0.068
4	2	2.70	0.216	0.216	0.95	0.076	0.076
4	3	2.31	0.185	0.185	0.85	0.068	0.068
4	4	2.07	0.166	0.166	1.30	0.104	0.104
4	5	2.90	0.232	0.232	2.32	0.185	0.185
4	6	2.37	0.189	0.189	2.84	0.227	0.227
4	7	3.86	0.309	0.309	4.98	0.398	0.398
4	8	2.68	0.215	0.215	2.86	0.228	0.228
5	1	1.75	0.140	0.140	0.90	0.072	0.072
5	2	1.95	0.156	0.156	<0.73	0.000	0.058
5	3	1.67	0.134	0.134	<0.73	0.000	0.058
5	4	1.35	0.108	0.108	0.76	0.061	0.061
5	5	1.77	0.142	0.142	1.21	0.097	0.097
5	6	1.25	0.100	0.100	2.25	0.180	0.180
5	7	2.08	0.166	0.166	3.62	0.290	0.290
5	8	1.50	0.120	0.120	2.98	0.239	0.239
			∑ min	∑ max		∑ min	∑ max
1			0.602	0.602			
2			1.245	1.245		0.378	0.612
3			0.728	0.728		1.424	1.541
4			1.637	1.637		1.354	1.354
5			1.067	1.067		0.938	1.054
Mean			1.056	1.056		1.024	1.140
SD			0.414	0.414		0.481	0.405

Table G. Continued CUS.

Stone	Fraction	LAB 1 CUS1 Cd µg/l	min r mg/m^2	max r mg/m^2	LAB 2 CUS1 Cd µg/l	min r mg/m2	max r mg/m2
1	1	0.02	0.002	0.002	0.10	0.01	0.01
1	2	0.03	0.002	0.002	0.12	0.01	0.01
1	3	0.03	0.002	0.002	0.26	0.02	0.02
1	4	0.01	0.001	0.001	0.31	0.02	0.02
1	5	0.03	0.002	0.002	0.43	0.03	0.03
1	6	0.03	0.002	0.002	0.28	0.02	0.02
1	7	0.17	0.014	0.014	0.31	0.02	0.02
1	8	0.10	0.008	0.008	0.24	0.02	0.02
2	1	0.04	0.003	0.003	0.04	0.00	0.00
2	2	0.03	0.002	0.002	0.05	0.00	0.00
2	3	0.03	0.002	0.002	0.16	0.01	0.01
2	4	0.03	0.002	0.002	0.25	0.02	0.02
2	5	0.02	0.002	0.002	0.52	0.04	0.04
2	6	0.03	0.003	0.003	0.40	0.03	0.03
2	7	0.19	0.015	0.015	0.45	0.04	0.04
2	8	0.13	0.011	0.011	0.31	0.02	0.02
3	1	0.04	0.003	0.003	0.02	0.00	0.00
3	2	0.05	0.004	0.004	0.06	0.00	0.00
3	3	0.06	0.005	0.005	0.23	0.02	0.02
3	4	0.06	0.005	0.005	0.21	0.02	0.02
3	5	0.15	0.012	0.012	0.26	0.02	0.02
3	6	0.14	0.011	0.011	0.16	0.01	0.01
3	7	0.61	0.049	0.049	0.21	0.02	0.02
3	8	0.31	0.025	0.025	0.18	0.01	0.01
4	1	0.03	0.002	0.002	0.27	0.02	0.02
4	2	0.03	0.002	0.002	0.17	0.01	0.01
4	3	0.03	0.002	0.002	0.47	0.04	0.04
4	4	0.03	0.002	0.002	0.83	0.07	0.07
4	5	0.02	0.002	0.002	1.32	0.11	0.11
4	6	0.05	0.004	0.004	1.02	0.08	0.08
4	7	0.42	0.033	0.033	1.23	0.10	0.10
4	8	0.22	0.018	0.018	1.06	0.08	0.08
5	1	0.03	0.002	0.002	0.04	0.00	0.00
5	2	0.01	0.001	0.001	0.04	0.00	0.00
5	3	0.02	0.002	0.002	0.13	0.01	0.01
5	4	0.02	0.001	0.001	0.22	0.02	0.02
5	5	0.04	0.003	0.003	0.37	0.03	0.03
5	6	0.04	0.003	0.003	0.20	0.02	0.02
5	7	0.28	0.022	0.022	0.27	0.02	0.02
5	8	0.21	0.017	0.017	0.23	0.02	0.02
			∑ min	∑ max		∑ min	∑ max
1			0.033	0.033		0.16	0.16
2			0.040	0.040		0.17	0.17
3			0.114	0.114		0.11	0.11
4			0.066	0.066		0.51	0.51
5			0.052	0.052		0.12	0.12
Mean			0.061	0.061		0.21	0.21
SD			0.032	0.032		0.17	0.17

Table G. Continued CUS.

Stone	Fraction	LAB 2 CUS2 Cd µg/l	min r mg/m²	max r mg/m²	LAB 2 CUS3 Cd µg/l	min r mg/m²	max r mg/m²
1	1	0.47	0.037	0.037			
1	2	0.52	0.041	0.041			
1	3	0.36	0.028	0.028			
1	4	0.30	0.024	0.024			
1	5	0.42	0.034	0.034			
1	6	0.39	0.031	0.031			
1	7	0.74	0.059	0.059			
1	8	0.53	0.042	0.042			
2	1	0.32	0.026	0.026	0.07	0.005	0.005
2	2	0.25	0.020	0.020	0.08	0.006	0.006
2	3	0.20	0.016	0.016	0.06	0.005	0.005
2	4	0.24	0.019	0.019	0.08	0.006	0.006
2	5	0.41	0.033	0.033	0.14	0.011	0.011
2	6	0.39	0.031	0.031	0.16	0.013	0.013
2	7	1.46	0.117	0.117	0.26	0.021	0.021
2	8	1.35	0.108	0.108	0.22	0.017	0.017
3	1	0.12	0.010	0.010	0.08	0.006	0.006
3	2	0.19	0.015	0.015	0.08	0.007	0.007
3	3	0.27	0.021	0.021	0.10	0.008	0.008
3	4	0.31	0.025	0.025	0.16	0.013	0.013
3	5	0.34	0.027	0.027	0.31	0.025	0.025
3	6	0.34	0.027	0.027	0.34	0.027	0.027
3	7	0.56	0.044	0.044	0.49	0.039	0.039
3	8	0.35	0.028	0.028	0.47	0.037	0.037
4	1	0.28	0.022	0.022	0.06	0.005	0.005
4	2	0.21	0.017	0.017	0.10	0.008	0.008
4	3	0.15	0.012	0.012	0.08	0.006	0.006
4	4	0.14	0.011	0.011	0.13	0.010	0.010
4	5	0.18	0.014	0.014	0.23	0.018	0.018
4	6	0.15	0.012	0.012	0.26	0.021	0.021
4	7	0.30	0.024	0.024	0.44	0.035	0.035
4	8	0.18	0.014	0.014	0.29	0.023	0.023
5	1	0.77	0.061	0.061	0.08	0.007	0.007
5	2	0.53	0.042	0.042	0.09	0.008	0.008
5	3	0.42	0.034	0.034	0.09	0.007	0.007
5	4	0.43	0.035	0.035	0.09	0.007	0.007
5	5	0.66	0.053	0.053	0.20	0.016	0.016
5	6	0.53	0.043	0.043	0.27	0.022	0.022
5	7	0.99	0.080	0.080	0.35	0.028	0.028
5	8	0.84	0.067	0.067	0.29	0.023	0.023
			∑ min	∑ max		∑ min	∑ max
1			0.297	0.297			
2			0.370	0.370		0.085	0.085
3			0.198	0.198		0.161	0.161
4			0.127	0.127		0.126	0.126
5			0.414	0.414		0.117	0.117
Mean			0.281	0.281		0.122	0.122
SD			0.119	0.119		0.032	0.032

Table G. Continued CUS.

Stone	Fraction	LAB 1 CUS1 Co µg/l	min r mg/m²	max r mg/m²	LAB 2 CUS1 Co µg/l	min r mg/m²	max r mg/m²
1	1	0.09	0.007	0.007	0.27	0.02	0.02
1	2	0.20	0.016	0.016	0.31	0.02	0.02
1	3	0.27	0.022	0.022	0.46	0.04	0.04
1	4	0.17	0.013	0.013	0.41	0.03	0.03
1	5	0.34	0.027	0.027	0.67	0.05	0.05
1	6	0.38	0.030	0.030	0.49	0.04	0.04
1	7	1.45	0.116	0.116	0.64	0.05	0.05
1	8	1.00	0.080	0.080	0.54	0.04	0.04
2	1	0.14	0.011	0.011	0.16	0.01	0.01
2	2	0.30	0.024	0.024	0.18	0.01	0.01
2	3	0.46	0.037	0.037	0.41	0.03	0.03
2	4	0.38	0.030	0.030	0.50	0.04	0.04
2	5	0.53	0.043	0.043	0.98	0.08	0.08
2	6	0.56	0.045	0.045	0.79	0.06	0.06
2	7	1.72	0.137	0.137	1.13	0.09	0.09
2	8	1.67	0.134	0.134	0.90	0.07	0.07
3	1	0.26	0.021	0.021	0.12	0.01	0.01
3	2	0.36	0.029	0.029	0.29	0.02	0.02
3	3	0.39	0.031	0.031	0.57	0.05	0.05
3	4	0.36	0.029	0.029	0.46	0.04	0.04
3	5	0.58	0.047	0.047	0.76	0.06	0.06
3	6	0.58	0.046	0.046	0.53	0.04	0.04
3	7	1.41	0.113	0.113	0.82	0.07	0.07
3	8	0.85	0.068	0.068	0.82	0.07	0.07
4	1	0.14	0.012	0.012	1.19	0.10	0.10
4	2	0.26	0.021	0.021	0.54	0.04	0.04
4	3	0.37	0.030	0.030	1.17	0.09	0.09
4	4	0.21	0.017	0.017	1.72	0.14	0.14
4	5	0.42	0.034	0.034	3.52	0.28	0.28
4	6	0.42	0.034	0.034	3.25	0.26	0.26
4	7	1.19	0.095	0.095	4.79	0.38	0.38
4	8	0.73	0.058	0.058	5.26	0.42	0.42
5	1	0.14	0.011	0.011	0.15	0.01	0.01
5	2	0.16	0.013	0.013	0.17	0.01	0.01
5	3	0.22	0.018	0.018	0.41	0.03	0.03
5	4	0.16	0.013	0.013	0.46	0.04	0.04
5	5	0.23	0.018	0.018	0.77	0.06	0.06
5	6	0.24	0.019	0.019	0.47	0.04	0.04
5	7	0.59	0.047	0.047	0.74	0.06	0.06
5	8	0.46	0.037	0.037	0.66	0.05	0.05
			∑ min	∑ max		∑ min	∑ max
1			0.312	0.312		0.30	0.30
2			0.461	0.461		0.40	0.40
3			0.384	0.384		0.35	0.35
4			0.300	0.300		1.71	1.71
5			0.176	0.176		0.31	0.31
Mean			0.327	0.327		0.62	0.62
SD			0.106	0.106		0.62	0.62

Table G. Continued CUS.

Stone	Fraction	LAB 2 CUS2 Co µg/l	min r mg/m²	max r mg/m²	LAB 2 CUS3 Co µg/l	min r mg/m²	max r mg/m²
1	1	0.33	0.026	0.026			
1	2	0.20	0.016	0.016			
1	3	<0.20	0.000	0.016			
1	4	<0.20	0.000	0.016			
1	5	0.22	0.017	0.017			
1	6	0.20	0.016	0.016			
1	7	0.38	0.030	0.030			
1	8	0.31	0.025	0.025			
2	1	0.59	0.048	0.048	0.11	0.009	0.009
2	2	0.30	0.024	0.024	0.11	0.009	0.009
2	3	0.32	0.026	0.026	0.10	0.008	0.008
2	4	0.35	0.028	0.028	0.10	0.008	0.008
2	5	0.60	0.048	0.048	0.15	0.012	0.012
2	6	0.45	0.036	0.036	0.18	0.014	0.014
2	7	1.84	0.147	0.147	0.28	0.022	0.022
2	8	2.12	0.170	0.170	0.29	0.023	0.023
3	1	0.31	0.025	0.025	0.11	0.009	0.009
3	2	0.26	0.021	0.021	0.08	0.006	0.006
3	3	0.34	0.027	0.027	0.07	0.006	0.006
3	4	0.36	0.028	0.028	0.08	0.007	0.007
3	5	0.38	0.030	0.030	0.17	0.014	0.014
3	6	0.39	0.031	0.031	0.20	0.016	0.016
3	7	0.70	0.056	0.056	0.34	0.027	0.027
3	8	0.57	0.046	0.046	0.35	0.028	0.028
4	1	0.30	0.024	0.024	0.08	0.006	0.006
4	2	<0.20	0.000	0.016	0.07	0.006	0.006
4	3	<0.20	0.000	0.016	0.07	0.005	0.005
4	4	<0.20	0.000	0.016	0.10	0.008	0.008
4	5	<0.20	0.000	0.016	0.18	0.014	0.014
4	6	<0.20	0.000	0.016	0.22	0.017	0.017
4	7	0.35	0.028	0.028	0.50	0.040	0.040
4	8	0.30	0.024	0.024	0.40	0.032	0.032
5	1	2.83	0.226	0.226	0.11	0.009	0.009
5	2	1.53	0.122	0.122	0.11	0.009	0.009
5	3	1.47	0.118	0.118	0.12	0.009	0.009
5	4	1.46	0.117	0.117	0.13	0.010	0.010
5	5	2.25	0.180	0.180	0.26	0.021	0.021
5	6	2.05	0.164	0.164	0.32	0.026	0.026
5	7	4.21	0.337	0.337	0.48	0.038	0.038
5	8	3.74	0.300	0.300	0.41	0.033	0.033
			∑ min	∑ max		∑ min	∑ max
1			0.131	0.163			
2			0.526	0.526		0.106	0.106
3			0.264	0.264		0.112	0.112
4			0.076	0.156		0.129	0.129
5			1.563	1.563		0.155	0.155
Mean			0.512	0.535		0.125	0.125
SD			0.613	0.594		0.022	0.022

Table G. Continued CUS.

Stone	Fraction	LAB 1 CUS1 Cr µg/l	min r mg/m^2	max r mg/m^2	LAB 2 CUS1 Cr µg/l	min r mg/m2	max r mg/m2
1	1	0.06	0.005	0.005	<0.09	0.000	0.007
1	2	0.08	0.007	0.007	<0.09	0.000	0.007
1	3	0.08	0.007	0.007	<0.09	0.000	0.007
1	4	<0.06	0.000	0.005	<0.09	0.000	0.007
1	5	0.08	0.006	0.006	<0.09	0.000	0.007
1	6	0.09	0.007	0.007	<0.09	0.000	0.007
1	7	<0.06	0.000	0.005	<0.09	0.000	0.007
1	8	<0.06	0.000	0.005	<0.09	0.000	0.007
2	1	0.10	0.008	0.008	<0.09	0.000	0.007
2	2	0.08	0.007	0.007	<0.09	0.000	0.007
2	3	<0.06	0.000	0.005	<0.09	0.000	0.007
2	4	0.07	0.006	0.006	<0.09	0.000	0.007
2	5	<0.06	0.000	0.005	<0.09	0.000	0.007
2	6	<0.06	0.000	0.005	<0.09	0.000	0.007
2	7	0.18	0.014	0.014	<0.09	0.000	0.007
2	8	<0.06	0.000	0.005	<0.09	0.000	0.007
3	1	0.10	0.008	0.008	<0.09	0.000	0.007
3	2	0.06	0.005	0.005	<0.09	0.000	0.007
3	3	0.08	0.006	0.006	<0.09	0.000	0.007
3	4	<0.06	0.000	0.005	<0.09	0.000	0.007
3	5	<0.06	0.000	0.005	<0.09	0.000	0.007
3	6	0.08	0.006	0.006	<0.09	0.000	0.007
3	7	<0.06	0.000	0.005	<0.09	0.000	0.007
3	8	<0.06	0.000	0.005	<0.09	0.000	0.007
4	1	0.13	0.010	0.010	<0.09	0.000	0.007
4	2	0.06	0.005	0.005	<0.09	0.000	0.007
4	3	0.07	0.005	0.005	<0.09	0.000	0.007
4	4	0.06	0.004	0.004	<0.09	0.000	0.007
4	5	<0.06	0.000	0.005	<0.09	0.000	0.007
4	6	<0.06	0.000	0.005	<0.09	0.007	0.007
4	7	<0.06	0.000	0.005	<0.09	0.000	0.007
4	8	<0.06	0.000	0.005	<0.09	0.000	0.007
5	1	0.12	0.009	0.009	<0.09	0.000	0.007
5	2	0.06	0.005	0.005	<0.09	0.000	0.007
5	3	0.07	0.005	0.005	<0.09	0.000	0.007
5	4	0.06	0.005	0.005	<0.09	0.000	0.007
5	5	0.07	0.006	0.006	<0.09	0.000	0.007
5	6	0.07	0.006	0.006	<0.09	0.000	0.007
5	7	<0.06	0.000	0.005	<0.09	0.000	0.007
5	8	0.09	0.007	0.007	<0.09	0.000	0.007
			∑ min	∑ max		∑ min	∑ max
1			0.031	0.046		0.000	0.057
2			0.035	0.054		0.000	0.058
3			0.026	0.045		0.000	0.058
4			0.025	0.044		0.007	0.058
5			0.043	0.048		0.000	0.058
Mean			0.032	0.047		0.001	0.058
SD			0.007	0.004		0.003	0.000

Table G. Continued CUS.

Stone	Fraction	LAB 2 CUS2 Cr µg/l	min r mg/m²	max r mg/m²	LAB 2 CUS3 Cr µg/l	min r mg/m²	max r mg/m²
1	1	<0.21	0.000	0.017			
1	2	<0.21	0.000	0.017			
1	3	<0.21	0.000	0.017			
1	4	<0.21	0.000	0.017			
1	5	<0.21	0.000	0.017			
1	6	<0.21	0.000	0.017			
1	7	<0.21	0.000	0.017			
1	8	<0.21	0.000	0.017			
2	1	<0.21	0.000	0.017	<0.09	0.000	0.007
2	2	<0.21	0.000	0.017	<0.09	0.000	0.007
2	3	<0.21	0.000	0.017	<0.09	0.000	0.007
2	4	<0.21	0.000	0.017	<0.09	0.000	0.007
2	5	<0.21	0.000	0.017	<0.09	0.000	0.007
2	6	<0.21	0.000	0.017	<0.09	0.000	0.007
2	7	<0.21	0.000	0.017	<0.09	0.000	0.007
2	8	<0.21	0.000	0.017	<0.09	0.000	0.007
3	1	<0.21	0.000	0.017	<0.09	0.000	0.007
3	2	<0.21	0.000	0.017	<0.09	0.000	0.007
3	3	<0.21	0.000	0.017	<0.09	0.000	0.007
3	4	<0.21	0.000	0.017	<0.09	0.000	0.007
3	5	<0.21	0.000	0.017	<0.09	0.000	0.007
3	6	<0.21	0.000	0.017	<0.09	0.000	0.007
3	7	<0.21	0.000	0.017	<0.09	0.000	0.007
3	8	<0.21	0.000	0.017	<0.09	0.000	0.007
4	1	<0.21	0.000	0.017	<0.09	0.000	0.007
4	2	<0.21	0.000	0.017	<0.09	0.000	0.007
4	3	<0.21	0.000	0.017	<0.09	0.000	0.007
4	4	<0.21	0.000	0.017	<0.09	0.000	0.007
4	5	<0.21	0.000	0.017	<0.09	0.000	0.007
4	6	<0.21	0.000	0.017	<0.09	0.000	0.007
4	7	<0.21	0.000	0.017	<0.09	0.000	0.007
4	8	<0.21	0.000	0.017	<0.09	0.000	0.007
5	1	<0.21	0.000	0.017	<0.09	0.000	0.007
5	2	<0.21	0.000	0.017	<0.09	0.000	0.007
5	3	<0.21	0.000	0.017	<0.09	0.000	0.007
5	4	<0.21	0.000	0.017	<0.09	0.000	0.007
5	5	<0.21	0.000	0.017	<0.09	0.000	0.007
5	6	<0.21	0.000	0.017	<0.09	0.000	0.007
5	7	<0.21	0.000	0.017	<0.09	0.000	0.007
5	8	<0.21	0.000	0.017	<0.09	0.000	0.007
			∑ min	∑ max		∑ min	∑ max
1			0.000	0.134			
2			0.000	0.134		0.000	0.058
3			0.000	0.134		0.000	0.058
4			0.000	0.134		0.000	0.058
5			0.000	0.134		0.000	0.058
Mean			0.000	0.134		0.000	0.058
SD			0.000	0.000		0.000	0.000

Table G. Continued CUS.

Stone	Fraction	LAB 1 CUS1 Cu µg/l	min r mg/m^2	max r mg/m^2	LAB 2 CUS1 Cu µg/l	min r mg/m2	max r mg/m2
1	1	8.88	0.71	0.71	27.16	2.17	2.17
1	2	7.97	0.64	0.64	7.14	0.57	0.57
1	3	8.59	0.69	0.69	18.51	1.48	1.48
1	4	15.25	1.22	1.22	36.96	2.95	2.95
1	5	21.39	1.71	1.71	79.90	6.38	6.38
1	6	17.84	1.43	1.43	89.25	7.13	7.13
1	7	49.50	3.96	3.96	105.67	8.44	8.44
1	8	27.40	2.19	2.19	105.42	8.42	8.42
2	1	13.59	1.09	1.09	4.36	0.35	0.35
2	2	9.35	0.75	0.75	2.43	0.19	0.19
2	3	5.04	0.40	0.40	6.81	0.54	0.54
2	4	13.52	1.08	1.08	17.58	1.41	1.41
2	5	22.81	1.82	1.82	64.18	5.13	5.13
2	6	14.67	1.17	1.17	94.70	7.57	7.57
2	7	44.42	3.55	3.55	129.76	10.38	10.38
2	8	34.97	2.80	2.80	143.47	11.47	11.47
3	1	15.17	1.21	1.21	3.36	0.27	0.27
3	2	13.00	1.04	1.04	5.30	0.42	0.42
3	3	17.60	1.41	1.41	23.45	1.88	1.88
3	4	18.87	1.51	1.51	42.81	3.42	3.42
3	5	16.81	1.35	1.35	85.30	6.82	6.82
3	6	37.00	2.96	2.96	85.68	6.85	6.85
3	7	36.13	2.89	2.89	112.71	9.01	9.01
3	8	16.94	1.35	1.35	118.85	9.50	9.50
4	1	10.61	0.85	0.85	4.41	0.35	0.35
4	2	6.32	0.51	0.51	2.87	0.23	0.23
4	3	8.65	0.69	0.69	5.42	0.43	0.43
4	4	14.06	1.12	1.12	15.76	1.26	1.26
4	5	14.80	1.18	1.18	50.68	4.05	4.05
4	6	22.31	1.78	1.78	97.63	7.80	7.80
4	7	22.64	1.81	1.81	153.73	12.28	12.28
4	8	13.94	1.11	1.11	169.83	13.57	13.57
5	1	44.11	3.53	3.53	5.21	0.42	0.42
5	2	9.84	0.79	0.79	2.51	0.20	0.20
5	3	7.90	0.63	0.63	9.78	0.78	0.78
5	4	19.66	1.57	1.57	24.85	1.99	1.99
5	5	25.08	2.01	2.01	77.46	6.20	6.20
5	6	21.75	1.74	1.74	80.32	6.43	6.43
5	7	42.78	3.42	3.42	105.56	8.45	8.45
5	8	38.75	3.10	3.10	113.81	9.11	9.11
			∑ min	∑ max		∑ min	∑ max
1			12.54	12.54		37.53	37.53
2			12.67	12.67		37.05	37.05
3			13.72	13.72		38.18	38.18
4			9.06	9.06		39.98	39.98
5			16.79	16.79		33.59	33.59
Mean			12.96	12.96		37.27	37.27
SD			2.77	2.77		2.34	2.34

Table G. Continued CUS.

Stone	Fraction	LAB 2 CUS2 Cu µg/l	min r mg/m²	max r mg/m²	LAB 2 CUS3 Cu µg/l	min r mg/m²	max r mg/m²
1	1	92.4	7.39	7.39			
1	2	48.0	3.84	3.84			
1	3	61.4	4.91	4.91			
1	4	71.3	5.70	5.70			
1	5	116	9.25	9.25			
1	6	141	11.31	11.31			
1	7	354	28.34	28.34			
1	8	374	29.89	29.89			
2	1	51.2	4.09	4.09	14.7	1.18	1.18
2	2	34.3	2.74	2.74	34.8	2.79	2.79
2	3	32.1	2.57	2.57	37.3	2.98	2.98
2	4	40.5	3.24	3.24	43.4	3.48	3.48
2	5	61.1	4.89	4.89	71.4	5.72	5.72
2	6	89.2	7.14	7.14	82.8	6.64	6.64
2	7	229	18.34	18.34	115.4	9.24	9.24
2	8	257	20.57	20.57	124.9	10.01	10.01
3	1	92.0	7.35	7.35	62.4	4.99	4.99
3	2	29.3	2.34	2.34	52.3	4.19	4.19
3	3	40.1	3.20	3.20	58.7	4.70	4.70
3	4	70.4	5.63	5.63	66.1	5.29	5.29
3	5	136	10.91	10.91	141.0	11.29	11.29
3	6	136	10.84	10.84	136.8	10.95	10.95
3	7	280	22.37	22.37	180.4	14.44	14.44
3	8	349	27.93	27.93	199.7	15.99	15.99
4	1	120.3	9.63	9.63	36.0	2.88	2.88
4	2	37.4	2.99	2.99	65.1	5.21	5.21
4	3	41.9	3.35	3.35	62.5	5.00	5.00
4	4	55.6	4.45	4.45	92.7	7.41	7.41
4	5	74.2	5.94	5.94	157.4	12.58	12.58
4	6	100	8.00	8.00	175.6	14.04	14.04
4	7	227	18.19	18.19	336.5	26.90	26.90
4	8	287	22.95	22.95	316.0	25.26	25.26
5	1	169	13.51	13.51	41.9	3.35	3.35
5	2	67.5	5.40	5.40	62.1	4.97	4.97
5	3	74.4	5.96	5.96	67.4	5.39	5.39
5	4	86.0	6.88	6.88	70.9	5.67	5.67
5	5	141	11.32	11.32	144.6	11.57	11.57
5	6	165	13.22	13.22	181.9	14.55	14.55
5	7	430	34.43	34.43	223.8	17.91	17.91
5	8	680	54.40	54.40	234.2	18.74	18.74
			∑ min	∑ max		∑ min	∑ max
1			100.6	100.6			
2			63.6	63.6		42.04	42.04
3			90.6	90.6		71.85	71.85
4			75.5	75.5		99.27	99.27
5			145.1	145.1		82.14	82.14
Mean			95.1	95.1		73.82	73.82
SD			31.3	31.3		24.02	24.02

Table G. Continued CUS.

Stone	Fraction	LAB 1 CUS1 Mn µg/l	min r mg/m^2	max r mg/m^2	LAB 2 CUS1 Mn µg/l	min r mg/m^2	max r mg/m^2
1	1	0.45	0.036	0.036	0.648	0.052	0.052
1	2	0.73	0.058	0.058	0.612	0.049	0.049
1	3	0.74	0.059	0.059	0.827	0.066	0.066
1	4	0.38	0.030	0.030	0.861	0.069	0.069
1	5	0.71	0.056	0.056	1.278	0.102	0.102
1	6	1.07	0.085	0.085	0.989	0.079	0.079
1	7	1.73	0.139	0.139	1.041	0.083	0.083
1	8	1.06	0.085	0.085	0.853	0.068	0.068
2	1	0.65	0.052	0.052	0.842	0.067	0.067
2	2	1.28	0.102	0.102	1.076	0.086	0.086
2	3	1.57	0.125	0.125	1.814	0.145	0.145
2	4	1.31	0.105	0.105	2.124	0.170	0.170
2	5	1.59	0.127	0.127	3.713	0.297	0.297
2	6	1.42	0.114	0.114	2.439	0.195	0.195
2	7	3.76	0.301	0.301	2.789	0.223	0.223
2	8	2.44	0.195	0.195	1.792	0.143	0.143
3	1	0.98	0.078	0.078	0.642	0.051	0.051
3	2	1.29	0.104	0.104	1.032	0.083	0.083
3	3	1.40	0.112	0.112	1.870	0.150	0.150
3	4	1.44	0.115	0.115	1.541	0.123	0.123
3	5	1.85	0.148	0.148	2.325	0.186	0.186
3	6	1.74	0.139	0.139	1.470	0.118	0.118
3	7	3.39	0.271	0.271	1.946	0.156	0.156
3	8	1.63	0.130	0.130	1.854	0.148	0.148
4	1	0.53	0.043	0.043	0.746	0.060	0.060
4	2	0.67	0.053	0.053	0.378	0.030	0.030
4	3	0.96	0.077	0.077	0.667	0.053	0.053
4	4	0.56	0.045	0.045	1.027	0.082	0.082
4	5	0.81	0.065	0.065	2.198	0.176	0.176
4	6	0.87	0.069	0.069	2.116	0.169	0.169
4	7	1.88	0.150	0.150	2.499	0.200	0.200
4	8	0.98	0.078	0.078	2.226	0.178	0.178
5	1	1.59	0.128	0.128	0.796	0.064	0.064
5	2	0.86	0.069	0.069	0.459	0.037	0.037
5	3	1.48	0.118	0.118	0.965	0.077	0.077
5	4	1.22	0.098	0.098	0.720	0.058	0.058
5	5	1.41	0.113	0.113	0.880	0.070	0.070
5	6	1.26	0.101	0.101	0.539	0.043	0.043
5	7	2.53	0.203	0.203	0.769	0.062	0.062
5	8	1.60	0.128	0.128	0.831	0.067	0.067
			∑ min	∑ max		∑ min	∑ max
1			0.549	0.549		0.568	0.568
2			1.121	1.121		1.327	1.327
3			1.099	1.099		1.014	1.014
4			0.581	0.581		0.948	0.948
5			0.956	0.956		0.477	0.477
Mean			0.861	0.861		0.867	0.867
SD			0.278	0.278		0.347	0.347

Table G. Continued CUS.

Stone	Fraction	LAB 2 CUS2 Mn µg/l	min r mg/m^2	max r mg/m^2	LAB 2 CUS3 Mn µg/l	min r mg/m^2	max r mg/m^2
1	1	2.19	0.175	0.175			
1	2	0.94	0.075	0.075			
1	3	0.72	0.058	0.058			
1	4	0.55	0.044	0.044			
1	5	0.68	0.054	0.054			
1	6	0.51	0.041	0.041			
1	7	0.89	0.071	0.071			
1	8	0.67	0.053	0.053			
2	1	1.32	0.106	0.106	11.1	0.89	0.89
2	2	0.71	0.057	0.057	15.7	1.26	1.26
2	3	0.63	0.051	0.051	17.2	1.38	1.38
2	4	0.66	0.053	0.053	19.2	1.54	1.54
2	5	1.08	0.086	0.086	32.9	2.64	2.64
2	6	3.35	0.268	0.268	36.6	2.93	2.93
2	7	2.66	0.213	0.213	54.8	4.39	4.39
2	8	2.50	0.200	0.200	49.6	3.97	3.97
3	1	2.48	0.198	0.198	32.0	2.56	2.56
3	2	3.16	0.253	0.253	37.0	2.96	2.96
3	3	3.15	0.252	0.252	40.8	3.27	3.27
3	4	2.92	0.234	0.234	50.8	4.07	4.07
3	5	2.63	0.211	0.211	96.6	7.73	7.73
3	6	2.56	0.205	0.205	99.8	7.99	7.99
3	7	4.01	0.321	0.321	148.8	11.92	11.92
3	8	3.41	0.273	0.273	132.4	10.60	10.60
4	1	1.87	0.149	0.149	21.7	1.73	1.73
4	2	0.70	0.056	0.056	32.7	2.61	2.61
4	3	0.50	0.040	0.040	34.4	2.75	2.75
4	4	0.41	0.033	0.033	53.9	4.31	4.31
4	5	0.51	0.041	0.041	95.6	7.64	7.64
4	6	0.40	0.032	0.032	88.2	7.05	7.05
4	7	0.75	0.060	0.060	132.2	10.57	10.57
4	8	0.67	0.053	0.053	86.0	6.88	6.88
5	1	4.89	0.391	0.391	36.2	2.90	2.90
5	2	2.60	0.208	0.208	38.7	3.10	3.10
5	3	2.24	0.179	0.179	40.3	3.22	3.22
5	4	1.95	0.156	0.156	43.5	3.48	3.48
5	5	3.10	0.248	0.248	82.3	6.59	6.59
5	6	2.25	0.180	0.180	81.8	6.54	6.54
5	7	4.13	0.330	0.330	90.0	7.20	7.20
5	8	4.06	0.325	0.325	65.9	5.28	5.28
			∑ min	∑ max		∑ min	∑ max
1			0.572	0.572			
2			1.033	1.033		19.00	19.0
3			1.946	1.946		51.10	51.1
4			0.465	0.465		43.55	43.5
5			2.019	2.019		38.30	38.3
Mean			1.207	1.207		37.99	37.99
SD			0.740	0.740		13.71	13.71

Table G. Continued CUS.

Stone	Fraction	LAB 1 CUS1 Mo µg/l	min r mg/m^2	max r mg/m^2	LAB 2 CUS1 Mo µg/l	min r mg/m^2	max r mg/m^2
1	1	2.73	0.219	0.219	1.27	0.101	0.101
1	2	1.28	0.103	0.103	0.95	0.076	0.076
1	3	0.57	0.046	0.046	0.94	0.075	0.075
1	4	0.50	0.040	0.040	1.05	0.084	0.084
1	5	0.81	0.065	0.065	2.11	0.169	0.169
1	6	0.98	0.079	0.079	2.19	0.175	0.175
1	7	2.29	0.183	0.183	4.58	0.365	0.365
1	8	2.11	0.169	0.169	5.26	0.420	0.420
2	1	1.75	0.140	0.140	1.20	0.096	0.096
2	2	1.10	0.088	0.088	0.95	0.076	0.076
2	3	1.20	0.096	0.096	0.83	0.066	0.066
2	4	1.04	0.083	0.083	0.84	0.067	0.067
2	5	1.59	0.127	0.127	1.71	0.137	0.137
2	6	1.97	0.157	0.157	1.65	0.132	0.132
2	7	4.41	0.353	0.353	3.54	0.283	0.283
2	8	3.72	0.298	0.298	4.21	0.337	0.337
3	1	2.76	0.220	0.220	2.03	0.163	0.163
3	2	1.89	0.152	0.152	1.06	0.085	0.085
3	3	2.03	0.162	0.162	0.68	0.054	0.054
3	4	1.79	0.143	0.143	0.68	0.055	0.055
3	5	2.86	0.229	0.229	1.33	0.107	0.107
3	6	3.52	0.281	0.281	1.28	0.103	0.103
3	7	7.16	0.573	0.573	2.68	0.214	0.214
3	8	5.70	0.456	0.456	3.13	0.250	0.250
4	1	1.44	0.115	0.115	2.60	0.208	0.208
4	2	0.91	0.073	0.073	2.78	0.222	0.222
4	3	0.81	0.064	0.064	2.03	0.162	0.162
4	4	0.67	0.053	0.053	1.76	0.141	0.141
4	5	1.09	0.087	0.087	2.90	0.232	0.232
4	6	1.39	0.111	0.111	2.42	0.193	0.193
4	7	3.05	0.244	0.244	4.44	0.355	0.355
4	8	2.54	0.203	0.203	4.94	0.395	0.395
5	1	1.41	0.112	0.112	2.60	0.208	0.208
5	2	1.20	0.096	0.096	1.96	0.157	0.157
5	3	1.13	0.090	0.090	1.03	0.082	0.082
5	4	1.09	0.087	0.087	0.88	0.071	0.071
5	5	1.69	0.135	0.135	1.60	0.128	0.128
5	6	2.10	0.168	0.168	1.43	0.115	0.115
5	7	4.57	0.366	0.366	3.08	0.246	0.246
5	8	3.76	0.301	0.301	3.745	0.300	0.300
			∑ min	∑ max		∑ min	∑ max
1			0.90	0.90		1.46	1.46
2			1.34	1.34		1.19	1.19
3			2.22	2.22		1.03	1.03
4			0.95	0.95		1.91	1.91
5			1.36	1.36		1.31	1.31
Mean			1.354	1.354		1.381	1.381
SD			0.527	0.527		0.335	0.335

Table G. Continued CUS.

Stone	Fraction	LAB 2 CUS2 Mo µg/l	min r mg/m^2	max r mg/m^2	LAB 2 CUS3 Mo µg/l	min r mg/m^2	max r mg/m^2
1	1	<0.31	0.000	0.025			
1	2	<0.31	0.000	0.025			
1	3	<0.31	0.000	0.025			
1	4	<0.31	0.000	0.025			
1	5	0.35	0.028	0.028			
1	6	0.40	0.032	0.032			
1	7	0.77	0.062	0.062			
1	8	0.75	0.060	0.060			
2	1	<0.31	0.000	0.025	0.93	0.074	0.074
2	2	<0.31	0.000	0.025	0.80	0.064	0.064
2	3	<0.31	0.000	0.025	0.84	0.067	0.067
2	4	<0.31	0.000	0.025	0.94	0.075	0.075
2	5	<0.31	0.000	0.025	1.72	0.138	0.138
2	6	0.34	0.027	0.027	1.74	0.139	0.139
2	7	<0.31	0.000	0.025	3.41	0.273	0.273
2	8	<0.31	0.000	0.025	2.66	0.213	0.213
3	1	0.53	0.043	0.043	0.93	0.075	0.075
3	2	0.44	0.035	0.035	0.86	0.069	0.069
3	3	<0.31	0.000	0.025	1.04	0.083	0.083
3	4	<0.31	0.000	0.025	1.37	0.110	0.110
3	5	0.35	0.028	0.028	2.56	0.205	0.205
3	6	0.32	0.026	0.026	2.97	0.238	0.238
3	7	0.58	0.047	0.047	6.27	0.502	0.502
3	8	0.51	0.041	0.041	5.71	0.457	0.457
4	1	0.32	0.025	0.025	0.94	0.075	0.075
4	2	0.31	0.025	0.025	1.07	0.085	0.085
4	3	<0.31	0.000	0.025	1.13	0.090	0.090
4	4	<0.31	0.000	0.025	1.36	0.109	0.109
4	5	0.58	0.046	0.046	2.54	0.203	0.203
4	6	0.55	0.044	0.044	2.40	0.192	0.192
4	7	0.94	0.075	0.075	4.57	0.366	0.366
4	8	0.70	0.056	0.056	3.97	0.317	0.317
5	1	<0.31	0.000	0.025	1.23	0.099	0.099
5	2	<0.31	0.000	0.025	0.80	0.064	0.064
5	3	<0.31	0.000	0.025	0.82	0.066	0.066
5	4	<0.31	0.000	0.025	0.85	0.068	0.068
5	5	<0.31	0.000	0.025	1.53	0.122	0.122
5	6	<0.31	0.000	0.025	1.59	0.127	0.127
5	7	0.43	0.034	0.034	3.03	0.243	0.243
5	8	0.43	0.034	0.034	2.51	0.201	0.201
			∑ min	∑ max		∑ min	∑ max
1			0.182	0.281			
2			0.027	0.201		1.044	1.044
3			0.219	0.269		1.739	1.739
4			0.271	0.321		1.438	1.438
5			0.068	0.217		0.989	0.989
Mean			0.154	0.258		1.303	1.303
SD			0.103	0.049		0.35	0.35

Table G. Continued CUS.

Stone	Fraction	LAB 1 CUS1 Ni µg/l	min r mg/m^2	max r mg/m^2	LAB 2 CUS1 Ni µg/l	min r mg/m^2	max r mg/m^2
1	1	0.55	0.044	0.044	1.70	0.136	0.136
1	2	0.74	0.060	0.060	1.26	0.101	0.101
1	3	1.26	0.101	0.101	2.22	0.178	0.178
1	4	0.99	0.079	0.079	2.42	0.193	0.193
1	5	2.28	0.182	0.182	4.20	0.335	0.335
1	6	2.91	0.233	0.233	3.35	0.267	0.267
1	7	13.60	1.088	1.088	4.29	0.342	0.342
1	8	8.65	0.692	0.692	3.56	0.284	0.284
2	1	0.99	0.079	0.079	0.89	0.071	0.071
2	2	1.21	0.097	0.097	0.96	0.077	0.077
2	3	1.97	0.158	0.158	2.22	0.178	0.178
2	4	1.87	0.150	0.150	2.93	0.234	0.234
2	5	3.15	0.252	0.252	6.51	0.520	0.520
2	6	3.49	0.279	0.279	5.83	0.466	0.466
2	7	11.41	0.912	0.912	8.86	0.709	0.709
2	8	8.59	0.687	0.687	7.22	0.577	0.577
3	1	0.90	0.072	0.072	0.50	0.040	0.040
3	2	1.04	0.083	0.083	1.04	0.083	0.083
3	3	1.16	0.093	0.093	2.62	0.210	0.210
3	4	1.18	0.094	0.094	2.71	0.217	0.217
3	5	2.04	0.163	0.163	5.42	0.433	0.433
3	6	2.11	0.169	0.169	4.16	0.332	0.332
3	7	5.06	0.405	0.405	6.78	0.542	0.542
3	8	2.41	0.193	0.193	6.53	0.522	0.522
4	1	0.78	0.062	0.062	4.06	0.324	0.324
4	2	0.97	0.077	0.077	1.95	0.156	0.156
4	3	1.55	0.124	0.124	4.28	0.342	0.342
4	4	0.99	0.079	0.079	8.23	0.658	0.658
4	5	2.34	0.187	0.187	20.70	1.654	1.654
4	6	2.38	0.191	0.191	21.14	1.689	1.689
4	7	7.01	0.561	0.561	28.88	2.308	2.308
4	8	3.70	0.296	0.296	27.94	2.233	2.233
5	1	1.14	0.091	0.091	0.72	0.057	0.057
5	2	0.59	0.047	0.047	0.57	0.046	0.046
5	3	0.93	0.074	0.074	1.52	0.121	0.121
5	4	0.76	0.061	0.061	2.13	0.170	0.170
5	5	1.23	0.098	0.098	4.45	0.356	0.356
5	6	1.28	0.103	0.103	2.97	0.238	0.238
5	7	4.20	0.336	0.336	4.83	0.387	0.387
5	8	2.36	0.188	0.188	4.31	0.345	0.345
			∑ min	∑ max		∑ min	∑ max
1			2.478	2.478		1.837	1.837
2			2.615	2.615		2.833	2.833
3			1.272	1.272		2.380	2.380
4			1.578	1.578		9.365	9.365
5			0.999	0.999		1.721	1.721
Mean			1.788	1.788		3.627	3.627
SD			0.723	0.723		3.238	3.238

Table G. Continued CUS.

		LAB 2 CUS2	min	max	LAB 2 CUS3	min	max
		Ni	r	r	Ni	r	r
Stone	Fraction	µg/l	mg/m^2	mg/m^2	µg/l	mg/m^2	mg/m^2
1	1	6.09	0.487	0.487			
1	2	3.71	0.297	0.297			
1	3	3.31	0.265	0.265			
1	4	3.27	0.261	0.261			
1	5	4.72	0.377	0.377			
1	6	4.44	0.355	0.355			
1	7	8.50	0.679	0.679			
1	8	7.80	0.623	0.623			
2	1	6.95	0.556	0.556	0.94	0.075	0.075
2	2	3.53	0.282	0.282	1.03	0.082	0.082
2	3	3.16	0.253	0.253	1.04	0.083	0.083
2	4	3.55	0.284	0.284	1.12	0.089	0.089
2	5	5.80	0.464	0.464	1.61	0.129	0.129
2	6	8.03	0.642	0.642	1.50	0.120	0.120
2	7	15.6	1.247	1.247	2.25	0.180	0.180
2	8	17.8	1.425	1.425	1.69	0.135	0.135
3	1	6.32	0.506	0.506	3.60	0.288	0.288
3	2	4.29	0.343	0.343	2.62	0.209	0.209
3	3	5.22	0.417	0.417	3.04	0.243	0.243
3	4	5.92	0.474	0.474	3.88	0.311	0.311
3	5	8.11	0.649	0.649	26.70	2.138	2.138
3	6	8.17	0.653	0.653	8.29	0.664	0.664
3	7	17.5	1.395	1.395	8.43	0.675	0.675
3	8	17.4	1.394	1.394	5.16	0.413	0.413
4	1	5.03	0.403	0.403	1.67	0.133	0.133
4	2	2.32	0.185	0.185	1.83	0.146	0.146
4	3	2.01	0.161	0.161	1.66	0.133	0.133
4	4	2.00	0.160	0.160	5.40	0.431	0.431
4	5	3.00	0.240	0.240	6.31	0.505	0.505
4	6	3.02	0.242	0.242	2.77	0.221	0.221
4	7	6.90	0.552	0.552	3.64	0.291	0.291
4	8	7.23	0.579	0.579	2.39	0.191	0.191
5	1	21.3	1.703	1.703	2.28	0.183	0.183
5	2	13.1	1.048	1.048	2.15	0.172	0.172
5	3	13.1	1.046	1.046	2.22	0.178	0.178
5	4	13.9	1.115	1.115	2.69	0.215	0.215
5	5	24.1	1.927	1.927	4.44	0.355	0.355
5	6	23.4	1.873	1.873	3.70	0.296	0.296
5	7	51.9	4.156	4.156	4.28	0.343	0.343
5	8	57.9	4.629	4.629	2.95	0.236	0.236
			∑ min	∑ max		∑ min	∑ max
1			3.345	3.345			
2			5.153	5.153		0.895	0.895
3			5.831	5.831		4.942	4.942
4			2.522	2.522		2.053	2.053
5			17.496	17.496		1.978	1.978
Mean			6.870	6.870		2.467	2.467
SD			6.088	6.088		1.733	1.733

Table G. Continued CUS.

Stone	Fraction	LAB 1 CUS1 Pb µg/l	min r mg/m^2	max r mg/m^2	LAB 2 CUS1 Pb µg/l	min r mg/m2	max r mg/m2
1	1	0.22	0.018	0.018	0.14	0.011	0.011
1	2	0.14	0.011	0.011	0.13	0.010	0.010
1	3	0.21	0.017	0.017	0.21	0.017	0.017
1	4	0.16	0.013	0.013	0.25	0.020	0.020
1	5	0.27	0.022	0.022	0.42	0.033	0.033
1	6	0.29	0.023	0.023	0.49	0.039	0.039
1	7	4.57	0.365	0.365	0.49	0.039	0.039
1	8	1.00	0.080	0.080	0.46	0.037	0.037
2	1	0.20	0.016	0.016	0.18	0.014	0.014
2	2	0.11	0.009	0.009	0.11	0.009	0.009
2	3	0.10	0.008	0.008	0.13	0.010	0.010
2	4	0.12	0.009	0.009	0.22	0.017	0.017
2	5	0.19	0.015	0.015	0.75	0.060	0.060
2	6	0.24	0.019	0.019	1.37	0.110	0.110
2	7	3.93	0.315	0.315	1.93	0.154	0.154
2	8	0.95	0.076	0.076	2.26	0.181	0.181
3	1	0.28	0.022	0.022	0.13	0.010	0.010
3	2	0.07	0.006	0.006	0.28	0.022	0.022
3	3	0.08	0.006	0.006	0.73	0.058	0.058
3	4	0.07	0.005	0.005	1.18	0.094	0.094
3	5	0.06	0.005	0.005	2.80	0.224	0.224
3	6	0.08	0.006	0.006	3.16	0.253	0.253
3	7	0.22	0.018	0.018	2.82	0.226	0.226
3	8	0.05	0.004	0.004	2.73	0.218	0.218
4	1	0.33	0.027	0.027	0.21	0.017	0.017
4	2	0.10	0.008	0.008	0.33	0.026	0.026
4	3	0.09	0.007	0.007	0.36	0.029	0.029
4	4	0.11	0.009	0.009	0.51	0.041	0.041
4	5	0.11	0.009	0.009	1.26	0.101	0.101
4	6	0.15	0.012	0.012	2.18	0.174	0.174
4	7	1.87	0.150	0.150	2.86	0.228	0.228
4	8	0.78	0.062	0.062	3.36	0.268	0.268
5	1	0.30	0.024	0.024	0.21	0.017	0.017
5	2	0.08	0.006	0.006	0.19	0.015	0.015
5	3	0.09	0.007	0.007	0.27	0.022	0.022
5	4	0.08	0.006	0.006	0.50	0.040	0.040
5	5	0.11	0.009	0.009	2.01	0.161	0.161
5	6	0.09	0.007	0.007	2.58	0.207	0.207
5	7	0.80	0.064	0.064	3.14	0.251	0.251
5	8	0.47	0.038	0.038	3.20	0.256	0.256
			∑ min	∑ max		∑ min	∑ max
1			0.549	0.549		0.206	0.206
2			0.467	0.467		0.556	0.556
3			0.072	0.072		1.105	1.105
4			0.283	0.283		0.884	0.884
5			0.162	0.162		0.969	0.969
Mean			0.307	0.307		0.744	0.744
SD			0.201	0.201		0.362	0.362

Table G. Continued CUS.

Stone	Fraction	LAB 2 CUS2 Pb µg/l	min r mg/m^2	max r mg/m^2	LAB 2 CUS3 Pb µg/l	min r mg/m^2	max r mg/m^2
1	1	4.2	0.34	0.34			
1	2	8.2	0.65	0.65			
1	3	11.9	0.95	0.95			
1	4	15.9	1.27	1.27			
1	5	23.4	1.87	1.87			
1	6	30.0	2.40	2.40			
1	7	64.6	5.17	5.17			
1	8	72.4	5.79	5.79			
2	1	17.6	1.41	1.41	0.38	0.030	0.030
2	2	24.6	1.97	1.97	0.34	0.027	0.027
2	3	32.8	2.62	2.62	0.38	0.030	0.030
2	4	46.2	3.70	3.70	0.43	0.035	0.035
2	5	79.9	6.40	6.40	0.63	0.051	0.051
2	6	14.4	1.15	1.15	1.05	0.084	0.084
2	7	298.3	23.86	23.86	1.93	0.155	0.155
2	8	321.1	25.68	25.68	4.70	0.377	0.377
3	1	3.2	0.26	0.26	0.17	0.013	0.013
3	2	3.4	0.27	0.27	1.11	0.089	0.089
3	3	4.6	0.37	0.37	0.20	0.016	0.016
3	4	10.0	0.80	0.80	0.15	0.012	0.012
3	5	24.5	1.96	1.96	0.38	0.030	0.030
3	6	24.5	1.96	1.96	0.36	0.029	0.029
3	7	50.4	4.03	4.03	0.54	0.043	0.043
3	8	68.2	5.46	5.46	0.69	0.055	0.055
4	1	6.1	0.49	0.49	0.26	0.020	0.020
4	2	6.9	0.56	0.56	0.20	0.016	0.016
4	3	9.6	0.77	0.77	0.19	0.015	0.015
4	4	14.5	1.16	1.16	0.23	0.018	0.018
4	5	18.5	1.48	1.48	0.86	0.069	0.069
4	6	28.0	2.24	2.24	0.35	0.028	0.028
4	7	49.9	3.99	3.99	0.92	0.074	0.074
4	8	67.2	5.38	5.38	1.18	0.094	0.094
5	1	10.0	0.80	0.80	0.23	0.018	0.018
5	2	10.4	0.83	0.83	0.17	0.014	0.014
5	3	13.3	1.06	1.06	0.25	0.020	0.020
5	4	17.7	1.41	1.41	0.16	0.012	0.012
5	5	21.8	1.75	1.75	0.13	0.010	0.010
5	6	29.4	2.35	2.35	0.46	0.037	0.037
5	7	49.0	3.92	3.92	0.55	0.044	0.044
5	8	66.3	5.31	5.31	1.12	0.090	0.090
			∑ min	∑ max		∑ min	∑ max
1			18.43	18.43			
2			66.79	66.79		0.789	0.789
3			15.10	15.10		0.289	0.289
4			16.08	16.08		0.335	0.335
5			17.43	17.43		0.245	0.245
Mean			26.77	26.77		0.415	0.415
SD			22.41	22.41		0.253	0.253

Table G. Continued CUS.

Stone	Fraction	LAB 1 CUS1 Sb µg/l	min r mg/m^2	max r mg/m^2	LAB 2 CUS1 Sb µg/l	min r mg/m2	max r mg/m2
1	1	0.10	0.008	0.008	<0.45	0.000	0.036
1	2	0.14	0.011	0.011	<0.45	0.000	0.036
1	3	0.19	0.015	0.015	<0.45	0.000	0.036
1	4	0.19	0.015	0.015	<0.45	0.000	0.036
1	5	0.35	0.028	0.028	<0.45	0.000	0.036
1	6	0.39	0.031	0.031	<0.45	0.000	0.036
1	7	0.98	0.079	0.079	<0.45	0.000	0.036
1	8	0.60	0.048	0.048	<0.45	0.000	0.036
2	1	0.13	0.011	0.011	<0.45	0.000	0.036
2	2	0.16	0.013	0.013	<0.45	0.000	0.036
2	3	0.25	0.020	0.020	<0.45	0.000	0.036
2	4	0.31	0.025	0.025	<0.45	0.000	0.036
2	5	0.46	0.037	0.037	<0.45	0.000	0.036
2	6	0.49	0.040	0.040	<0.45	0.000	0.036
2	7	1.07	0.086	0.086	0.46	0.037	0.037
2	8	0.78	0.062	0.062	<0.45	0.000	0.036
3	1	0.11	0.009	0.009	<0.45	0.000	0.036
3	2	0.13	0.010	0.010	<0.45	0.000	0.036
3	3	0.17	0.014	0.014	<0.45	0.000	0.036
3	4	0.21	0.016	0.016	<0.45	0.000	0.036
3	5	0.34	0.027	0.027	<0.45	0.000	0.036
3	6	0.41	0.033	0.033	<0.45	0.000	0.036
3	7	1.02	0.082	0.082	<0.45	0.000	0.036
3	8	0.67	0.054	0.054	<0.45	0.000	0.036
4	1	0.11	0.009	0.009	<0.45	0.000	0.036
4	2	0.14	0.011	0.011	<0.45	0.000	0.036
4	3	0.19	0.015	0.015	<0.45	0.000	0.036
4	4	0.19	0.015	0.015	<0.45	0.000	0.036
4	5	0.29	0.024	0.024	<0.45	0.000	0.036
4	6	0.37	0.030	0.030	<0.45	0.000	0.036
4	7	0.90	0.072	0.072	0.48	0.038	0.038
4	8	0.64	0.051	0.051	0.49	0.039	0.039
5	1	0.14	0.011	0.011	<0.45	0.000	0.036
5	2	0.14	0.012	0.012	<0.45	0.000	0.036
5	3	0.21	0.017	0.017	<0.45	0.000	0.036
5	4	0.21	0.016	0.016	<0.45	0.000	0.036
5	5	0.32	0.025	0.025	<0.45	0.000	0.036
5	6	0.38	0.030	0.030	<0.45	0.000	0.036
5	7	0.83	0.066	0.066	<0.45	0.000	0.036
5	8	0.68	0.054	0.054	<0.45	0.000	0.036
			∑ min	∑ max		∑ min	∑ max
1			0.235	0.235		0.000	0.287
2			0.293	0.293		0.037	0.289
3			0.246	0.246		0.000	0.288
4			0.226	0.226		0.077	0.293
5			0.232	0.232		0.000	0.288
Mean			0.247	0.247		0.023	0.289
SD			0.027	0.027		0.034	0.002

Table G. Continued CUS.

Stone	Fraction	LAB 2 CUS2 Sb µg/l	min r mg/m^2	max r mg/m^2	LAB 2 CUS3 Sb µg/l	min r mg/m^2	max r mg/m^2
1	1	<0.37	0.000	0.030			
1	2	<0.37	0.000	0.030			
1	3	<0.37	0.000	0.030			
1	4	<0.37	0.000	0.030			
1	5	0.43	0.034	0.034			
1	6	0.48	0.038	0.038			
1	7	0.67	0.054	0.054			
1	8	0.56	0.045	0.045			
2	1	<0.37	0.000	0.030	<0.46	0.000	0.037
2	2	<0.37	0.000	0.030	<0.46	0.000	0.037
2	3	<0.37	0.000	0.030	<0.46	0.000	0.037
2	4	<0.37	0.000	0.030	<0.46	0.000	0.037
2	5	0.43	0.034	0.034	<0.46	0.000	0.037
2	6	0.42	0.033	0.033	<0.46	0.000	0.037
2	7	0.90	0.072	0.072	<0.46	0.000	0.037
2	8	0.88	0.071	0.071	<0.46	0.000	0.037
3	1	<0.37	0.000	0.030	<0.46	0.000	0.037
3	2	<0.37	0.000	0.030	<0.46	0.000	0.037
3	3	<0.37	0.000	0.030	<0.46	0.000	0.037
3	4	<0.37	0.000	0.030	<0.46	0.000	0.037
3	5	0.43	0.035	0.035	<0.46	0.000	0.037
3	6	0.43	0.034	0.034	<0.46	0.000	0.037
3	7	0.58	0.046	0.046	<0.46	0.000	0.037
3	8	0.64	0.051	0.051	<0.46	0.000	0.037
4	1	<0.37	0.000	0.030	<0.46	0.000	0.037
4	2	<0.37	0.000	0.030	<0.46	0.000	0.037
4	3	<0.37	0.000	0.030	<0.46	0.000	0.037
4	4	<0.37	0.000	0.030	<0.46	0.000	0.037
4	5	<0.37	0.000	0.030	<0.46	0.000	0.037
4	6	<0.37	0.000	0.030	<0.46	0.000	0.037
4	7	0.44	0.035	0.035	<0.46	0.000	0.037
4	8	0.41	0.033	0.033	<0.46	0.000	0.037
5	1	<0.37	0.000	0.030	<0.46	0.000	0.037
5	2	<0.37	0.000	0.030	<0.46	0.000	0.037
5	3	<0.37	0.000	0.030	<0.46	0.000	0.037
5	4	<0.37	0.000	0.030	<0.46	0.000	0.037
5	5	<0.37	0.000	0.030	<0.46	0.000	0.037
5	6	<0.37	0.000	0.030	<0.46	0.000	0.037
5	7	<0.37	0.000	0.030	<0.46	0.000	0.037
5	8	<0.37	0.000	0.030	<0.46	0.000	0.037
			∑ min	∑ max		∑ min	∑ max
1			0.171	0.290			
2			0.210	0.328		0.000	0.295
3			0.166	0.285		0.000	0.295
4			0.068	0.245		0.000	0.294
5			0.000	0.237		0.000	0.294
Mean			0.123	0.277		0.000	0.295
SD			0.09	0.04		0.000	0.000

Table G. Continued CUS.

Stone	Fraction	LAB 1 CUS1 Se µg/l	min r mg/m²	max r mg/m²	LAB 2 CUS1 Se µg/l	min r mg/m2	max r mg/m2
1	1	0.07	0.006	0.006	<0.26	0.000	0.021
1	2	<0.04	0.000	0.003	<0.26	0.000	0.021
1	3	<0.04	0.000	0.003	<0.26	0.000	0.021
1	4	<0.04	0.000	0.003	<0.26	0.000	0.021
1	5	<0.04	0.000	0.003	<0.26	0.000	0.021
1	6	<0.04	0.000	0.003	<0.26	0.000	0.021
1	7	0.06	0.005	0.005	<0.26	0.000	0.021
1	8	<0.04	0.000	0.003	<0.26	0.000	0.021
2	1	0.07	0.005	0.005	<0.26	0.000	0.021
2	2	<0.04	0.000	0.003	<0.26	0.000	0.021
2	3	<0.04	0.000	0.003	<0.26	0.000	0.021
2	4	<0.04	0.000	0.003	<0.26	0.000	0.021
2	5	<0.04	0.000	0.003	<0.26	0.000	0.021
2	6	<0.04	0.000	0.003	<0.26	0.000	0.021
2	7	0.04	0.003	0.003	<0.26	0.000	0.021
2	8	0.04	0.003	0.003	<0.26	0.000	0.021
3	1	0.07	0.006	0.006	<0.26	0.000	0.021
3	2	0.05	0.004	0.004	<0.26	0.000	0.021
3	3	0.05	0.004	0.004	<0.26	0.000	0.021
3	4	0.04	0.003	0.003	<0.26	0.000	0.021
3	5	0.07	0.006	0.006	<0.26	0.000	0.021
3	6	0.08	0.007	0.007	<0.26	0.000	0.021
3	7	0.15	0.012	0.012	<0.26	0.000	0.021
3	8	0.08	0.006	0.006	<0.26	0.000	0.021
4	1	0.07	0.006	0.006	<0.26	0.000	0.021
4	2	<0.04	0.000	0.003	<0.26	0.000	0.021
4	3	0.04	0.003	0.003	<0.26	0.000	0.021
4	4	0.04	0.003	0.003	<0.26	0.000	0.021
4	5	0.06	0.004	0.004	<0.26	0.000	0.021
4	6	0.06	0.005	0.005	<0.26	0.000	0.021
4	7	0.10	0.008	0.008	<0.26	0.000	0.021
4	8	0.04	0.003	0.003	<0.26	0.000	0.021
5	1	0.09	0.007	0.007	<0.26	0.000	0.021
5	2	0.05	0.004	0.004	<0.26	0.000	0.021
5	3	0.05	0.004	0.004	<0.26	0.000	0.021
5	4	0.04	0.003	0.003	<0.26	0.000	0.021
5	5	0.06	0.005	0.005	<0.26	0.000	0.021
5	6	0.05	0.004	0.004	<0.26	0.000	0.021
5	7	0.09	0.007	0.007	<0.26	0.000	0.021
5	8	0.04	0.003	0.003	<0.26	0.000	0.021
			∑ min	∑ max		∑ min	∑ max
1			0.010	0.030		0.000	0.166
2			0.012	0.028		0.000	0.166
3			0.048	0.048		0.000	0.166
4			0.033	0.036		0.000	0.166
5			0.036	0.036		0.000	0.167
Mean			0.028	0.036		0.000	0.166
SD			0.016	0.008		0.000	0.000

Table G. Continued CUS.

Stone	Fraction	LAB 2 CUS2 Se µg/l	min r mg/m^2	max r mg/m^2	LAB 2 CUS3 Se µg/l	min r mg/m^2	max r mg/m^2
1	1	<0.96	0.000	0.077			
1	2	<0.96	0.000	0.077			
1	3	<0.96	0.000	0.077			
1	4	<0.96	0.000	0.077			
1	5	<0.96	0.000	0.077			
1	6	<0.96	0.000	0.077			
1	7	<0.96	0.000	0.077			
1	8	<0.96	0.000	0.077			
2	1	<0.96	0.000	0.077	<0.96	0.000	0.077
2	2	<0.96	0.000	0.077	<0.96	0.000	0.077
2	3	<0.96	0.000	0.077	<0.96	0.000	0.077
2	4	<0.96	0.000	0.077	<0.96	0.000	0.077
2	5	<0.96	0.000	0.077	<0.96	0.000	0.077
2	6	<0.96	0.000	0.077	<0.96	0.000	0.077
2	7	<0.96	0.000	0.077	<0.96	0.000	0.077
2	8	<0.96	0.000	0.077	<0.96	0.000	0.077
3	1	<0.96	0.000	0.077	<0.96	0.000	0.077
3	2	<0.96	0.000	0.077	<0.96	0.000	0.077
3	3	<0.96	0.000	0.077	<0.96	0.000	0.077
3	4	<0.96	0.000	0.077	<0.96	0.000	0.077
3	5	<0.96	0.000	0.077	<0.96	0.000	0.077
3	6	<0.96	0.000	0.077	<0.96	0.000	0.077
3	7	<0.96	0.000	0.077	<0.96	0.000	0.077
3	8	<0.96	0.000	0.077	<0.96	0.000	0.077
4	1	<0.96	0.000	0.077	<0.96	0.000	0.077
4	2	<0.96	0.000	0.077	<0.96	0.000	0.077
4	3	<0.96	0.000	0.077	<0.96	0.000	0.077
4	4	<0.96	0.000	0.077	<0.96	0.000	0.077
4	5	<0.96	0.000	0.077	<0.96	0.000	0.077
4	6	<0.96	0.000	0.077	<0.96	0.000	0.077
4	7	<0.96	0.000	0.077	<0.96	0.000	0.077
4	8	<0.96	0.000	0.077	<0.96	0.000	0.077
5	1	<0.96	0.000	0.077	<0.96	0.000	0.077
5	2	<0.96	0.000	0.077	<0.96	0.000	0.077
5	3	<0.96	0.000	0.077	<0.96	0.000	0.077
5	4	<0.96	0.000	0.077	<0.96	0.000	0.077
5	5	<0.96	0.000	0.077	<0.96	0.000	0.077
5	6	<0.96	0.000	0.077	<0.96	0.000	0.077
5	7	<0.96	0.000	0.077	<0.96	0.000	0.077
5	8	<0.96	0.000	0.077	<0.96	0.000	0.077
			∑ min	∑ max		∑ min	∑ max
1			0.000	0.614			
2			0.000	0.614		0.000	0.615
3			0.000	0.614		0.000	0.615
4			0.000	0.615		0.000	0.614
5			0.000	0.614		0.000	0.614
Mean			0.000	0.614		0.000	0.615
SD			0.000	0.000		0.000	0.001

Table G. Continued CUS.

Stone	Fraction	LAB 1 CUS1 Sn µg/l	min r mg/m^2	max r mg/m^2	LAB 2 CUS1 Sn µg/l	min r mg/m^2	max r mg/m^2
1	1	0.10	0.008	0.008	<0.38	0.000	0.030
1	2	0.06	0.005	0.005	<0.38	0.000	0.030
1	3	0.06	0.005	0.005	<0.38	0.000	0.030
1	4	0.04	0.003	0.003	<0.38	0.000	0.030
1	5	0.05	0.004	0.004	<0.38	0.000	0.030
1	6	0.06	0.005	0.005	<0.38	0.000	0.030
1	7	0.15	0.012	0.012	<0.38	0.000	0.030
1	8	0.10	0.008	0.008	<0.38	0.000	0.030
2	1	0.10	0.008	0.008	<0.38	0.000	0.030
2	2	0.06	0.005	0.005	<0.38	0.000	0.030
2	3	0.07	0.006	0.006	<0.38	0.000	0.030
2	4	0.06	0.005	0.005	<0.38	0.000	0.030
2	5	0.05	0.004	0.004	<0.38	0.000	0.030
2	6	0.06	0.005	0.005	<0.38	0.000	0.030
2	7	0.18	0.015	0.015	<0.38	0.000	0.030
2	8	0.11	0.009	0.009	<0.38	0.000	0.030
3	1	0.31	0.025	0.025	<0.38	0.000	0.030
3	2	0.10	0.008	0.008	<0.38	0.000	0.030
3	3	0.06	0.005	0.005	<0.38	0.000	0.030
3	4	0.04	0.004	0.004	<0.38	0.000	0.030
3	5	0.06	0.004	0.004	<0.38	0.000	0.030
3	6	0.08	0.006	0.006	<0.38	0.000	0.030
3	7	0.11	0.009	0.009	<0.38	0.000	0.030
3	8	0.08	0.006	0.006	<0.38	0.000	0.030
4	1	0.31	0.025	0.025	<0.38	0.000	0.030
4	2	0.07	0.006	0.006	<0.38	0.000	0.030
4	3	0.06	0.005	0.005	<0.38	0.000	0.030
4	4	0.06	0.005	0.005	<0.38	0.000	0.030
4	5	0.05	0.004	0.004	<0.38	0.000	0.030
4	6	0.07	0.005	0.005	<0.38	0.000	0.030
4	7	0.12	0.009	0.009	<0.38	0.000	0.030
4	8	0.10	0.008	0.008	<0.38	0.000	0.030
5	1	0.19	0.015	0.015	<0.38	0.000	0.030
5	2	0.10	0.008	0.008	<0.38	0.000	0.030
5	3	0.12	0.010	0.010	<0.38	0.000	0.030
5	4	0.07	0.006	0.006	<0.38	0.000	0.030
5	5	0.08	0.006	0.006	<0.38	0.000	0.030
5	6	0.11	0.008	0.008	<0.38	0.000	0.030
5	7	0.22	0.018	0.018	<0.38	0.000	0.030
5	8	0.19	0.015	0.015	<0.38	0.000	0.030
			∑ min	∑ max			
1			0.050	0.050		0.000	0.243
2			0.056	0.056		0.000	0.243
3			0.067	0.067		0.000	0.243
4			0.067	0.067		0.000	0.243
5			0.086	0.086		0.000	0.243
Mean			0.065	0.065		0.000	0.243
SD			0.014	0.014		0.000	0.000

Table G. Continued CUS.

Stone	Fraction	LAB 2 CUS2 Sn µg/l	min r mg/m^2	max r mg/m^2	LAB 2 CUS3 Sn µg/l	min r mg/m^2	max r mg/m^2
1	1	<0.17	0.000	0.014			
1	2	<0.17	0.000	0.014			
1	3	<0.17	0.000	0.014			
1	4	0.23	0.018	0.018			
1	5	<0.17	0.000	0.014			
1	6	<0.17	0.000	0.014			
1	7	<0.17	0.000	0.014			
1	8	<0.17	0.000	0.014			
2	1	<0.17	0.000	0.014	<0.08	0.000	0.006
2	2	<0.17	0.000	0.014	<0.08	0.000	0.006
2	3	0.20	0.016	0.016	<0.08	0.000	0.006
2	4	<0.17	0.000	0.014	<0.08	0.000	0.006
2	5	<0.17	0.000	0.014	<0.08	0.000	0.006
2	6	<0.17	0.000	0.014	<0.08	0.000	0.006
2	7	<0.17	0.000	0.014	<0.08	0.000	0.006
2	8	<0.17	0.000	0.014	<0.08	0.000	0.006
3	1	<0.17	0.000	0.014	<0.08	0.000	0.006
3	2	<0.17	0.000	0.014	<0.08	0.000	0.006
3	3	<0.17	0.000	0.014	<0.08	0.000	0.006
3	4	<0.17	0.000	0.014	<0.08	0.000	0.006
3	5	<0.17	0.000	0.014	<0.08	0.000	0.006
3	6	<0.17	0.000	0.014	<0.08	0.000	0.006
3	7	<0.17	0.000	0.014	<0.08	0.000	0.006
3	8	<0.17	0.000	0.014	<0.08	0.000	0.006
4	1	<0.17	0.000	0.014	<0.08	0.000	0.006
4	2	<0.17	0.000	0.014	<0.08	0.000	0.006
4	3	<0.17	0.000	0.014	<0.08	0.000	0.006
4	4	<0.17	0.000	0.014	<0.08	0.000	0.006
4	5	<0.17	0.000	0.014	<0.08	0.000	0.006
4	6	<0.17	0.000	0.014	<0.08	0.000	0.006
4	7	<0.17	0.000	0.014	<0.08	0.000	0.006
4	8	<0.17	0.000	0.014	<0.08	0.000	0.006
5	1	<0.17	0.000	0.014	<0.08	0.000	0.006
5	2	<0.17	0.000	0.014	<0.08	0.000	0.006
5	3	0.20	0.016	0.016	<0.08	0.000	0.006
5	4	<0.17	0.000	0.014	<0.08	0.000	0.006
5	5	<0.17	0.000	0.014	<0.08	0.000	0.006
5	6	<0.17	0.000	0.014	<0.08	0.000	0.006
5	7	<0.17	0.000	0.014	<0.08	0.000	0.006
5	8	<0.17	0.000	0.014	<0.08	0.000	0.006
			∑ min	∑ max		∑ min	∑ max
1			0.018	0.113			
2			0.016	0.112		0.000	0.051
3			0.000	0.109		0.000	0.051
4			0.000	0.109		0.000	0.051
5			0.016	0.111		0.000	0.051
Mean			0.010	0.111		0.000	0.051
SD			0.009	0.002		0.000	0.000

Table G. Continued CUS.

Stone	Fraction	LAB 1 CUS1 Sr µg/l	min r mg/m^2	max r mg/m^2	LAB 2 CUS1 Sr µg/l	min r mg/m^2	max r mg/m^2
1	1	5.07	0.41	0.41	2.73	0.22	0.22
1	2	8.38	0.67	0.67	1.95	0.16	0.16
1	3	6.21	0.50	0.50	1.44	0.11	0.11
1	4	2.20	0.18	0.18	1.12	0.09	0.09
1	5	6.95	0.56	0.56	1.50	0.12	0.12
1	6	7.23	0.58	0.58	1.34	0.11	0.11
1	7	7.01	0.56	0.56	1.94	0.16	0.16
1	8	7.09	0.57	0.57	2.12	0.17	0.17
2	1	8.90	0.71	0.71	2.78	0.22	0.22
2	2	8.23	0.66	0.66	2.99	0.24	0.24
2	3	5.34	0.43	0.43	2.40	0.19	0.19
2	4	7.01	0.56	0.56	1.48	0.12	0.12
2	5	4.69	0.38	0.38	1.72	0.14	0.14
2	6	7.20	0.58	0.58	1.22	0.10	0.10
2	7	9.98	0.80	0.80	1.84	0.15	0.15
2	8	6.87	0.55	0.55	1.67	0.13	0.13
3	1	8.26	0.66	0.66	2.17	0.17	0.17
3	2	7.89	0.63	0.63	1.78	0.14	0.14
3	3	7.86	0.63	0.63	1.23	0.10	0.10
3	4	4.12	0.33	0.33	0.72	0.06	0.06
3	5	3.86	0.31	0.31	0.85	0.07	0.07
3	6	8.23	0.66	0.66	0.67	0.05	0.05
3	7	8.62	0.69	0.69	0.96	0.08	0.08
3	8	7.03	0.56	0.56	1.31	0.10	0.10
4	1	7.72	0.62	0.62	2.78	0.22	0.22
4	2	8.02	0.64	0.64	2.75	0.22	0.22
4	3	5.57	0.45	0.45	2.64	0.21	0.21
4	4	6.62	0.53	0.53	1.74	0.14	0.14
4	5	2.55	0.20	0.20	1.58	0.13	0.13
4	6	7.23	0.58	0.58	1.16	0.09	0.09
4	7	7.81	0.62	0.62	1.75	0.14	0.14
4	8	7.75	0.62	0.62	2.39	0.19	0.19
5	1	8.40	0.67	0.67	2.46	0.20	0.20
5	2	3.35	0.27	0.27	1.82	0.15	0.15
5	3	6.81	0.54	0.54	1.23	0.10	0.10
5	4	3.95	0.32	0.32	0.80	0.06	0.06
5	5	7.59	0.61	0.61	0.91	0.07	0.07
5	6	7.84	0.63	0.63	0.73	0.06	0.06
5	7	8.06	0.64	0.64	1.21	0.10	0.10
5	8	7.25	0.58	0.58	1.56	0.12	0.12
			∑ min	∑ max		∑ min	∑ max
1			4.01	4.01		1.13	1.13
2			4.66	4.66		1.29	1.29
3			4.47	4.47		0.77	0.77
4			4.26	4.26		1.34	1.34
5			4.26	4.26		0.86	0.86
Mean			4.33	4.33		1.08	1.08
SD			0.24	0.24		0.25	0.25

Table G. Continued CUS.

Stone	Fraction	LAB 2 CUS2 Sr µg/l	min r mg/m^2	max r mg/m^2	LAB 2 CUS3 Sr µg/l	min r mg/m^2	max r mg/m^2
1	1	0.72	0.058	0.058			
1	2	<0.50	0.000	0.040			
1	3	<0.50	0.000	0.040			
1	4	<0.50	0.000	0.040			
1	5	<0.50	0.000	0.040			
1	6	<0.50	0.000	0.040			
1	7	<0.50	0.000	0.040			
1	8	<0.50	0.000	0.040			
2	1	<0.50	0.000	0.040	<0.50	0.000	0.040
2	2	<0.50	0.000	0.040	<0.50	0.000	0.040
2	3	<0.50	0.000	0.040	<0.50	0.000	0.040
2	4	<0.50	0.000	0.040	<0.50	0.000	0.040
2	5	<0.50	0.000	0.040	<0.50	0.000	0.040
2	6	<0.50	0.000	0.040	<0.50	0.000	0.040
2	7	<0.50	0.000	0.040	<0.50	0.040	0.040
2	8	0.74	0.059	0.059	<0.50	0.040	0.040
3	1	1.08	0.087	0.087	<0.50	0.000	0.040
3	2	0.55	0.044	0.044	<0.50	0.000	0.040
3	3	0.51	0.040	0.040	<0.50	0.000	0.040
3	4	<0.50	0.000	0.040	<0.50	0.000	0.040
3	5	0.61	0.049	0.049	1.07	0.085	0.085
3	6	<0.50	0.000	0.040	0.64	0.051	0.051
3	7	0.52	0.041	0.041	1.00	0.080	0.080
3	8	0.74	0.059	0.059	0.85	0.068	0.068
4	1	0.95	0.076	0.076	<0.50	0.000	0.040
4	2	<0.50	0.000	0.040	<0.50	0.000	0.040
4	3	<0.50	0.000	0.040	<0.50	0.000	0.040
4	4	<0.50	0.000	0.040	<0.50	0.000	0.040
4	5	<0.50	0.000	0.040	<0.50	0.040	0.040
4	6	<0.50	0.000	0.040	<0.50	0.040	0.040
4	7	<0.50	0.000	0.040	0.71	0.057	0.057
4	8	0.81	0.065	0.065	0.71	0.057	0.057
5	1	1.06	0.085	0.085	0.57	0.046	0.046
5	2	0.50	0.040	0.040	0.71	0.057	0.057
5	3	<0.50	0.000	0.040	<0.50	0.000	0.040
5	4	<0.50	0.000	0.040	<0.50	0.000	0.040
5	5	0.73	0.058	0.058	<0.50	0.000	0.040
5	6	<0.50	0.000	0.040	0.64	0.051	0.051
5	7	<0.50	0.000	0.040	0.78	0.063	0.063
5	8	0.74	0.059	0.059	0.64	0.051	0.051
			∑ min	∑ max		∑ min	∑ max
1			0.058	0.338			
2			0.059	0.339		0.080	0.320
3			0.320	0.400		0.285	0.445
4			0.141	0.381		0.194	0.354
5			0.241	0.401		0.267	0.387
Mean			0.164	0.372		0.206	0.377
SD			0.115	0.032		0.093	0.053

Table G. Continued CUS.

Stone	Fraction	LAB 1 CUS1 U µg/l	min r mg/m^2	max r mg/m^2	LAB 2 CUS1 U µg/l	min r mg/m2	max r mg/m2
1	1	<0.04	0.0	0.003	<0.01	0.000	0.001
1	2	<0.04	0.0	0.003	<0.01	0.000	0.001
1	3	<0.04	0.0	0.003	<0.01	0.000	0.001
1	4	<0.04	0.0	0.003	<0.01	0.000	0.001
1	5	<0.04	0.0	0.003	<0.01	0.000	0.001
1	6	<0.04	0.0	0.003	<0.01	0.000	0.001
1	7	<0.04	0.0	0.003	<0.01	0.000	0.001
1	8	<0.04	0.0	0.003	<0.01	0.000	0.001
2	1	<0.04	0.0	0.003	<0.01	0.000	0.001
2	2	<0.04	0.0	0.003	<0.01	0.000	0.001
2	3	<0.04	0.0	0.003	<0.01	0.000	0.001
2	4	<0.04	0.0	0.003	<0.01	0.000	0.001
2	5	<0.04	0.0	0.003	<0.01	0.000	0.001
2	6	<0.04	0.0	0.003	<0.01	0.000	0.001
2	7	<0.04	0.0	0.003	<0.01	0.000	0.001
2	8	<0.04	0.0	0.003	<0.01	0.000	0.001
3	1	<0.04	0.0	0.003	<0.01	0.000	0.001
3	2	<0.04	0.0	0.003	<0.01	0.000	0.001
3	3	<0.04	0.0	0.003	<0.01	0.000	0.001
3	4	<0.04	0.0	0.003	<0.01	0.000	0.001
3	5	<0.04	0.0	0.003	<0.01	0.000	0.001
3	6	<0.04	0.0	0.003	<0.01	0.000	0.001
3	7	<0.04	0.0	0.003	<0.01	0.000	0.001
3	8	<0.04	0.0	0.003	<0.01	0.000	0.001
4	1	<0.04	0.0	0.003	<0.01	0.000	0.001
4	2	<0.04	0.0	0.003	<0.01	0.000	0.001
4	3	<0.04	0.0	0.003	<0.01	0.000	0.001
4	4	<0.04	0.0	0.003	<0.01	0.000	0.001
4	5	<0.04	0.0	0.003	<0.01	0.000	0.001
4	6	<0.04	0.0	0.003	<0.01	0.000	0.001
4	7	<0.04	0.0	0.003	<0.01	0.000	0.001
4	8	<0.04	0.0	0.003	<0.01	0.000	0.001
5	1	<0.04	0.0	0.003	<0.01	0.000	0.001
5	2	<0.04	0.0	0.003	<0.01	0.000	0.001
5	3	<0.04	0.0	0.003	<0.01	0.000	0.001
5	4	<0.04	0.0	0.003	<0.01	0.000	0.001
5	5	<0.04	0.0	0.003	<0.01	0.000	0.001
5	6	<0.04	0.0	0.003	<0.01	0.000	0.001
5	7	<0.04	0.0	0.003	<0.01	0.000	0.001
5	8	<0.04	0.0	0.003	<0.01	0.000	0.001
			∑ min	∑ max		∑ min	∑ max
1			0.000	0.026		0.000	0.006
2			0.000	0.026		0.000	0.006
3			0.000	0.026		0.000	0.006
4			0.000	0.026		0.000	0.006
5			0.000	0.026		0.000	0.006
Mean			0.000	0.026		0.000	0.006
SD			0.000	0.000		0.000	0.000

Table G. Continued CUS.

Stone	Fraction	LAB 2 CUS2 U µg/l	min r mg/m^2	max r mg/m^2	LAB 2 CUS3 U µg/l	min r mg/m^2	max r mg/m^2
1	1	<0.08	0.000	0.006			
1	2	<0.08	0.000	0.006			
1	3	<0.08	0.000	0.006			
1	4	<0.08	0.000	0.006			
1	5	<0.08	0.000	0.006			
1	6	<0.08	0.000	0.006			
1	7	<0.08	0.000	0.006			
1	8	<0.08	0.000	0.006			
2	1	<0.08	0.000	0.006	<0.02	0.000	0.002
2	2	<0.08	0.000	0.006	<0.02	0.000	0.002
2	3	<0.08	0.000	0.006	<0.02	0.000	0.002
2	4	<0.08	0.000	0.006	<0.02	0.000	0.002
2	5	<0.08	0.000	0.006	<0.02	0.000	0.002
2	6	<0.08	0.000	0.006	<0.02	0.000	0.002
2	7	<0.08	0.000	0.006	<0.02	0.000	0.002
2	8	<0.08	0.000	0.006	<0.02	0.000	0.002
3	1	<0.08	0.000	0.006	<0.02	0.000	0.002
3	2	<0.08	0.000	0.006	<0.02	0.000	0.002
3	3	<0.08	0.000	0.006	<0.02	0.000	0.002
3	4	<0.08	0.000	0.006	<0.02	0.000	0.002
3	5	<0.08	0.000	0.006	<0.02	0.000	0.002
3	6	<0.08	0.000	0.006	<0.02	0.000	0.002
3	7	<0.08	0.000	0.006	<0.02	0.000	0.002
3	8	<0.08	0.000	0.006	<0.02	0.000	0.002
4	1	<0.08	0.000	0.006	<0.02	0.000	0.002
4	2	<0.08	0.000	0.006	<0.02	0.000	0.002
4	3	<0.08	0.000	0.006	<0.02	0.000	0.002
4	4	<0.08	0.000	0.006	<0.02	0.000	0.002
4	5	<0.08	0.000	0.006	<0.02	0.000	0.002
4	6	<0.08	0.000	0.006	<0.02	0.000	0.002
4	7	<0.08	0.000	0.006	<0.02	0.000	0.002
4	8	<0.08	0.000	0.006	<0.02	0.000	0.002
5	1	<0.08	0.000	0.006	<0.02	0.000	0.002
5	2	<0.08	0.000	0.006	<0.02	0.000	0.002
5	3	<0.08	0.000	0.006	<0.02	0.000	0.002
5	4	<0.08	0.000	0.006	<0.02	0.000	0.002
5	5	<0.08	0.000	0.006	<0.02	0.000	0.002
5	6	<0.08	0.000	0.006	<0.02	0.000	0.002
5	7	<0.08	0.000	0.006	<0.02	0.000	0.002
5	8	<0.08	0.000	0.006	<0.02	0.000	0.002
			∑ min	∑ max		∑ min	∑ max
1			0.000	0.051			
2			0.000	0.051		0.000	0.013
3			0.000	0.051		0.000	0.013
4			0.000	0.051		0.000	0.013
5			0.000	0.051		0.000	0.013
Mean			0.000	0.051		0.000	0.013
SD			0.000	0.000		0.000	0.000

Table G. Continued CUS.

Stone	Fraction	LAB 1 CUS1 V µg/l	min r mg/m²	max r mg/m²	LAB 2 CUS1 V µg/l	min r mg/m²	max r mg/m²
1	1	0.03	0.003	0.003	<0.02	0.000	0.002
1	2	0.02	0.002	0.002	<0.02	0.000	0.002
1	3	0.02	0.002	0.002	<0.02	0.000	0.002
1	4	<0.02	0.000	0.002	<0.02	0.000	0.002
1	5	0.02	0.002	0.002	<0.02	0.000	0.002
1	6	0.05	0.004	0.004	<0.02	0.000	0.002
1	7	0.04	0.003	0.003	<0.02	0.000	0.002
1	8	<0.02	0.000	0.002	<0.02	0.000	0.002
2	1	0.04	0.003	0.003	<0.02	0.000	0.002
2	2	0.03	0.002	0.002	<0.02	0.000	0.002
2	3	<0.02	0.000	0.002	<0.02	0.000	0.002
2	4	<0.02	0.000	0.002	<0.02	0.000	0.002
2	5	<0.02	0.000	0.002	<0.02	0.002	0.002
2	6	<0.02	0.000	0.002	<0.02	0.000	0.002
2	7	0.04	0.004	0.004	<0.02	0.000	0.002
2	8	<0.02	0.000	0.002	<0.02	0.000	0.002
3	1	0.03	0.002	0.002	<0.02	0.000	0.002
3	2	<0.02	0.000	0.002	<0.02	0.000	0.002
3	3	<0.02	0.000	0.002	<0.02	0.000	0.002
3	4	<0.02	0.000	0.002	<0.02	0.000	0.002
3	5	<0.02	0.000	0.002	<0.02	0.000	0.002
3	6	0.02	0.002	0.002	<0.02	0.000	0.002
3	7	0.04	0.003	0.003	<0.02	0.000	0.002
3	8	<0.02	0.000	0.002	<0.02	0.000	0.002
4	1	0.04	0.003	0.003	<0.02	0.000	0.002
4	2	0.02	0.002	0.002	<0.02	0.000	0.002
4	3	0.03	0.002	0.002	<0.02	0.000	0.002
4	4	<0.02	0.000	0.002	<0.02	0.000	0.002
4	5	0.02	0.002	0.002	<0.02	0.000	0.002
4	6	0.02	0.002	0.002	<0.02	0.000	0.002
4	7	0.04	0.003	0.003	<0.02	0.000	0.002
4	8	<0.02	0.000	0.002	<0.02	0.000	0.002
5	1	0.07	0.006	0.006	<0.02	0.002	0.002
5	2	0.03	0.002	0.002	<0.02	0.000	0.002
5	3	0.03	0.002	0.002	<0.02	0.000	0.002
5	4	0.02	0.002	0.002	<0.02	0.000	0.002
5	5	0.03	0.002	0.002	<0.02	0.000	0.002
5	6	0.03	0.002	0.002	<0.02	0.000	0.002
5	7	0.05	0.004	0.004	<0.02	0.000	0.002
5	8	0.02	0.002	0.002	<0.02	0.000	0.002
			∑ min	∑ max		∑ min	∑ max
1			0.016	0.019		0.000	0.013
2			0.009	0.017		0.002	0.013
3			0.007	0.015		0.000	0.013
4			0.015	0.018		0.000	0.013
5			0.021	0.021		0.002	0.013
Mean			0.014	0.018		0.001	0.013
SD			0.006	0.002		0.001	0.000

Table G. Continued CUS.

Stone	Fraction	LAB 2 CUS2 V µg/l	min r mg/m^2	max r mg/m^2	LAB 2 CUS3 V µg/l	min r mg/m^2	max r mg/m^2
1	1	<0.20	0.000	0.016			
1	2	<0.20	0.000	0.016			
1	3	<0.20	0.000	0.016			
1	4	<0.20	0.000	0.016			
1	5	<0.20	0.000	0.016			
1	6	<0.20	0.000	0.016			
1	7	<0.20	0.000	0.016			
1	8	<0.20	0.000	0.016			
2	1	<0.20	0.000	0.016	<0.06	0.000	0.005
2	2	<0.20	0.000	0.016	<0.06	0.000	0.005
2	3	<0.20	0.000	0.016	<0.06	0.000	0.005
2	4	<0.20	0.000	0.016	<0.06	0.000	0.005
2	5	<0.20	0.000	0.016	<0.06	0.000	0.005
2	6	<0.20	0.000	0.016	<0.06	0.000	0.005
2	7	<0.20	0.000	0.016	<0.06	0.000	0.005
2	8	<0.20	0.000	0.016	<0.06	0.000	0.005
3	1	<0.20	0.000	0.016	<0.06	0.000	0.005
3	2	<0.20	0.000	0.016	<0.06	0.000	0.005
3	3	<0.20	0.000	0.016	<0.06	0.000	0.005
3	4	<0.20	0.000	0.016	<0.06	0.000	0.005
3	5	<0.20	0.000	0.016	0.07	0.006	0.006
3	6	<0.20	0.000	0.016	0.07	0.005	0.005
3	7	<0.20	0.000	0.016	<0.06	0.000	0.005
3	8	<0.20	0.000	0.016	<0.06	0.000	0.005
4	1	<0.20	0.000	0.016	<0.06	0.000	0.005
4	2	<0.20	0.000	0.016	<0.06	0.000	0.005
4	3	<0.20	0.000	0.016	<0.06	0.000	0.005
4	4	<0.20	0.000	0.016	<0.06	0.000	0.005
4	5	<0.20	0.000	0.016	<0.06	0.000	0.005
4	6	<0.20	0.000	0.016	<0.06	0.000	0.005
4	7	<0.20	0.000	0.016	<0.06	0.000	0.005
4	8	<0.20	0.000	0.016	<0.06	0.000	0.005
5	1	<0.20	0.000	0.016	<0.06	0.000	0.005
5	2	<0.20	0.000	0.016	<0.06	0.000	0.005
5	3	<0.20	0.000	0.016	<0.06	0.000	0.005
5	4	<0.20	0.000	0.016	<0.06	0.000	0.005
5	5	<0.20	0.000	0.016	<0.06	0.000	0.005
5	6	<0.20	0.000	0.016	<0.06	0.000	0.005
5	7	<0.20	0.000	0.016	<0.06	0.000	0.005
5	8	<0.20	0.000	0.016	<0.06	0.000	0.005
			∑ min	∑ max		∑ min	∑ max
1			0.000	0.128			
2			0.000	0.128		0.000	0.038
3			0.000	0.128		0.011	0.040
4			0.000	0.128		0.000	0.038
5			0.000	0.128		0.000	0.038
Mean			0.000	0.128		0.003	0.039
SD			0.000	0.000		0.005	0.001

Table G. Continued CUS.

Stone	Fraction	LAB 1 CUS1 Zn µg/l	min r mg/m²	max r mg/m²	LAB 2 CUS1 Zn µg/l	min r mg/m²	max r mg/m²
1	1	283	22.61	22.61	27.4	2.19	2.19
1	2	469	37.53	37.53	26.1	2.09	2.09
1	3	395	31.57	31.57	60.6	4.84	4.84
1	4	153	12.21	12.21	70.9	5.66	5.66
1	5	439	35.12	35.12	103	8.21	8.21
1	6	303	24.26	24.26	71.7	5.73	5.73
1	7	338	27.06	27.06	85.5	6.83	6.83
1	8	281	22.45	22.45	73.1	5.84	5.84
2	1	493	39.41	39.41	14.1	1.13	1.13
2	2	300	23.97	23.97	11.1	0.89	0.89
2	3	256	20.51	20.51	35.5	2.84	2.84
2	4	256	20.44	20.44	59.2	4.74	4.74
2	5	251	20.10	20.10	134	10.7	10.7
2	6	327	26.12	26.12	102	8.16	8.16
2	7	595	47.57	47.57	118	9.46	9.46
2	8	285	22.82	22.82	85.3	6.82	6.82
3	1	427	34.14	34.14	10.1	0.81	0.81
3	2	412	32.93	32.93	11.4	0.91	0.91
3	3	453	36.27	36.27	47.6	3.81	3.81
3	4	345	27.61	27.61	43.9	3.51	3.51
3	5	286	22.84	22.84	60.1	4.81	4.81
3	6	392	31.33	31.33	37.8	3.02	3.02
3	7	537	42.95	42.95	50.8	4.06	4.06
3	8	368	29.40	29.40	53.2	4.26	4.26
4	1	387	30.93	30.93	38.3	3.06	3.06
4	2	454	36.33	36.33	21.4	1.71	1.71
4	3	229	18.31	18.31	58.3	4.66	4.66
4	4	424	33.91	33.91	132.0	10.6	10.6
4	5	182	14.58	14.58	298	23.8	23.8
4	6	325	26.00	26.00	269	21.5	21.5
4	7	415	33.18	33.18	337	26.9	26.9
4	8	331	26.44	26.44	340	27.1	27.1
5	1	286	22.87	22.87	18.7	1.50	1.50
5	2	231	18.48	18.48	7.3	0.58	0.58
5	3	266	21.25	21.25	26.7	2.14	2.14
5	4	219	17.48	17.48	43.9	3.52	3.52
5	5	448	35.82	35.82	71.5	5.73	5.73
5	6	452	36.14	36.14	40.7	3.26	3.26
5	7	396	31.65	31.65	59.4	4.76	4.76
5	8	299	23.91	23.91	58.8	4.71	4.71
			∑ min	∑ max		∑ min	∑ max
1			213	213		41.4	41.4
2			221	221		44.7	44.7
3			257	257		25.2	25.2
4			220	220		119	119
5			208	208		26.2	26.2
Mean			224	224		51.4	51.4
SD			20	20		39.0	39.0

Table G. Continued CUS.

Stone	Fraction	LAB 2 CUS2 Zn µg/l	min r mg/m^2	max r mg/m^2	LAB 2 CUS3 Zn µg/l	min r mg/m^2	max r mg/m^2
1	1	61.6	4.93	4.93			
1	2	48.1	3.85	3.85			
1	3	29.9	2.39	2.39			
1	4	22.4	1.79	1.79			
1	5	33.0	2.64	2.64			
1	6	26.9	2.15	2.15			
1	7	44.1	3.53	3.53			
1	8	42.7	3.42	3.42			
2	1	52.6	4.20	4.20	14.39	1.15	1.15
2	2	34.7	2.78	2.78	17.62	1.41	1.41
2	3	26.8	2.15	2.15	12.22	0.98	0.98
2	4	28.1	2.25	2.25	12.52	1.00	1.00
2	5	53.8	4.31	4.31	19.99	1.60	1.60
2	6	39.7	3.17	3.17	20.34	1.63	1.63
2	7	146	11.69	11.69	24.20	1.94	1.94
2	8	179	14.32	14.32	29.40	2.36	2.36
3	1	19.1	1.53	1.53	15.38	1.23	1.23
3	2	23.6	1.89	1.89	16.92	1.35	1.35
3	3	32.0	2.56	2.56	18.39	1.47	1.47
3	4	35.2	2.81	2.81	23.96	1.92	1.92
3	5	35.5	2.84	2.84	42.34	3.39	3.39
3	6	33.8	2.70	2.70	34.92	2.80	2.80
3	7	47.4	3.79	3.79	40.53	3.24	3.24
3	8	40.2	3.22	3.22	42.71	3.42	3.42
4	1	58.0	4.64	4.64	10.31	0.82	0.82
4	2	33.5	2.68	2.68	11.85	0.95	0.95
4	3	22.8	1.82	1.82	9.62	0.77	0.77
4	4	18.7	1.50	1.50	16.01	1.28	1.28
4	5	22.3	1.79	1.79	28.74	2.30	2.30
4	6	18.0	1.44	1.44	15.94	1.27	1.27
4	7	30.3	2.43	2.43	22.46	1.80	1.80
4	8	31.0	2.48	2.48	22.41	1.79	1.79
5	1	97.8	7.82	7.82	12.47	1.00	1.00
5	2	60.1	4.81	4.81	12.63	1.01	1.01
5	3	49.7	3.98	3.98	12.21	0.98	0.98
5	4	43.8	3.51	3.51	12.71	1.02	1.02
5	5	78.1	6.25	6.25	21.35	1.71	1.71
5	6	58.4	4.67	4.67	23.62	1.89	1.89
5	7	92.1	7.37	7.37	20.40	1.63	1.63
5	8	94.3	7.54	7.54	20.16	1.61	1.61
			∑ min	∑ max		∑ min	∑ max
1			24.68	24.68			
2			44.86	44.86		12.07	12.07
3			21.33	21.33		18.83	18.83
4			18.79	18.79		10.98	10.98
5			45.94	45.94		10.84	10.84
Mean			31.12	31.12		13.18	13.18
SD			13.21	13.21		3.80	3.80

Table H. DSLT results LDS (LAB 1 DSLT contract laboratory, LAB 2 DSLT BfG)

Stone	Fraction	LAB 1 Al µg/l	min r mg/m²	max r mg/m²	LAB 2 Al µg/l	min r mg/m²	max r mg/m²
1	1	57	5	5	46	3.7	3.7
1	2	42	3	3	43	3.4	3.4
1	3	60	5	5	42	3.4	3.4
1	4	73	6	6	49	3.9	3.9
1	5	103	8	8	49	3.9	3.9
1	6	73	6	6	80	6.4	6.4
1	7	1363	109	109	93	7.4	7.4
1	8	1414	113	113	161	12.9	12.9
2	1	<40.8	0	3	50	4.0	4.0
2	2	<40.8	0	3	109	8.7	8.7
2	3	<40.8	0	3	161	12.9	12.9
2	4	<40.8	0	3	210	16.8	16.8
2	5	<40.8	0	3	371	29.7	29.7
2	6	78	6	6	611	48.9	48.9
2	7	<40.8	0	3	717	57.3	57.3
2	8	<40.8	0	3	628	50.2	50.2
3	1	921	74	74	47	3.7	3.7
3	2	1916	153	153	83	6.6	6.6
3	3	2893	231	231	116	9.3	9.3
3	4	3039	243	243	203	16.2	16.2
3	5	2775	222	222	374	29.9	29.9
3	6	568	45	45	508	40.6	40.6
3	7	3384	271	271	573	45.9	45.9
3	8	1948	156	156	622	49.8	49.8
4	1	83	7	7	43	3.5	3.5
4	2	185	15	15	57	4.6	4.6
4	3	233	19	19	74	5.9	5.9
4	4	187	15	15	108	8.6	8.6
4	5	313	25	25	191	15.3	15.3
4	6	226	18	18	268	21.5	21.5
4	7	364	29	29	369	29.6	29.6
4	8	292	23	23	442	35.4	35.4
5	1	<40.8	0	3	44	3.6	3.6
5	2	75	6	6	94	7.5	7.5
5	3	102	8	8	131	10.5	10.5
5	4	104	8	8	201	16.1	16.1
5	5	137	11	11	343	27.4	27.4
5	6	87	7	7	477	38.1	38.1
5	7	113	9	9	564	45.1	45.1
5	8	62	5	5	585	46.8	46.8
			∑ Min	∑ Max		∑ Min	∑ Max
1			255	255		45.0	45.0
2			6	29		228.5	228.5
3			1396	1396		202.1	202.1
4			151	151		124.3	124.3
5			55	58		195.0	195.0
Mean			372	378		159.0	159.0
SD			580	576		74.5	74.5

Table H. Continued LDS.

Stone	Fraction	LAB 1 As µg/l	min r mg/m^2	max r mg/m^2	LAB 2 As µg/l	min r mg/m^2	max r mg/m^2
1	1	0.05	0.004	0.004	<0.04	0.000	0.003
1	2	0.04	0.003	0.003	<0.04	0.000	0.003
1	3	0.03	0.003	0.003	<0.04	0.000	0.003
1	4	0.03	0.003	0.003	<0.04	0.000	0.003
1	5	0.04	0.003	0.003	<0.04	0.000	0.003
1	6	0.04	0.003	0.003	<0.04	0.000	0.003
1	7	<0.03	0.000	0.002	<0.04	0.000	0.003
1	8	<0.03	0.000	0.002	<0.04	0.000	0.003
2	1	0.04	0.003	0.003	<0.04	0.000	0.003
2	2	0.04	0.003	0.003	<0.04	0.000	0.003
2	3	0.04	0.003	0.003	<0.04	0.000	0.003
2	4	0.03	0.003	0.003	<0.04	0.000	0.003
2	5	0.04	0.003	0.003	<0.04	0.000	0.003
2	6	0.05	0.004	0.004	<0.04	0.000	0.003
2	7	0.03	0.003	0.003	<0.04	0.000	0.003
2	8	0.03	0.002	0.002	<0.04	0.000	0.003
3	1	0.05	0.004	0.004	<0.04	0.000	0.003
3	2	0.03	0.002	0.002	<0.04	0.000	0.003
3	3	0.04	0.003	0.003	<0.04	0.000	0.003
3	4	0.02	0.002	0.002	<0.04	0.000	0.003
3	5	0.04	0.003	0.003	<0.04	0.000	0.003
3	6	0.05	0.004	0.004	<0.04	0.000	0.003
3	7	0.03	0.003	0.003	<0.04	0.000	0.003
3	8	0.08	0.006	0.006	<0.04	0.000	0.003
4	1	0.03	0.002	0.002	<0.04	0.000	0.003
4	2	<0.03	0.000	0.002	<0.04	0.000	0.003
4	3	0.03	0.002	0.002	<0.04	0.000	0.003
4	4	<0.03	0.000	0.002	<0.04	0.000	0.003
4	5	<0.03	0.000	0.002	<0.04	0.000	0.003
4	6	<0.03	0.000	0.002	<0.04	0.000	0.003
4	7	<0.03	0.000	0.002	<0.04	0.000	0.003
4	8	<0.03	0.000	0.002	<0.04	0.000	0.003
5	1	0.03	0.003	0.003	<0.04	0.000	0.003
5	2	0.03	0.002	0.002	<0.04	0.000	0.003
5	3	0.03	0.002	0.002	<0.04	0.000	0.003
5	4	0.03	0.002	0.002	<0.04	0.000	0.003
5	5	<0.03	0.000	0.002	<0.04	0.000	0.003
5	6	<0.03	0.000	0.002	<0.04	0.000	0.003
5	7	<0.03	0.000	0.002	<0.04	0.000	0.003
5	8	<0.03	0.000	0.002	<0.04	0.000	0.003
			∑ Min	∑ Max		∑ Min	∑ Max
1			0.018	0.023		0.000	0.026
2			0.024	0.024		0.000	0.026
3			0.027	0.027		0.000	0.026
4			0.005	0.019		0.000	0.026
5			0.009	0.019		0.000	0.026
Mean			0.017	0.022		0.000	0.026
SD			0.010	0.004		0.000	0.000

Table H. Continued LDS.

Stone	Fraction	LAB 1 Ba µg/l	min r mg/m^2	max r mg/m^2	LAB 2 Ba µg/l	min r mg/m^2	max r mg/m^2
1	1	492	39.3	39.3	1.14	0.09	0.09
1	2	469	37.5	37.5	1.21	0.10	0.10
1	3	500	40.0	40.0	1.30	0.10	0.10
1	4	492	39.3	39.3	1.71	0.14	0.14
1	5	26	2.1	2.1	2.24	0.18	0.18
1	6	487	39.0	39.0	2.48	0.20	0.20
1	7	378	30.3	30.3	3.10	0.25	0.25
1	8	428	34.2	34.2	3.65	0.29	0.29
2	1	366	29.3	29.3	1.49	0.12	0.12
2	2	461	36.9	36.9	1.42	0.11	0.11
2	3	497	39.8	39.8	1.80	0.14	0.14
2	4	235	18.8	18.8	2.44	0.19	0.19
2	5	484	38.7	38.7	4.86	0.39	0.39
2	6	488	39.0	39.0	8.48	0.68	0.68
2	7	175	14.0	14.0	13.87	1.11	1.11
2	8	385	30.8	30.8	18.36	1.47	1.47
3	1	445	35.6	35.6	1.09	0.09	0.09
3	2	121	9.7	9.7	1.33	0.11	0.11
3	3	143	11.5	11.5	1.67	0.13	0.13
3	4	203	16.2	16.2	2.41	0.19	0.19
3	5	499	39.9	39.9	4.73	0.38	0.38
3	6	505	40.4	40.4	6.90	0.55	0.55
3	7	429	34.4	34.4	10.30	0.82	0.82
3	8	571	45.6	45.6	12.67	1.01	1.01
4	1	506	40.5	40.5	1.19	0.10	0.10
4	2	498	39.9	39.9	<0.81	0.00	0.06
4	3	345	27.6	27.6	1.26	0.10	0.10
4	4	473	37.8	37.8	1.39	0.11	0.11
4	5	469	37.5	37.5	2.13	0.17	0.17
4	6	391	31.3	31.3	2.66	0.21	0.21
4	7	420	33.6	33.6	3.59	0.29	0.29
4	8	371	29.6	29.6	4.13	0.33	0.33
5	1	281	22.4	22.4	1.29	0.10	0.10
5	2	440	35.2	35.2	1.16	0.09	0.09
5	3	508	40.7	40.7	1.78	0.14	0.14
5	4	529	42.3	42.3	2.72	0.22	0.22
5	5	22	1.8	1.8	4.65	0.37	0.37
5	6	431	34.5	34.5	6.95	0.56	0.56
5	7	368	29.4	29.4	12.30	0.98	0.98
5	8	342	27.4	27.4	17.60	1.41	1.41
			∑ Min	∑ Max		∑ Min	∑ Max
1			261.6	261.6		1.35	1.35
2			247.3	247.3		4.22	4.22
3			233.3	233.3		3.29	3.29
4			277.7	277.7		1.31	1.37
5			233.6	233.6		3.88	3.88
Mean			250.7	250.7		2.81	2.82
SD			19.1	19.1		1.39	1.37

Table H. Continued LDS.

Stone	Fraction	LAB 1 Cd µg/l	min r mg/m2	max r mg/m2	LAB 2 Cd µg/l	min r mg/m2	max r mg/m2
1	1	0.0218	0.002	0.002	<0.01	0.000	0.001
1	2	0.0286	0.002	0.002	<0.01	0.000	0.001
1	3	0.0314	0.003	0.003	<0.01	0.000	0.001
1	4	0.0274	0.002	0.002	<0.01	0.000	0.001
1	5	<0.01	0.000	0.001	<0.01	0.000	0.001
1	6	0.0289	0.002	0.002	<0.01	0.000	0.001
1	7	0.0122	0.001	0.001	<0.01	0.000	0.001
1	8	0.0083	0.001	0.001	<0.01	0.000	0.001
2	1	0.0211	0.002	0.002	<0.01	0.000	0.001
2	2	0.0293	0.002	0.002	<0.01	0.000	0.001
2	3	0.0261	0.002	0.002	<0.01	0.000	0.001
2	4	0.0175	0.001	0.001	<0.01	0.000	0.001
2	5	0.0231	0.002	0.002	<0.01	0.000	0.001
2	6	0.0265	0.002	0.002	<0.01	0.000	0.001
2	7	0.0131	0.001	0.001	<0.01	0.000	0.001
2	8	0.0225	0.002	0.002	<0.01	0.000	0.001
3	1	0.0353	0.003	0.003	<0.01	0.000	0.001
3	2	0.0127	0.001	0.001	<0.01	0.000	0.001
3	3	0.0120	0.001	0.001	<0.01	0.000	0.001
3	4	0.0169	0.001	0.001	<0.01	0.000	0.001
3	5	0.0331	0.003	0.003	<0.01	0.000	0.001
3	6	0.0388	0.003	0.003	<0.01	0.000	0.001
3	7	0.0289	0.002	0.002	<0.01	0.000	0.001
3	8	0.0293	0.002	0.002	<0.01	0.000	0.001
4	1	0.0243	0.002	0.002	<0.01	0.000	0.001
4	2	0.0127	0.001	0.001	<0.01	0.000	0.001
4	3	0.0155	0.001	0.001	<0.01	0.000	0.001
4	4	0.0169	0.001	0.001	<0.01	0.000	0.001
4	5	0.0199	0.002	0.002	<0.01	0.000	0.001
4	6	0.0143	0.001	0.001	<0.01	0.000	0.001
4	7	0.0130	0.001	0.001	<0.01	0.000	0.001
4	8	0.0075	0.001	0.001	<0.01	0.000	0.001
5	1	0.0120	0.001	0.001	<0.01	0.000	0.001
5	2	0.0247	0.002	0.002	<0.01	0.000	0.001
5	3	0.0203	0.002	0.002	<0.01	0.000	0.001
5	4	0.0297	0.002	0.002	<0.01	0.000	0.001
5	5	<0.01	0.000	0.001	<0.01	0.000	0.001
5	6	0.0168	0.001	0.001	<0.01	0.000	0.001
5	7	0.0153	0.001	0.001	<0.01	0.000	0.001
5	8	0.0142	0.001	0.001	<0.01	0.000	0.001
			∑ Min	∑ Max		∑ Min	∑ Max
1			0.013	0.013		0.000	0.006
2			0.014	0.014		0.000	0.006
3			0.017	0.017		0.000	0.006
4			0.010	0.010		0.000	0.006
5			0.011	0.011		0.000	0.006
Mean			0.013	0.013		0.000	0.006
SD			0.003	0.003		0.000	0.000

Table H. Continued LDS.

Stone	Fraction	LAB 1 Co µg/l	min r mg/m2	max r mg/m2	LAB 2 Co µg/l	min r mg/m^2	max r mg/m^2
1	1	0.08	0.006	0.006	<0.01	0.000	0.001
1	2	0.07	0.006	0.006	<0.01	0.000	0.001
1	3	0.08	0.007	0.007	<0.01	0.000	0.001
1	4	0.07	0.006	0.006	<0.01	0.000	0.001
1	5	<0.02	0.000	0.002	<0.01	0.000	0.001
1	6	0.07	0.005	0.005	<0.01	0.000	0.001
1	7	0.08	0.007	0.007	<0.01	0.000	0.001
1	8	0.05	0.004	0.004	<0.01	0.000	0.001
2	1	0.06	0.005	0.005	<0.01	0.000	0.001
2	2	0.07	0.005	0.005	<0.01	0.000	0.001
2	3	0.09	0.007	0.007	<0.01	0.000	0.001
2	4	0.05	0.004	0.004	<0.01	0.000	0.001
2	5	0.07	0.005	0.005	<0.01	0.000	0.001
2	6	0.12	0.009	0.009	<0.01	0.000	0.001
2	7	0.07	0.005	0.005	<0.01	0.000	0.001
2	8	0.11	0.009	0.009	<0.01	0.000	0.001
3	1	0.08	0.006	0.006	<0.01	0.000	0.001
3	2	0.04	0.004	0.004	<0.01	0.000	0.001
3	3	0.06	0.005	0.005	<0.01	0.000	0.001
3	4	0.05	0.004	0.004	<0.01	0.000	0.001
3	5	0.07	0.006	0.006	<0.01	0.000	0.001
3	6	0.08	0.006	0.006	<0.01	0.000	0.001
3	7	0.11	0.009	0.009	<0.01	0.000	0.001
3	8	0.11	0.008	0.008	<0.01	0.000	0.001
4	1	0.07	0.005	0.005	0.02	0.002	0.002
4	2	0.04	0.003	0.003	<0.01	0.000	0.001
4	3	0.07	0.005	0.005	<0.01	0.000	0.001
4	4	0.04	0.004	0.004	<0.01	0.000	0.001
4	5	0.04	0.004	0.004	<0.01	0.000	0.001
4	6	0.09	0.007	0.007	<0.01	0.000	0.001
4	7	0.09	0.007	0.007	<0.01	0.000	0.001
4	8	0.07	0.005	0.005	<0.01	0.000	0.001
5	1	0.04	0.003	0.003	<0.01	0.000	0.001
5	2	0.06	0.005	0.005	<0.01	0.000	0.001
5	3	0.11	0.008	0.008	<0.01	0.000	0.001
5	4	0.08	0.006	0.006	<0.01	0.000	0.001
5	5	0.01	0.001	0.001	<0.01	0.000	0.001
5	6	0.09	0.007	0.007	<0.01	0.000	0.001
5	7	0.09	0.007	0.007	<0.01	0.000	0.001
5	8	0.09	0.007	0.007	<0.01	0.000	0.001
			∑ Min	∑ Max		∑ Min	∑ Max
1			0.040	0.042		0.000	0.006
2			0.050	0.050		0.000	0.006
3			0.047	0.047		0.000	0.006
4			0.040	0.040		0.002	0.007
5			0.044	0.044		0.000	0.006
Mean			0.045	0.045		0.000	0.007
SD			0.004	0.004		0.001	0.000

Table H. Continued LDS.

Stone	Fraction	LAB 1 Cr µg/l	min r mg/m^2	max r mg/m^2	LAB 2 Cr µg/l	min r mg/m^2	max r mg/m^2
1	1	0.17	0.013	0.013	0.67	0.054	0.054
1	2	0.15	0.012	0.012	0.74	0.060	0.060
1	3	0.21	0.017	0.017	0.52	0.042	0.042
1	4	0.18	0.014	0.014	0.59	0.047	0.047
1	5	0.34	0.027	0.027	1.09	0.087	0.087
1	6	0.52	0.042	0.042	1.29	0.103	0.103
1	7	0.63	0.050	0.050	2.14	0.171	0.171
1	8	0.70	0.056	0.056	2.01	0.161	0.161
2	1	0.35	0.028	0.028	0.70	0.056	0.056
2	2	0.37	0.030	0.030	0.69	0.055	0.055
2	3	0.34	0.027	0.027	0.37	0.029	0.029
2	4	0.39	0.031	0.031	0.33	0.026	0.026
2	5	0.80	0.064	0.064	0.50	0.040	0.040
2	6	1.06	0.085	0.085	0.56	0.045	0.045
2	7	2.44	0.195	0.195	0.91	0.073	0.073
2	8	2.14	0.172	0.172	0.99	0.079	0.079
3	1	0.25	0.020	0.020	0.32	0.026	0.026
3	2	0.14	0.011	0.011	0.37	0.030	0.030
3	3	0.16	0.013	0.013	0.39	0.031	0.031
3	4	0.14	0.011	0.011	0.59	0.047	0.047
3	5	0.23	0.018	0.018	1.42	0.114	0.114
3	6	0.27	0.021	0.021	1.59	0.128	0.128
3	7	0.42	0.033	0.033	2.77	0.222	0.222
3	8	0.54	0.044	0.044	2.60	0.208	0.208
4	1	0.59	0.047	0.047	0.70	0.056	0.056
4	2	0.43	0.034	0.034	0.41	0.033	0.033
4	3	0.39	0.032	0.032	0.34	0.027	0.027
4	4	0.32	0.025	0.025	0.41	0.033	0.033
4	5	0.54	0.043	0.043	0.86	0.069	0.069
4	6	0.59	0.047	0.047	1.10	0.088	0.088
4	7	0.89	0.071	0.071	1.95	0.156	0.156
4	8	0.79	0.063	0.063	2.00	0.160	0.160
5	1	0.82	0.066	0.066	0.28	0.022	0.022
5	2	0.82	0.065	0.065	0.26	0.021	0.021
5	3	0.84	0.068	0.068	0.24	0.019	0.019
5	4	0.85	0.068	0.068	0.32	0.025	0.025
5	5	1.32	0.106	0.106	0.66	0.052	0.052
5	6	1.55	0.124	0.124	0.71	0.057	0.057
5	7	2.10	0.168	0.168	1.33	0.106	0.106
5	8	2.06	0.165	0.165	1.45	0.116	0.116
			∑ Min	∑ Max		∑ Min	∑ Max
1			0.231	0.231		0.725	0.725
2			0.632	0.632		0.403	0.403
3			0.172	0.172		0.805	0.805
4			0.363	0.363		0.622	0.622
5			0.829	0.829		0.419	0.419
Mean			0.445	0.445		0.595	0.595
SD			0.278	0.278		0.180	0.180

Table H. Continued LDS.

Stone	Fraction	LAB 1 Cu µg/l	min r mg/m^2	max r mg/m^2	LAB 2 Cu µg/l	min r mg/m^2	max r mg/m^2
1	1	4.57	0.366	0.366	0.04	0.003	0.003
1	2	2.31	0.184	0.184	<0.04	0.000	0.003
1	3	1.76	0.141	0.141	<0.04	0.000	0.003
1	4	1.63	0.131	0.131	<0.04	0.000	0.003
1	5	0.92	0.073	0.073	0.08	0.007	0.007
1	6	1.82	0.146	0.146	0.04	0.003	0.003
1	7	1.78	0.143	0.143	0.07	0.005	0.005
1	8	2.11	0.169	0.169	0.09	0.008	0.008
2	1	2.93	0.234	0.234	0.05	0.004	0.004
2	2	1.93	0.154	0.154	<0.04	0.000	0.003
2	3	1.49	0.119	0.119	0.05	0.000	0.004
2	4	1.62	0.130	0.130	<0.04	0.000	0.003
2	5	1.26	0.100	0.100	0.05	0.004	0.004
2	6	1.46	0.117	0.117	0.05	0.004	0.004
2	7	1.91	0.153	0.153	0.11	0.009	0.009
2	8	1.51	0.121	0.121	8.69	0.695	0.695
3	1	3.32	0.266	0.266	0.06	0.005	0.005
3	2	1.65	0.132	0.132	0.07	0.006	0.006
3	3	1.25	0.100	0.100	<0.04	0.000	0.003
3	4	1.27	0.102	0.102	<0.04	0.000	0.003
3	5	1.84	0.147	0.147	0.08	0.007	0.007
3	6	2.58	0.206	0.206	0.11	0.009	0.009
3	7	1.21	0.097	0.097	0.11	0.009	0.009
3	8	3.21	0.256	0.256	0.14	0.011	0.011
4	1	4.04	0.323	0.323	0.74	0.059	0.059
4	2	1.21	0.097	0.097	0.05	0.004	0.004
4	3	1.27	0.102	0.102	<0.04	0.000	0.003
4	4	1.00	0.080	0.080	<0.04	0.000	0.003
4	5	1.29	0.104	0.104	0.10	0.008	0.008
4	6	0.90	0.072	0.072	0.06	0.005	0.005
4	7	2.09	0.168	0.168	0.11	0.009	0.009
4	8	1.63	0.130	0.130	0.11	0.009	0.009
5	1	3.02	0.241	0.241	0.13	0.010	0.010
5	2	1.56	0.125	0.125	0.07	0.006	0.006
5	3	1.28	0.103	0.103	0.06	0.005	0.005
5	4	2.05	0.164	0.164	0.05	0.004	0.004
5	5	0.66	0.053	0.053	0.10	0.008	0.008
5	6	1.33	0.106	0.106	0.07	0.005	0.005
5	7	2.21	0.177	0.177	0.12	0.009	0.009
5	8	1.02	0.082	0.082	0.14	0.011	0.011
			∑ Min	∑ Max		∑ Min	∑ Max
1			1.353	1.353		0.026	0.036
2			1.128	1.128		0.716	0.726
3			1.307	1.307		0.046	0.053
4			1.075	1.075		0.095	0.101
5			1.051	1.051		0.058	0.058
Mean			1.183	1.183		0.188	0.195
SD			0.138	0.138		0.296	0.298

Table H. Continued LDS.

Stone	Fraction	LAB 1 Mn µg/l	min r mg/m²	max r mg/m²	LAB 2 Mn µg/l	min r mg/m²	max r mg/m²
1	1	0.76	0.061	0.061	0.73	0.058	0.058
1	2	0.57	0.046	0.046	0.55	0.044	0.044
1	3	0.54	0.044	0.044	0.48	0.038	0.038
1	4	0.47	0.037	0.037	0.49	0.039	0.039
1	5	0.50	0.040	0.040	0.36	0.029	0.029
1	6	0.66	0.053	0.053	0.33	0.026	0.026
1	7	0.44	0.035	0.035	0.23	0.019	0.019
1	8	0.40	0.032	0.032	0.42	0.034	0.034
2	1	0.77	0.061	0.061	0.78	0.062	0.062
2	2	0.90	0.072	0.072	0.53	0.043	0.043
2	3	0.79	0.063	0.063	0.52	0.041	0.041
2	4	0.63	0.051	0.051	0.52	0.042	0.042
2	5	1.12	0.090	0.090	0.58	0.047	0.047
2	6	1.62	0.130	0.130	0.93	0.075	0.075
2	7	1.27	0.102	0.102	0.33	0.027	0.027
2	8	1.38	0.111	0.111	3.06	0.245	0.245
3	1	0.82	0.065	0.065	0.84	0.067	0.067
3	2	0.71	0.057	0.057	0.64	0.051	0.051
3	3	0.59	0.047	0.047	0.50	0.040	0.040
3	4	0.59	0.047	0.047	0.46	0.037	0.037
3	5	1.08	0.086	0.086	1.04	0.083	0.083
3	6	3.22	0.257	0.257	0.56	0.045	0.045
3	7	2.47	0.198	0.198	0.25	0.020	0.020
3	8	2.86	0.229	0.229	0.25	0.020	0.020
4	1	0.52	0.042	0.042	1.23	0.098	0.098
4	2	0.25	0.020	0.020	0.66	0.053	0.053
4	3	0.30	0.024	0.024	0.54	0.044	0.044
4	4	0.27	0.022	0.022	0.46	0.037	0.037
4	5	0.32	0.025	0.025	0.44	0.035	0.035
4	6	0.26	0.021	0.021	0.46	0.037	0.037
4	7	0.23	0.018	0.018	0.36	0.029	0.029
4	8	0.16	0.013	0.013	0.27	0.022	0.022
5	1	0.28	0.022	0.022	0.44	0.035	0.035
5	2	0.37	0.029	0.029	0.61	0.049	0.049
5	3	0.36	0.029	0.029	0.57	0.046	0.046
5	4	0.39	0.032	0.032	0.50	0.040	0.040
5	5	0.12	0.010	0.010	0.53	0.043	0.043
5	6	0.31	0.025	0.025	0.52	0.042	0.042
5	7	0.24	0.019	0.019	0.32	0.026	0.026
5	8	0.22	0.018	0.018	0.26	0.021	0.021
			∑ Min	∑ Max		∑ Min	∑ Max
1			0.348	0.348		0.288	0.288
2			0.679	0.679		0.581	0.581
3			0.986	0.986		0.364	0.364
4			0.185	0.185		0.353	0.353
5			0.183	0.183		0.301	0.301
Mean			0.476	0.476		0.377	0.377
SD			0.349	0.349		0.118	0.118

Table H. Continued LDS.

Stone	Fraction	LAB 1 Mo µg/l	min r mg/m²	max r mg/m²	LAB 2 Mo µg/l	min r mg/m²	max r mg/m²
1	1	0.09	0.007	0.007	0.20	0.016	0.016
1	2	0.08	0.006	0.006	0.24	0.019	0.019
1	3	0.08	0.006	0.006	0.17	0.013	0.013
1	4	0.10	0.008	0.008	0.18	0.015	0.015
1	5	0.18	0.015	0.015	0.30	0.024	0.024
1	6	0.23	0.019	0.019	0.31	0.025	0.025
1	7	0.42	0.033	0.033	0.49	0.039	0.039
1	8	0.37	0.030	0.030	0.46	0.037	0.037
2	1	0.14	0.011	0.011	0.12	0.009	0.009
2	2	0.16	0.012	0.012	0.13	0.010	0.010
2	3	0.14	0.011	0.011	0.11	0.009	0.009
2	4	0.14	0.011	0.011	0.12	0.010	0.010
2	5	0.28	0.022	0.022	0.23	0.019	0.019
2	6	0.34	0.027	0.027	0.27	0.021	0.021
2	7	0.71	0.057	0.057	0.43	0.034	0.034
2	8	0.59	0.047	0.047	0.44	0.035	0.035
3	1	0.11	0.008	0.008	0.22	0.018	0.018
3	2	0.08	0.006	0.006	0.26	0.021	0.021
3	3	0.09	0.007	0.007	0.25	0.020	0.020
3	4	0.08	0.006	0.006	0.32	0.026	0.026
3	5	0.14	0.011	0.011	0.63	0.051	0.051
3	6	0.14	0.012	0.012	0.72	0.058	0.058
3	7	0.34	0.027	0.027	1.15	0.092	0.092
3	8	0.35	0.028	0.028	1.13	0.091	0.091
4	1	0.13	0.011	0.011	0.22	0.018	0.018
4	2	0.12	0.010	0.010	0.15	0.012	0.012
4	3	0.14	0.011	0.011	0.13	0.011	0.011
4	4	0.12	0.010	0.010	0.15	0.012	0.012
4	5	0.19	0.016	0.016	0.30	0.024	0.024
4	6	0.21	0.017	0.017	0.33	0.026	0.026
4	7	0.34	0.027	0.027	0.49	0.040	0.040
4	8	0.29	0.023	0.023	0.46	0.037	0.037
5	1	0.24	0.019	0.019	<0.11	0.000	0.009
5	2	0.21	0.017	0.017	<0.11	0.000	0.009
5	3	0.22	0.017	0.017	<0.11	0.000	0.009
5	4	0.23	0.019	0.019	0.12	0.009	0.009
5	5	0.42	0.034	0.034	0.20	0.016	0.016
5	6	0.49	0.040	0.040	0.20	0.016	0.016
5	7	0.79	0.063	0.063	0.31	0.025	0.025
5	8	0.74	0.059	0.059	0.28	0.022	0.022
			∑ Min	∑ Max		∑ Min	∑ Max
1			0.124	0.124		0.188	0.188
2			0.199	0.199		0.148	0.148
3			0.106	0.106		0.375	0.375
4			0.124	0.124		0.179	0.179
5			0.267	0.267		0.089	0.115
Mean			0.164	0.164		0.196	0.201
SD			0.068	0.068		0.108	0.102

Table H. Continued LDS.

Stone	Fraction	LAB 1 Ni µg/l	min r mg/m²	max r mg/m²	LAB 2 Ni µg/l	min r mg/m²	max r mg/m²
1	1	0.31	0.025	0.025	0.05	0.004	0.004
1	2	0.25	0.020	0.020	0.02	0.001	0.001
1	3	0.31	0.024	0.024	0.02	0.001	0.001
1	4	0.34	0.027	0.027	0.03	0.002	0.002
1	5	0.07	0.005	0.005	0.04	0.003	0.003
1	6	0.53	0.043	0.043	0.05	0.004	0.004
1	7	0.17	0.013	0.013	0.04	0.003	0.003
1	8	0.16	0.012	0.012	0.11	0.009	0.009
2	1	0.27	0.021	0.021	0.04	0.003	0.003
2	2	0.23	0.018	0.018	0.06	0.005	0.005
2	3	0.29	0.023	0.023	0.05	0.004	0.004
2	4	0.19	0.015	0.015	0.01	0.001	0.001
2	5	0.20	0.016	0.016	0.03	0.002	0.002
2	6	0.25	0.020	0.020	0.02	0.002	0.002
2	7	0.18	0.014	0.014	0.02	0.002	0.002
2	8	0.23	0.019	0.019	0.02	0.002	0.002
3	1	0.26	0.021	0.021	0.09	0.007	0.007
3	2	0.22	0.017	0.017	0.04	0.003	0.003
3	3	0.18	0.015	0.015	0.03	0.002	0.002
3	4	0.16	0.013	0.013	0.02	0.001	0.001
3	5	0.23	0.018	0.018	0.04	0.003	0.003
3	6	1.02	0.082	0.082	0.06	0.005	0.005
3	7	0.17	0.013	0.013	0.03	0.002	0.002
3	8	0.37	0.029	0.029	0.05	0.004	0.004
4	1	0.29	0.023	0.023	3.77	0.302	0.302
4	2	0.17	0.013	0.013	0.04	0.003	0.003
4	3	0.21	0.017	0.017	0.02	0.001	0.001
4	4	0.18	0.014	0.014	0.03	0.002	0.002
4	5	0.17	0.014	0.014	0.05	0.004	0.004
4	6	0.19	0.015	0.015	0.05	0.004	0.004
4	7	0.17	0.013	0.013	0.07	0.005	0.005
4	8	0.16	0.013	0.013	0.03	0.003	0.003
5	1	0.21	0.017	0.017	0.28	0.022	0.022
5	2	0.21	0.017	0.017	0.03	0.003	0.003
5	3	0.25	0.020	0.020	0.03	0.003	0.003
5	4	0.32	0.025	0.025	0.02	0.002	0.002
5	5	0.07	0.006	0.006	0.03	0.002	0.002
5	6	0.31	0.025	0.025	0.04	0.003	0.003
5	7	0.22	0.018	0.018	0.03	0.002	0.002
5	8	0.15	0.012	0.012	0.10	0.008	0.008
			∑ Min	∑ Max		∑ Min	∑ Max
1			0.171	0.171		0.028	0.028
2			0.146	0.146		0.020	0.020
3			0.208	0.208		0.027	0.027
4			0.122	0.122		0.324	0.324
5			0.140	0.140		0.045	0.045
Mean			0.158	0.158		0.089	0.089
SD			0.033	0.033		0.132	0.132

Table H. Continued LDS.

Stone	Fraction	LAB 1 Pb µg/l	min r mg/m²	max r mg/m²	LAB 2 Pb µg/l	min r mg/m²	max r mg/m²
1	1	0.23	0.018	0.018	<0.02	0.000	0.002
1	2	0.16	0.013	0.013	<0.02	0.000	0.002
1	3	0.16	0.013	0.013	<0.02	0.000	0.002
1	4	0.11	0.008	0.008	<0.02	0.000	0.002
1	5	0.30	0.024	0.024	<0.02	0.000	0.002
1	6	0.44	0.035	0.035	<0.02	0.000	0.002
1	7	<0.03	0.000	0.002	<0.02	0.000	0.002
1	8	0.17	0.014	0.014	<0.02	0.000	0.002
2	1	0.26	0.021	0.021	<0.02	0.000	0.002
2	2	0.32	0.025	0.025	<0.02	0.000	0.002
2	3	0.25	0.020	0.020	0.02	0.002	0.002
2	4	0.23	0.019	0.019	<0.02	0.000	0.002
2	5	0.47	0.038	0.038	<0.02	0.000	0.002
2	6	0.52	0.042	0.042	<0.02	0.000	0.002
2	7	0.53	0.042	0.042	<0.02	0.000	0.002
2	8	0.35	0.028	0.028	<0.02	0.000	0.002
3	1	0.38	0.030	0.030	<0.02	0.000	0.002
3	2	0.10	0.008	0.008	<0.02	0.000	0.002
3	3	0.12	0.010	0.010	<0.02	0.000	0.002
3	4	0.07	0.005	0.005	<0.02	0.000	0.002
3	5	0.30	0.024	0.024	<0.02	0.000	0.002
3	6	0.46	0.037	0.037	<0.02	0.000	0.002
3	7	0.09	0.008	0.008	<0.02	0.000	0.002
3	8	0.11	0.009	0.009	<0.02	0.000	0.002
4	1	0.83	0.066	0.066	0.03	0.003	0.003
4	2	0.17	0.013	0.013	<0.02	0.000	0.002
4	3	0.10	0.008	0.008	<0.02	0.000	0.002
4	4	0.10	0.008	0.008	<0.02	0.000	0.002
4	5	0.06	0.005	0.005	<0.02	0.000	0.002
4	6	<0.03	0.000	0.002	<0.02	0.000	0.002
4	7	0.04	0.003	0.003	<0.02	0.000	0.002
4	8	<0.03	0.000	0.002	<0.02	0.000	0.002
5	1	0.39	0.032	0.032	<0.02	0.000	0.002
5	2	0.31	0.025	0.025	<0.02	0.000	0.002
5	3	0.30	0.024	0.024	<0.02	0.000	0.002
5	4	0.14	0.011	0.011	<0.02	0.000	0.002
5	5	0.15	0.012	0.012	<0.02	0.000	0.002
5	6	0.03	0.003	0.003	<0.02	0.000	0.002
5	7	<0.03	0.000	0.002	<0.02	0.000	0.002
5	8	<0.03	0.000	0.002	<0.02	0.000	0.002
			∑ Min	∑ Max		∑ Min	∑ Max
1			0.126	0.129		0.000	0.013
2			0.235	0.235		0.002	0.013
3			0.130	0.130		0.000	0.013
4			0.104	0.109		0.003	0.014
5			0.106	0.110		0.000	0.013
Mean			0.140	0.143		0.001	0.013
SD			0.054	0.053		0.001	0.000

Table H. Continued LDS.

Stone	Fraction	LAB 1 Sb µg/l	min r mg/m^2	max r mg/m^2	LAB 2 Sb µg/l	min r mg/m^2	max r mg/m^2
1	1	0.08	0.006	0.006	<0.54	0.000	0.043
1	2	0.07	0.006	0.006	<0.54	0.000	0.043
1	3	0.07	0.006	0.006	<0.54	0.000	0.043
1	4	0.09	0.007	0.007	<0.54	0.000	0.043
1	5	<0.06	0.000	0.005	<0.54	0.000	0.043
1	6	0.07	0.006	0.006	<0.54	0.000	0.043
1	7	0.32	0.025	0.025	<0.54	0.000	0.043
1	8	0.18	0.014	0.014	<0.54	0.000	0.043
2	1	0.07	0.005	0.005	<0.54	0.000	0.043
2	2	0.07	0.005	0.005	<0.54	0.000	0.043
2	3	0.08	0.006	0.006	<0.54	0.000	0.043
2	4	<0.06	0.000	0.005	<0.54	0.000	0.043
2	5	0.08	0.006	0.006	<0.54	0.000	0.043
2	6	0.28	0.022	0.022	<0.54	0.000	0.043
2	7	0.15	0.012	0.012	<0.54	0.000	0.043
2	8	0.19	0.015	0.015	<0.54	0.000	0.043
3	1	0.07	0.006	0.006	<0.54	0.000	0.043
3	2	<0.06	0.000	0.005	<0.54	0.000	0.043
3	3	<0.06	0.000	0.005	<0.54	0.000	0.043
3	4	0.07	0.006	0.006	<0.54	0.000	0.043
3	5	<0.06	0.000	0.005	<0.54	0.000	0.043
3	6	0.08	0.006	0.006	<0.54	0.000	0.043
3	7	0.29	0.023	0.023	<0.54	0.000	0.043
3	8	0.16	0.013	0.013	<0.54	0.000	0.043
4	1	0.07	0.006	0.006	<0.54	0.000	0.043
4	2	0.08	0.006	0.006	<0.54	0.000	0.043
4	3	0.07	0.006	0.006	<0.54	0.000	0.043
4	4	0.08	0.006	0.006	<0.54	0.000	0.043
4	5	0.08	0.006	0.006	<0.54	0.000	0.043
4	6	0.31	0.025	0.025	<0.54	0.000	0.043
4	7	0.30	0.024	0.024	<0.54	0.000	0.043
4	8	0.20	0.016	0.016	<0.54	0.000	0.043
5	1	0.07	0.005	0.005	<0.54	0.000	0.043
5	2	<0.06	0.000	0.005	<0.54	0.000	0.043
5	3	0.08	0.006	0.006	<0.54	0.000	0.043
5	4	0.09	0.008	0.008	<0.54	0.000	0.043
5	5	<0.06	0.000	0.005	<0.54	0.000	0.043
5	6	0.31	0.025	0.025	<0.54	0.000	0.043
5	7	0.28	0.023	0.023	<0.54	0.000	0.043
5	8	0.22	0.017	0.017	<0.54	0.000	0.043
			∑ Min	∑ Max		∑ Min	∑ Max
1			0.070	0.075		0.000	0.346
2			0.073	0.078		0.000	0.345
3			0.054	0.068		0.000	0.346
4			0.095	0.095		0.000	0.346
5			0.084	0.093		0.000	0.346
Mean			0.075	0.082		0.000	0.346
SD			0.015	0.012		0.000	0.000

Table H. Continued LDS.

Stone	Fraction	LAB 1 Se µg/l	min r mg/m²	max r mg/m²	LAB 2 Se µg/l	min r mg/m²	max r mg/m²
1	1	0.05	0.004	0.004	<1.98	0.000	0.158
1	2	0.05	0.004	0.004	<1.98	0.000	0.158
1	3	0.04	0.003	0.003	<1.98	0.000	0.158
1	4	<0.04	0.000	0.003	<1.98	0.000	0.158
1	5	0.05	0.004	0.004	<1.98	0.000	0.158
1	6	0.04	0.003	0.003	<1.98	0.000	0.158
1	7	0.08	0.007	0.007	<1.98	0.000	0.158
1	8	0.07	0.005	0.005	<1.98	0.000	0.158
2	1	0.08	0.007	0.007	<1.98	0.000	0.158
2	2	0.07	0.005	0.005	<1.98	0.000	0.158
2	3	0.07	0.005	0.005	<1.98	0.000	0.158
2	4	0.06	0.005	0.005	<1.98	0.000	0.158
2	5	0.11	0.009	0.009	<1.98	0.000	0.158
2	6	0.08	0.007	0.007	<1.98	0.000	0.158
2	7	0.11	0.009	0.009	<1.98	0.000	0.158
2	8	0.08	0.007	0.007	<1.98	0.000	0.158
3	1	0.08	0.006	0.006	<1.98	0.000	0.158
3	2	0.08	0.006	0.006	<1.98	0.000	0.158
3	3	0.07	0.006	0.006	<1.98	0.000	0.158
3	4	0.06	0.005	0.005	<1.98	0.000	0.158
3	5	0.09	0.007	0.007	<1.98	0.000	0.158
3	6	0.14	0.012	0.012	<1.98	0.000	0.158
3	7	0.19	0.015	0.015	<1.98	0.000	0.158
3	8	0.15	0.012	0.012	<1.98	0.000	0.158
4	1	0.06	0.005	0.005	<1.98	0.000	0.158
4	2	0.05	0.004	0.004	<1.98	0.000	0.158
4	3	0.05	0.004	0.004	<1.98	0.000	0.158
4	4	<0.04	0.000	0.003	<1.98	0.000	0.158
4	5	0.07	0.006	0.006	<1.98	0.000	0.158
4	6	0.06	0.004	0.004	<1.98	0.000	0.158
4	7	0.10	0.008	0.008	<1.98	0.000	0.158
4	8	0.06	0.005	0.005	<1.98	0.000	0.158
5	1	0.07	0.005	0.005	<1.98	0.000	0.158
5	2	0.05	0.004	0.004	<1.98	0.000	0.158
5	3	0.05	0.004	0.004	<1.98	0.000	0.158
5	4	0.05	0.004	0.004	<1.98	0.000	0.158
5	5	0.08	0.006	0.006	<1.98	0.000	0.158
5	6	0.07	0.006	0.006	<1.98	0.000	0.158
5	7	0.10	0.008	0.008	<1.98	0.000	0.158
5	8	0.08	0.006	0.006	<1.98	0.000	0.158
			∑ Min	∑ Max		∑ Min	∑ Max
1			0.030	0.033		0.000	1.267
2			0.053	0.053		0.000	1.267
3			0.069	0.069		0.000	1.268
4			0.036	0.039		0.000	1.267
5			0.043	0.043		0.000	1.267
Mean			0.046	0.047		0.000	1.267
SD			0.015	0.014		0.000	0.000

Table H. Continued LDS.

Stone	Fraction	LAB 1 Sn µg/l	min r mg/m²	max r mg/m²	LAB 2 Sn µg/l	min r mg/m²	max r mg/m²
1	1	0.12	0.010	0.010	<0.10	0.000	0.008
1	2	0.09	0.008	0.008	<0.10	0.000	0.008
1	3	0.08	0.006	0.006	<0.10	0.000	0.008
1	4	0.06	0.005	0.005	<0.10	0.000	0.008
1	5	0.08	0.007	0.007	<0.10	0.000	0.008
1	6	0.06	0.004	0.004	<0.10	0.000	0.008
1	7	0.11	0.008	0.008	<0.10	0.000	0.008
1	8	0.12	0.009	0.009	<0.10	0.000	0.008
2	1	0.18	0.014	0.014	<0.10	0.000	0.008
2	2	0.13	0.010	0.010	<0.10	0.000	0.008
2	3	0.10	0.008	0.008	<0.10	0.000	0.008
2	4	0.08	0.007	0.007	<0.10	0.000	0.008
2	5	0.06	0.005	0.005	<0.10	0.000	0.008
2	6	0.06	0.004	0.004	<0.10	0.000	0.008
2	7	0.10	0.008	0.008	<0.10	0.000	0.008
2	8	0.05	0.004	0.004	<0.10	0.000	0.008
3	1	0.23	0.019	0.019	<0.10	0.000	0.008
3	2	0.16	0.013	0.013	<0.10	0.000	0.008
3	3	0.15	0.012	0.012	<0.10	0.000	0.008
3	4	0.10	0.008	0.008	<0.10	0.000	0.008
3	5	0.15	0.012	0.012	<0.10	0.000	0.008
3	6	0.05	0.004	0.004	<0.10	0.000	0.008
3	7	0.14	0.011	0.011	<0.10	0.000	0.008
3	8	0.06	0.005	0.005	<0.10	0.000	0.008
4	1	0.31	0.025	0.025	<0.10	0.000	0.008
4	2	0.22	0.018	0.018	<0.10	0.000	0.008
4	3	0.22	0.017	0.017	<0.10	0.000	0.008
4	4	0.10	0.008	0.008	<0.10	0.000	0.008
4	5	0.07	0.006	0.006	<0.10	0.000	0.008
4	6	0.05	0.004	0.004	<0.10	0.000	0.008
4	7	0.20	0.016	0.016	<0.10	0.000	0.008
4	8	0.10	0.008	0.008	<0.10	0.000	0.008
5	1	0.20	0.016	0.016	<0.10	0.000	0.008
5	2	0.14	0.011	0.011	<0.10	0.000	0.008
5	3	0.13	0.010	0.010	<0.10	0.000	0.008
5	4	0.11	0.008	0.008	<0.10	0.000	0.008
5	5	0.06	0.005	0.005	<0.10	0.000	0.008
5	6	0.05	0.004	0.004	<0.10	0.000	0.008
5	7	0.14	0.011	0.011	<0.10	0.000	0.008
5	8	0.05	0.004	0.004	<0.10	0.000	0.008
			∑ Min	∑ Max		∑ Min	∑ Max
1			0.058	0.058		0.000	0.064
2			0.060	0.060		0.000	0.064
3			0.084	0.084		0.000	0.064
4			0.102	0.102		0.000	0.064
5			0.070	0.070		0.000	0.064
Mean			0.075	0.075		0.000	0.064
SD			0.018	0.018		0.000	0.000

Table H. Continued LDS.

Stone	Fraction	LAB 1 Sr µg/l	min r mg/m²	max r mg/m²	LAB 2 Sr µg/l	min r mg/m²	max r mg/m²
1	1	9.25	0.74	0.74	3.45	0.28	0.28
1	2	10.64	0.85	0.85	5.44	0.44	0.44
1	3	12.28	0.98	0.98	6.57	0.53	0.53
1	4	14.32	1.15	1.15	8.38	0.67	0.67
1	5	19.19	1.54	1.54	14.69	1.18	1.18
1	6	31.25	2.50	2.50	19.14	1.53	1.53
1	7	86.17	6.89	6.89	30.57	2.45	2.45
1	8	173.20	13.85	13.85	33.78	2.70	2.70
2	1	7.55	0.60	0.60	3.38	0.27	0.27
2	2	9.34	0.75	0.75	6.30	0.50	0.50
2	3	10.63	0.85	0.85	7.89	0.63	0.63
2	4	8.32	0.67	0.67	9.97	0.80	0.80
2	5	16.45	1.32	1.32	20.71	1.66	1.66
2	6	18.94	1.52	1.52	29.10	2.33	2.33
2	7	19.68	1.57	1.57	48.77	3.90	3.90
2	8	22.96	1.84	1.84	59.47	4.76	4.76
3	1	8.85	0.71	0.71	3.45	0.28	0.28
3	2	5.25	0.42	0.42	6.74	0.54	0.54
3	3	6.65	0.53	0.53	8.85	0.71	0.71
3	4	8.30	0.66	0.66	13.41	1.07	1.07
3	5	14.72	1.18	1.18	28.38	2.27	2.27
3	6	18.12	1.45	1.45	36.22	2.90	2.90
3	7	22.06	1.77	1.77	56.10	4.49	4.49
3	8	21.99	1.76	1.76	60.17	4.82	4.82
4	1	14.28	1.14	1.14	4.96	0.40	0.40
4	2	22.99	1.84	1.84	6.03	0.48	0.48
4	3	26.79	2.14	2.14	7.77	0.62	0.62
4	4	26.10	2.09	2.09	9.94	0.80	0.80
4	5	44.02	3.52	3.52	19.05	1.52	1.52
4	6	53.37	4.27	4.27	21.65	1.73	1.73
4	7	81.15	6.49	6.49	34.57	2.77	2.77
4	8	86.84	6.95	6.95	35.98	2.88	2.88
5	1	8.78	0.70	0.70	3.72	0.30	0.30
5	2	18.93	1.51	1.51	6.45	0.52	0.52
5	3	24.71	1.98	1.98	8.12	0.65	0.65
5	4	28.19	2.26	2.26	11.58	0.93	0.93
5	5	35.50	2.84	2.84	20.47	1.64	1.64
5	6	48.55	3.88	3.88	26.58	2.13	2.13
5	7	61.95	4.96	4.96	45.72	3.66	3.66
5	8	40.02	3.20	3.20	53.61	4.29	4.29
			∑ Min	∑ Max		∑ Min	∑ Max
1			28.50	28.50		9.76	9.76
2			9.11	9.11		14.84	14.84
3			8.48	8.48		17.07	17.07
4			28.44	28.44		11.20	11.20
5			21.33	21.33		14.10	14.10
Mean			19.17	19.17		13.39	13.39
SD			9.92	9.92		2.92	2.92

Table H. Continued LDS.

Stone	Fraction	LAB 1 U µg/l	min r mg/m^2	max r mg/m^2	LAB 2 U µg/l	min r mg/m^2	max r mg/m^2
1	1	<0.04	0.000	0.003	<0.02	0.000	0.002
1	2	<0.04	0.000	0.003	<0.02	0.000	0.002
1	3	<0.04	0.000	0.003	<0.02	0.000	0.002
1	4	<0.04	0.000	0.003	<0.02	0.000	0.002
1	5	<0.04	0.000	0.003	<0.02	0.000	0.002
1	6	<0.04	0.000	0.003	<0.02	0.000	0.002
1	7	<0.04	0.000	0.003	<0.02	0.000	0.002
1	8	<0.04	0.000	0.003	<0.02	0.000	0.002
2	1	<0.04	0.000	0.003	<0.02	0.000	0.002
2	2	<0.04	0.000	0.003	<0.02	0.000	0.002
2	3	<0.04	0.000	0.003	<0.02	0.000	0.002
2	4	<0.04	0.000	0.003	<0.02	0.000	0.002
2	5	<0.04	0.000	0.003	<0.02	0.000	0.002
2	6	<0.04	0.000	0.003	<0.02	0.000	0.002
2	7	<0.04	0.000	0.003	<0.02	0.000	0.002
2	8	<0.04	0.000	0.003	<0.02	0.000	0.002
3	1	<0.04	0.000	0.003	<0.02	0.000	0.002
3	2	<0.04	0.000	0.003	<0.02	0.000	0.002
3	3	<0.04	0.000	0.003	<0.02	0.000	0.002
3	4	<0.04	0.000	0.003	<0.02	0.000	0.002
3	5	<0.04	0.000	0.003	<0.02	0.000	0.002
3	6	<0.04	0.000	0.003	<0.02	0.000	0.002
3	7	<0.04	0.000	0.003	<0.02	0.000	0.002
3	8	<0.04	0.000	0.003	<0.02	0.000	0.002
4	1	<0.04	0.000	0.003	<0.02	0.000	0.002
4	2	<0.04	0.000	0.003	<0.02	0.000	0.002
4	3	<0.04	0.000	0.003	<0.02	0.000	0.002
4	4	<0.04	0.000	0.003	<0.02	0.000	0.002
4	5	<0.04	0.000	0.003	<0.02	0.000	0.002
4	6	<0.04	0.000	0.003	<0.02	0.000	0.002
4	7	<0.04	0.000	0.003	<0.02	0.000	0.002
4	8	<0.04	0.000	0.003	<0.02	0.000	0.002
5	1	<0.04	0.000	0.003	<0.02	0.000	0.002
5	2	<0.04	0.000	0.003	<0.02	0.000	0.002
5	3	<0.04	0.000	0.003	<0.02	0.000	0.002
5	4	<0.04	0.000	0.003	<0.02	0.000	0.002
5	5	<0.04	0.000	0.003	<0.02	0.000	0.002
5	6	<0.04	0.000	0.003	<0.02	0.000	0.002
5	7	<0.04	0.000	0.003	<0.02	0.000	0.002
5	8	<0.04	0.000	0.003	<0.02	0.000	0.002
			∑ Min	∑ Max		∑ Min	∑ Max
1			0.000	0.026		0.000	0.013
2			0.000	0.026		0.000	0.013
3			0.000	0.026		0.000	0.013
4			0.000	0.026		0.000	0.013
5			0.000	0.026		0.000	0.013
Mean			0.000	0.026		0.000	0.013
SD			0.000	0.000		0.000	0.000

Table H. Continued LDS.

Stone	Fraction	LAB 1 V µg/l	min r mg/m^2	max r mg/m^2	LAB 2 V µg/l	min r mg/m^2	max r mg/m^2
1	1	15.2	1.21	1.21	14.9	1.19	1.19
1	2	20.8	1.67	1.67	36.2	2.90	2.90
1	3	27.8	2.23	2.23	41.0	3.28	3.28
1	4	34.8	2.78	2.78	53.8	4.30	4.30
1	5	78.7	6.30	6.30	100.3	8.03	8.03
1	6	97.7	7.81	7.81	111.6	8.93	8.93
1	7	33.1	2.65	2.65	167.2	13.38	13.38
1	8	1.47	0.12	0.12	141.8	11.35	11.35
2	1	13.4	1.07	1.07	15.1	1.21	1.21
2	2	27.3	2.18	2.18	30.4	2.43	2.43
2	3	37.5	3.00	3.00	32.8	2.62	2.62
2	4	46.1	3.69	3.69	38.2	3.06	3.06
2	5	99.0	7.92	7.92	68.8	5.50	5.50
2	6	110	8.81	8.81	77.2	6.17	6.17
2	7	191	15.31	15.31	110.0	8.79	8.79
2	8	138	11.08	11.08	102.1	8.17	8.17
3	1	4.51	0.36	0.36	24.2	1.94	1.94
3	2	5.01	0.40	0.40	49.7	3.97	3.97
3	3	5.23	0.42	0.42	57.8	4.63	4.63
3	4	5.77	0.46	0.46	77.2	6.18	6.18
3	5	8.67	0.69	0.69	150.5	12.05	12.05
3	6	12.85	1.03	1.03	167.7	13.42	13.42
3	7	15.49	1.24	1.24	241.5	19.33	19.33
3	8	11.66	0.93	0.93	220.4	17.64	17.64
4	1	101	8.08	8.08	20.1	1.61	1.61
4	2	172	13.74	13.74	36.8	2.94	2.94
4	3	201	16.10	16.10	39.2	3.14	3.14
4	4	160	12.78	12.78	44.7	3.58	3.58
4	5	285	22.78	22.78	82.5	6.60	6.60
4	6	438	35.03	35.03	78.1	6.25	6.25
4	7	499	39.94	39.94	114.4	9.15	9.15
4	8	519	41.51	41.51	101.5	8.13	8.13
5	1	49.0	3.92	3.92	14.4	1.16	1.16
5	2	79.0	6.32	6.32	29.6	2.37	2.37
5	3	90.5	7.24	7.24	31.4	2.51	2.51
5	4	98.0	7.84	7.84	39.4	3.15	3.15
5	5	176	14.08	14.08	72.6	5.81	5.81
5	6	204	16.33	16.33	74.9	5.99	5.99
5	7	257	20.57	20.57	123.3	9.86	9.86
5	8	256	20.46	20.46	121.4	9.71	9.71
			∑ Min	∑ Max		∑ Min	∑ Max
1			24.8	24.8		53.36	53.36
2			53.1	53.1		37.96	37.96
3			5.5	5.5		79.15	79.15
4			189.9	189.9		41.40	41.40
5			96.8	96.8		40.56	40.56
Mean			74.0	74.0		50.49	50.49
SD			73.3	73.3		17.09	17.09

Table H. Continued LDS.

Stone	Fraction	LAB 1 Zn µg/l	min r mg/m^2	max r mg/m^2	LAB 2 Zn µg/l	min r mg/m^2	max r mg/m^2
1	1	227	18	18	0.71	0.057	0.057
1	2	433	35	35	0.33	0.026	0.026
1	3	437	35	35	0.35	0.028	0.028
1	4	464	37	37	0.59	0.047	0.047
1	5	27	2	2	0.47	0.038	0.038
1	6	406	32	32	0.42	0.033	0.033
1	7	340	27	27	0.80	0.064	0.064
1	8	351	28	28	0.45	0.036	0.036
2	1	283	23	23	0.89	0.071	0.071
2	2	425	34	34	0.35	0.028	0.028
2	3	271	22	22	0.40	0.032	0.032
2	4	255	20	20	0.35	0.028	0.028
2	5	249	20	20	0.59	0.047	0.047
2	6	317	25	25	0.45	0.036	0.036
2	7	100	8	8	0.51	0.041	0.041
2	8	215	17	17	0.66	0.053	0.053
3	1	415	33	33	0.60	0.048	0.048
3	2	188	15	15	0.54	0.043	0.043
3	3	234	19	19	0.55	0.044	0.044
3	4	299	24	24	0.38	0.030	0.030
3	5	307	25	25	0.80	0.064	0.064
3	6	499	40	40	0.93	0.074	0.074
3	7	272	22	22	0.51	0.041	0.041
3	8	423	34	34	0.60	0.048	0.048
4	1	418	33	33	1.75	0.140	0.140
4	2	173	14	14	0.30	0.024	0.024
4	3	321	26	26	0.42	0.034	0.034
4	4	206	16	16	0.66	0.053	0.053
4	5	349	28	28	0.41	0.033	0.033
4	6	199	16	16	0.38	0.030	0.030
4	7	202	16	16	0.56	0.045	0.045
4	8	143	11	11	0.63	0.051	0.051
5	1	178	14	14	0.68	0.054	0.054
5	2	426	34	34	0.30	0.024	0.024
5	3	189	15	15	0.44	0.035	0.035
5	4	437	35	35	0.35	0.028	0.028
5	5	28	2	2	0.50	0.040	0.040
5	6	169	14	14	0.46	0.037	0.037
5	7	196	16	16	0.89	0.072	0.072
5	8	178	14	14	0.61	0.049	0.049
			∑ Min	∑ Max		∑ Min	∑ Max
1			215	215		0.330	0.330
2			169	169		0.336	0.336
3			211	211		0.393	0.393
4			161	161		0.410	0.410
5			144	144		0.339	0.339
Mean			180	180		0.362	0.362
SD			31	31		0.037	0.037

Table I. Conductivity and pH values of the DSLTs measured by LAB2.

		Karbon Quartzite		Basalt		Granodiorite		Granite		CUS1		CUS2		CUS3		LDS	
**time [h]**	**time [d]**	**pH**	**conductivity [µS/cm]**	**pH**	**conductivity [µS/cm]**	**pH**	**conductivity [µS/cm]**	**pH**	**conductivity [µS/cm]**	**pH**	**conductivity [µS/cm]**	**pH**	**conductivity [µS/cm]**	**pH**	**conductivity [µS/cm]**	**pH**	**conductivity [µS/cm]**
6	0.25	6.6	3.6	6.5	5.7	6.5	2.7	5.7	3.3	6.2	8.0	6.0	6.3			9.0	18.4
18	1	6.5	1.5	6.2	4.9	6.5	2.4	5.8	2.1	6.2	3.5	6.1	2.0			9.1	26.8
30	2.25	6.2	1.9	6.3	3.8	6.4	2.5	5.7	1.6	6.3	2.5	6.0	2.3			9.4	28.9
42	4	6.2	1.5	6.5	3.8	6.5	2.5	6.6	1.5	6.1	2.2	6.1	2.6			9.7	41.6
5	9	6.4	2.6	6.7	6.2	6.6	4.5	6.0	1.9	6.1	2.7	6.2	1.5			9.7	72.6
7	16	6.5	2.8	6.9	6.4	6.9	3.9	6.1	1.9	6.1	2.6	6.2	1.5			10.1	99.5
20	36	6.7	3.5	7.0	14.1	6.8	8.4	6.6	2.5	6.3	3.6	6.1	2.4			11.4	185.8
28	64	6.3	3.5	7.2	10.3	8.0	7.0	6.9	2.5	6.2	3.6	6.3	2.8			10.8	257.4
6	0.25	6.4	4.6	6.8	3.4	6.6	2.3	6.3	2.5	6.6	5.3	6.2	2.9	5.9	3.2	9.2	20.1
18	1	6.4	2.5	6.1	3.2	6.9	1.9	6.3	3.1	6.5	4.0	6.1	1.3	5.8	3.5	9.6	35.6
30	2.25	6.2	4.0	6.4	2.1	6.6	2.2	6.4	3.8	6.5	3.1	6.1	1.7	6.0	2.1	10.0	49.1
42	4	6.1	3.7	6.3	2.9	6.6	2.1	6.7	4.9	6.2	2.1	6.2	1.5	5.8	1.8	10.3	77.0
5	9	6.4	9.2	6.6	3.0	6.6	4.4	6.8	10.6	6.1	2.7	6.2	1.9	6.0	2.8	10.6	187.3
7	16	6.7	11.8	6.9	3.4	6.9	4.4	7.0	12.2	6.3	2.3	6.1	1.9	6.0	3.8	10.9	291.5
20	36	6.8	24.3	6.9	8.4	6.8	7.5	7.4	21.1	6.3	3.1	6.2	3.3	6.2	2.5	11.4	600.0
28	64	7.2	25.9	6.9	5.3	7.5	7.3	7.0	24.3	6.3	3.0	6.3	3.3	6.0	2.3	11.5	742.0
6	0.25	6.0	3.5	6.8	3.5	6.6	2.0	6.7	1.3	6.6	3.9	6.2	4.7	5.9	6.0	9.2	19.7
18	1	6.0	1.4	6.3	2.7	6.4	1.3	6.0	1.5	6.3	2.5	6.3	1.9	6.1	1.8	9.5	33.1
30	2.25	6.0	1.3	6.5	2.4	6.6	1.3	5.7	1.3	6.4	1.7	6.3	1.7	6.0	2.3	9.7	43.0
42	4	5.8	1.4	6.3	2.4	6.5	1.3	6.1	1.3	6.3	2.3	6.3	1.6	6.1	2.7	10.1	76.2
5	9	6.0	2.0	6.6	3.7	6.6	2.3	6.4	2.1	6.0	1.8	6.4	2.0	6.1	2.9	10.6	173.6
7	16	5.9	3.0	6.9	4.2	6.9	2.5	6.6	2.3	6.0	1.6	6.3	1.9	6.3	4.7	10.8	237.9
20	36	6.0	3.5	6.9	11.1	6.9	5.2	6.9	3.7	6.3	2.4	6.2	3.6	6.2	2.8	11.2	419.0
28	64	5.9	3.6	6.9	6.5	6.8	5.1	6.9	3.7	6.3	2.7	6.4	2.9	6.2	3.1	11.2	478.0

Table I. Continued conductivity and pH values of the DSLTs measured by LAB2.

		Karbon Quartzite		Basalt		Granodiorite		Granite		CUS1		CUS2		CUS3		LDS	
**time [h]**	**time [d]**	**pH**	**conductivity [μS/cm]**	**pH**	**conductivity [μS/cm]**	**pH**	**conductivity [μS/cm]**	**pH**	**conductivity [μS/cm]**	**pH**	**conductivity [μS/cm]**	**pH**	**conductivity [μS/cm]**	**pH**	**conductivity [μS/cm]**	**pH**	**conductivity [μS/cm]**
6	0.25	6.1	1.8	6.8	2.4	6.8	5.9	6.3	3.1	6.3	7.1	6.0	4.1	6.0	2.6	9.2	24.8
18	1	5.9	1.3	5.8	2.1	6.6	2.4	6.7	4.6	6.3	4.0	6.1	1.2	5.9	1.5	9.6	32.2
30	2.25	6.2	1.2	6.5	2.1	6.8	2.4	7.0	5.3	6.5	3.0	6.0	1.4	6.0	2.5	9.7	39.3
42	4	7.0	1.3	6.1	2.4	6.9	2.4	6.6	6.6	6.4	2.4	6.1	1.4	6.0	2.0	10.0	57.2
5	9	6.3	1.7	6.4	4.7	6.9	4.7	6.9	14.3	6.1	3.1	6.3	1.2	6.2	2.1	10.4	122.2
7	16	6.1	2.0	6.7	5.7	7.2	4.4	7.4	17.3	6.2	3.4	6.2	1.4	6.4	2.2	10.6	162.2
20	36	6.3	2.7	6.9	15.6	7.2	9.0	7.5	29.5	6.3	4.7	6.1	2.2	6.3	3.3	11.0	312.0
28	64	6.2	2.7	7.0	11.1	6.7	8.0	7.3	33.2	6.3	4.8	6.3	2.3	6.2	2.6	11.2	411.0
6	0.25	6.4	2.3	6.6	3.1	7.1	3.8	6.3	1.4	5.9	5.9	6.0	5.0	6.0	3.2	8.8	13.7
18	1	5.9	1.3	6.2	2.5	6.4	2.9	6.2	1.3	6.2	3.3	6.1	1.8	5.8	1.7	9.5	24.5
30	2.25	6.4	1.1	6.5	2.2	6.9	3.5	6.1	1.8	6.7	1.7	6.2	1.9	5.9	2.4	9.5	29.3
42	4	7.1	1.2	6.1	3.3	7.0	3.7	6.3	1.9	6.1	1.4	6.2	2.1	5.9	1.9	9.8	45.0
5	9	6.4	1.8	6.4	2.9	7.3	7.7	6.7	2.9	6.2	2.1	6.5	3.7	6.1	2.1	10.2	90.8
7	16	7.0	1.8	6.9	3.3	7.2	8.1	7.0	2.9	6.1	1.8	6.3	2.1	6.1	1.9	10.5	124.0
20	36	6.3	2.5	6.7	13.6	7.3	18.3	6.6	4.1	6.2	2.8	6.3	44.7	6.2	2.3	11.0	264.0
28	64	6.0	2.6	6.8	4.9	6.9	18.2	6.9	3.8	6.1	2.9	6.6	4.5	6.1	2.5	11.1	339.0

DSLT practical considerations and potential improvements of potential following technical specification The blank criterion defined by the technical specification can be summarized as: 1. Performance of a vessel leaching test over 24 h, preparation of the sample as the others; 2. repetition of step 1 and measurement of the conductivity; 3. the frequency of blank testing is related to the number of tests performed by a laboratory; 4. in the first eluate of the blank test, the concentration of each substance considered, shall be less than 10% of the calculated average concentration in the first three eluate fractions of the product test, or below the limit of quantification; 5. the electrical conductivity of the second blank eluate shall be <0.5 mS/m and 6. if these requirements are not fulfilled, the necessary steps to reduce the contamination have to be taken.

Two main reasons for not fulfilling the blank criteria in this study are visible (Table J and K): The first is based on the 10% criterion of the TS [3] (Table L LAB2) which is difficult to fulfill if the material is inert in combination with low LoQs. The other reason is caused by contaminations (Table J LAB1, Cu, Ni, Pb, Sn and V) and may occur in addition to the first. In point 6.2 of the TS the quality of the leachate (resp. eluent) is defined: "Use as a leachant demineralized water or deionized water or water of equivalent purity with a conductivity < 0.5 mS/m according to grade 3 specified by EN ISO 3696 [2]. In this study the contract laboratory fulfilled this criterion, but the blanks analyzed by the authors revealed that the Cu, Ni, Pb and Zn data could not be used in the product evaluation due to elevated background values and, hence, had to be excluded. For these two reasons ((i) the stricter, the better and (ii) quality criterion of the leachant < 0.5 mS/m) from an applied analytical point of view the blank test criterion as defined by the TS does not help ensure to gain reliable data. In addition, the technical specification does not deliver provision on how the data set has to be treated if quality criteria are not fulfilled. With respect to contracting and

commissioning, it seems easily possible to demand additional and stricter criteria for good laboratory practice. Regarding regulative demands, it is questionable if in case of a harmonized EU document like the TS it is possible to demand stricter criteria on blank values in nationally based daughter specification. From the authors point of view there is no practical benefit from the blank test as defined by the TS [3] if no additional quality criteria are required. In the recent version of the TS it is not possible to distinguish between exceedance due to very low limits of quantification (e.g., in ICP-MS analyses) combined with high quality products tested and exceedance caused by contamination from e.g., leachants that contain brass alloy metals since both end up with evaluation: blank criterion not fulfilled.

Reduced test times

To reduce the manufacturers' and costumers workload DSLTs with shorter test times should be available after the initial product testing. Shortening the test from 64 days with eight fractions, to nine days and five fractions delivers, on the one hand enough reliability with respect to the release (significantly after peak release and enough samples to identify outliers). On the other hand, this shorter test is fast and cost efficient in the continuous product monitoring. Table A10 compares the nine days test MRLs with the results from the DSLTs. To derive the nine days MRLs in the calculation in Table 2 64 d was exchanged by 9 d taking into account that with this approach stricter criteria are defined (in this setup the peak release strongly impacts the evaluation). Hence, the future procedure after initial material testing might be that all materials are tested with nine days DSLTs. For materials that fulfill the slightly stricter criteria, no need for 64 d monitoring tests is given. Materials which do not fulfill the nine days criteria must be tested over the full timespan and must comply with the 64 d criteria.

Table J. Mean release values [mg/m^2/9d] of n = 5 DSLTs (Lab2; Table B-H. SD = standard deviation) compared to MRLs. Values > low frequency exchange MRL = italic. underlined and light grey; values > high frequency exchange value 6 h = bold. underlined and dark grey. *EQS Cr(III) **<LoQs; the LoQs are given in Table B-H for the respective elements. All data based on DSLTs and analyses performed by BfG.

Analyte	As	SD	Ba	SD	Cd	SD	Cr*	SD	Cu	SD	Ni	SD	Pb	SD	Se	SD	Sn	SD	V	SD	Zn	SD
**MRL 24h**	0.1	-	9.0	-	0.04	-	0.5	-	1.2	-	0.6	-	0.2	-	0.4	-	0.5	-	0.4	-	1.6	-
**MRL 6h**	0.6	-	36	-	0.1	-	2.0	-	4.7	-	2.4	-	0.7	-	1.8	-	2.1	-	1.4	-	6.6	-
**Karbon Quarzite**	0.02	0.005	0.49	0.61	<LoQ	-	0.05	0.06	<LoQ	-	0.03	0.02	<LoQ	-	<LoQ	-	<LoQ	-	<LoQ	-	0.21	0.02
**Basalt**	<LoQ	-	0.14	0.03	<LoQ	-	<LoQ	-	0.33	0.30	0.02	0.00	0.02	0.01	<LoQ	-	<LoQ	-	0.09	0.07	0.33	0.27
**Greywacke**	0.06	0.03	2.84	1.55	<LoQ	-	0.01	0.01	0.02	0.01	0.03	0.01	0.02	0.02	0.03	0.005	<LoQ	-	0.01	0.002	0.72	0.37
**Granodiorite**	0.04	0.01	0.41	0.02	<LoQ	-	<LoQ	-	0.05	0.04	0.06	0.07	0.01	0.001	<LoQ	-	<LoQ	-	0.02	0.01	0.30	0.21
**Granite**	*0.18*	0.17	0.12	0.02	<LoQ	-	0.01	0.00	0.02	0.01	0.01	0.01	0.005	0.001	<LoQ	-	<LoQ	-	0.01	0.004	*3.47*	2.41
**CUS1**	*0.33*	0.07	2.0	0.26	**0.11**	0.08	<LoQ	-	**9.98**	3.16	*1.38*	0.99	*0.22*	0.13	<LoQ	-	<LoQ	-	<LoQ	-	**23**	12
**CUS2**	*0.20*	0.09	0.56	0.24	**0.14**	0.06	<LoQ	-	**29**	9.2	**2.8**	2.3	**7.0**	5.1	<LoQ	-	<LoQ	-	<LoQ	-	**16**	5.9
**CUS3**	*0.45*	0.31	0.34	0.21	*0.05*	0.01	<LoQ	-	**28**	7.8	*1.5*	1.2	0.1	0.04	<LoQ	-	<LoQ	-	0.02	4E-04	**6.8**	1.7
**LDS**	<LoQ	-	0.79	0.20	<LoQ	-	0.22	0.06	0.03	0.03	0.08	0.13	0.01	4E-04	<LoQ	-	<LoQ	-	**19**	5.71	0.22	0.04

Table K. Evaluation of the blank criteria as described by the TS [3] LAB1.

material	stone	As	Ba	Cd	Cr	Cu	Ni	Pb	Se	Sn	V	Zn	criterion fulfilled	criterion not fulfilled
Karbon Quartzite	1	yes	yes	yes	yes	**no**	**no**	**no**	yes	yes	**no**	yes	7	4
	2	yes	yes	yes	yes	**no**	**no**	**no**	yes	yes	**no**	yes	7	4
	3	yes	yes	yes	yes	**no**	**no**	**no**	yes	yes	**no**	yes	7	4
	4	yes	yes	yes	yes	**no**	**no**	**no**	yes	yes	**no**	yes	7	4
	5	yes	yes	yes	yes	**no**	**no**	**no**	yes	yes	**no**	yes	7	4
Basalt	1	yes	yes	yes	yes	**no**	**no**	**no**	yes	**no**	**no**	yes	6	5
	2	yes	yes	yes	yes	**no**	**no**	**no**	yes	**no**	**no**	yes	6	5
	3	yes	yes	yes	yes	**no**	**no**	**no**	yes	**no**	**no**	yes	6	5
	4	yes	yes	yes	yes	**no**	**no**	**no**	yes	**no**	yes	yes	7	4
	5	yes	yes	yes	yes	**no**	**no**	**no**	yes	**no**	yes	yes	7	4
Greywacke	1	yes	yes	yes	yes	**no**	yes	**no**	yes	**no**	**no**	yes	7	4
	2	yes	yes	yes	yes	**no**	**no**	**no**	yes	**no**	**no**	yes	6	5
	3	yes	yes	yes	yes	**no**	**no**	**no**	yes	**no**	**no**	yes	6	5
	4	yes	yes	yes	yes	**no**	**no**	**no**	yes	**no**	**no**	yes	6	5
	5	yes	yes	yes	yes	**no**	**no**	**no**	yes	**no**	**no**	yes	6	5
Granodiorite	1	yes	yes	yes	yes	**no**	**no**	**no**	yes	**no**	**no**	yes	6	5
	2	yes	yes	yes	yes	**no**	**no**	**no**	yes	**no**	**no**	yes	6	5
	3	yes	yes	yes	yes	**no**	**no**	**no**	yes	**no**	**no**	yes	6	5
	4	yes	yes	yes	yes	**no**	**no**	**no**	yes	**no**	**no**	yes	6	5
	5	yes	yes	yes	yes	**no**	**no**	**no**	yes	**no**	**no**	yes	6	5
Granite	1	yes	yes	yes	yes	**no**	**no**	**no**	yes	**no**	**no**	yes	6	5
	2	yes	yes	yes	yes	**no**	**no**	**no**	yes	**no**	**no**	yes	6	5
	3	yes	yes	yes	yes	**no**	**no**	**no**	yes	**no**	**no**	yes	6	5
	4	yes	yes	yes	yes	**no**	**no**	**no**	yes	**no**	**no**	yes	6	5
	5	yes	yes	yes	yes	**no**	**no**	**no**	yes	**no**	**no**	yes	6	5
CUS1	1	yes	yes	yes	yes	**no**	**no**	**no**	yes	**no**	**no**	yes	6	5
	2	yes	yes	yes	yes	**no**	**no**	**no**	yes	**no**	**no**	yes	6	5
	3	yes	yes	yes	yes	**no**	**no**	**no**	yes	**no**	**no**	yes	6	5
	4	yes	yes	yes	yes	**no**	**no**	**no**	yes	**no**	**no**	yes	6	5
	5	yes	yes	yes	yes	**no**	**no**	**no**	yes	**no**	**no**	yes	6	5
LDS	1	yes	yes	yes	yes	**no**	**no**	**no**	yes	**no**	yes	yes	7	4
	2	yes	yes	yes	yes	**no**	**no**	**no**	yes	**no**	yes	yes	7	4
	3	yes	yes	yes	yes	**no**	**no**	**no**	yes	**no**	yes	yes	7	4
	4	yes	yes	yes	yes	**no**	**no**	**no**	yes	**no**	yes	yes	7	4
	5	yes	yes	yes	yes	**no**	**no**	**no**	yes	**no**	yes	yes	7	4

Table L. Evaluation of the blank criteria as described by the TS [3] LAB2.

material	stone	As	Ba	Cd	Co	Cr	Cu	Ni	Pb	Sn	V	Zn	criterion fulfilled	criterion not fulfilled
Karbon Quartzite	1	yes	**no**	yes	yes	yes	yes	yes	yes	yes	yes	yes	10	1
	2	yes	**no**	yes	yes	yes	yes	yes	yes	yes	yes	yes	10	1
	3	yes	**no**	yes	yes	yes	yes	yes	yes	yes	yes	yes	10	1
	4	yes	**no**	yes	yes	yes	yes	yes	yes	yes	yes	yes	10	1
	5	yes	**no**	yes	yes	yes	yes	yes	yes	yes	yes	yes	10	1
Basalt	1	yes	yes	yes	yes	yes	**no**	yes	**no**	yes	yes	yes	9	2
	2	yes	yes	yes	yes	yes	**no**	yes	**no**	yes	yes	**no**	8	3
	3	yes	yes	yes	yes	yes	yes	yes	**no**	yes	yes	**no**	9	2
	4	yes	yes	yes	yes	yes	**no**	yes	**no**	yes	yes	**no**	8	3
	5	yes	yes	yes	yes	yes	yes	yes	**no**	yes	yes	**no**	9	2
Greywacke	1	yes	yes	yes	yes	yes	yes	yes	**no**	yes	yes	yes	10	1
	2	yes	yes	yes	yes	yes	yes	yes	yes	yes	yes	yes	11	0
	3	yes	yes	yes	yes	yes	yes	yes	**no**	yes	yes	yes	10	1
	4	yes	yes	yes	yes	yes	yes	yes	**no**	yes	yes	yes	10	1
	5	yes	yes	yes	yes	yes	yes	yes	**no**	yes	yes	yes	10	1
Granodiorite	1	yes	yes	yes	yes	yes	yes	yes	yes	yes	yes	yes	11	0
	2	yes	yes	yes	yes	yes	yes	yes	yes	yes	yes	yes	11	0
	3	yes	yes	yes	yes	yes	yes	yes	yes	yes	yes	yes	11	0
	4	yes	yes	yes	yes	yes	yes	yes	yes	yes	yes	yes	11	0
	5	yes	yes	yes	yes	yes	yes	yes	yes	yes	yes	yes	11	0
Granite	1	yes	**no**	yes	yes	yes	yes	yes	yes	yes	**no**	**no**	8	3
	2	yes	**no**	yes	yes	yes	yes	yes	yes	yes	**no**	**no**	8	3
	3	yes	**no**	yes	yes	yes	yes	yes	yes	yes	**no**	**no**	8	3
	4	yes	**no**	yes	yes	yes	yes	yes	yes	yes	**no**	**no**	8	3
	5	yes	**no**	yes	yes	yes	yes	yes	yes	yes	**no**	**no**	8	3
CUS2	1	yes	yes	yes	yes	yes	yes	yes	yes	yes	yes	yes	11	0
	2	yes	yes	yes	yes	yes	yes	yes	yes	yes	yes	yes	11	0
	3	yes	yes	yes	yes	yes	yes	yes	yes	yes	yes	yes	11	0
	4	yes	yes	yes	yes	yes	yes	yes	yes	yes	yes	yes	11	0
	5	yes	yes	yes	yes	yes	yes	yes	yes	yes	yes	yes	11	0
CUS3	1	*	*	*	*	*	*	*	*	*	*	*	*	*
	2	yes	yes	yes	yes	yes	yes	yes	yes	yes	yes	yes	11	0
	3	yes	yes	yes	yes	yes	yes	yes	yes	yes	yes	yes	11	0
	4	yes	yes	yes	yes	yes	yes	yes	yes	yes	yes	yes	11	0
	5	yes	yes	yes	yes	yes	yes	yes	yes	yes	yes	yes	11	0
LDS	1	yes	yes	yes	yes	yes	yes	yes	yes	yes	yes	**no**	10	1
	2	yes	yes	yes	yes	yes	yes	yes	yes	yes	yes	**no**	10	1
	3	yes	yes	yes	yes	yes	yes	yes	yes	yes	yes	**no**	10	1
	4	yes	yes	yes	yes	yes	yes	yes	yes	yes	yes	**no**	10	1
	5	yes	yes	yes	yes	yes	yes	yes	yes	yes	yes	**no**	10	1

*only 4 Stones were available.

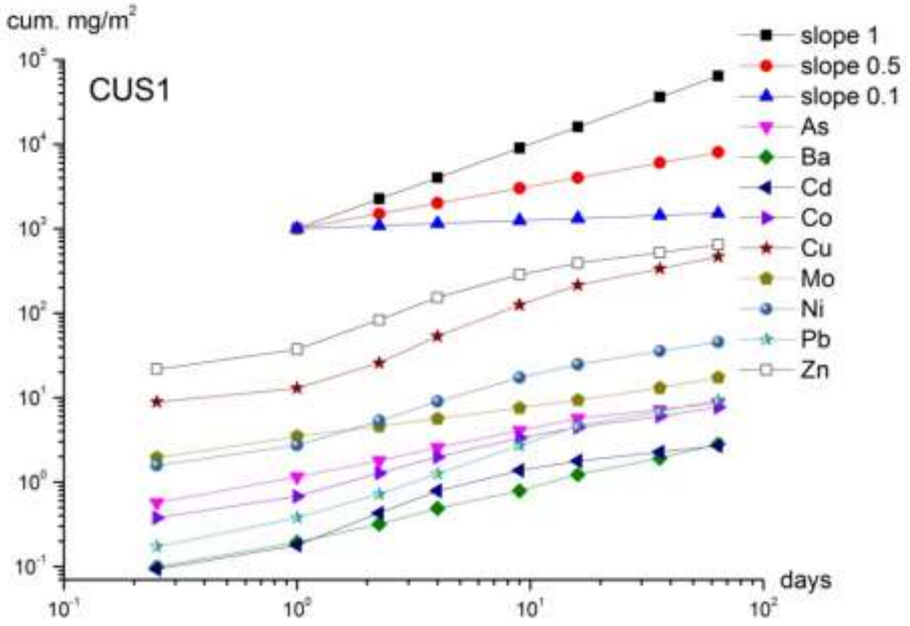

Fig I. log/log diagrams on the release of metal(loid)s from CUS1. Missing analytes were below the respective LoQ (Tables B-H).

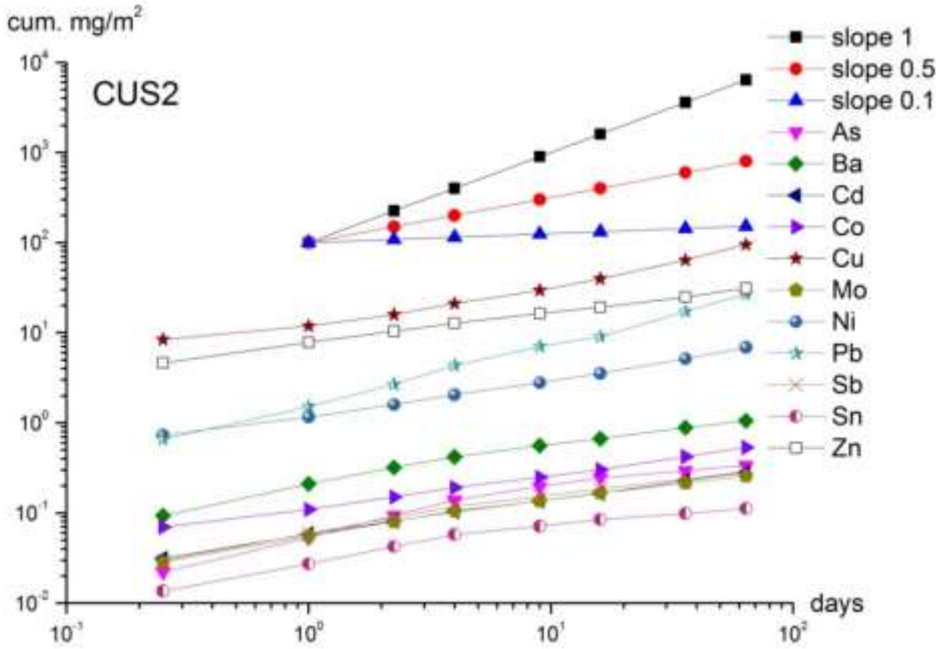

Fig J. log/log diagrams on the release of metal(loid)s from CUS2. Missing analytes were below the respective LoQ (Tables B-H).

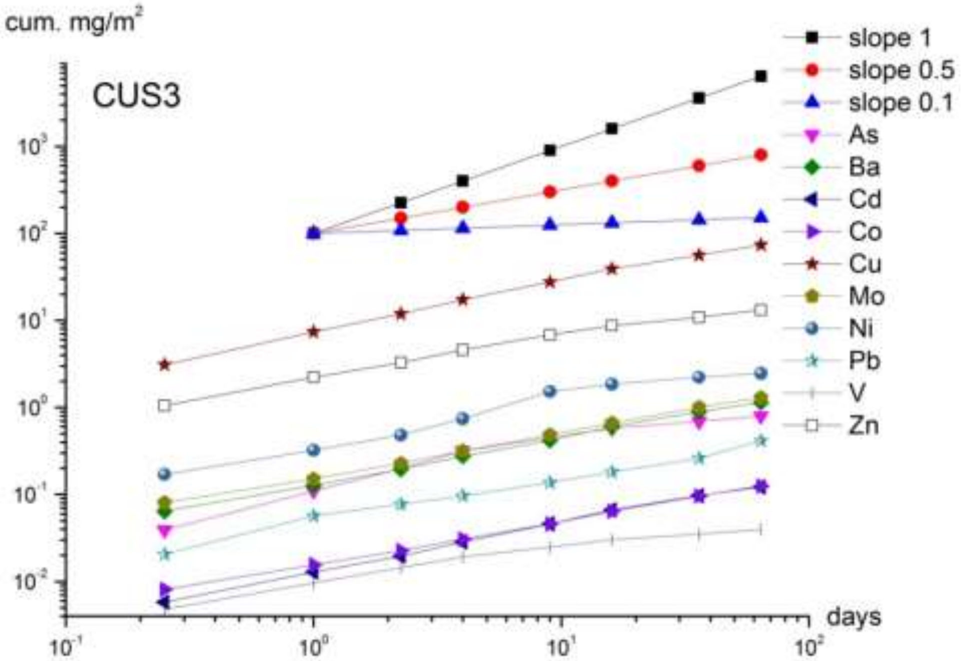

Fig K. log/log diagrams on the release of metal(loid)s from CUS3. Missing analytes were below the respective LoQ (Tables B-H).

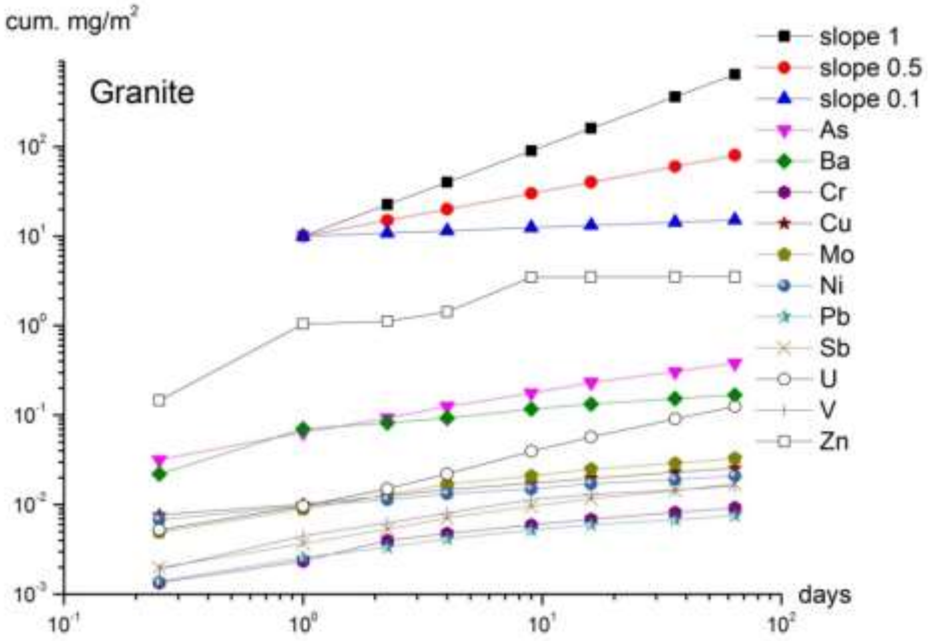

Fig L. log/log diagrams on the release of metal(loid)s from Granite. Missing analytes were below the respective LoQ (Tables B-H).

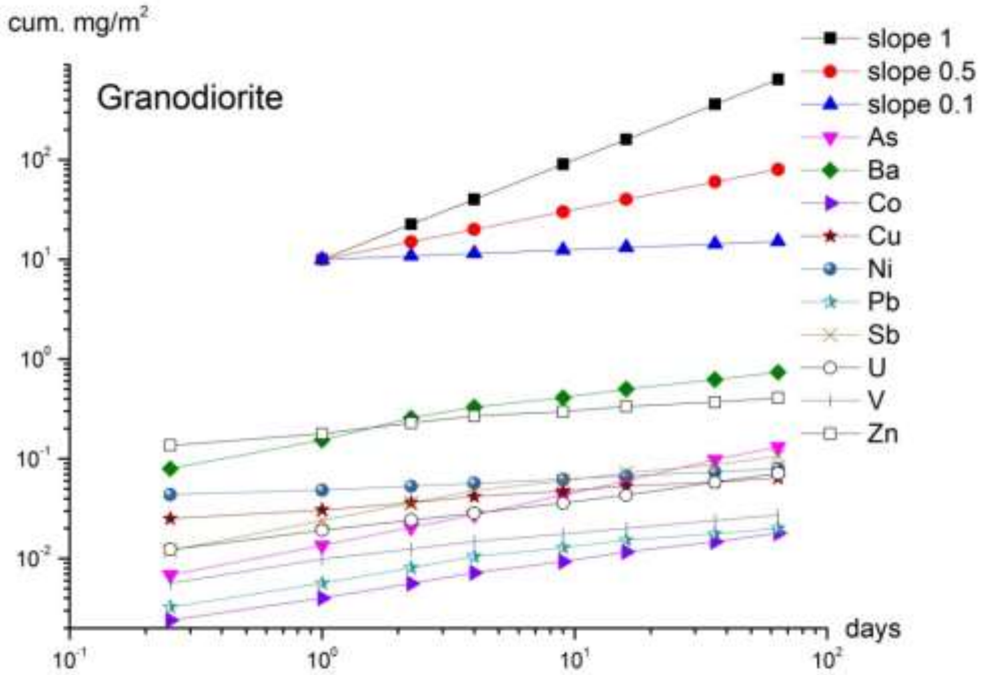

Fig M. log/log diagrams on the release of metal(loid)s from Granodiorite. Missing analytes were below the respective LoQ (Tables B-H).

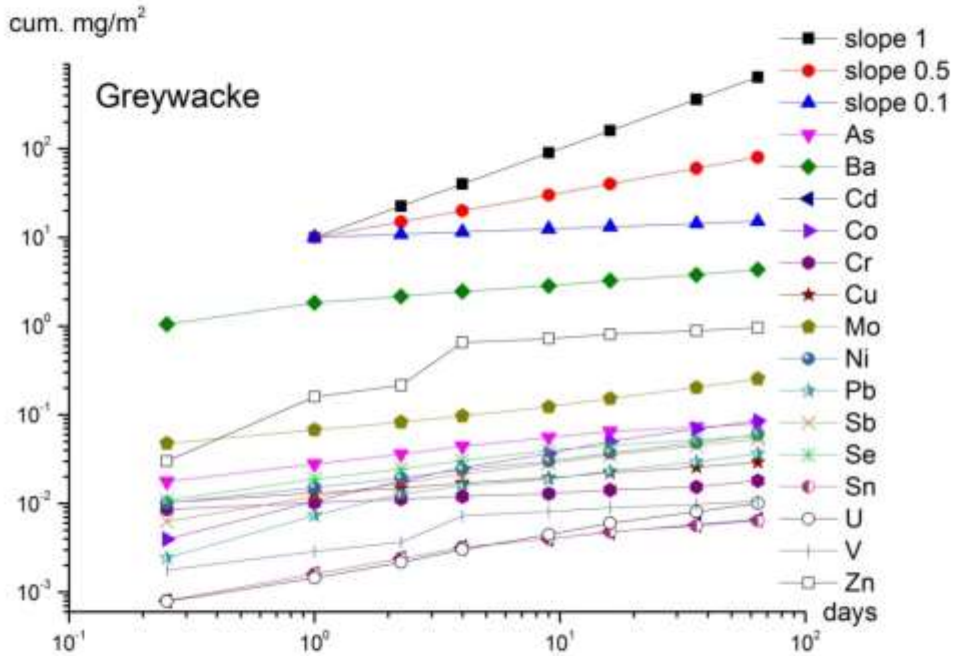

Fig O. log/log diagrams on the release of metal(loid)s from Greywacke. Missing analytes were below the respective LoQ (Tables B-H).

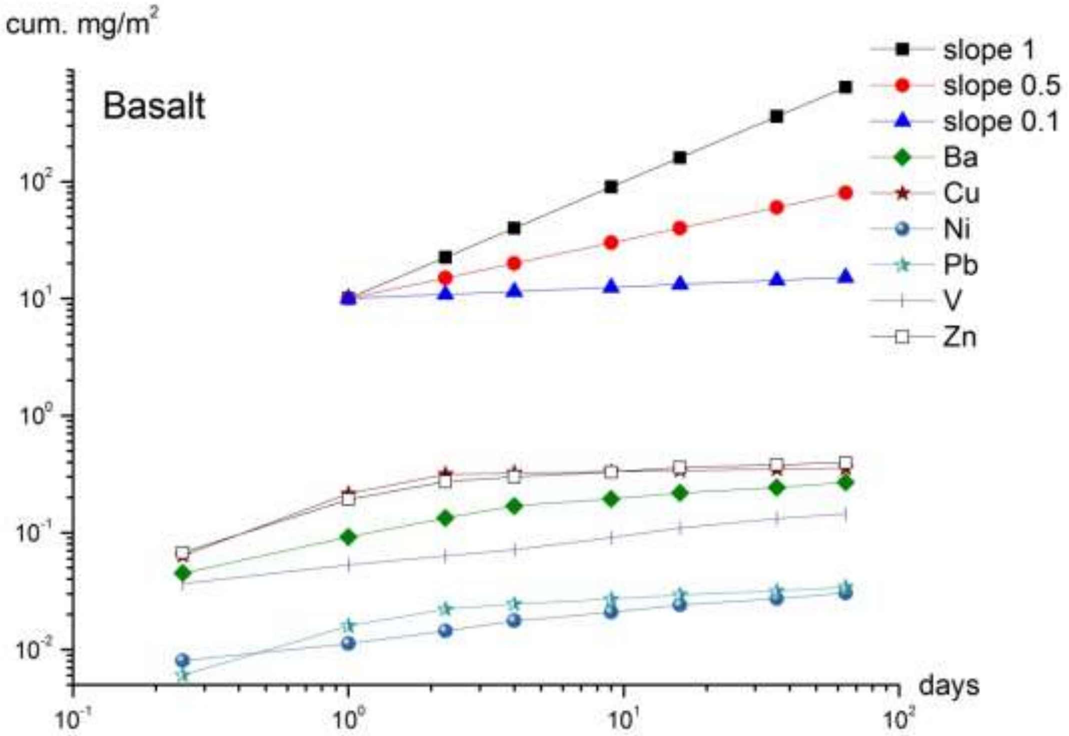

Fig P. log/log diagrams on the release of metal(loid)s from Basalt. Missing analytes were below the respective LoQ (Tables B-H).

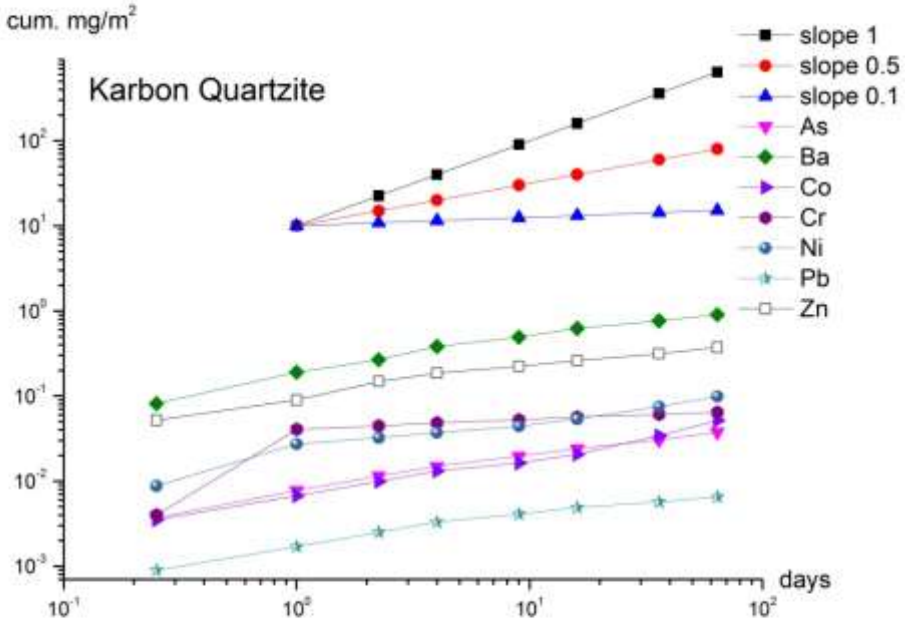

Fig Q. log/log diagrams on the release of metal(loid)s from Karbon Quartzite. Missing analytes were below the respective LoQ (Tables B-H).

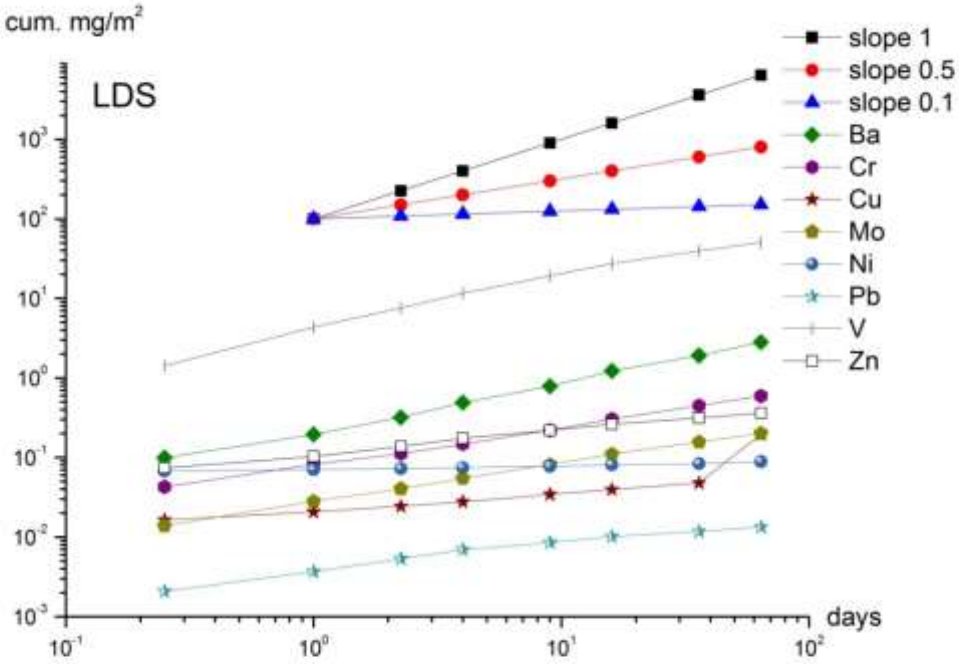

Fig R. log/log diagrams on the release of metal(loid)s from LDS. Missing analytes were below the respective LoQ (Tables B-H).

References

[1] A. Schmukat, L. Duester, D. Ecker, H. Schmid, C. Heil, P. Heininger, T.A. Ternes, Leaching of metal(loid)s from a construction material: Influence of the particle size, specific surface area and ionic strength, Journal of Hazardous Materials, 227-228 (2012) 257-264.

[2] ISO 3696 Water for analytical laboratory use -- Specification and test methods, in, 1987.

[3] DIN CEN/TS 16637-2; DIN SPEC 18046-2:2014-11: Construction products - Assessment of release of dangerous substances - Part 2: Horizontal dynamic surface leaching test; 2014.
